# Supplementary material for: Effectiveness of complex behaviour change interventions tested in randomised controlled trials for people with multiple long-term conditions (M-LTCs): systematic review with meta-analysis
Source: BMJ Open. 2024 Jun 16;14(6):e081104. doi: 10.1136/bmjopen-2023-081104 (PMC11184186; doi:10.1136/bmjopen-2023-081104)
Supplement: Supplementary data [file bmjopen-2023-081104supp005.pdf]

Lead Author<br>Year<br>Country	Participant condition 1<br>Participant condition 2<br>Other conditions	LTC combination	Study n<br>Intervention n<br>Control n<br>Carers n	Intervention length<br>Follow-up timepoints	Primary outcome<br>Timepoint<br>Effectiveness	Category	Quality assessment (RoB-2)	Intervention name, description and modifications	Intervention components<br>Leader<br>Recipient<br>One-to-one or Group<br>Mode of delivery and location<br>Number/length sessions
Ali<br>2020<br>India	Depression<br>Type 2 diabetes	Physical-Mental	404<br>196<br>208	12 months<br>Baseline, 6, 12, 18, 24 months	Composite depression and clinical<br>24 months<br>Yes	Collaborative care	Low	Collaborative care intervention targetting patient-, clinician-, and system-level improvements. Administered PHQ-9 to monitor depression, reviewed glucose logs, and counseling toward achieving treatment goals. Intergrated decision making tool for HCPs within electronic health record system	Psychiatrist and diabetologist and care co-ordinators<br>Person with depression and T2DM<br>One-to-one<br>Face-to-face in clinic or telephone<br>Every 2-4 weeks for 12 months

Aragones<br>2019<br>Spain	Depression and Chronic Musculoskeletal Pain	Physical-Mental	41<br>21<br>20	3 months<br>Baseline, 3, 6, 12 months.	Depression<br>12 months<br>Yes	Self-management	Low	DROP (DepRessiOn and Pain) programme:<br>1. Optimised management of major depression through computerised clinical guideline to aid decision making<br>2. Care management of patient pain through calls with psychologist<br>3. Psychoeducational intervention group programme, with discussions and homework covering: understanding pain; managing emotions and attention; basic relaxation techniques; cognitive restructuring strategies; problem-solving; setting life goals; relationships between pain and physical activity; healthy posture and sleep; maintenance of the strategies learned; and a relapse plan	Care co-ordinator (psychologist)<br>Primary care physicians and people with conditions<br>Primary Care Centres<br>Face-to-face - 9 weekly 2 hour group sessions<br>Telephone - Monthly for first 3 months and then every 3 months for next 9 months

Barley, 2014, UK	Coronary heart disease<br>Depression	Physical-Mental	81<br>41<br>40	6 months.<br>Baseline, 1, 3, 6, 12 months.	N/A Feasibility and acceptability	Self-management	Low	Personalised Care: the nurse case manager conducts a standardised, face to face, biopsychosocial assessment. Patients then identify up to three problems which contribute to their depression and they want to address. The nurse-case managers provide information, sign-post to existing resources and use evidence based behaviour change techniques to help patients set and achieve goals. The underlying intention of the intervention is to increase the patient's self-efficacy to achieve their desired goals. The action plan is recorded in a 'personalised health plan' which the patient holds.	Nurse<br>Person with CHD and depression<br>One-to-one<br>Face-to-face and telephone<br>1 hour session<br>Weekly 15 minute phone calls for 6 months
Barrowclough, 2001, UK	Schizophrenia<br>Substance Abuse disorder	Mental-Mental	33 patient carer dyads	9 months.<br>Baseline, 9 months and 12 months.	Functioning 9 and 12 months<br>Yes	Cognitive and/or Behavioural Activation	Some concerns	Allocated a family support worker from Making Space, providing information, giving advice on benefits, advocacy, emotional support, and practical help. The integrated treatment program attempted to combine three treatment approaches: motivational interviewing, individual cognitive behavior therapy, and family or caregiver intervention.	Family support worker<br>Patient and primary care giver dyad<br>Face-to-face<br>Home or clinic<br>23 weekly sessions followed by 6 biweekly sessions and 10-16 family sessions

Barr Taylor, 2003, USA	Diabetes<br>And one or more of:<br>Hypertension<br>CVD<br>Dyslipidemia	Physical-Physical	169<br>84<br>85	12 months<br>Baseline, 12 months	Clincal outcome (HbA1c)<br>12 months<br>Yes	Collaborative care	Low	Nurse care management system. Initial consultation to review medical, lifestyle, and social status, and develop a self-management action plan. Group classes followed a workbook specifically created for the program and included some didactic material, although the focus was on group discussion, participation and problem-solving. Follow-up calls were used to review patient goals, medication use, glucose/BP monitoring, and self-management activities. Higher levels of depression and alcohol problems were also addressed. Algorithims were used throughout for medication managament.	Nurse care manager<br>Patient<br>Group classes once a week for 4 weeks (1-2 hours)<br>8 Follow-up telephone calls lasted 15 minutes, over 9 months.

Battersby, 2013, Australia	Alcohol use disorder AND one or more of:<br>-Cardiovascular<br>-Musculoskeletal<br>-Gastrointestinal<br>-Respiratory<br>-Diabetes<br>-Skin conditions<br>-Cancer<br>-Genitourinary<br>-Alcohol dependence<br>-PTSD<br>-Major depression<br>-Generalised Anxiety Disorder<br>-Panic Disorder	Physical-Mental	77<br>46<br>31<br>SCDSMP course = 22 veterans and 15 of their partners from intervention group	9-month intervention. Seen at 1, 3, 6 and 9 months, with telephone calls at 4.5 and 7.5 months<br>Follow-up measures at 18 months	Behaviour change (AUDIT)<br>9 months<br>Yes	Self-management	High	Flinders Programme<br>Targeted self-management support to assist the patient to achieve improved health outcomes over a period of 12 months. A health professional is trained to tailor the interventions to meet the persons needs whilst simultaneously motivating the patient to achieve their medical, psychosocial, or lifestyle risk factor goals. Veterans offered SCDSMP course (Stanford School of Medicine) if considered appropriate - this builds the person's self-efficacy by teaching skills in problem-solving, decision-making, resource utilisation, managing the patient–provider partnership, action planning and emotional management	Research nurse (RN)<br>Veterans<br>One-to-one<br>Face-to-face: begins with developing care plan, follow-up visits monitor outcome of this plan<br>SCDSMP: 6 weekly face-to-face group sessions (2.5 hours each) delivered jointly by research officer and veteran peer leader to veterans and their partners if desired

Blank 2011, USA	HIV<br>Schizophrenia Spectrum Disorder/ Affective Disorder/ Other serious mental ilness	Physical-Mental	238<br>128<br>110	12 months. Baseline, 3 months, 6 months, 12 months<br>Follow-up at 24 months.	Clinical (Viral load)<br>12 months<br>No	Self-management	Some concerns	Preventing Aids Through Health (PATH)<br>Driven by theory of reasoned action; helps patients actively cope with barriers to medication adherence and promote participants self-care abilities. The protocol includes one face-to-face meeting a week as a minimum with basic intervention (psychoeducation, pillboxes, beeping watches). The intervention was then cascaded to suit the needs of the individual if adherence fell - this was a gradual increase in intensity and included activation of social networks, followed by use of beepers with alphanumeric displays, then prepaid cellular phones to encourage participants to follow their regimen, then the final step was directly observed therapy.	Advanced Practice Nurse (same nurse each session where possible)<br>Patient<br>One-to-one<br>Community-based: weekly face-to-face home visits from the APN
Boeschoten, 2017, The Netherlands	MS<br>Depression<br>Anxiety	Physical-Mental	171<br>85<br>86	5 to 10 weeks.<br>Baseline, within a week of intervention, 4 months	Depression (BDI-II)<br>5-10 weeks and 4 months<br>No	Cognitive and/or Behavioural Activation	Low	"Minder Zorgen" (Worry Less) - a guided self help intervention using Cognitive Behavioural Therapy with a focus on coping skills. Five modules with texts, examples and assignments that patients could access online from their own computer. This intervention was an existing IPST and adapated for MS patients.	Psychologists and masters student<br>Patient<br>One-to-one<br>Online - digital<br>One module a week for 10 weeks

Bogner, 2008, USA	Hypertension<br>Depression	Physical-Mental	64<br>32<br>32	4 weeks.<br>Baseline, 2, 4 and 6 weeks.	Depression (CES-D)<br>6 weeks<br>Yes	Collaborative care	Low	Integrated Care Intervention. Involved education about depression and hypertension, emphasizing the importance of controlling depression to manage hypertension; encouragement and relief from stigma; helped to identify target symptoms for both conditions; explained the rationale for antidepressant and antihypertensive medication usage; assessed for side-effects and assisted in their management; assessed progress; assisted with referrals; and monitored and responded to life-threatening symptoms.	Integrated care manager (ICM)<br>Patient<br>One-to-one<br>3, 30-minute in-person sessions and 2, 15-minute telephone-monitoring contacts during the 4 weeks

Chwastiak, 2018, USA	Type II diabetes<br>Schizophrenia<br>Schizoaffective disorder<br>Bipolar disorder<br>Major depressive disorder with psychosis	Physical-Mental	35<br>18<br>17	3 months. Baseline, 3 months.	Clinical (HbA1c)<br>3 months<br>N/A test not completed	Collaborative care	Low	CMHC-based collaborative care model, adapted from the primary care–based TEAMcare model and adapted for patients with psychosis.<br>Initial 60-min nurse care manager visit for a comprehensive health assessment and an individualized health plan and then 30-minute visits for the support of chronic illness self-management (including medication adherence, healthy nutrition, and regular physical activity) every other week for 12 weeks. Nurses used motivational interviewing and behavioural activation to address barriers to self management. Diabetic education materials were modified to address needs of patients with psychosis.	A CMHC nurse care manager, a CMHC psychiatrist, the advanced practice registered nurse who provided primary care onsite at the CMHC, and an endocrinologist consultant.<br>Patients<br>One-on-one<br>Hospital clinic

Cummings, 2019, USA	Type 2 diabetes<br>Diabetes-related distress or depression	Physical-Mental	139<br>67 (CBT=48; Lifestyle=19)<br>72	12 months.<br>Baseline, 6 months (to adjust intervention if necessary), 12 months.	Clinical (HbA1c)<br>12 months<br>No (approached p=0.06)	Cognitive and/or Behavioural Activation	Low	CBT (plus usual care): Session content used cognitive techniques to identify and challenge general and diabetes-specific cognitive distortions that result in maladaptive behavior, in combination with behavioral techniques, including behavioral activation and specific behavior change strategies related to diabetes and/or mood. Those with more intermediate concerns were also provided with problem solving therapy focusing on effective coping and adaptive problem-solving skills.<br>Small Changes Lifestyle Coaching (plus usual care): Focused on lifestyle modifications to improve diabetes and mood.<br>Patients were reevaluated at 6 months and either entered a maintenance phase, or switched to CBT or SCLC.	SCLC - nurse care manager; CBT - a psychologist and clinical health psychology doctoral student; and a community health worker provided navigation and social support.<br>Patient.<br>One-on-one.<br>12 tailored sessions 30-60 minutes long for the first 6 months of either:<br>CBT: Face-to-face or over the telephone if necessary.<br>Lifestyle: over telephone

Druss, 2018, USA	Medical illness<br>Diabetes<br>Heart disease<br>Hyperlipidemia<br>Hypertension<br>Arthritis<br>Hepatitis<br>Asthma/COPD<br>HIV<br>SMI<br>Schizophrenia/ schizoaffective<br>Bipolar disorder<br>Depression<br>OCD<br>PTSD	Physical-Mental	400<br>198<br>202	3 months. Baseline, 3 months, 6 months.	Quality of life<br>6 months<br>Yes	Self-management	Some concerns	Health and Recovery Peer Programme (HARP)<br>The program builds on the Chronic Disease Self-Management Program (CDSMP), a 6-session health education program. It provides skills that help patients become more effective self-managers including modeling of healthy behaviors, problem-solving, reinterpretation of symptoms, and training in specific disease management techniques. Action plans help develop self-efficacy and build to long-term health goals.<br>Modifications of CDSMP to HARP addressed specific patient, provider, and community level determinates of health in patients with comorbid medical and serious mental disorders.<br>Materials were added addressing the mental health impact of chronic conditions and dealing with social isolation.	Two certified peer specialists led each session.<br>Patient.<br>Six 2.5 hour face-to-face sessions in groups of 6-10, with one-on-one peer coaching meetings held in between sessions for reinforcement.

Ell, 2010, USA	Type 1 or 2 diabetes Depression	Physical-Mental	387 193 194	12 months. Baseline, 6, 12, and 18 months.	Depression (SLC-20) 6-12-18 months Yes	Collaborative care	Low	Multifaceted Diabetes and Depression Program (collaborative care intervention). Patients chose problem-solving therapy or antidepressant, and a stepped-care algorithm was used to ensure patients received treatment consistent with their preference, clinical presentations and responses over time. Usual care was enhanced by providing patients with depression educational pamphlets and a community resource list.	1) PST provided by bilingual graduate social work diabetes depression clinical specialists (DDCSs) and/or antidepressant medications prescribed by the treating primary care physician. Patient. One-to-one but support group sessions available.

Ell, 2017, USA	Depression<br>Diabetes OR heart disease OR both	Physical-Mental	348<br>178<br>170		6 weeks (plus 3 monthly boosters).<br>Baseline, 6 months, 12 months.	Depression (PHQ-9)<br>12 months<br>No	Self-management	Some concerns	A-Helping-hand (AHH)<br>Psychoeducational intervention following a problem-solving framework, using self-care management of chronic care conditions as a specific skill-building focus with review of healthy behaviors outlined in the written materials provided to patients. Intervention provided problem-solving modeling and opportunities for patients to practice problem solving skills: 6 tasks included engagement, problem formulation, education, action planning and evaluation.<br>Study participants in both groups were given depression educational brochures, depression photo novels, and materials on specific chronic illness treatments and self-care.	3 promotoras (trained and supervised by the research team) were added to a standard team of physician, nurse, and medical assistant.<br>Patient.<br>One-to-one.<br>Six sessions over six weeks administered face-to-face (or telephone if preferred) at the patients home/any other setting preferred by patient, followed by 3 monthly booster sessions for reinforcement.

Fisher, 2020, Canada	At least 3 chronic conditions<br>Most common:<br>Cardiovascular<br>Kidney/Urogenital<br>Arthritis<br>Gastrointestinal<br>Endocrine<br>Hearing/Vision	Physical-Physical	59<br>30<br>29	6 months.<br>Baseline and 6 months.	SF-12 Physical<br>6 months<br>No	Self-management	High	Self-management intervention.<br>An individualised care plan was developed to help self-management of chronic conditions, and caregivers were actively consulted in all aspects of care. Focus on client building on their strengths and abilities to enhance self-efficacy and achieve personal goals.<br>Adapted from two previous community-based self-management interventions for older adults (targeting diabetes and stroke) to target multi-morbidities.	Care Coordinator, Registered Nurse, Physiotherapist, Occupational Therapist, and Personal Support Worker<br>Patient and informal/family caregiver.<br>One-to-one<br>1. Minimum of one in-home visit from CC and 3 from PT/OT.<br>2. Monthly case conferences with provider team to discuss ongoing care plan.<br>3. Ongoing case management by CC's which involved system navigation and facilitating participant access to appropriate health and social services.

Garvey, 2015, Ireland	43 chronic coniditions identified, most common:<br>Arthritis<br>Congestive cardiac failure<br>Diabetes<br>Depression<br>Hypertension	Physical-Physical	50<br>26<br>24	6 weeks.<br>Baseline and 6 weeks	N/A (participation)<br>6 weeks<br>yes	Self-management	Some concerns	Occupational therapy led self-management support programme (OPTIMAL).<br>Occupational therapy focus with peer support; goals were discussed and revised at each session, considering patient preference. Intervention components included: self-management; fatigue and energy management; managing stress/anxiety and maintaining mental health/wellbeing; keeping physically active; healthy eating; managing medications; effective communication strategies; goal setting.<br>Based on the Stanford Chronic Disease Self-Management Programme: key adaptations were an occupational therapy focus, groups being professionally led and a clear focus on the specific challenges of multimorbidity.	Facilitated and led by local community-based occupational therapists.<br>Patient.<br>Group.<br>Weekly 3-hour meetings for 6 weeks held in local community health centres.

Goldberg, 2013, USA	Schizophrenia spectrum disorder<br>OR bipolar disorder<br>AND one or more chronic condition, including:<br>Diabetes<br>Arthritis<br>Respiratory disease<br>Cardiovascular disease	Physical-Mental	63<br>32<br>31	3 months<br>Baseline, 3 months and 5 months	SF-12 Physical<br>3 months<br>Yes	Self-management	Some concerns	Living Well.<br>Sessions focused on action planning, peer feedback, support, modeling, problem solving, training in specific disease management techniques and the application of these skills to the topics of nutrition, exercise, sleep, medication management, addiction behaviours.<br>Modified from CDSMP for patients with serious mental illness as follows - delivery by two mental health peers OR mental health provider and peer; increased length of intervention; action plan tracker and health workbook; extra module on communication with HCP's; extra topics on mental illness; two monthly booster sessions after intervention.	Six groups - four were cofacilitated by two mental health peers who had at least one chronic condition, two were cofacilitated by a mental health professional and a peer.<br>Patients.<br>Group.<br>Weekly 60-75 minute face-to-face sessions and two monthly booster sessions post-intervention.<br>Clinics
Goorden, 2017, The Netherlands	Major depressive disorder<br>AND one or more chronic physical condition:<br>Diabetes<br>COPD<br>IBS<br>Heart failure	Physical-Mental	81<br>42<br>39	12 months.<br>Baseline, 3, 6, 9, 12 months.	N/A cost effectiveness<br>12 months	Collaborative care	High	Collaborative Care<br>Guided self-help and problem-solving treatment, prescription of anti-depressant medications according to an algorithm, and consultations with psychiatrist if necessary.	Provided by a team consisting of patient, consultant psychiatrist nurse, and consultant-liaison psychiatrist.<br>Patient.<br>One-to-one.<br>Face to face at outpatient clinic of general hospital.

Griva, 2019, Singapore	Diabetes<br>End stage renal disease	Physical-Mental	44<br>20<br>24	3 weeks.<br>Baseline, 3 months	Clinical endpoint (HbA1c)<br>3 months<br>No	Self-management	Low	Combined Diabetes and Renal Control Trial (C-DIRECT) Intervention (plus usual care). Centered around self-management strategies. Patients chose a topic to focus on at each session. Session 1: feedback on Hba1c; information/advice on chosen topic; goal setting; food record assignment. Session 2: review goal setting and problem solve any barriers; revise goals as needed; review food record and provide support if necessary; choose topic (information/advice and new goal setting). Session 3: review goals and problem solve barriers; revise goals as needed; choose topic (information/advice and new goal setting). Provide additional advice and resourse links to go away with.	DM link nurse.<br>Patient.<br>One-to-one.<br>3 weekly sessions 30-60 minutes long, delivered at patient bedside at dialysis centre upon cannulation and connection to haemodialysis machine.

Guo, 2020, China	Atrial Fibrilliation AND<br>Congestive heart failure<br>Hypertension<br>Diabetes<br>Prior ischemic stroke<br>Vascular disease<br>Coronary artery disease<br>Peripheral arterial disease<br>Renal dysfunction<br>Liver dysfunction<br>Prior bleeding<br>Prior brain bleeding<br>Hyperthyroidism<br>Cardiomyopathy	Physical-Physical	40 hopsitals randomised (20 to each arm)<br>3324<br>1646<br>1678	12 months<br>Baseline, 6 and 12 months.	N/A<br>composite<br>hospital<br>12 months<br>Yes	Collaborative care	Low	mobile Atrial Fibrillation Application (mAFA).<br>Installed on smart phones.<br>Provided clinical decision support tools for doctors to facilitate treatment recommendations, educational materials, patient self-care, and structured follow-up, to support AF management: Avoid stroke (tracking bleeding events/trends; managing treatment and anticoagulant use), Better symptom management (tracking symptoms, monitoring cardiac rhythm using smart watches), Cardiovascular and other comorbidities risk management (tracking BP and lifestyle factors). The app encouraged patient engagement through participation in educational programs, provision of informative articles, videos, game playing, etc.	Doctors.<br>Doctors and patients.<br>One-to-one.<br>Followed up at outpatient clinics.

Hernandez-Quiles, 2021, Spain	Advanced heart/lung failure/both.<br>AND one of:<br>Hypertension<br>Heart Failure<br>Atrial fibrillation<br>Dyslipemia<br>Diabetes<br>CHD<br>COPD<br>Chronic kidney disease<br>Obesity<br>Chronic anemia<br>Valvular heart disease<br>Pulmonary hypertension<br>Sleep apnea hypopnea syndrome<br>Hypothyroidism<br>Depression	Physical-Physical	510<br>255<br>255		180 days<br>0, 15, 45, 90, 180 days.	N/A hospital admissions<br>45, 90 and 180 days<br>Yes	Self-management	Low	TELECARE (plus usual care).<br>Usual care was frequent clinical and bio-parameters self-checks (BP, heart rate, oxygen saturation, weight, and blood glucose)Patients were provided with educational material and instructed how to recognize exacerbation symptoms. For the intervention group, a synchronous monitoring equipment was added for biological and medical questionnaires so data was available in real time for healthcare team. An alarm system was added to induce a healthcare response if there was variation in data or missing data.	Health care team<br>Patient tracked own data.<br>One-to-one.<br>Patients inserted data daily for first 15 days (frequency was then altered depending on stability and ocurrence of incidents).

House, 2018, UK	Type 2 diabetes<br>Obesity<br>Intellectual disability	Physical-Physical	82<br>41<br>41	4 months<br>Baseline, 6 month follow-up (reduced to 4 because of project deadlines)	Clinical endpoint (HbA1c)<br>4-6 months<br>No	Self-management	Some concerns	Supported self-management<br>The intervention emphasized realistic goal setting, identifying resources and barriers likely to influence success in reaching goals, and regular self-monitoring of goal attainment.<br>Four components: (1) Establishing the participant's daily routines and lifestyle; (2) Identifying all supporters and helpers and their roles; (3) Setting realistic goals for change; (4) Monitoring progress against agreed upon goals.<br>Materials: for nurses - weekly timetable, charts to record friends/family/helpers, charts to complete in collaboration with participant; for participants - OK Diabetes board to record goals and visual prompts, a written action plan, tear off slips to record daily actions; for supporters/helpers - information sheet on study, card summarising their role in helping the person with diabetes.<br>The intervention was modelled on existing self-management interventions, with adjustments [illegible]	Diabetes specialist nurse.<br>Patient (73% had their supporter present for at least one session).<br>One-to-one.<br>4 sessions, most (92%) took place in participants homes, ranging from 13-95 minutes (typical total intervention time was 2 hours).

Jackson, 2021, Nigeria	HIV<br>Hypertension<br>Other:<br>Diabetes<br>Peptic ulcer disease<br>Renal disease<br>Hypertensive heart disease<br>Osetoarthritis<br>Haemorrhoids<br>Benign prostatic hyperplasia	Physical-Physical	206<br>103<br>103	12 months. Baseline, 6 months, 12 months.	Clinical outcomes (Blood pressure)<br>12 months<br>Yes	Self-management	Low	Pharmaceutical care (PC) (plus routine care).<br>Routine care - BP taken by nurse, physician prescribes medication/orders lab tests, meeting with adherence counsellor, then medication/ counselling from pharmacist.<br>Intervention also exposed to PC from research pharmacist. This involved structured education/ counselling focused on self-monitoring of BP, healthy eating, maintaining an ideal body weight, proper foot and dental care, healthy coping, use and storage of medications; lifestyle modifications with emphasis on the dietary approaches to stop hypertension (DASH) diet, physical activity, smoking cessation, moderation of alcohol intake; reviewing date of the next appointment and prescription with the patient, expected side effects of the medications, and how to prevent/ manage such side effects.	Research pharmacist.<br>Patient.<br>One-to-one.<br>Face-to-face bi-monthly appoointments at hospital, as well as two text messages per month.

Kamradt, 2019, Germany	Type 2 diabetes and<br>Two or more of the following:<br>Coronary heart disease<br>Chronic pain<br>Depression<br>Chronic heart failure<br>COPD<br>Artherosclerosis<br>Cerebrovascular disease	Physical-Physical	495<br>252<br>243	9 months.<br>Baseline, 9 months.	Quality of life<br>9 months<br>No	Collaborative care	Some concerns	Care management intervention. Embedded in a regional network of primary care practices. It included two home visits with structured assessments of clinical and social needs, aiming to reduce barriers to patient's access of support and healthcare systems. Frequent telephone monitoring was also used to improve and sustain changes in self-care behaviour, and give feedback related to patients' control of diseases and current (self-care) activities to reinforce positive behavioral changes.	Specially trained medical assistants, physicians specialising in family/internal medicine, and net-care managers.<br>Patient.<br>One-to-one.<br>Two home visits and 15 structured telephone monitoring contacts over 9 months.

Kanwal, 2018, USA	HCV Depression	Physical-Mental	309<br>156<br>153	6 months<br>Baseline, 6 months, 12 months.	Depression (SLC-20)<br>12 months<br>Yes	Collaborative care	Some concerns	Hepatitis C Translating Initiatives for Depression Into Effective Solutions (HEPTIDES)<br>The intervention included: participant education and activation, assessment of treatment barriers and possible resolutions, depression symptom and treatment monitoring, substance abuse monitoring, and instruction in self-management. The intervention used a stepped-care model for depression treatment, with five steps: self-management education, depression care team treatment suggestions, pharmacotherapy suggestions, combination pharmacotherapy and specialty mental health counseling, and referral to specialty mental health.	Depression care manager, pharmacist and psychiatrist. They convened once a week and communicated with HCV and mental health clinicians at each hospital site to discuss treatment and make decisions.<br>Patient.<br>One-to-one via telephone delivered by DCM, every 2 weeks during acute treatment and every 4 weeks during self-management education or continuation treatment.

Katon, 2004, USA	Diabetes Depression	Physical-Mental	329<br>164<br>165	3-6 months. Baseline, 3 months, 6 months, 12 months.	Depression (SLC-90)<br>6 and 12 months<br>Yes	Collaborative care	Low	Collaborative care.<br>Individualised, stepped-care depression treatment programme designed to improve quality of care and outcomes of depression. Patients were offerd an intial choice of two treatments: antidepressant medication or problem solving treatment (PST). If patients still had persistent depressive symptoms (< 50% decrease in severity based on the PHQ-9) 10 to 12 weeks after initial treatment, they could (1) switch to a second antidepressant; (2) switch to the alternative treatment (from PST to medication or vice versa); (3) receive augmentation with PST or medication with the first treatment they had received; or (4) receive a psychiatric consultation. If there was still no improvement after a further 8 to 12 weeks, referral to speciality care was made. Once patients reached a significant decrease in clinical symptoms (< 50% decrement in symptoms), the nurse began continuation phase	Depression clinical specialist nurse in collaboration with the primary care physician.<br>Patient.<br>One-to-one.<br>Initial 1-hour visit followed by twice-a-month, half-hour appointments (telephone and in person) for 12 weeks followed by monthly scheduled telephone contacts for continuation phase treatment.

Katon, 2010, USA	Depression Diabetes/Coronary heart disease/both	Physical-Mental	214<br>106<br>108	12 months. Baseline, 6 months, 12 months.	Composite depression and clinical 12 months Yes	Collaborative care	Low	Collaborative care management. The intervention combined support for self-care with pharmacotherapy, aiming to manage depression and improve glycemic, BP and lipid control. Patients worked collaboratively with nurses and primary care physicians to establish individualized clinical and self-care goals. Using motivational and encouraging coaching, nurses helped patients solve problems and set goals for improved medication adherence and self-care. Patients also received self-care materials and self-monitoring devices. Nurses monitored the patient's progress and medications were adjusted where necessary according to protocol. Once target levels were achieved, a maintenance plan was developed that included stress reduction, behavioral goals, continued use of medications, and identification of prodromal symptoms associated with worsening depression and glycemic control.	Nurses, in collaboration with primary care physicians.<br>Patient.<br>One-to-one.<br>Visits every 2 to 3 weeks at the patients primary care clinic; once maintenance plan was in place. Patients whose disease control worsened were offered follow-up visits and telephone calls with increased treatment intensity.

Khunti, 2021, UK	Conditions accepted in inclusion criteria:<br>Cardiovsacular<br>Asthma/COPD<br>High dependency/ LTC's<br>Depression<br>Musculoskeletal	Physical-Physical	353<br>180<br>173	6 months.<br>Baseline, 6 months, 12 months.	Behaviour change (physical activity)<br>12 months<br>Yes	Self-management	Low	Movement through Active Personalised engagement (MAP) programme.<br>Four sessions with person-centred self monitoring and goal setting. The sessions focused on increasing PA, and addressed key non-disease-specific self-management challenges and themes (mastering emotions, managing treatments, communication within health care). Increasing PA was further facilitated and encouraged by providing resistance bands in order to complete resistance training at home and pedometers to help track PA. Regular reminder and motivational texts were sent to encourage long-term behaviour change.	Trained facilitator.<br>Patient.<br>Group.<br>1.5 hour face-to-face sessions delivered at two week intervals in local community.<br>Texts were sent daily during the 8 weeks of sessions up until 2 weeks after the 6-month timepoint, then 3 times a week over the remainder of the 12 month period.

Koesoemadinata, 2021, Indonesia	Tuberculosis<br>Diabetes	Physical-Physical	108<br>60<br>48	6 months.<br>Baseline, 6 months.	N/A patient knowledge<br>6 months<br>No	Cognitive and/or Behavioural Activation	Some concerns	Structured counselling/education. At each visit, blood glucose level was measured, and DM medication was adjusted according to a structured algorithm. Educational messages were discussed according to a specified checklist using flip charts. Topics included: TB: Cause, transmission, treatment compliance, possible side effects; What is DM? What are the symptoms? What can be done?; Blood glucose measurement and recording; Importance of glycaemic control; Metformin and insulin; Hyper- and hypoglycaemia; The importance of smoking cessation; DM and TB together; Healthy lifestyle: diet, exercise, and smoking cessation; Management after the TB treatment is completed. A leaflet was also provided to patients with information regarding recommended food types and amounts.	Doctor.<br>Patient.<br>One-on-one.<br>Face-to-face in a research clinic at weeks 1, 2 and 4, then monthly until 6 months.

Kroenke, 2009, USA	Musculoskeletal pain<br>Depression	Physical-Mental	250<br>123<br>127	12 months. Baseline, 1, 3, 6, and 12 months.	Depression (SLC-20)<br>12 months<br>Yes	Collaborative care	Low	Stepped Care for Affective Disorders and Musculoskeletal Pain (SCAMP).<br>Step 1 (weeks 1-12): Optimised antidepressant therapy. Adjustments in medication as per an algorithim to optimise antidepressant management.<br>Step 2 (weeks 13-26): Pain self-management programme. Focus on increasing self-efficacy, social support to self-manage pain, problem solving techniques, behavioural change strategies, learning about chronic pain and receiving feedback from nurse.<br>Step 3 (weeks 27-52): continuation phase. Evaluation. The care manager assessed current self-management strategies and assisted patients with new behavioral plans. Antidepressant dose could be increased, or if necessary a referral to a psychiatrist offered.	Nurse care manager and physician depression specialist.<br>Patient.<br>One-to-one.<br>5 inperson contacts (baseline, 6, 12, 16, and 20 weeks) and 8 telephone contacts (1, 3, 9, 14, 18, and 22 weeks, and at 8 and 10 months). Extra contacts could occur depending on treatment changes or a patient’s clinical needs.

Lear, 2021, Canada	2 or more of:<br>Diabetes<br>Heart failure<br>Ischemic heart disease<br>Chronic kidney disease<br>COPD	Physical-Physical	230 (219 - one withdrew after randomisation)<br>117 (116 - see above)<br>113	Two years. Baseline, 12 months, 24 months.	N/A Hospital admissions<br>24 months<br>No	Self-management	Low	Internet chronic disease management (CDM).<br>Participants used the CDM website to complete daily symptom reports which asked questions about disease-specific symptoms and biometric data, which generated alert for specific scenarios or targets not being met. The nurse would then call to discuss/readjust targets, and support patient self-management or recommend follow up with primary care practicioner/hospital referral if necessary. Every 8 weeks, participants answered a lifestyle questionnaire, and alerts were sent based on thresholds for these questions. If necessary the nurse gave a referral to a dietitian/exercise specialist or use of a psychosocial support workbook. Participants had access to a public forum, graphical presentations of their biometric data overlaid with relevant alerts, their action plan, and external online resources.	Coordinated care between participant, their primary care practicioner, and a nurse managing the CDM programme (supported by a dietitian and an exercise specialist).<br>Patient.<br>One-to-one.<br>Telephone.

Lenferink, 2019, The Netherlands and Australia	COPD and one or more of: Ishemic heart disease Chronic heart failure Diabetes Anxiety Depression	Physical-Physical	201 102 99	4 weeks intensive and 3 follow up calls at weeks 8, 20 and 36 Baseline, 6 months, 12 months.	COPD exacerbation 12 months No	Self-management	Some concerns	Self-management intervention. Week 1: knowledge on COPD/ comorbidities, symptom recognition/ monitoring, self-treatment action plan (linked to diary), breathing/relaxation exercises, how to check blood glucose. Week 2: record 'usual symptoms', diary training, exacerbation action plan training, mastery of skills (e.g. inhaler use). Week 3: importance of exericse, diet/lifestyle behaviours, re-iteration of diary/action plan use, reiteration of breathing/relaxation. Week 4: re-iteration of diary/action plan use, feedback on diary completion, feedback on actions. Both intervention and usual care groups completed daily symptom diaries for the 12 month period. For the intervention group, self-management behaviours were reinforced, and exacerbation action plans for all co-morbidities were directly linked to their diaries.	Researchers Patient. Weeks 1 and 3: group. Weeks 2 and 4: individual. Phone calls at weeks 8, 20, and 36. Location of face-to-face sessions unspecified.

Markle-Reid, 2018, Canada	Type 2 diabetes<br>Top 3 most common chronic conditions:<br>Hypertension<br>Cardiovascular<br>Arthritis	Physical-Physical	159<br>80<br>79	6 months.<br>Baseline, 6 months.	SF-12 physical and mental<br>6 months<br>Mental - yes<br>Physical - no	Self-management	Low	Self-management programme.<br>The intervention focused on self-efficacy, self-management, holistic care, and individual and caregiver engagement. It was client-driven therefore fleixble to client needs; the indvidual was fully engaged in developing and tailoring their own care plan. The programme offered up to 3 in-home visits from nurse/dietitian/both, monthly group wellness sessions, monthly case conferences amongst the team, and ongoing nurse-led care coordination.	Nurses and dietitians, a program coordinator from a community partner, and peer volunteers.<br>Patient and carer (optional)<br>One-on-one sessions at patients home, group sessions at diabetes education centre.

McDermott, 2015, Australia	Type 2 diabetes One or more other significant comorbidity	Physical-Physical	213 100 113	18 months. Baseline, 18 months.	Clinical (HbA1c) 18 months yes	Self-management	Low	"Getting better at chronic care". Health-worker led case management intervention (one recruited at each site to work as part of primary care team and given caseload of 9 to 26 clients). The roles of the health workers included helping patients make and keep appointments, understand their medications and nutrition and the effects of smoking and work with the family to help support the patient in self-management. Home visits and out-of-clinic care were conducted according to patient preference. Specific training and practice in: chronic care model and evidence-based management and treatment goals for chronic conditions; "hands-on” case management (regular client home visits, including basic diabetes care); working in a primary care team with clear roles/ responsibilities; engaging with families and using local resources to support effective client self-management.	Indigenous healthcare worker (IHW) from community. Health worker/client. One-to-one. Home visits OR out of clinc care (based on preference).

Merlin, 2018, USA	HIV<br>Chronic pain	Physical-Physical	44<br>22<br>22	12 weeks.<br>Baseline, 12 weeks	Pain (BPI)<br>12 weeks<br>no	Self-management	Some concerns	Skills TO Manage Pain (STOMP) plus usual care.<br>Incorporated self-regulation, self-efficacy, observational learning, and outcome expectations.<br>Individual sessions: to build pain self-management skills. All participants received a pain education session, and then selected 5 of the remaining 9 sessions (physical activity and your pain, losing weight to improve your pain, relaxation skills to prevent your pain, sleeping better to help your pain, thinking diferently about your pain, building self-worth, talking with our family and friends about pain, taking opioid pain medications).<br>Group sessions: to enhance peer support related to chronic pain. Each session included sharing refections on lessons learned and goals set during one-on-one sessions, and challenges encountered.	Two peers with HIV and chronic pain ("pain pals") and two research staff ("pain coaches"), working in pal-coach pairs.<br>Participant.<br>Six (in-person) one-to-one sessions (led by pain coach) and six group sessions (led by pal-coach pair), alternating weekly.

Miklavcic, 2020, Canada	Type 2 diabetes<br>Two or more other chronic conditions	Physical-Physical	132<br>70<br>62	6 months.<br>Baseline, 6 months.	Physical functioning<br>6 months<br>No	Collaborative care	High	Aging, Community and Health Research Unit Community Partnership Program.<br>Focused on the role of self-efficacy in achieving self management behaviours, and tailored to patient preferences and needs.The program included up to 3 home visits by RN or RD; 6 monthly group sessions which included education, exercise, a light meal, and peer support; monthly intervention team case conferences (share observations about participants strengths, challenges and goals, identify needs related to other community-based service, and prepare for upcoming group sessions); care coordination to link the client to relevant health or social services.	Interprofessional team consisting of a Registered Nurse (RN), Registered Dietitian (RD), and Program Coordinator (PC).<br>Patient (family caregiver was also invited to attend).<br>One-to-one sessions at patients home, group sessions at community site once a month.

Morgan, 2013, Australia	Depression Type 2 diabetes/ coronary heart disease (or both)	Physical-Mental	400 206 194	6 months. Baseline, 6 months, 12 months	Depression (PHQ-9) 6 months Yes	Cognitive and/or Behavioural Activation	Some concerns	TrueBlue Collaborative care approach. Involved visit with PN followed by usual GP, in which stepped care (psychotherapy or pharmacotherapy) was offered if PHQ-9 scores had not improved or dropped below 5. The PN obtained physical measures and reviewed recent pathology results and lifestyle risk factors. They worked with the patient to identify barriers to achieving goals and discuss ways to overcome them. They assisted the patient with self-management by discussing available educational resources and setting personal goals for review at the next visit.	Practice Nurse (PN) Patient One-to-one. Patient visited their practice every 3 months for 45 minutes (then 15 minutes with the GP).

Naik, 2019, USA	Diabetes Depression	Physical-Mental	225 136 89	6 months Baseline, 6 months, 12 months.	Depression (PHQ-9) and clinical (HbA1c) 6 and 12 months No	Self-management	Low	Healthy Outcomes through Patient Empowerment (HOPE). The first 2 sessions focused on building rapport, introducing and clarifying values, collaboratively setting initial goals, identifying potential skill sets to address goals, and empowering patients to advocate for their health through active communication with clinicians. Sessions 3-6 focused on discrete skill modules (increasing pleasant activities, using thoughts to improve wellness, diet, physical activity, medication management, and relaxation) customized to meet their goals. Sessions 7-9 focused on maintenance skills (reviewing action plans and overcoming barriers).	Trained health professional. Participant. One-to-one. 9 telephone sessions: biweekly (30-40 minutes) in months 1-3, and monthly (15 minutes) from months 4-6). No contact in following 6 months.

Ose, 2019, USA	Type 2 diabetes<br>Two or more other chronic conditions including:<br>Artherosclerosis<br>Coronary heart disease<br>Chronic obstructive lung disease<br>Asthma<br>Cerebrovascular diseases<br>Depression<br>Heart failure<br>Parkinsons<br>Chronic pain	Physical-Physical	495<br>252<br>243	9 months<br>Baseline, 9 months.	Self-care (Summary of Diabetes Self-Care Activities Measure (SDSCA-G)<br>9 months<br>No	Self-management	Low	Care management intervention.<br>3 steps: (1) Structured, IT-supported assessment performed by net-care managers to capture medical/non-medical needs; (2) goal setting between patient and primary care physician based on results of assessment; (3) telephone monitoring by net-care managers to detect acute clinical needs and provide support.<br>Embedded in PCN (net-care manager links to responsible physician via a medical assistant).	Net Care Manager.<br>Patient.<br>One-to-one.<br>(1) at-home visits/face-to-face meetings 3 times within initial 2 weeks, then again at 6 months; (2) once at week 2; (3) every 2 weeks for the first 6 months then every month for the next 3 months.

O'Toole, 2021, Ireland	Conditions not listed. Mean 4.5 per participant	Physical-Physical	149<br>78<br>71	6 weeks. Baseline, 6 weeks, 6 months.	Quality of life (EQ-5D-3L) and behaviour change (participation)<br>6 weeks<br>No	Self-management	Some concerns - a lot of analyses were completed	Occupational therapy-led self-management support programme (OPTIMAL).<br>Intervention incorporated performance accomplishments, vicarious learning, social/verbal persuasion, reinterpretation of physiological and emotional states. Patients' programme goals were set in week 1 and reviewed weekly. They were also given educational and health-promoting resources/materials. Week 1: Introduction to self-management, activity and health, and goal-setting. Week 2: Fatigue management and healthy eating. Week 3: Maintaining physical activity. Week 4: Maintaining mental wellbeing. Week 5: Managing medications. Week 6: Communication and programme review.	Occupational therapist.<br>Patient.<br>Group.<br>2.5 hour face-to-face session once a week for 6 weeks, in a primary care centre or community resource centre.

Park, 2014, South Korea	Two or more of: Stroke Parkinsons Dementia	Physical-Physical	43<br>21<br>22	8 weeks. Baseline, 8 weeks	self-management behaviors, self-efficacy, and health status<br>8 weeks<br>Yes	Cognitive and/or Behavioural Activation	Low	Health coaching self management programme (HCSMP).<br>Group level approach: structured health education developed for older adults, regarding participants' diseases and self-management strategies, then a period of physical activity to enhance cognition and body movements.<br>Individual level approach: individual counselling and goal setting for self-management behaviours.<br>Facility-level approach: principal investigator met frequently with the director and chief manager of the nursing home to help support and facilitate patient progress.	Trained research team members who were geriatric nurse speciaists.<br>Participant.<br>Group sessions delivered face to face at nursing home for 8 weeks; education (Monday) and exercise (Thursday), both lasting 1 hour.<br>Individual sessions: 8 face-to-face coaching sessions for approx 20 minutes,

Pibernik-Okanovic, 2009, Croatia	Diabetes<br>Depression	Physical-Mental	50<br>25<br>25	5 weeks<br>Baseline, 6 months, 12 months	Depression (CES-D) and clinical (HbA1c)<br>6 and 12 months<br>No	Self-management	Low	Psycho-education.<br>Four interactive meetings on the following topics: (1) symptoms of depression, interaction of depression and diabetes; (2) alleviaing burden of depression through activities and problem solving; (3) associations between depression and cognitive processes – thoughts, beliefs and attitudes that induce and maintain depression; (4) developing a personal plan for managing depression-related problems in the future. Patients were provided with a self-help manual and kept daily mood and activities diaries.	Not specified.<br>Patient.<br>Group.<br>Four 90 minute sessions - first two held within the first week and the third and fourth held at two week intervals

Rose, 2018, Canada	COPD and two or more of:<br>Cardiovascular disease<br>Depression<br>Diabetes<br>Osteopenia and osteoporosis<br>Gastro-oesophageal reflux disease<br>Hypothyroidism<br>Osteoarthritis<br>Glaucoma and cataracts<br>Cachexia and malnutrition<br>Chrornic kidney disease<br>Anxiety<br>Peripheral muscle dysfunction<br>Obstructive sleep apnoea<br>Lung cancer<br>Cerebrovascular accident	Physical-Physical	470<br>236<br>234	12 months.<br>Baseline, 3 months, 6 months, and 12 months.	Hospital admissions<br>12 months<br>No	Cognitive and/or Behavioural Activation	Low	Multi-component, case manager led intervention.<br>Components included: (1) standardised 40 minute COPD education session on enrolment; (2) individualised care and action plans for COPD exacerbation recognition, self-management and management of comorbidites; (3) telephone consultations containing standardised reinforcement/ motivational interviewing focusing on health behaviours, action plan teach-back sessions, symptom monitoring, and problem solving strategies; (4) ongoing case manager communication with family physicians and with hospital specialists including respirologists; and 5) priority access to ambulatory outpatient clinics.	Case manager.<br>Patient.<br>One-to-one.<br>12 weekly telephone calls (after initial education session at ED/hospital/outpatient clinic), followed by monthly calls for 9 months.

Ross, 2005, Canada	Asthma<br>Panic disorder	Physical-Mental	48 (25 analysed)<br>25 (15 analsyed)<br>23 (10 analysed)	8 weeks.<br>Intervention group: Baseline, 8 weeks, and 6 month follow-up.<br>Wait-list control group: baseline (coincided with IG pre), 8 weeks (coincided with IG post), 16 weeks, and 6 month follow-up	Panic disorder and asthma outcomes<br>8 weeks<br>Yes	Cognitive and/or Behavioural Activation	Some Concerns - multiple scales used to measure the same outcome	CBT-AE.<br>CBT: (a) education about anxiety and panic; (b) cognitive therapy techniques aimed regarding anxiety and panic; (c) training in slow diaphragmatic breathing to aid physical symptoms of panic attacks; and (d) interoceptive exposure exercises designed to reduce participants' fear of anxiety/panic attack symptons.<br>AE: (a) information about airway inflammation and bronchospasm; (b) rationale, correct use, and side effects of medications, (c) inhaler techniques; (d) methods of self-monitoring, symptoms, and peak flow; (e) asthma triggers and strategies for control; and (f) action plans. Asthma diaries kept by the participants were reviewed during each treatment session to reinforce the importance of the participant's role in self-management and self monitoring.	Two nurse clinicians (one Asthma Educator, one with postdoctoral training in cognitive therapy).<br>Participant.<br>Small groups.<br>12 face-to-face 90 minute sessions with both CBT and AE components. Sessions 1-8 conducted twice weekly, and sessions 9-12 spaced one week apart.
Sajatovic, 2017, USA	Serious mental illness<br>Type 2 diabetes	Physical-Mental	200<br>100<br>100	60 weeks.<br>Baseline, 13 weeks, 30 weeks, 60 weeks	Psychiatric symptoms<br>60 weeks<br>yes	Self-management	Some Concerns	Targeted Training in Illness Management.<br>Group-based psychosocial treatment that blends psychoeducation, problem-identification, goal-setting, behavioral modeling, and care linkage.	Nurse and peer educators.<br>Participant.<br>Group/one-to-one.<br>12 weekly face-to-face sessions, followed by 15 minute one-to-one telephone calls every 2-4 weeks.

Salisbury, 2018, UK	Cardiovascular disease or chronic kidney disease<br>Stroke or transient ischaemic attack<br>Diabetes<br>COPD or asthma<br>Epilepsy<br>Atrial fibrillation<br>Serious mental illness<br>Depression<br>Dementia<br>Learning disability<br>Rheumatoid arthritis	Physical-Physical	33 practices randomised (17 to intervention, 16 to control).<br><br>1546<br>797<br>749	6 months. Baseline, 9 months, 15 months	Quality of life (EQ-5D-3L)<br>15 months<br>No	Collaborative care	Some concerns - self-completed measure for primary outcome and all were aware of group allocation	3D intervention<br>Replace separate disease focused review with one 6-monthly comprehensive multidisciplinary review. Done in 3 stages: (1) nurse review - identifies important health problems and how these affect life, and disease-specific care that the patient needs; (2) pharmacist reviews medication and makes recommendations for treatment; and (3) physician review - considers nurse and pharmacist reviews, then agrees on collaborative health plan with patient (which is printed off) specifying goals and how these will be achieved over the next 6 months of consultations.	Nurse, physician and pharmacist.<br>Patient.<br>One-to-one.<br>Two 6-monthly reviews consisting of two appointments (nurse and physician) and a medication review, then consultations over 6 months as per the health plan.

Schneider, 2015, USA	Type 2 diabetes<br>Depression	Physical-Mental	29<br>15<br>14	6 months.<br>Baseline, 3 months, 6 months.	Clinical outcomes (HbA1c) and depression (BDI-II)<br>3 and 6 months<br>No	Cognitive and/or Behavioural Activation	Low	Behavioural Activation and Exercise Condition.<br>Classes contained 3 parts - (i) warm-up, (ii) exercise (different types for first 12 weeks then based on paricipant enjoyment for next 12, gradually increasing in intensity each week), and (iii) cool down and BA (activity and mood monitoring, exploration of ways to make exercise more enjoyable, a behavioural contract with friends/ family to promote exercise, a list of life values and how to connect exercise to them, and exercise counseling strategies including self-monitoring activity using a pedometer, monitoring exercise intensity via perceived exertion and heart rate, identifying facilitators to exercise and engaging in problem solving for exercise barriers).	Female group leader.<br>Participant.<br>Group.<br>One orientation session and 38 classes over 4 weeks at a medical school site. The 90-minute classes occurred 2 times per week for 16 weeks, once a week for 4 weeks, and then once every other week for 4 weeks.

Takahashi, 2012, USA	Myocardial infarction<br>Congestive heart failure<br>COPD<br>Diabetes<br>Renal disease	Physical-Physical	205<br>102<br>103	12 months<br>Baseline, 12 months	Hospital admissions<br>12 months<br>No	Self-management	Some concerns - outcome assessors were not blind at follow-up, more deaths in intervention group	Telemonitoring.<br>An FDA approved telemonitoring device was used in the patients home. Patients performed daily assessment of symptoms and biometrics, and data were downloaded and reviewed daily by the health care team. A registered nurse communicated with the individuals via phone or videoconference if alerts arose, communicating with their primary physician about treatment options if needed.	Registered nurse and (if required) primary physician oversaw the process.<br>Patient.<br>One-to-one.<br>Daily 5-10 minute sessions<br>Home or clinical

Vera, 2010, USA	Depression<br>And one or more of:<br>Diabetes<br>Hypothryoidism<br>Asthma<br>Hypertension<br>Chronic bronchitis<br>Arthritis<br>Heart disease<br>High cholesterol<br>Stroke	Physical-Mental	179<br>89<br>90	6 months.<br>Baseline, 8, 16 and 24 weeks.	Depression (SCL-20) and Functioning (SF-36)<br>6 months<br>Yes	Collaborative care	Low	Collaborative care.<br>A multicomponent intervention, including program oversight and teamwork among physicians, mental health specialists, and care managers. Patients were provided education about depression and a choice of 13 CBT sessions or antidepressant medication as initial treatment. The care manager participated in the coordination of treatment initiation, as well as monitoring treatment adherence, side effects, and clinical response, while liaising with the psychiatrist and patients physician. Extra contacts were available to help patients overcome barriers and provide treatment adherence support.	Care manager.<br>Patient<br>One-to-one.<br>Patients were contacted by telephone or in person every 2 weeks initially, and then monthly up to 6 months.

Wakefield, 2011, USA	Type 2 diabetes<br>Hypertension	Physical-Physical	302<br>102 (low intensity) / 93 (high intensity)<br>107	6 months.<br>Baseline, 6 months, 12 months	Clinical (HbA1c and SBP)<br>6 month<br>Yes	Self-management	Low	Telehealth intervention.<br>In a home telehealth device, all intervention participants logged blood pressure, blood glucose and responses to standardised questions (which were reinforced when answered correctly and reviewed/explained when answered incorrectly), which were reviewed daily by a registered nurse to determine whether the participant needed a follow-up (e.g. if clinical parameters were out of range or the participant needed extra support). In the high intensity group, a disease management algorithm was used, with scheduled prompts for rotating questions and education focused on diet, exercise, smoking cessation, foot care, advice for sick days, medications, weight management, preventive care, and behavior modification and lifestyle adjustments. In the low intensity group, participants responded to a smaller subset of questions from the HI group and did not use the algorithm.	Nurse.<br>Participant.<br>One-to-one.<br>Telehealth device was in patient home - follow-ups were made via telephone, letter or directly through the device as and when needed.

Williams, 2012, Australia	Diabetes<br>Chronic kidney disease/diabetic kidney disease<br>Hypertension	Physical-Physical	80<br>39<br>41	3 months.<br>Baseline, 3 months, 6 months, 9 months.	Medication adherence<br>3-6-9 months<br>No	Self-management	Low	Medication Self-Management Intervention (MESMI).<br>The intervention consisted of self-monitoring of blood pressure (participants were taught how to do this and recorded it daily for 3 months), an individualized medication review, a 20-minute DVD (using an interactive, psychosocial approach to motivate people to take their medications), and motivational interviewing to support blood pressure control and optimal medication self-management.	Intervention nurse.<br>Participant.<br>One-to-one.<br>Motivational interviewing was done fortnightly via telephone calls, starting 2 weeks after the intervention home visit until 3 months.
Wilson, 2018, USA	Depression<br>Any other chronic disease (most common: chronic pain, multiple sclerosis, cardiovascular disease, mental disorder)	Physical-Mental	53<br>27<br>26	8 weeks.<br>Baseline, 4 weeks, 8 weeks.	Depression (PHQ-8)<br>8 weeks<br>Yes	Cognitive and/or Behavioural Activation	Some concerns - as unblinded participants completing assessments, including primary outcome	Think Clearly About Depression.<br>Computer based intervention to teach mildly/moderately depressed individuals about the role of thinking in the development and maintenance of depression, using videos, interactive activities and homework exercises to challenge depressive thoughts and practice hopeful and constructive thinking, as well as a daily mood tracker and a schedue for mood boosting self-management activities. To increase engagement, the researchers created a program guide to assist participants and sent weekly prompts for the first 4 weeks.	Research team (two psychiatric nurse practicioners and a psychiatrist).<br>Participant.<br>One-to-one.<br>The program could be accessed at any time of day in any setting of the participants' choosing where they had Internet services and a personal computer or smartphone.

Yu, 2020, Canada	Diabetes AND two of:<br>Heart disease<br>Stroke<br>Hypertension<br>Cancer<br>Chronic lung disease<br>Arthritis<br>Inflammatory bowel disorder<br>Urinary incontinence	Physical-Physical	10 primary care practices were randomsed (5 in each arm).<br>213<br>102<br>111	6 months<br>Baseline, 6 months, 12 months.	Behaviour change (Decisional conflict)<br>12 months<br>No	Self-management	Some concerns - as unblinded participants completing assessments, including primary outcome	MyDiabetesPlan.<br>Web-based intervention where participants provide cardiometabolic and psychosocial profiles and general care priorities. An individualised action plan is then generated with diabetes-specific goals and strategies. For the first 6 months, MyDiabetesPlan was completed by a member of the clinical team with the patient, then their action plan was reviewed. At the 6-month mark patients were given a "how to" guide for MyDiabetesPlan and from then on logged the information themselves before their appointment.	Clinician (nurse, dietitian, physician, or pharmacist).<br>Patient.<br>One-to-one.<br>Clinical encounters were done face-to-face at the participants primary care clinic.
