# Supplementary material for: Effectiveness of complex behaviour change interventions tested in randomised controlled trials for people with multiple long-term conditions (M-LTCs): systematic review with meta-analysis
Source: BMJ Open. 2024 Jun 16;14(6):e081104. doi: 10.1136/bmjopen-2023-081104 (PMC11184186; doi:10.1136/bmjopen-2023-081104)

Supplementary figure 3

Table 2 significant effects forest plots

Psychological distress (depression) – post-intervention overall effect

	Intervention			Control				Std. Mean Difference
Study or Subgroup	Mean	SD	Total	Mean	SD	Total	Weight	IV, Random, 95% CI
Ali 2020	0.52	0.38	192	0.82	0.54	202	5.2%	-0.64 [-0.84, -0.44]
Aragones 2019	1.29	0.77	167	1.42	0.73	161	5.2%	-0.17 [-0.39, 0.04]
Barley 2014	10.3	3.8	35	9.2	4.6	39	4.7%	0.26 [-0.20, 0.71]
Boeschoten 2017	20.3	8.8	74	21	9.1	78	5.0%	-0.08 [-0.40, 0.24]
Bogner 2008	9.9	10.7	32	19.3	15.2	32	4.5%	-0.71 [-1.21, -0.20]
Cummings 2019	6.3	5.9	67	7.9	7	72	5.0%	-0.25 [-0.58, 0.09]
Ell 2017	1.02	0.07	178	1.15	0.07	170	5.1%	-1.85 [-2.10, -1.60]
Garvey 2015	6.32	4.19	22	7.82	3.83	22	4.3%	-0.37 [-0.96, 0.23]
House 2018	2.4	1.87	30	1.1	1.8	29	4.5%	0.70 [0.17, 1.23]
Katon 2010	0.83	0.68	105	1.14	0.66	106	5.1%	-0.46 [-0.73, -0.19]
Kroenke 2009	1.14	0.69	123	1.69	0.74	127	5.1%	-0.77 [-1.02, -0.51]
Markle-Reid 2018	4.67	4.83	71	5.71	5.24	65	5.0%	-0.21 [-0.54, 0.13]
Miklavcic 2020	4.94	4.46	63	5.82	4.93	50	4.9%	-0.19 [-0.56, 0.19]
Morgan 2013	7.1	0.8	164	9	0.9	146	5.1%	-2.23 [-2.52, -1.95]
Naik 2019	10.9	6.1	107	12.4	6	80	5.0%	-0.25 [-0.54, 0.04]
Park 2014	5.3	3.4	22	7.6	5.8	21	4.3%	-0.48 [-1.09, 0.13]
Ross 2005	11.8	9.93	15	16.78	7.89	9	3.6%	-0.52 [-1.36, 0.32]
Sajatovic 2017	15.92	10	100	18.55	8.8	100	5.1%	-0.28 [-0.56, 0.00]
Salisbury 2018	6.1	4.4	654	6.6	4.5	641	5.3%	-0.11 [-0.22, -0.00]
Schneider 2016	12.5	14.8	15	12.3	4.1	14	3.9%	0.02 [-0.71, 0.75]
Wilson 2018	17.46	6.16	22	20.5	5.3	25	4.3%	-0.52 [-1.11, 0.06]
**Total (95% CI)**			**2258**			**2189**	**100.0%**	**-0.45 [-0.73, -0.16]**

Heterogeneity: $Tau^2 = 0.38$; $Chi^2 = 366.51$, df = 20 ($P < 0.00001$); $I^2 = 95\%$
Test for overall effect: Z = 3.11 (P = 0.002)

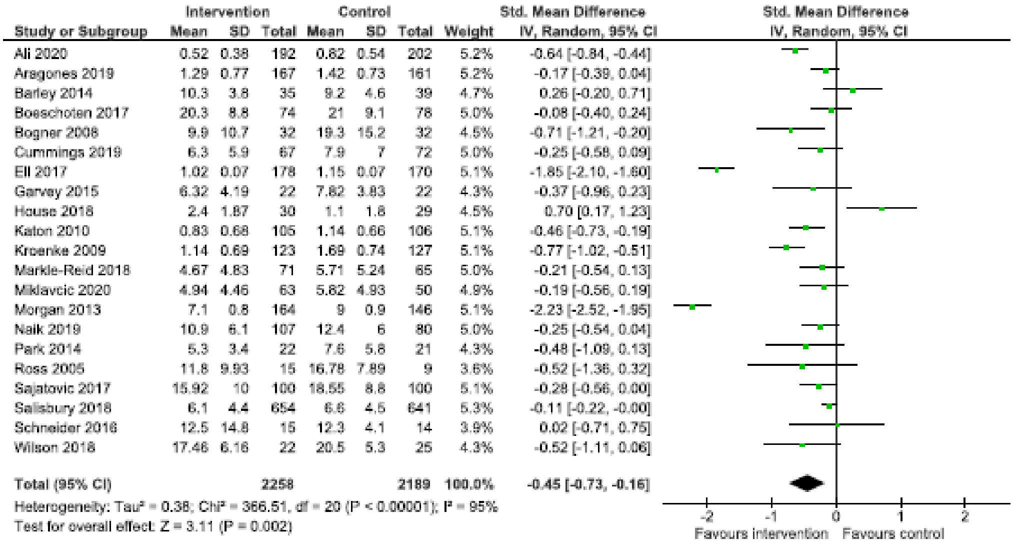

Psychological distress (anxiety) – post-intervention overall effect

	Intervention			Control				Std. Mean Difference
Study or Subgroup	Mean	SD	Total	Mean	SD	Total	Weight	IV, Random, 95% CI
Barley 2014	11	5	35	10.4	5	39	7.5%	0.12 [-0.34, 0.58]
Battersby 2013	48.95	10.39	44	47	13.75	28	7.1%	0.16 [-0.31, 0.64]
Boeschoten 2017	9	3.8	74	9.3	3.8	78	12.4%	-0.08 [-0.40, 0.24]
Garvey 2015	9.5	4.71	22	9.09	4.96	22	4.9%	0.08 [-0.51, 0.67]
Kroenke 2009	5.8	5	123	8	5.1	127	16.2%	-0.43 [-0.69, -0.18]
Markle-Reid 2018	2.34	2.92	77	2.71	3.27	66	11.9%	-0.12 [-0.45, 0.21]
Miklavcic 2020	2.37	3.32	63	3.24	3.68	50	10.1%	-0.25 [-0.62, 0.12]
Ross 2005	31.73	22.29	15	57.56	31.56	9	2.4%	-0.96 [-1.84, -0.08]
Salisbury 2018	5.7	4.6	652	6.1	4.7	638	27.4%	-0.09 [-0.20, 0.02]
**Total (95% CI)**			**1105**			**1057**	**100.0%**	**-0.14 [-0.28, 0.00]**

Heterogeneity: $Tau^2 = 0.02$; $Chi^2 = 13.27$, df = 8 ($P = 0.10$); $I^2 = 40\%$
Test for overall effect: Z = 1.95 (P = 0.05)

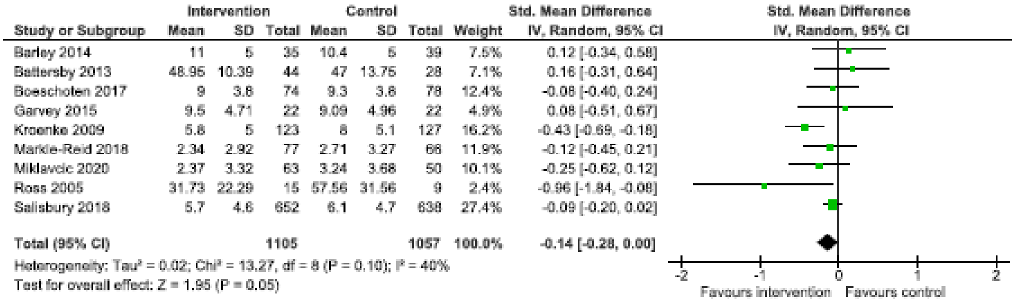

Emotional wellbeing – post-intervention overall effect

	Intervention			Control				Std. Mean Difference
Study or Subgroup	Mean	SD	Total	Mean	SD	Total	Weight	IV, Random, 95% CI
Barley 2014	32.4	11.2	35	35.4	11.8	39	11.9%	-0.26 [-0.72, 0.20]
Druss 2018	34.49	11.15	198	34.25	11.97	202	16.4%	0.02 [-0.18, 0.22]
Ell 2010	47.31	11.48	193	43.6	12.46	194	16.3%	0.31 [0.11, 0.51]
Ell 2017	44.54	1.03	178	43.67	1.02	170	16.0%	0.85 [0.63, 1.07]
Goldberg 2013	50.5	11.1	28	43.2	10.7	30	10.7%	0.66 [0.13, 1.19]
Miklavcic 2020	56.69	8.37	65	53.88	7.67	56	13.6%	0.35 [-0.01, 0.71]
Sajatovic 2017	42.05	11.1	100	39.58	11.4	100	15.1%	0.22 [-0.06, 0.50]
**Total (95% CI)**			**797**			**791**	**100.0%**	**0.31 [0.04, 0.58]**

Heterogeneity: $Tau^2 = 0.11$; $Chi^2 = 39.32$, df = 6 ($P < 0.00001$); $I^2 = 85\%$
Test for overall effect: Z = 2.23 (P = 0.03)

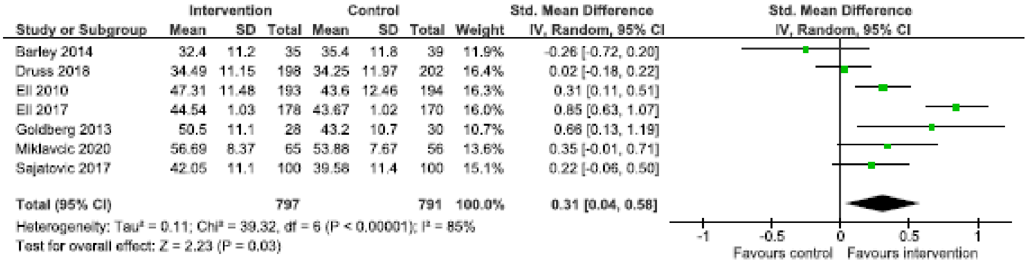

Emotional wellbeing – maintenance overall effect

	Intervention			Control				Std. Mean Difference
Study or Subgroup	Mean	SD	Total	Mean	SD	Total	Weight	IV, Random, 95% CI
Barley 2014	34.5	11.6	32	33.6	12.5	37	10.5%	0.07 [-0.40, 0.55]
Druss 2018	36.64	12.28	198	34.54	11.82	202	18.7%	0.17 [-0.02, 0.37]
Ell 2010	45.1	12.19	193	43.49	11.66	194	18.6%	0.13 [-0.06, 0.33]
Ell 2017	31.24	0.87	178	30.59	0.89	170	18.0%	0.74 [0.52, 0.95]
Goldberg 2013	46.9	10.7	28	43.9	11.5	29	9.4%	0.27 [-0.26, 0.79]
Griva 2019	2.38	0.72	20	2.15	0.63	24	7.9%	0.34 [-0.26, 0.93]
Kanwal 2018	41.8	13.8	114	39.4	11.9	128	16.9%	0.19 [-0.07, 0.44]
**Total (95% CI)**			**763**			**784**	**100.0%**	**0.28 [0.07, 0.49]**

Heterogeneity: Tau² = 0.05; Chi² = 21.42, df = 6 (P = 0.002); I² = 72%
Test for overall effect: Z = 2.63 (P = 0.009)

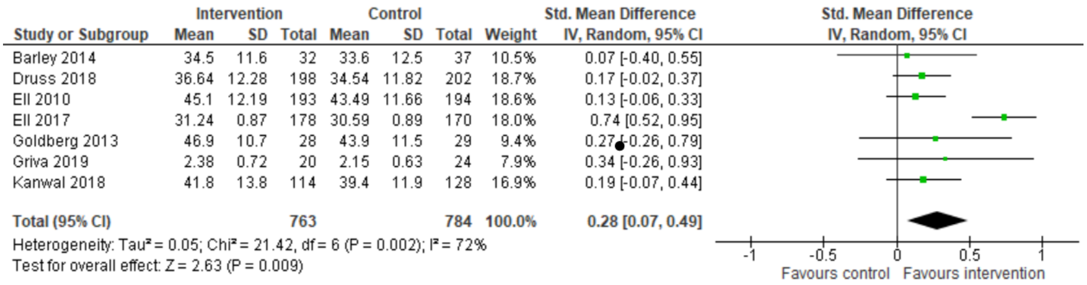

Table 3 significant effects forest plots

Quality of life – post-intervention by intervention type

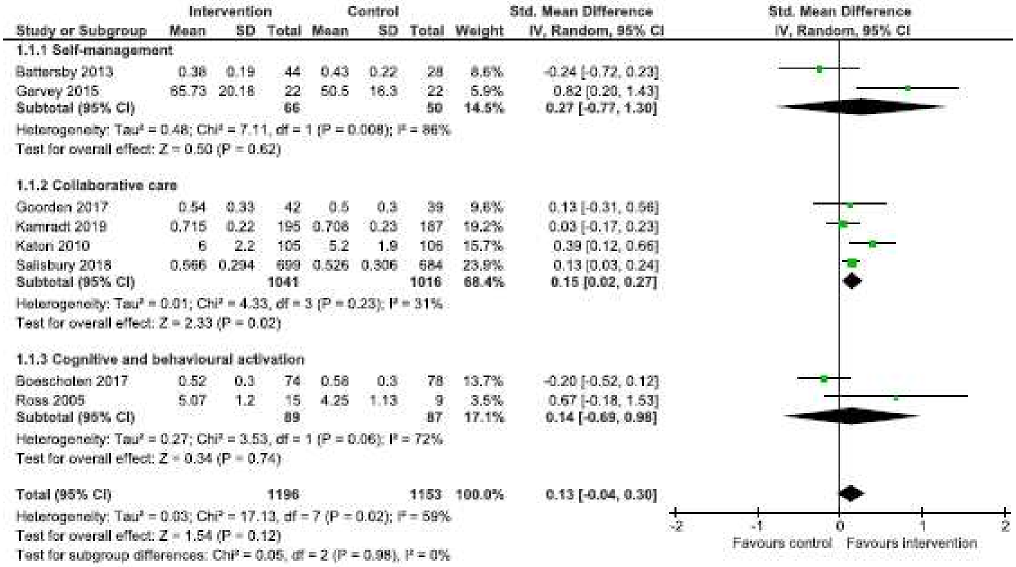

Psychological distress (depression) – post-intervention by intervention type

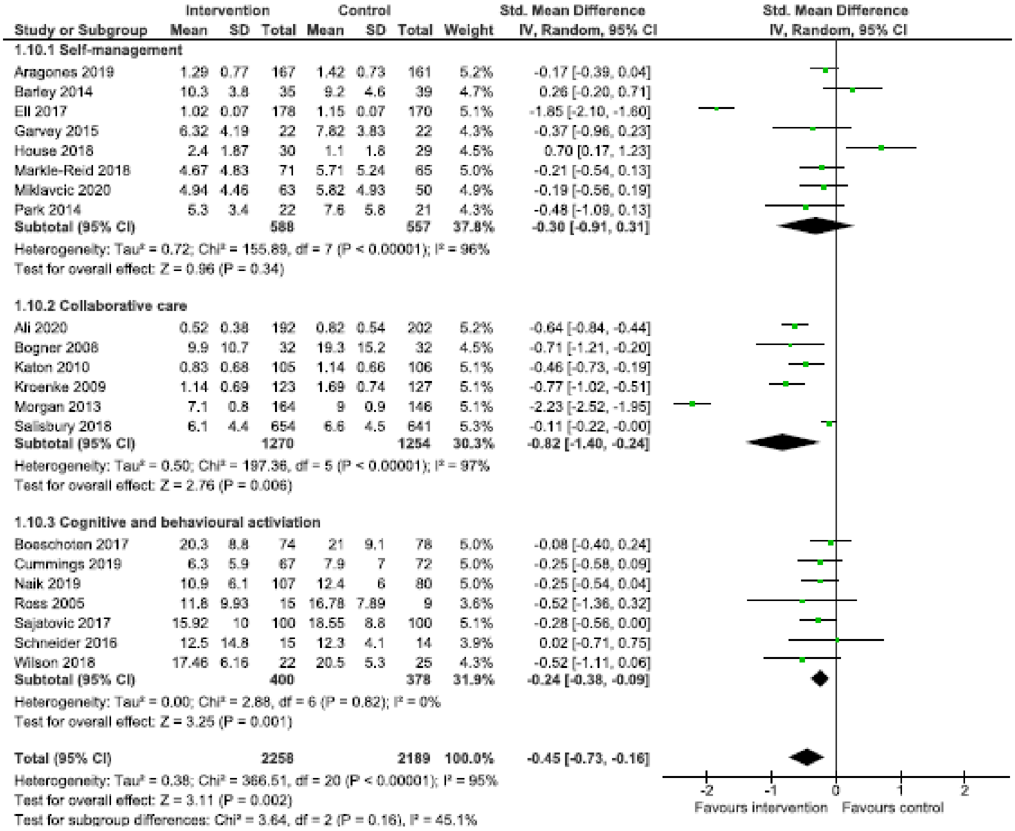

Emotional wellbeing – post-intervention by intervention type

Study or Subgroup	Intervention Mean	Intervention SD	Intervention Total	Control Mean	Control SD	Control Total	Weight	Std. Mean Difference IV, Random, 95% CI
**1.8.1 Self-management**								
Barley 2014	32.4	11.2	35	35.4	11.8	39	11.9%	-0.26 [-0.72, 0.20]
Druss 2018	34.49	11.15	198	34.25	11.97	202	16.4%	0.02 [-0.18, 0.22]
Ell 2017	44.54	1.03	178	43.67	1.02	170	16.0%	0.85 [0.63, 1.07]
Goldberg 2013	50.5	11.1	28	43.2	10.7	30	10.7%	0.66 [0.13, 1.19]
Miklavcic 2020	56.69	8.37	65	53.88	7.67	56	13.6%	0.35 [-0.01, 0.71]
**Subtotal (95% CI)**			**504**			**497**	**68.6%**	**0.33 [-0.10, 0.76]**
Heterogeneity: Tau² = 0.21; Chi² = 38.60, df = 4 (P < 0.00001); I² = 90%								
Test for overall effect: Z = 1.49 (P = 0.14)								
**1.8.2 Collaborative Care**								
Ell 2010	47.31	11.48	193	43.6	12.46	194	16.3%	0.31 [0.11, 0.51]
**Subtotal (95% CI)**			**193**			**194**	**16.3%**	**0.31 [0.11, 0.51]**
Heterogeneity: Not applicable								
Test for overall effect: Z = 3.02 (P = 0.003)								
**1.8.3 Cognitive and behavioural activiation**								
Sajatovic 2017	42.05	11.1	100	39.58	11.4	100	15.1%	0.22 [-0.06, 0.50]
**Subtotal (95% CI)**			**100**			**100**	**15.1%**	**0.22 [-0.06, 0.50]**
Heterogeneity: Not applicable								
Test for overall effect: Z = 1.54 (P = 0.12)								
**Total (95% CI)**			**797**			**791**	**100.0%**	**0.31 [0.04, 0.58]**
Heterogeneity: Tau² = 0.11; Chi² = 39.32, df = 6 (P < 0.00001); I² = 85%								
Test for overall effect: Z = 2.23 (P = 0.03)								
Test for subgroup differences: Chi² = 0.31, df = 2 (P = 0.86), I² = 0%								

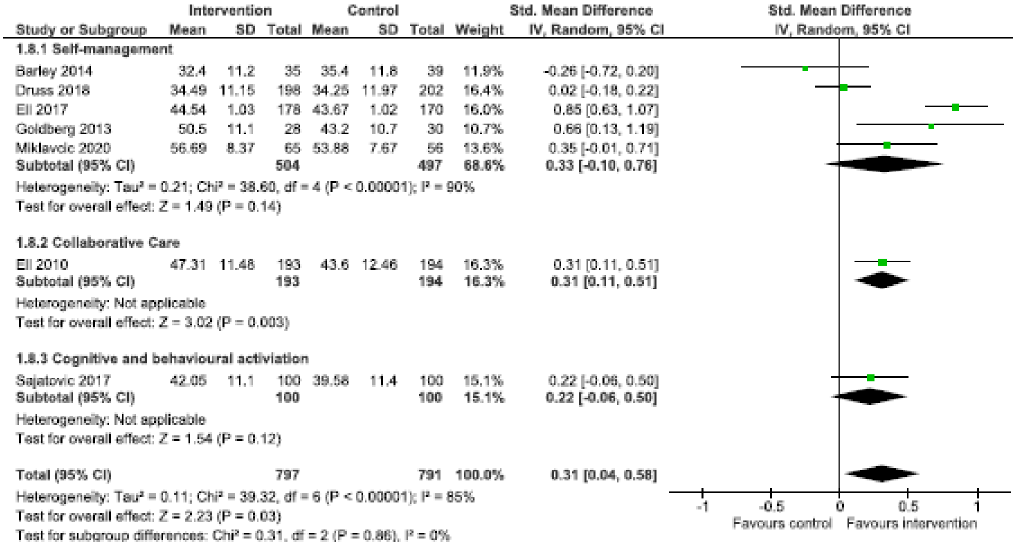

Emotional wellbeing – maintenance by intervention type

Study or Subgroup	Intervention Mean	Intervention SD	Intervention Total	Control Mean	Control SD	Control Total	Weight	Std. Mean Difference IV, Random, 95% CI
**1.9.1 Self-management**								
Barley 2014	34.5	11.6	32	33.6	12.5	37	10.5%	0.07 [-0.40, 0.55]
Druss 2018	36.64	12.28	198	34.54	11.82	202	18.7%	0.17 [-0.02, 0.37]
Ell 2017	31.24	0.87	178	30.59	0.89	170	18.0%	0.74 [0.52, 0.95]
Goldberg 2013	46.9	10.7	28	43.9	11.5	29	9.4%	0.27 [-0.26, 0.79]
Griva 2019	2.38	0.72	20	2.15	0.63	24	7.9%	0.34 [-0.26, 0.93]
**Subtotal (95% CI)**			**456**			**462**	**64.5%**	**0.34 [0.03, 0.65]**
Heterogeneity: Tau² = 0.09; Chi² = 16.42, df = 4 (P = 0.003); I² = 76%								
Test for overall effect: Z = 2.14 (P = 0.03)								
**1.9.2 Collaborative care**								
Ell 2010	45.1	12.19	193	43.49	11.66	194	18.6%	0.13 [-0.06, 0.33]
Kanwal 2018	41.8	13.8	114	39.4	11.9	128	16.9%	0.19 [-0.07, 0.44]
**Subtotal (95% CI)**			**307**			**322**	**35.5%**	**0.15 [-0.00, 0.31]**
Heterogeneity: Tau² = 0.00; Chi² = 0.10, df = 1 (P = 0.75); I² = 0%								
Test for overall effect: Z = 1.93 (P = 0.05)								
**1.9.3 Cognitive and behavioural activiation**								
**Subtotal (95% CI)**			**0**			**0**		**Not estimable**
Heterogeneity: Not applicable								
Test for overall effect: Not applicable								
**Total (95% CI)**			**763**			**784**	**100.0%**	**0.28 [0.07, 0.49]**
Heterogeneity: Tau² = 0.05; Chi² = 21.42, df = 6 (P = 0.002); I² = 72%								
Test for overall effect: Z = 2.63 (P = 0.009)								
Test for subgroup differences: Chi² = 1.09, df = 1 (P = 0.30), I² = 7.9%								

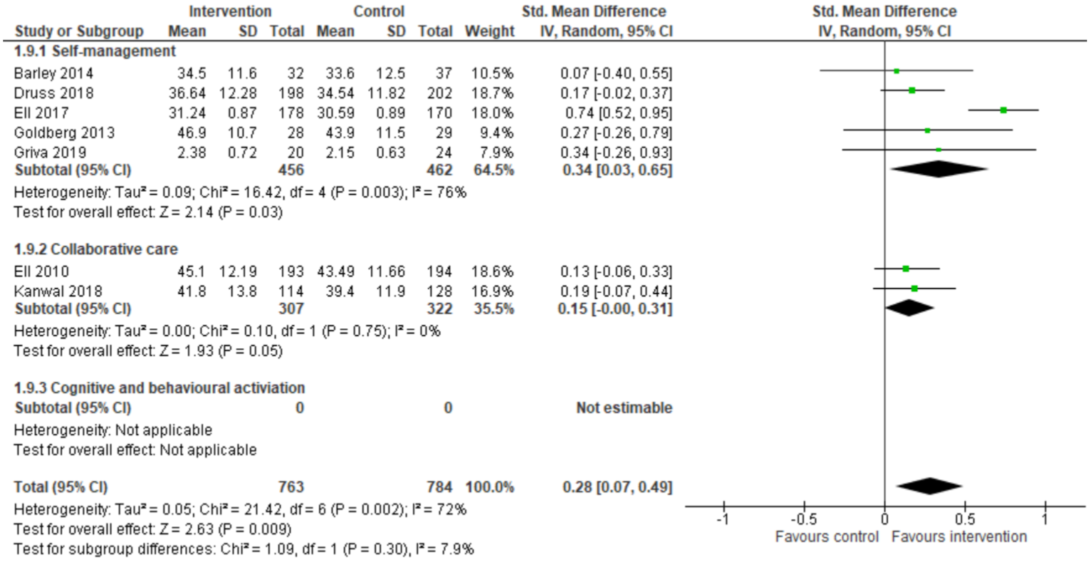

Psychological distress (depression) – maintenance by intervention type

Study or Subgroup	Intervention Mean	Intervention SD	Intervention Total	Control Mean	Control SD	Control Total	Weight	Std. Mean Difference IV, Random, 95% CI
**1.11.1 Self-management**								
Aragones 2019	1.11	0.72	167	1.34	0.76	161	13.3%	-0.31 [-0.53, -0.09]
Barley 2014	9.5	4.6	32	8.8	4.8	37	11.1%	0.15 [-0.33, 0.62]
Ell 2017	1.69	0.06	178	1.63	0.06	170	13.3%	1.00 [0.77, 1.22]
Griva 2019	5.3	4.22	20	4.22	3.88	24	9.9%	0.26 [-0.33, 0.86]
**Subtotal (95% CI)**			**397**			**392**	**47.7%**	**0.28 [-0.48, 1.04]**
Heterogeneity: Tau² = 0.56; Chi² = 68.16, df = 3 (P < 0.00001); I² = 96%								
Test for overall effect: Z = 0.71 (P = 0.47)								
**1.11.2 Collaborative care**								
Ali 2020	0.39	0.27	185	0.53	0.37	193	13.4%	-0.43 [-0.63, -0.23]
Salisbury 2018	6.1	4.6	630	6.8	4.6	625	13.9%	-0.15 [-0.26, -0.04]
**Subtotal (95% CI)**			**815**			**818**	**27.3%**	**-0.28 [-0.55, -0.01]**
Heterogeneity: Tau² = 0.03; Chi² = 5.50, df = 1 (P = 0.02); I² = 82%								
Test for overall effect: Z = 2.01 (P = 0.04)								
**1.11.3 Cognitive and behavioural activiation**								
Boeschoten 2017	20.8	10.4	64	20.1	9.2	67	12.4%	0.07 [-0.27, 0.41]
Naik 2019	10.1	6.9	96	12.6	6.5	70	12.6%	-0.37 [-0.68, -0.06]
**Subtotal (95% CI)**			**160**			**137**	**25.0%**	**-0.16 [-0.59, 0.28]**
Heterogeneity: Tau² = 0.07; Chi² = 3.48, df = 1 (P = 0.06); I² = 71%								
Test for overall effect: Z = 0.71 (P = 0.48)								
**Total (95% CI)**			**1372**			**1347**	**100.0%**	**0.02 [-0.33, 0.36]**
Heterogeneity: Tau² = 0.22; Chi² = 113.01, df = 7 (P < 0.00001); I² = 94%								
Test for overall effect: Z = 0.10 (P = 0.92)								
Test for subgroup differences: Chi² = 1.87, df = 2 (P = 0.39), I² = 0%								

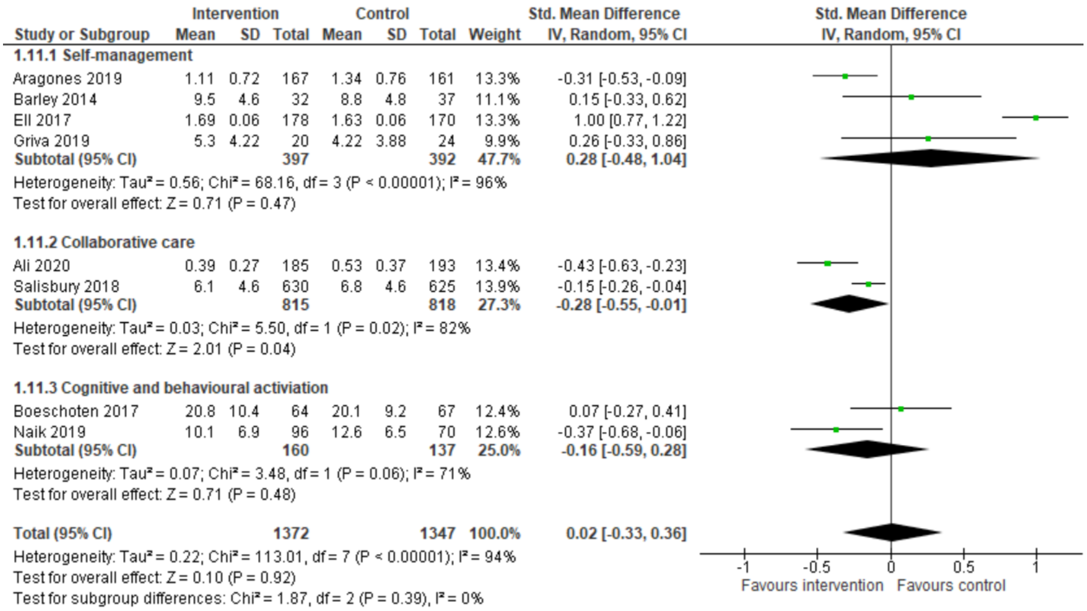

Table 4 significant effects forest plots

Clinical endpoints – post-intervention by intervention length

Study or Subgroup	Intervention Mean	Intervention SD	Intervention Total	Control Mean	Control SD	Control Total	Weight	Std. Mean Difference IV, Random, 95% CI
**1.3.1 <3months**								
**Subtotal (95% CI)**			**0**			**0**		**Not estimable**
Heterogeneity: Not applicable								
Test for overall effect: Not applicable								
**1.3.2 3-6months**								
House 2018	7.1	1.24	37	7.1	1.79	38	8.1%	0.00 [-0.45, 0.45]
Morgan 2013	6.9	0.26	89	7.4	0.36	67	8.9%	-1.62 [-1.99, -1.26]
Naik 2019	9.1	1.7	119	8.7	1.7	82	9.6%	0.23 [-0.05, 0.52]
Schneider 2016	7.8	0.9	15	7.7	0.8	14	5.7%	0.11 [-0.62, 0.84]
**Subtotal (95% CI)**			**260**			**201**	**32.2%**	**-0.33 [-1.30, 0.65]**
Heterogeneity: $Tau^2 = 0.93$; $Chi^2 = 66.74$, $df = 3$ ($P < 0.00001$); $I^2 = 96\%$								
Test for overall effect: $Z = 0.66$ ($P = 0.51$)								
**1.3.3 >6months**								
Ali 2020	8.19	1.8	192	8.44	1.82	202	10.2%	-0.14 [-0.34, 0.06]
Cummings 2019	8.96	2.1	67	9.04	2.2	72	9.1%	-0.04 [-0.37, 0.30]
Ell 2010	8.52	2.01	193	8.59	2.26	194	10.2%	-0.03 [-0.23, 0.17]
Jackson 2021	137.8	17.4	91	148.6	20.1	91	9.5%	-0.57 [-0.87, -0.28]
Katon 2010	131	18.2	105	132.3	17.4	106	9.7%	-0.07 [-0.34, 0.20]
McDermott 2015	9.8	2.3	84	10.3	2.2	105	9.5%	-0.22 [-0.51, 0.07]
Sajatovic 2017	7.69	1.9	100	7.77	2	100	9.6%	-0.04 [-0.32, 0.24]
**Subtotal (95% CI)**			**832**			**870**	**67.8%**	**-0.15 [-0.28, -0.02]**
Heterogeneity: $Tau^2 = 0.01$; $Chi^2 = 10.66$, $df = 6$ ($P = 0.10$); $I^2 = 44\%$								
Test for overall effect: $Z = 2.25$ ($P = 0.02$)								
**Total (95% CI)**			**1092**			**1071**	**100.0%**	**-0.22 [-0.47, 0.03]**
Heterogeneity: $Tau^2 = 0.15$; $Chi^2 = 80.56$, $df = 10$ ($P < 0.00001$); $I^2 = 88\%$								
Test for overall effect: $Z = 1.72$ ($P = 0.09$)								
Test for subgroup differences: $Chi^2 = 0.13$, $df = 1$ ($P = 0.72$), $I^2 = 0\%$								

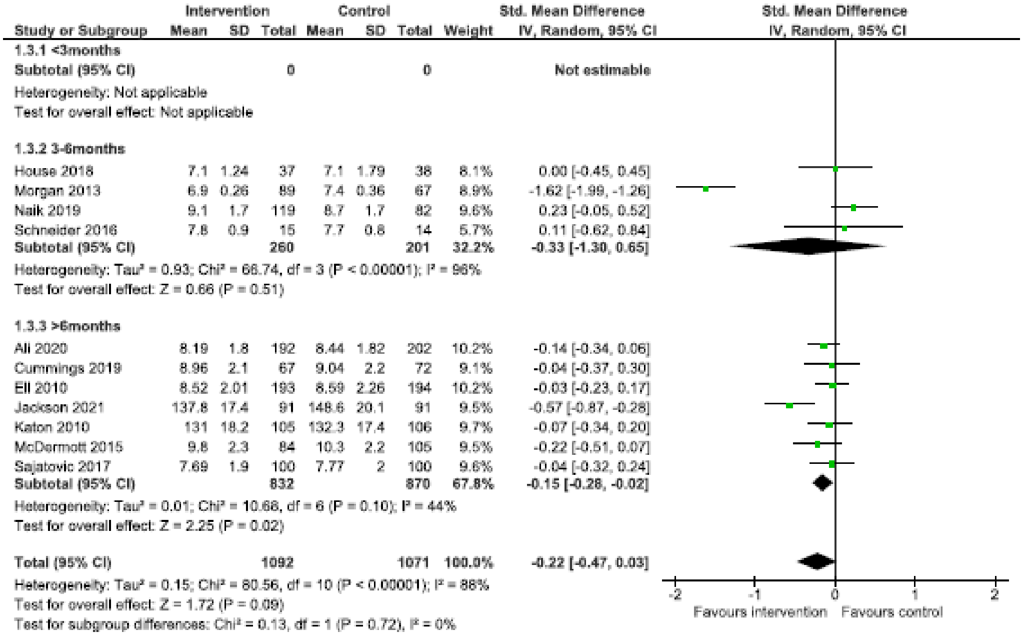

Psychological distress (depression) – post-intervention by intervention length

Study or Subgroup	Intervention Mean	Intervention SD	Intervention Total	Control Mean	Control SD	Control Total	Weight	Std. Mean Difference IV, Random, 95% CI
**1.10.1 <3months**								
Boeschoten 2017	20.3	8.8	74	21	9.1	78	5.0%	-0.08 [-0.40, 0.24]
Bogner 2008	9.9	10.7	32	19.3	15.2	32	4.5%	-0.71 [-1.21, -0.20]
Garvey 2015	6.32	4.19	22	7.82	3.83	22	4.3%	-0.37 [-0.96, 0.23]
Park 2014	5.3	3.4	22	7.6	5.8	21	4.3%	-0.48 [-1.09, 0.13]
Ross 2005	11.8	9.93	15	16.78	7.89	9	3.6%	-0.52 [-1.36, 0.32]
Wilson 2018	17.46	6.16	22	20.5	5.3	25	4.3%	-0.52 [-1.11, 0.06]
**Subtotal (95% CI)**			**187**			**187**	**26.0%**	**-0.36 [-0.57, -0.14]**
Heterogeneity: Tau² = 0.01; Chi² = 5.38, df = 5 (P = 0.37); I² = 7%								
Test for overall effect: Z = 3.21 (P = 0.001)								
**1.10.2 3-6months**								
Aragones 2019	1.29	0.77	167	1.42	0.73	161	5.2%	-0.17 [-0.39, 0.04]
Barley 2014	10.3	3.8	35	9.2	4.6	39	4.7%	0.26 [-0.20, 0.71]
Ell 2017	1.02	0.07	178	1.15	0.07	170	5.1%	-1.85 [-2.10, -1.60]
House 2018	2.4	1.87	30	1.1	1.8	29	4.5%	0.70 [0.17, 1.23]
Markle-Reid 2018	4.67	4.83	71	5.71	5.24	65	5.0%	-0.21 [-0.54, 0.13]
Miklavcic 2020	4.94	4.46	63	5.82	4.93	50	4.9%	-0.19 [-0.56, 0.19]
Morgan 2013	7.1	0.8	164	9	0.9	146	5.1%	-2.23 [-2.52, -1.95]
Naik 2019	10.9	6.1	107	12.4	6	80	5.0%	-0.25 [-0.54, 0.04]
Salisbury 2018	6.1	4.4	654	6.6	4.5	641	5.3%	-0.11 [-0.22, -0.00]
Schneider 2016	12.5	14.8	15	12.3	4.1	14	3.9%	0.02 [-0.71, 0.75]
**Subtotal (95% CI)**			**1484**			**1395**	**48.6%**	**-0.42 [-0.96, 0.12]**
Heterogeneity: Tau² = 0.71; Chi² = 347.55, df = 9 (P < 0.00001); I² = 97%								
Test for overall effect: Z = 1.53 (P = 0.13)								
**1.10.3 >6months**								
Ali 2020	0.52	0.38	192	0.82	0.54	202	5.2%	-0.64 [-0.84, -0.44]
Cummings 2019	6.3	5.9	67	7.9	7	72	5.0%	-0.25 [-0.58, 0.09]
Katon 2010	0.83	0.68	105	1.14	0.66	106	5.1%	-0.46 [-0.73, -0.19]
Kroenke 2009	1.14	0.69	123	1.69	0.74	127	5.1%	-0.77 [-1.02, -0.51]
Sajatovic 2017	15.92	10	100	18.55	8.8	100	5.1%	-0.28 [-0.56, 0.00]
**Subtotal (95% CI)**			**587**			**607**	**25.4%**	**-0.50 [-0.69, -0.30]**
Heterogeneity: Tau² = 0.03; Chi² = 10.51, df = 4 (P = 0.03); I² = 62%								
Test for overall effect: Z = 5.07 (P < 0.00001)								
**Total (95% CI)**			**2258**			**2189**	**100.0%**	**-0.45 [-0.73, -0.16]**
Heterogeneity: Tau² = 0.38; Chi² = 366.51, df = 20 (P < 0.00001); I² = 95%								
Test for overall effect: Z = 3.11 (P = 0.002)								
Test for subgroup differences: Chi² = 0.88, df = 2 (P = 0.64), I² = 0%								

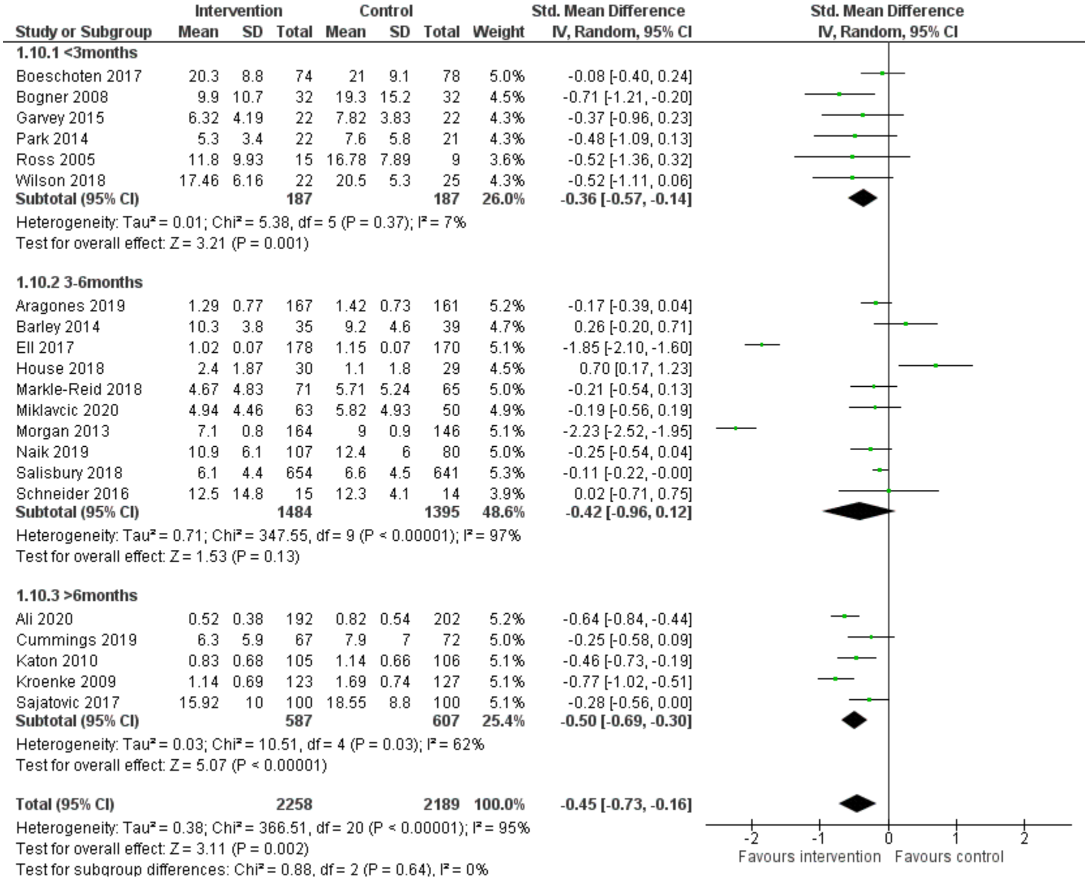

Emotional wellbeing – post-intervention by intervention length

Study or Subgroup	Intervention Mean	Intervention SD	Intervention Total	Control Mean	Control SD	Control Total	Weight	Std. Mean Difference IV, Random, 95% CI
**1.8.1 <3months**								
Druss 2018	34.49	11.15	198	34.25	11.97	202	16.4%	0.02 [-0.18, 0.22]
Goldberg 2013	50.5	11.1	28	43.2	10.7	30	10.7%	0.66 [0.13, 1.19]
**Subtotal (95% CI)**			**226**			**232**	**27.0%**	**0.29 [-0.33, 0.91]**
Heterogeneity: Tau² = 0.16; Chi² = 4.93, df = 1 (P = 0.03); I² = 80%								
Test for overall effect: Z = 0.92 (P = 0.36)								
**1.8.2 3-6months**								
Barley 2014	32.4	11.2	35	35.4	11.8	39	11.9%	-0.26 [-0.72, 0.20]
Ell 2017	44.54	1.03	178	43.67	1.02	170	16.0%	0.85 [0.63, 1.07]
Miklavcic 2020	56.69	8.37	65	53.88	7.67	56	13.6%	0.35 [-0.01, 0.71]
**Subtotal (95% CI)**			**278**			**265**	**41.5%**	**0.34 [-0.28, 0.96]**
Heterogeneity: Tau² = 0.27; Chi² = 20.11, df = 2 (P < 0.0001); I² = 90%								
Test for overall effect: Z = 1.07 (P = 0.28)								
**1.8.3 >6months**								
Ell 2010	47.31	11.48	193	43.6	12.46	194	16.3%	0.31 [0.11, 0.51]
Sajatovic 2017	42.05	11.1	100	39.58	11.4	100	15.1%	0.22 [-0.06, 0.50]
**Subtotal (95% CI)**			**293**			**294**	**31.4%**	**0.28 [0.12, 0.44]**
Heterogeneity: Tau² = 0.00; Chi² = 0.27, df = 1 (P = 0.61); I² = 0%								
Test for overall effect: Z = 3.35 (P = 0.0008)								
**Total (95% CI)**			**797**			**791**	**100.0%**	**0.31 [0.04, 0.58]**
Heterogeneity: Tau² = 0.11; Chi² = 39.32, df = 6 (P < 0.00001); I² = 85%								
Test for overall effect: Z = 2.23 (P = 0.03)								
Test for subgroup differences: Chi² = 0.03, df = 2 (P = 0.98), I² = 0%								

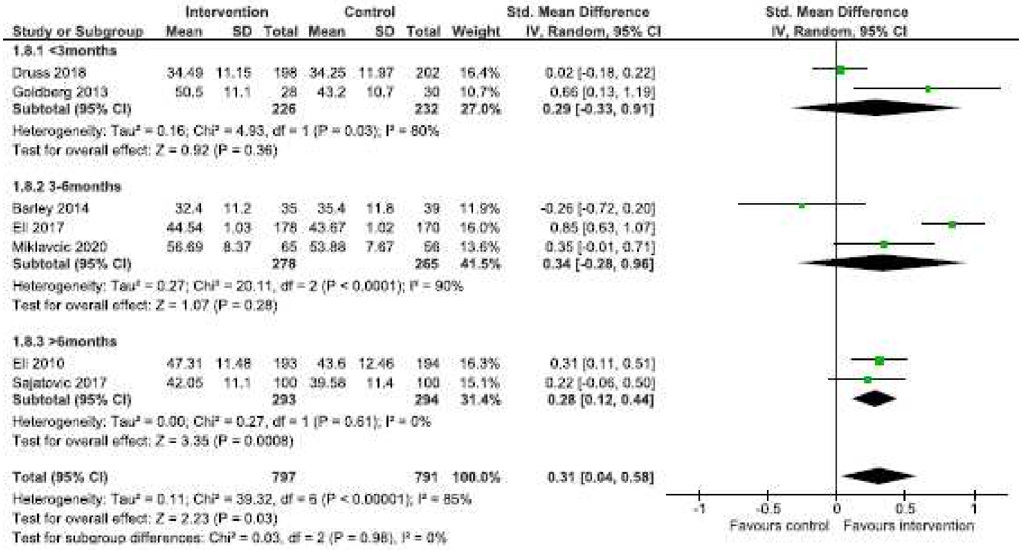

Clinical endpoints – maintenance by intervention length

Study or Subgroup	Intervention Mean	Intervention SD	Intervention Total	Control Mean	Control SD	Control Total	Weight	Std. Mean Difference IV, Random, 95% CI
**1.4.1 <3months**								
Bogner 2008	127.3	17.7	32	141.3	18.8	32	11.1%	-0.76 [-1.27, -0.25]
Griva 2019	8.78	1.54	20	9.5	2.05	24	8.7%	-0.38 [-0.98, 0.21]
**Subtotal (95% CI)**			**52**			**56**	**19.8%**	**-0.60 [-0.99, -0.21]**
Heterogeneity: Tau² = 0.00; Chi² = 0.86, df = 1 (P = 0.35); I² = 0%								
Test for overall effect: Z = 3.04 (P = 0.002)								
**1.4.2 3-6months**								
Naik 2019	8.7	1.6	108	8.9	2	78	21.9%	-0.11 [-0.40, 0.18]
**Subtotal (95% CI)**			**108**			**78**	**21.9%**	**-0.11 [-0.40, 0.18]**
Heterogeneity: Not applicable								
Test for overall effect: Z = 0.75 (P = 0.45)								
**1.4.3 >6months**								
Ali 2020	8.73	1.79	185	8.71	1.69	193	29.0%	0.01 [-0.19, 0.21]
Ell 2010	8.34	2.04	193	8.5	2.17	194	29.2%	-0.08 [-0.28, 0.12]
**Subtotal (95% CI)**			**378**			**387**	**58.3%**	**-0.03 [-0.17, 0.11]**
Heterogeneity: Tau² = 0.00; Chi² = 0.36, df = 1 (P = 0.55); I² = 0%								
Test for overall effect: Z = 0.45 (P = 0.65)								
**Total (95% CI)**			**538**			**521**	**100.0%**	**-0.16 [-0.36, 0.04]**
Heterogeneity: Tau² = 0.02; Chi² = 8.53, df = 4 (P = 0.07); I² = 53%								
Test for overall effect: Z = 1.59 (P = 0.11)								
Test for subgroup differences: Chi² = 7.30, df = 2 (P = 0.03), I² = 72.6%								

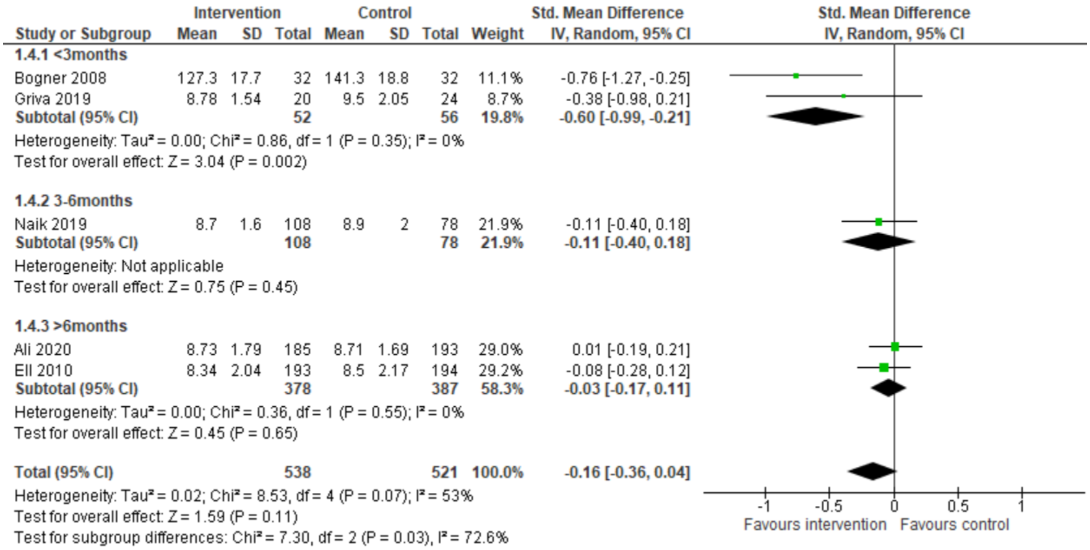

Psychological distress (depression) – maintenance by intervention length

Study or Subgroup	Intervention Mean	Intervention SD	Intervention Total	Control Mean	Control SD	Control Total	Weight	Std. Mean Difference IV, Random, 95% CI
**1.11.1 <3months**								
Boeschoten 2017	20.8	10.4	64	20.1	9.2	67	12.4%	0.07 [-0.27, 0.41]
Griva 2019	5.3	4.22	20	4.22	3.88	24	9.9%	0.26 [-0.33, 0.86]
**Subtotal (95% CI)**			**84**			**91**	**22.3%**	**0.12 [-0.18, 0.42]**
Heterogeneity: Tau² = 0.00; Chi² = 0.30, df = 1 (P = 0.58); I² = 0%								
Test for overall effect: Z = 0.78 (P = 0.43)								
**1.11.2 3-6months**								
Barley 2014	9.5	4.6	32	8.8	4.8	37	11.1%	0.15 [-0.33, 0.62]
Ell 2017	1.69	0.06	178	1.63	0.06	170	13.3%	1.00 [0.77, 1.22]
Naik 2019	10.1	6.9	96	12.6	6.5	70	12.6%	-0.37 [-0.68, -0.06]
**Subtotal (95% CI)**			**306**			**277**	**37.1%**	**0.26 [-0.67, 1.20]**
Heterogeneity: Tau² = 0.65; Chi² = 51.33, df = 2 (P < 0.00001); I² = 96%								
Test for overall effect: Z = 0.55 (P = 0.58)								
**1.11.3 >6months**								
Ali 2020	0.39	0.27	185	0.53	0.37	193	13.4%	-0.43 [-0.63, -0.23]
Aragones 2019	1.11	0.72	167	1.34	0.76	161	13.3%	-0.31 [-0.53, -0.09]
Salisbury 2018	6.1	4.6	630	6.8	4.6	625	13.9%	-0.15 [-0.26, -0.04]
**Subtotal (95% CI)**			**982**			**979**	**40.6%**	**-0.28 [-0.46, -0.10]**
Heterogeneity: Tau² = 0.02; Chi² = 6.11, df = 2 (P = 0.05); I² = 67%								
Test for overall effect: Z = 3.11 (P = 0.002)								
**Total (95% CI)**			**1372**			**1347**	**100.0%**	**0.02 [-0.33, 0.36]**
Heterogeneity: Tau² = 0.22; Chi² = 113.01, df = 7 (P < 0.00001); I² = 94%								
Test for overall effect: Z = 0.10 (P = 0.92)								
Test for subgroup differences: Chi² = 5.95, df = 2 (P = 0.05), I² = 66.4%								

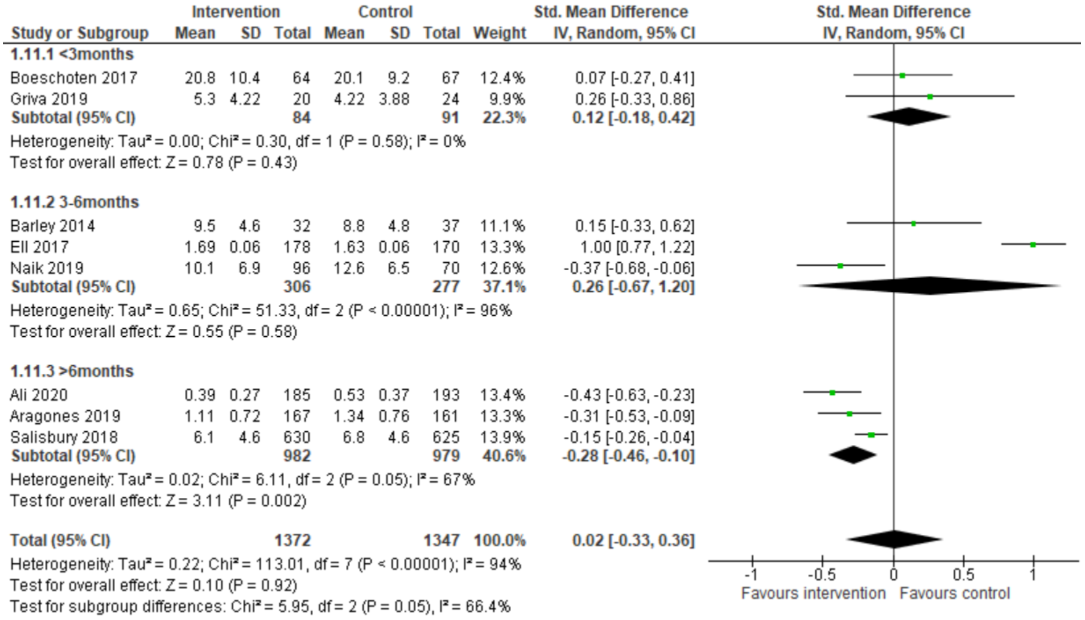

Emotional wellbeing – maintenance by intervention length

Study or Subgroup	Intervention Mean	Intervention SD	Intervention Total	Control Mean	Control SD	Control Total	Weight	Std. Mean Difference IV, Random, 95% CI
**1.9.1 <3months**								
Goldberg 2013	46.9	10.7	28	43.9	11.5	29	9.4%	0.27 [-0.26, 0.79]
Griva 2019	2.38	0.72	20	2.15	0.63	24	7.9%	0.34 [-0.26, 0.93]
**Subtotal (95% CI)**			**48**			**53**	**17.3%**	**0.30 [-0.10, 0.69]**
Heterogeneity: Tau² = 0.00; Chi² = 0.03, df = 1 (P = 0.86); I² = 0%								
Test for overall effect: Z = 1.48 (P = 0.14)								
**1.9.2 3-6months**								
Barley 2014	34.5	11.6	32	33.6	12.5	37	10.5%	0.07 [-0.40, 0.55]
Druss 2018	36.64	12.28	198	34.54	11.82	202	18.7%	0.17 [-0.02, 0.37]
Ell 2010	45.1	12.19	193	43.49	11.66	194	18.6%	0.13 [-0.06, 0.33]
Ell 2017	31.24	0.87	178	30.59	0.89	170	18.0%	0.74 [0.52, 0.95]
Kanwal 2018	41.8	13.8	114	39.4	11.9	128	16.9%	0.19 [-0.07, 0.44]
**Subtotal (95% CI)**			**715**			**731**	**82.7%**	**0.28 [0.02, 0.53]**
Heterogeneity: Tau² = 0.06; Chi² = 21.39, df = 4 (P = 0.0003); I² = 81%								
Test for overall effect: Z = 2.15 (P = 0.03)								
**1.9.3 >6months**								
**Subtotal (95% CI)**			**0**			**0**		**Not estimable**
Heterogeneity: Not applicable								
Test for overall effect: Not applicable								
**Total (95% CI)**			**763**			**784**	**100.0%**	**0.28 [0.07, 0.49]**
Heterogeneity: Tau² = 0.05; Chi² = 21.42, df = 6 (P = 0.002); I² = 72%								
Test for overall effect: Z = 2.63 (P = 0.009)								
Test for subgroup differences: Chi² = 0.01, df = 1 (P = 0.93), I² = 0%								

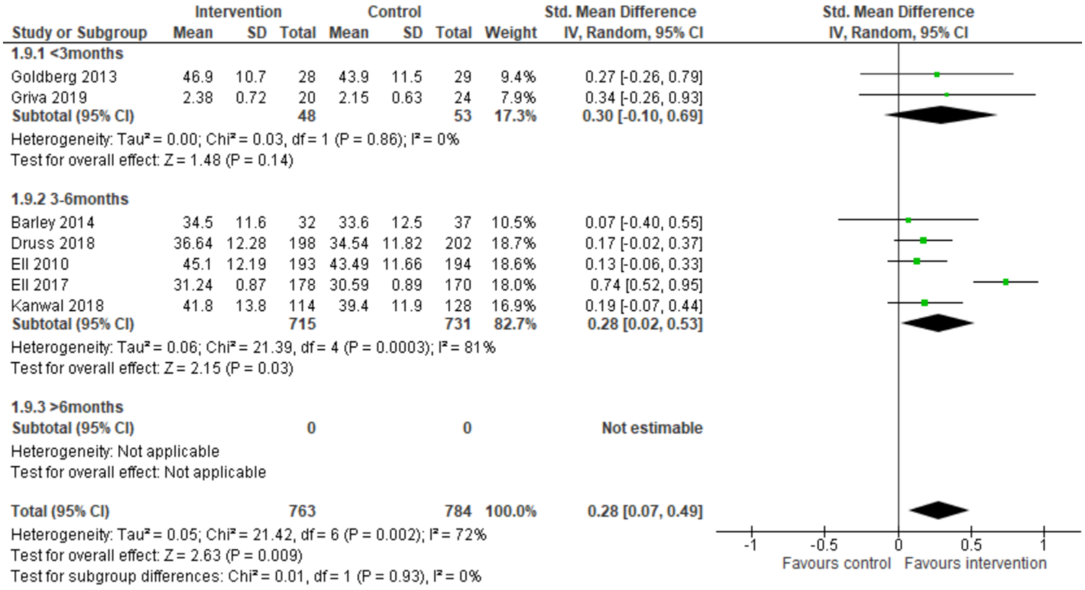

Supplementary table 4 significant effects forest plots

Psychological distress (depression) – post-intervention MLTCs combination type

	Intervention			Control				Std. Mean Difference
**Study or Subgroup**	**Mean**	**SD**	**Total**	**Mean**	**SD**	**Total**	**Weight**	**IV, Random, 95% CI**
**1.10.1 Physical-Physical**								
Garvey 2015	6.32	4.19	22	7.82	3.83	22	4.3%	-0.37 [-0.96, 0.23]
House 2018	2.4	1.87	30	1.1	1.8	29	4.5%	0.70 [0.17, 1.23]
Markle-Reid 2018	4.67	4.83	71	5.71	5.24	65	5.0%	-0.21 [-0.54, 0.13]
Miklavcic 2020	4.94	4.46	63	5.82	4.93	50	4.9%	-0.19 [-0.56, 0.19]
Park 2014	5.3	3.4	22	7.6	5.8	21	4.3%	-0.48 [-1.09, 0.13]
Salisbury 2018	6.1	4.4	654	6.6	4.5	641	5.3%	-0.11 [-0.22, -0.00]
**Subtotal (95% CI)**			**862**			**828**	**28.2%**	**-0.11 [-0.34, 0.12]**
Heterogeneity: $Tau^2 = 0.04$; $Chi^2 = 11.64$, df = 5 (P = 0.04); $I^2 = 57\%$								
Test for overall effect: Z = 0.93 (P = 0.35)								
**1.10.2 Physical-Mental**								
Ali 2020	0.52	0.38	192	0.82	0.54	202	5.2%	-0.64 [-0.84, -0.44]
Aragones 2019	1.29	0.77	167	1.42	0.73	161	5.2%	-0.17 [-0.39, 0.04]
Barley 2014	10.3	3.8	35	9.2	4.6	39	4.7%	0.26 [-0.20, 0.71]
Boeschoten 2017	20.3	8.8	74	21	9.1	78	5.0%	-0.08 [-0.40, 0.24]
Bogner 2008	9.9	10.7	32	19.3	15.2	32	4.5%	-0.71 [-1.21, -0.20]
Cummings 2019	6.3	5.9	67	7.9	7	72	5.0%	-0.25 [-0.58, 0.09]
Ell 2017	1.02	0.07	178	1.15	0.07	170	5.1%	-1.85 [-2.10, -1.60]
Katon 2010	0.83	0.68	105	1.14	0.66	106	5.1%	-0.46 [-0.73, -0.19]
Kroenke 2009	1.14	0.69	123	1.69	0.74	127	5.1%	-0.77 [-1.02, -0.51]
Morgan 2013	7.1	0.8	164	9	0.9	146	5.1%	-2.23 [-2.52, -1.95]
Naik 2019	10.9	6.1	107	12.4	6	80	5.0%	-0.25 [-0.54, 0.04]
Ross 2005	11.8	9.93	15	16.78	7.89	9	3.6%	-0.52 [-1.36, 0.32]
Sajatovic 2017	15.92	10	100	18.55	8.8	100	5.1%	-0.28 [-0.56, 0.00]
Schneider 2016	12.5	14.8	15	12.3	4.1	14	3.9%	0.02 [-0.71, 0.75]
Wilson 2018	17.46	6.16	22	20.5	5.3	25	4.3%	-0.52 [-1.11, 0.06]
**Subtotal (95% CI)**			**1396**			**1361**	**71.8%**	**-0.58 [-0.94, -0.21]**
Heterogeneity: $Tau^2 = 0.47$; $Chi^2 = 278.61$, df = 14 (P < 0.00001); $I^2 = 95\%$								
Test for overall effect: Z = 3.10 (P = 0.002)								
**1.10.3 Mental-Mental**								
**Subtotal (95% CI)**			**0**			**0**		**Not estimable**
Heterogeneity: Not applicable								
Test for overall effect: Not applicable								
**Total (95% CI)**			**2258**			**2189**	**100.0%**	**-0.45 [-0.73, -0.16]**
Heterogeneity: $Tau^2 = 0.38$; $Chi^2 = 366.51$, df = 20 (P < 0.00001); $I^2 = 95\%$								
Test for overall effect: Z = 3.11 (P = 0.002)								
Test for subgroup differences: $Chi^2 = 4.57$, df = 1 (P = 0.03), $I^2 = 78.1\%$								

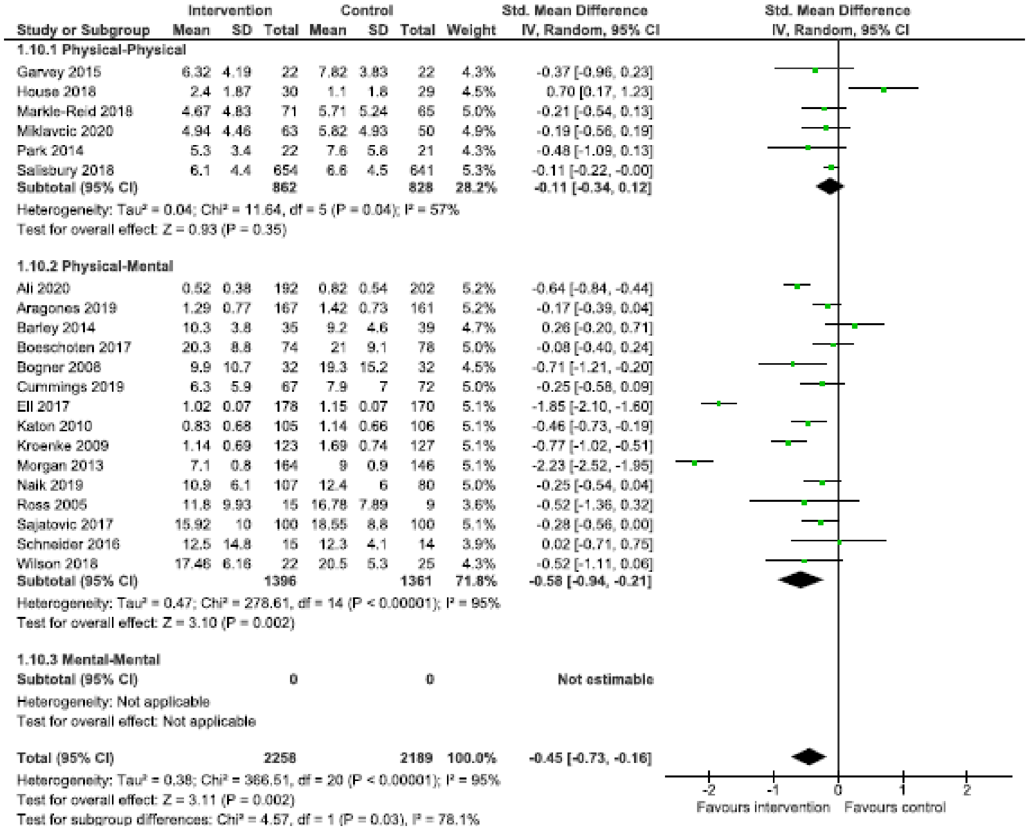

Supplement: Supplementary data [file bmjopen-2023-081104supp003.pdf]
