# Supplementary material for: Effectiveness of complex behaviour change interventions tested in randomised controlled trials for people with multiple long-term conditions (M-LTCs): systematic review with meta-analysis
Source: BMJ Open. 2024 Jun 16;14(6):e081104. doi: 10.1136/bmjopen-2023-081104 (PMC11184186; doi:10.1136/bmjopen-2023-081104)
Supplement: Supplementary data [file bmjopen-2023-081104supp006.pdf]

Study	Outcome	Effective	Intervention type	Intervention length	M-LTCs combination
Blank, 2011	Clinical endpoint	No	Self-management	>6months	Physical-Mental
Chwastiak, 2018	Clinical endpoint	No	Collaborative care	3-6months	Physical-Mental
Ell, 2010	Psychological distress (depression)	Yes	Collaborative care	>6months	Physical-Mental
Fisher, 2020	Functioning	No	Self-management	3-6months	Physical-Physical
Guo, 2020	N/A	N/A	Collaborative care	>6months	Physical-Physical
Hernandez, 2021	N/A	N/A	Self-management	3-6months	Physical-Physical
Kamradt, 2019	Quality of life	No	Collaborative care	>6months	Physical-Physical
Katon, 2004	Psychological distress (depression)	Yes	Collaborative care	>6months	Physical-Mental
Khunti, 2021	Behaviour change	Yes	Self-management	>6months	Physical-Physical
Koesoemadinata, 2021	N/A	N/A	Cognitive and/or Behavioural activation	3-6months	Physical-Physical
Lear, 2021	N/A	N/A	Self-management	>6months	Physical-Physical
Lenferink, 2019	Clinical endpoint	No	Self-management	>6months	Physical-Physical
Merlin, 2018	Pain	No	Self-management	<3months	Physical-Physical
Ose, 2019	N/A	N/A	Self-management	>6months	Physical-Physical
O'Toole, 2021	Quality of life<br>Behaviour change	No<br>No	Self-management	<3months	Physical-Physical
Pibernik, 2009	Psychological distress (depression)<br>Clinical endpoint	No<br>No	Self-management	<3months	Physical-Mental
Rose, 2018	N/A	N/A	Cognitive and/or Behavioural activation	>6months	Physical-Physical
Takahashi, 2012	N/A	N/A	Self-management	>6months	Physical-Physical
Taylor, 2003	Clinical endpoint	Yes	Collaborative care	>6months	Physical-Physical
Vera 2010	Psychological disress (depression)<br>Functioning	Yes<br>Yes	Collaborative care	3-6months	Physical-Mental
Wakefield, 2011	Clinical endpoint	Yes	Self-management	3-6months	Physical-Physical
Williams, 2012	Behaviour change	No	Self-management	3-6months	Physical-Physical
