# Supplementary material for: Effectiveness of complex behaviour change interventions tested in randomised controlled trials for people with multiple long-term conditions (M-LTCs): systematic review with meta-analysis
Source: BMJ Open. 2024 Jun 16;14(6):e081104. doi: 10.1136/bmjopen-2023-081104 (PMC11184186; doi:10.1136/bmjopen-2023-081104)
Supplement: Supplementary data [file bmjopen-2023-081104supp007.pdf]

Supplementary Table 4. Pooled effects of outcomes, stratified by types of long-term condition combination types.

	Physical-Physical			Physical-Mental		
**Post-intervention**	*n*	SMD [95% CI]	I^2	*n*	SMD [95% CI]	I^2
Quality of life	3	0.16 [-0.06, 0.39]	65%	5	0.10 [-0.22, 0.41]	65%
Clinical endpoints	3	-0.29 [-0.61, 0.02]	61%	8	-0.20 [-0.53, 0.12]	90%
Behaviour change	2	N/A	N/A	4	-0.14 [-0.32, 0.05]	14%
Functioning	5	0.03 [-0.15, 0.20]	41%	8	0.04 [-0.09, 0.17]	45%
Psychological distress (depression)	6	-0.11 [-0.34, 0.12]	57%	15	**-0.58 [-0.94, -0.21]**	95%
Psychological distress (anxiety)	4	-0.10 [-0.19, 0.00]	0%	5	-0.17 [-0.48, 0.13]	63%
Emotional wellbeing	1	N/A	N/A	6	0.30 [-0.01, 0.61]	87%
**Maintenance**						
Functioning	1	N/A	N/A	6	0.03 [-0.07, 0.13]	0%
Psychological distress (depression)	1	N/A	N/A	7	0.05 [-0.42, 0.51]	94%
