# Supplementary material for: Effectiveness of complex behaviour change interventions tested in randomised controlled trials for people with multiple long-term conditions (M-LTCs): systematic review with meta-analysis
Source: BMJ Open. 2024 Jun 16;14(6):e081104. doi: 10.1136/bmjopen-2023-081104 (PMC11184186; doi:10.1136/bmjopen-2023-081104)
Supplement: Author's manuscript [file bmjopen-2023-081104.draft_revisions.pdf]

BMJ Open is committed to open peer review. As part of this commitment we make the peer review history of every article we publish publicly available.

When an article is published we post the peer reviewers' comments and the authors' responses online. We also post the versions of the paper that were used during peer review. These are the versions that the peer review comments apply to.

The versions of the paper that follow are the versions that were submitted during the peer review process. They are not the versions of record or the final published versions. They should not be cited or distributed as the published version of this manuscript.

BMJ Open is an open access journal and the full, final, typeset and author-corrected version of record of the manuscript is available on our site with no access controls, subscription charges or pay-per-view fees (http://bmjopen.bmj.com).

If you have any questions on BMJ Open's open peer review process please email info.bmjopen@bmj.com

BMJ Open

Intervention effectiveness and outcome appropriateness in testing complex interventions for people with multiple long-term conditions (M-LTCs): a meta-analysis and systematic review

Journal:	*BMJ Open*
Manuscript ID	bmjopen-2023-081104
Article Type:	Original research
Date Submitted by the Author:	18-Oct-2023
Complete List of Authors:	Rookes, Tasmin; University College London Research Department of Primary Care and Population Health<br>Nimmons, Danielle; University College London Research Department of Primary Care and Population Health<br>Armstrong, Megan; Queen Mary University of London Wolfson Institute of Population Health<br>Davies, Laura; University College London Research Department of Primary Care and Population Health<br>Ross, Jamie; Queen Mary University of London Wolfson Institute of Population Health<br>Hopkins, Jane; Public Contributor<br>Mistry, Manoj; Public Contributor<br>Walters, Kate; University College London, Primary Care and Population Health<br>Taylor, Stephanie; Queen Mary University of London, Wolfson Institute of Population Health<br>Frost, Rachael; University College London Research Department of Primary Care and Population Health, Research Department of Primary Care and Population Health
Keywords:	Meta-Analysis, Systematic Review, Aging, Patient Reported Outcome Measures, Primary Care < Primary Health Care, PUBLIC HEALTH

SCHOLARONE™
Manuscripts

I, the Submitting Author has the right to grant and does grant on behalf of all authors of the Work (as defined in the below author licence), an exclusive licence and/or a non-exclusive licence for contributions from authors who are: i) UK Crown employees; ii) where BMJ has agreed a CC-BY licence shall apply, and/or iii) in accordance with the terms applicable for US Federal Government officers or employees acting as part of their official duties; on a worldwide, perpetual, irrevocable, royalty-free basis to BMJ Publishing Group Ltd ("BMJ") its licensees and where the relevant Journal is co-owned by BMJ to the co-owners of the Journal, to publish the Work in this journal and any other BMJ products and to exploit all rights, as set out in our licence.

The Submitting Author accepts and understands that any supply made under these terms is made by BMJ to the Submitting Author unless you are acting as an employee on behalf of your employer or a postgraduate student of an affiliated institution which is paying any applicable article publishing charge ("APC") for Open Access articles. Where the Submitting Author wishes to make the Work available on an Open Access basis (and intends to pay the relevant APC), the terms of reuse of such Open Access shall be governed by a Creative Commons licence – details of these licences and which Creative Commons licence will apply to this Work are set out in our licence referred to above.

Other than as permitted in any relevant BMJ Author's Self Archiving Policies, I confirm this Work has not been accepted for publication elsewhere, is not being considered for publication elsewhere and does not duplicate material already published. I confirm all authors consent to publication of this Work and authorise the granting of this licence.

For peer review only

Intervention effectiveness and outcome appropriateness in testing complex interventions for people with multiple long-term conditions (M-LTCs): a meta-analysis and systematic review

Tasmin Rookes, Danielle Nimmons, Rachael Frost, Megan Armstrong, Laura Davies, Jamie Ross, Jane Hopkins, Manoj Mistry, Stephanie JC Taylor, and Kate Walters

Corresponding Author:
Tasmin Rookes: t.rookes@ucl.ac.uk

Abstract:

Introduction

Prevalence of multiple long-term conditions (M-LTCs) increases as adults age and impacts quality of life and health outcomes. To help people to manage these conditions, complex behaviour change interventions are utilised, often based on research conducted in those with single LTCs. However, the needs of those with M-LTCs can differ due to complex health decision making and engagement with multiple health and care teams.

Objectives

The aim of this review is to identify whether current interventions are effective for people living with M-LTCs, and which outcomes are most appropriate to detect this change.

Methods

Five databases (MEDLINE, Embase, PsychInfo, CINAHL and Web of Science) were systematically searched, between 1999-2022, to identify RCTs evaluating effectiveness of behaviour change interventions in people with M-LTCs. Intervention characteristics, intervention effectiveness, and outcome measures were meta-analysed and narratively synthesised.

Results

Fifty-three eligible articles were included. Emotional wellbeing and psychological distress outcomes were most amenable to change (Figure 1.), particularly for interventions with a collaborative care approach. Interventions targeting those with a physical and mental health condition and those with cognitive and/or behavioural activation approach saw larger reductions in psychological distress outcomes. Interventions that lasted for longer than 6-months significantly improved the widest variety of outcomes.

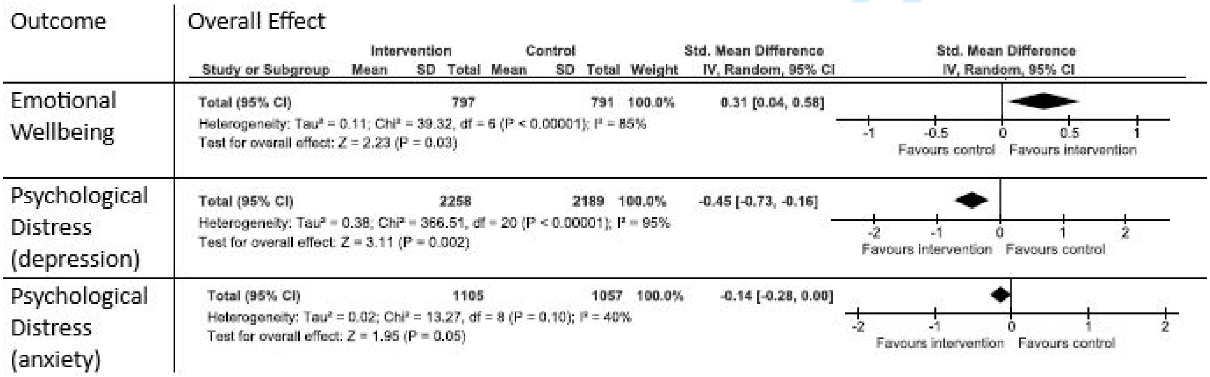

Figure 1. Pooled effect size for emotional wellbeing, and psychological distress measures, which were most amenable to change.

Conclusion

Complex interventions can be successfully delivered to those with M-LTCs. These are most effective at reducing psychological distress in those with physical and mental LTCs.

Further research is needed to identify the effective components of interventions for people with two or more physical LTCs and which outcome is most appropriate for detecting this change.

Introduction:

Living with multiple long-term conditions (M-LTCs) is defined as an individual having two or more health conditions at the same time. Globally, the presence of M-LTCs is reported to be 27.2% in all adults, rising to 67.0% in those aged 74 and over (1). With an ageing population, this is predicted to rise in those over 65 in the UK, from 54% in 2015, to 68% in 2035 (2). In comparison with the general population, those with M-LTCs are found to have lower quality of life and higher mortality risk (3, 4), as well as greater healthcare costs and utilisation (5). As prevalence increases, nationally and globally, the pressure and reliance on healthcare services, people living with the conditions, and their carers also increases, making the challenge of managing M-LTCs a priority for current research agendas and healthcare systems, such as the NHS and the James Lind Alliance (1, 6, 7).

Despite this increased focus on managing M-LTCs, many of the beneficial complex interventions designed to help people with M-LTCs are based on findings from research and interventions aimed at people with single health conditions and applied more widely to those with M-LTCs. Complex interventions contain multiple interacting components and often depend on the behaviours of those delivering and receiving the intervention and need to be adapted to the specific participant group and setting (8). The chronic disease self-management program (CDSMP) has found mixed effectiveness in people with M-LTCs, depending on the combination and type of health conditions participants experience, being more effective for people with physical health conditions and probable depression (9, 10). Manging M-LTCs presents a different challenge to those with single LTCs due to the often-fragmented care across multiple primary and secondary care teams and the varied and competing needs of the individual (11, 12). These needs include complex priority setting and decision-making to adhere to multiple healthcare regimes and weighing up conflicting advice from different specialties (13). This is reflected in poorer outcomes when receiving interventions to help with the management of their M-LTCs, suggesting that the specific needs of these people are not being addressed by current interventions (14).

Systematic reviews, highlight that the effectiveness of complex interventions varies depending on the combination of LTCs, the type of intervention being used, and the outcome measure being used to assess effectiveness (9, 14). Determining the most appropriate outcome measure is particularly difficult in this population, given the wide variety of outcomes that may be relevant depending on the conditions and challenges faced by the population e.g., clinical outcomes, wellbeing, quality of life, and distress. Therefore, it is essential we first identify whether complex interventions currently being tested in people with M-LTCs are effective for this population and then explore which outcome measure is most sensitive to detecting a significant positive change in outcomes.

Aims:

1. To evaluate the evidence around the effectiveness of behaviour change interventions in people with M-LTCs.
2. To identify the outcome measures most sensitive to change, to identify effectiveness of complex behaviour change interventions in people with M-LTCs.

Methods:

This review was follows the PRISMA guidelines (supplementary table 1) and registered on PROSPERO (ID: CRD42021287847).

Search

For peer review only

The databases MEDLINE, Embase, PsychInfo, CINAHL and Web of Science were systematically searched from January 1999 to January 2022, and an example search term list can be seen in supplementary figure 1. This cutoff date was chosen, to align with the dates of papers identified in a previous Cochrane systematic review for complex interventions in people with M-LTCs (12) and to ensure that the findings were applicable to current healthcare settings. The identified articles were exported into Endnote and duplicates removed. 10% of identified titles and abstracts were independently screened by two reviewers (T.A.R. and D.N.) against the inclusion criteria, using Rayyan (15). As there was a less than 5% conflict (n=41/938; 4.37%), single screening was conducted by T.A.R. for the remaining titles and abstracts. For full text screening, 20% were independently reviewed by T.A.R. and D.N., and again, there was only a 5% discrepancy (n=2/40; 5%), and so the remainder were independently screened by T.A.R. for eligibility.

Inclusion Criteria
Participants: People with two or more physical and/or mental health conditions.
Intervention: A complex behaviour change intervention to help with the management of health conditions aiming to improve individual health outcomes, which could be targeted at the individual or organisational level.
Comparator: Treatment as usual.
Outcomes: Any individual health outcomes, such as quality of life, emotional wellbeing, clinical outcomes, psychological distress, behaviour change, and pain.
Setting: Interventions delivered in primary and community care settings.
Timeframe: published between 1st January 1999 and 17th January 2022.

Data extraction and Outcome measures
Study characteristics were extracted by L.D. and checked by T.A.R. for completeness. Outcome measures were extracted by T.A.R. and checked by D.N. for completeness. For all studies, we extracted trial authors, year of publication, country, intervention description and length, study follow-up length, M-LTCs targeted, sample size at each timepoint, means and standard deviations for relevant outcome measures. Outcome measures were grouped into six categories, 1) quality of life, 2) clinical endpoints, 3) behaviour change, 4) pain, 5) wellbeing, and 6) psychological distress. Where data were not reported in the paper, we contacted the authors for further details (11 approached, 1 responded providing further data).

Study quality assessment
Study quality of the eligible trials was assessed using the Risk of Bias 2 (RoB 2) tool (16). Initial assessment for 20% of eligible studies (n=12) was completed independently by two reviewers (T.A.R. and D.N.), with agreement for 8/12 trials (75%). Therefore, a further 10% (n=6) were independently reviewed, with 100% agreement between the two reviewers. The remaining 38 trials were assessed for quality by T.A.R. Study quality was used to inform narrative synthesis but was not used to exclude studies.

Data analysis
Where there were 3 or more studies reporting the same outcome, we conducted a meta-analysis. RevMan 5.4 (17) was used to calculate, and pool mean differences or standardised mean differences and 95% confidence intervals, using inverse-variance random-effects models for all six continuous outcome categories. Psychological distress was separated into anxiety and depression, as many studies reported both outcomes and we felt the two conditions were different.

Interventions were grouped into three types, self-management, collaborative care, and cognitive and/or behavioural activation, based on their predominant features (table 1). Exploratory supplementary subgroup analyses were carried out to explore 1) Intervention type: self-management, collaborative care, and cognitive and/or behavioural activation), 2)

For peer review only

Length of intervention: <3-months, 3-6-months, and 6+months, for outcomes immediately post-intervention delivery, 3) Maintenance of effect: Time since intervention was complete to final follow-up, grouped as <3 months, 3+months post intervention, and 4) Type of M-LTCs combinations: physical-physical, mental-physical and mental-mental.

Heterogeneity was assessed across studies using I^2 statistics, with proportions greater than 25%, 50%, and 75% considered to have low, moderate, and high heterogeneity, respectively (18). Studies for which data were not reported in a form that could be meta-analysed were synthesised narratively.

Table 1. Example interventions and their characteristics for allocation into the three types of intervention for supplementary subgroup analysis.

Intervention Type	Example of intervention and characteristics
Self-management	Psychoeducational interventions, with goal setting and problem solving, to help people manage the symptoms and improve their health outcomes related to their health conditions. Also included telemonitoring approaches, where participants received information and had their symptoms monitored over time.
Collaborative care	Interventions targeting the participants, the clinical team, and the system they are embedded within. Also included stepped care, where participants started with one treatment pathway and depending on progress, could be stepped up to more intensive treatment.
Cognitive and/or behavioural activation	Often use motivational interviewing, cognitive behavioural therapy, behavioural activation, and/or problem solving to empower people to help people understand the association between health conditions and cognitive processes and create healthier behaviours for future problems.

Results

After searching and de-duplication, 9,354 titles and abstracts were screened. 200 full text articles were screened and 53 included in the synthesis (see figure 2 and supplementary figure 2).

Study characteristics

Across 53 RCTs there were a total of 14,740 participants with study sizes ranging from 25 to 3324. The studies were published between 2001 and 2021, with a steady increase in publications from 2009 to the present day, with 58% published since 2017. Studies were conducted in the United States (n=22), Canada (n=7), Australia (n=5), UK (n=5), Netherlands (n=3), Spain (n=2), Ireland (n=2), and the remaining eight in Singapore, China, Nigeria, Germany, Indonesia, South Korea, Croatia, and India (one study was conducted across two countries). Interventions were aimed at people with a combination of physical and mental health conditions (n=27), two or more physical health conditions (n=25), and 1 study was for people with a combination of two mental health conditions. The health conditions included diabetes, depression, schizophrenia, coronary heart disease, chronic pain, HIV, and hypertension, as well as interventions which were open to people with multiple different health conditions.

The types of behaviour change interventions could be grouped into three categories: self-management (n=28), collaborative care (n=15), and cognitive and/or behavioural activation (n=10). Interventions were heterogeneous in terms of the person delivering them; they were delivered by individual or combinations of nurses, doctors, psychologists, other health-care professionals, and trained peer support leaders. The mode of delivery varied from face-to-face, in clinics or homes, telephone, and through digital platforms. The length

For peer review only

of these interventions ranged from 3 weeks to 2 years, with most lasting between 3-12 months. Post-intervention, maintenance follow-up lengths varied from 6-weeks to 12-months. A summary of the studies and the interventions can be found in supplementary table 2.

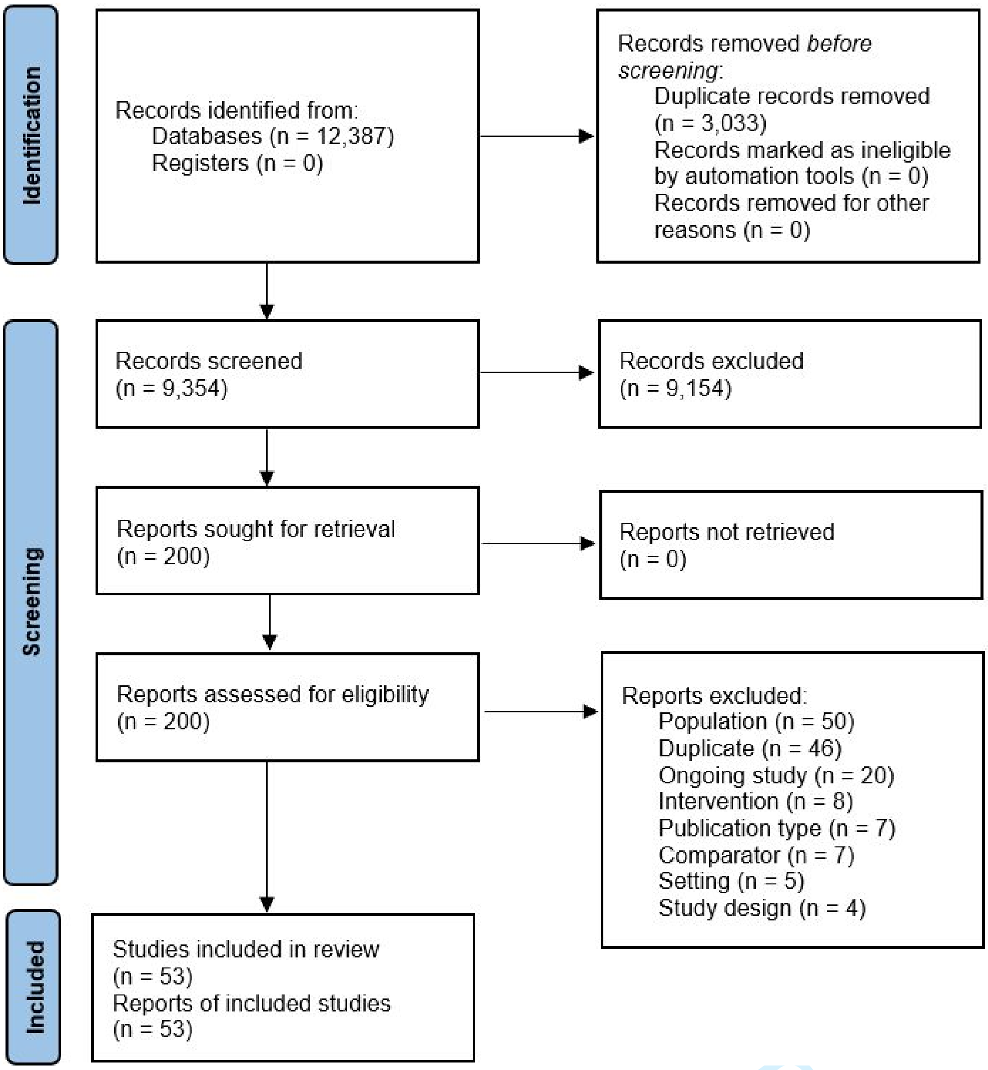

Figure 2. Prisma flow chart. (19)

Quality assessment
Overall study quality was very variable, 29 studies were rated as low risk of bias, the rating some concerns regarding risk of bias in 20 studies and four studies were assessed as having a high risk of bias. All the studies measuring quality of life and pain, half of the studies measuring clinical endpoints, 80% measuring functioning and 40% of those measuring psychological distress had at least some concerns with risk of bias (see supplementary table 2).

Outcome characteristics
For the primary outcome, 10 measured a clinical endpoint relevant to the included LTCs (e.g., HbA1c, blood pressure, cholesterol), 10 studies used a psychological distress measure, five used a behaviour change measure (e.g., alcohol use (AUDIT), five a functioning measure (e.g., physical functioning), four studies measured quality of life, one study measured pain, no studies used emotional wellbeing as the primary outcome, and six studies used a composite of these measures (often psychological distress combined

with a clinical endpoint) and 12 studies used an outcome measure that did not fit into these categories e.g., acceptability of intervention.

Main analysis: Meta-analysis of individual outcomes

Outcome data assessed immediately post-intervention was available from 31 studies for meta-analysis across the seven outcome types. Effectiveness of interventions was detected when emotional wellbeing (0.31 [0.04, 0.58]) and psychological distress, both depression (-0.45 [-0.73, -0.16]) and anxiety (-0.14 [-0.28, -0.00]), were measured. The effect sizes for psychological distress (depression) and emotional wellbeing (both post-intervention and maintenance) were moderate, but heterogeneity was high. For psychological distress (anxiety) the effect size was small and heterogeneity moderate. No other outcomes detected a significant effect from the interventions (see table 2).

Outcome data assessed at follow-up, to measure the effect of maintenance, was available from 15 studies for meta-analysis across all outcomes. Effectiveness of interventions was only detected when emotional wellbeing was measured (0.28 [0.07, 0.49]), with moderate heterogeneity. All other outcomes did not detect a significant effect (see table 2).

Table 2. Summary of pooled effects of outcome measures, for each outcome, assessed immediately post-intervention and at a follow-up/maintenance timepoint.

	Overall effect		
**Post-intervention**	*n*	SMD [95% CI]	I^2
Quality of life	8	0.13 [-0.04, 0.30]	59%
Clinical endpoints	11	-0.22 [-0.47, 0.03]	88%
Behaviour change	6	0.06 [-0.18, 0.29]	78%
Pain	4	-0.32 [-0.70, 0.07]	77%
Functioning	14	0.05 [-0.06, 0.15]	42%
Psychological distress (depression)	21	**-0.45 [-0.73, -0.16]**	95%
Psychological distress (anxiety)	9	**-0.14 [-0.28, 0.00]**	40%
Emotional wellbeing	7	**0.31 [0.04, 0.58]**	85%
**Maintenance**			
Quality of life	3	0.61 [-1.54, 2.75]	99%
Clinical endpoints	5	-0.16 [-0.36, 0.04]	53%
Behaviour change	3	0.02 [-0.10, 0.15]	23%
Functioning	8	0.05 [-0.05, 0.16]	16%
Psychological distress (depression)	8	0.02 [-0.33, 0.36]	94%
Psychological distress (anxiety)	4	-0.08 [-0.18, 0.02]	0%
Emotional wellbeing	7	**0.28 [0.07, 0.49]**	72%

n is number of studies measuring the outcome. Positive results for psychological distress, indicated by a decrease in scores and for emotional wellbeing by an increase in scores.

Narrative synthesis:

For the 22 studies where meta-analysis was not possible due to data not being available, 15 measured at least one of the seven outcomes, and found results mostly consistent with the meta-analysis. Seven studies found positive intervention effects, which measured psychological distress (n=3), clinical endpoints (n=2), behaviour change (n=1), and

For peer review only

functioning (n=1) (see supplementary table 3). Studies measuring quality of life (n=2) found no effects.

Supplementary analysis:

We conducted three supplementary analyses to explore outcomes by intervention type, duration of intervention and MLTC combination type:

1) *Intervention type*

As interventions were heterogeneous, a further supplementary meta-analysis was conducted, grouping interventions by type of approach.

For interventions with a self-management approach, none of the outcomes detected a significant benefit immediately post intervention and only emotional wellbeing at follow-up significantly improved (0.34 [0.03, 0.65]), but with high heterogeneity, see table 3.

For interventions with a collaborative care approach, significant differences post-intervention were detected for quality of life (0.15 [0.02, 0.27]), with low heterogeneity, emotional wellbeing (0.31 [0.11, 0.51]), and psychological distress (depression; -0.82 [-1.40, -0.24]), with high heterogeneity, (table 3). When measuring maintenance effects, significant effects were found for emotional wellbeing (0.15 [0.00, 0.31]), with no heterogeneity, and psychological distress (depression; -0.28 [-0.55, -0.01]), again with high heterogeneity.

For interventions with a cognitive and/or behavioural activation approach, post-intervention effects were seen for psychological distress (depression) only (-0.24 [-0.38, -0.09]), with no heterogeneity. For maintenance effects, functioning was significantly improved (0.78 [0.05, 1.50]).

Table 3. Pooled effects of outcomes from meta-analysis, stratified by intervention type.

	Self-management			Collaborative care			Cognitive and/or behavioural activation		
**Post-intervention**	*n*	SMD [95% CI]	I^2	*n*	SMD [95% CI]	I^2	*n*	SMD [95% CI]	I^2
Quality of life	2	0.27 [-0.77, 1.30]	86%	4	**0.15 [0.02, 0.27]**	31%	2	0.14 [-0.69, 0.98]	72%
Clinical endpoints	3	-0.29 [-0.61, 0.02]	61%	4	-0.45 [-1.00, 0.11]	95%	4	0.06 [-0.10, 0.23]	0%
Behaviour change	3	0.01 [-0.60, 0.61]	91%	1	0.07 [-0.03, 0.17]	N/A	2	0.07 [-0.23, 0.38]	0%
Functioning	9	0.05 [-0.06, 0.16]	26%	3	0.08 [-0.20, 0.35]	74%	2	0.18 [-0.53, 0.89]	72%
Psychological distress (depression)	8	-0.30 [-0.91, 0.31]	96%	6	**-0.82 [-1.40, -0.24]**	97%	7	**-0.24 [-0.38, -0.09]**	0%
Psychological distress (anxiety)	5	-0.05 [-0.23, 0.14]	0%	2	-0.24 [-0.58, 0.10]	84%	2	-0.42 [-1.26, 0.42]	71%
Emotional wellbeing	5	0.33 [-0.10, 0.76]	90%	1	**0.31 [0.11, 0.51]**	N/A	1	0.22 [-0.06, 0.50]	N/A
**Maintenance**									
Clinical endpoints	1	-0.38 [-0.98, 0.21]	N/A	3	-0.18 [-0.48, 0.12]	74%	1	-0.11 [-0.40, 0.18]	N/A
Functioning	5	0.10 [-0.02, 0.22]	0%	2	-0.07 [-0.23, 0.08]	0%	1	**0.78 [0.05, 1.50]**	N/A
Psychological distress (depression)	4	0.28 [-0.48, 1.04]	96%	2	**-0.28 [-0.55, -0.01]**	82%	2	-0.16 [-0.59, 0.28]	71%
Emotional wellbeing	5	**0.34 [0.03, 0.65]**	76%	2	**0.15 [-0.00, 0.31]**	0%	0	N/A	N/A

n is number of studies measuring the outcome. *p* value is considered significant if ≤0.05.

In the 22 studies synthesised narratively, two of the nine self-management interventions (22%), and four of the six collaborative care interventions (67%) found significant effects

on outcomes, three of which were psychological distress (depression) measures. These findings generally support the meta-analysis conclusions (see supplementary table 3).

2) *Duration of intervention*

For interventions lasting for less than 3-months, positive effects post-intervention were only detected with psychological distress (depression) measures (-0.36 [-0.57, -0.14]), with very low heterogeneity. For behaviour change outcomes, a negative effect was found, with those in the control group doing better post-intervention than those receiving the intervention (-0.22 [-0.41, -0.04]), with no heterogeneity. When exploring maintenance effects of interventions, clinical endpoints were the only outcome to detect a significant effect (-0.60 [-0.99, -0.21]), with no heterogeneity.

Significant post-intervention effects for interventions lasting between 3- and 6-months were not found for any of the outcomes. However, significant differences at maintenance were detected when emotional wellbeing was measured (0.28 [0.02, 0.53]), but heterogeneity was high.

For interventions lasting for longer than 6-months, effects were seen post-intervention for clinical endpoints (-0.15 [-0.28, -0.02]), with low heterogeneity, emotional wellbeing (0.28 [0.12, 0.44]), with no heterogeneity, and psychological distress (depression; -0.50 [-0.69, -0.30]) measures, with moderate heterogeneity, (see table 4). Maintenance effects in interventions lasting longer than 6-months were only identified with psychological distress (depression) measures (-0.28 [-0.46, -0.10]), but heterogeneity was high (table 4).

Table 4. Pooled effects of outcomes when analysed by intervention length.

	<3-months			3-6months			>6months		
**Post-intervention**	*n*	SMD [95% CI]	I^2	*n*	SMD [95% CI]	I^2	*n*	SMD [95% CI]	I^2
Quality of life	3	0.38 [-0.38, 1.13]	81%	1	**0.13 [0.03, 0.24]**	N/A	4	0.11 [-0.13, 0.35]	55%
Clinical endpoints	0	N/A	N/A	4	-0.33 [-1.30, 0.65]	96%	7	**-0.15 [-0.28, -0.02]**	44%
Behaviour change	2	-0.22 [-0.41, -0.04]	0%	2	0.08 [-0.02, 0.17]	0%	2	0.34 [-0.20, 0.87]	83%
Functioning	1	-0.56 [-1.16, 0.04]	N/A	8	0.07 [-0.02, 0.16]	0%	5	0.07 [-0.15, 0.30]	65%
Psychological distress (depression)	6	**-0.36 [-0.57, -0.14]**	7%	10	-0.42 [-0.96, 0.12]	97%	**5**	**-0.50 [-0.69, -0.30]**	62%
Psychological distress (anxiety)	3	-0.20 [-0.65, 0.26]	50%	4	-0.09 [-0.19, 0.01]	0%	2	-0.17 [-0.75, 0.41]	79%
Emotional wellbeing	2	0.29 [-0.33, 0.91]	80%	3	0.34 [-0.28, 0.96]	90%	2	**0.28 [0.12, 0.44]**	0%
**Maintenance**									
Clinical endpoints	2	**-0.60 [-0.99, -0.21]**	0%	1	-0.11 [-0.40, 0.18]	N/A	2	-0.03 [-0.17, 0.11]	0%
Functioning	1	0.26 [-0.26, 0.79]	N/A	6	0.03 [-0.10, 0.17]	31%	1	0.11 [-0.10, 0.32]	N/A
Psychological distress (depression)	2	0.12 [-0.18, 0.42]	0%	3	0.26 [-0.67, 1.20]	96%	3	**-0.28 [-0.46, -0.10]**	94%
Emotional wellbeing	2	0.30 [-0.10, 0.69]	0%	5	**0.28 [0.02, 0.53]**	81%	0	N/A	N/A

n is number of studies measuring the outcome. *p* value is considered significant if ≤0.05.

Exploring the 22 studies without data for meta-analysis, none of the 3 studies lasting less than 3-months, two of the five studies lasting between 3- and 6-months (40%), and four out of the seven studies lasting longer than 6 months (57%) had significant primary outcome effects (see supplementary table 3).

3) *M-LTCs combination type*

For peer review only

Analysis around effectiveness of interventions depending on the combination of M-LTCs types, grouped as physical-physical, physical-mental, and mental-mental was explored post-hoc, following conversations about potential impact with public contributors. The only significant effect found was post-intervention for psychological distress (depression) measures in people with combined physical and mental health conditions (-0.58 [-0.94, -0.21]), but heterogeneity was high (see supplementary table 4).

In the narrative synthesis, of those measuring a relevant outcome, three out of nine studies measuring interventions targeted at people with two physical health conditions found positive effects (33%), two of which were measuring clinical endpoints. Of those measuring effects in people with a physical and mental health condition, three out of six studies found positive effects (50%), and these were all measuring psychological distress (depression) (see supplementary table 3).

Discussion

Across the 53 included studies, types of complex interventions, LTCs targeted, and outcome measures used varied widely. Overall, there is evidence that complex interventions can improve the outcomes of people with M-LTCs, and the outcome measures most sensitive to change were emotional wellbeing and psychological distress.

In subgroup analyses, interventions that lasted for longer periods of time, and had a collaborative care approach were associated with greater beneficial outcomes than shorter interventions with a self-management focus. Interventions with a cognitive and/or behavioural activation approach and interventions targeted at people with a combination of a mental and physical health condition had better outcomes when measuring psychological distress (depression) measures.

Results in context

Developing interventions for people with single LTCs centred around psychological variables is not a new concept, with psychological distress and emotional wellbeing factors being the most amenable to change (20). This is particularly important when exploring the psychological factors, such as psychological capability, that enable a person with a health condition to believe they can engage in the necessary behaviour to improve their outcomes (21). In the absence of psychological capability, an intervention targeting this, through goal setting, problem solving, and self-efficacy, could improve wellbeing outcomes (22). These concepts for people with single LTCs appear to transfer over to those with M-LTCs, with psychological distress measures showing a significant improvement in those with a combination of physical and mental health conditions. This is perhaps unsurprising, given the likely higher psychological burden placed on those living with M-LTCs.

In people with single mental LTCs, cognitive and/or behavioural activation centred interventions have a strong evidence base from systematic reviews for reducing peoples' psychological distress and depression symptoms (23, 24). The findings from this review suggest these interventions have similar improvements for psychological distress in people with M-LTCs. Despite this, often quality of life is chosen as the primary outcome to test the effectiveness of complex interventions, when single clinical outcomes cannot be used. However, here we have shown that emotional wellbeing or psychological distress may be the outcomes more able to detect a change in people with M-LTCs.

Looking across the outcomes, the most effective intervention type for people with M-LTCs appears to be collaborative care. Collaborative care involves integrating health and care services across specialties and services to improve the outcomes of the people they are caring for (25). Other reviews have found positive effects of collaborative care interventions for people with a combination of at least one physical LTC and depression (26, 27). The mechanisms that underpin this change are thought to be two-fold, at the

For peer review only

individual level, with the person being able to manage their physical LTC better once the depressive symptoms have been reduced, and at the institutional level, with improved delivery of care with a patient centred focus (26).

Alongside the type of intervention, the length of the intervention also seems to be an important factor for effectiveness, with interventions lasting for longer than 6-months improving outcomes post-intervention. In line with guidance, when designing an intervention, the minimum clinically effective dose should be identified and empirically tested (8, 28). There is no evidence suggesting that length and effectiveness of interventions are linked in people with single LTCs. However, it may be that the complex and interacting needs of people with M-LTCs require longer interventions to ensure that the skills to self-manage and the systems to support the individual are in place (11). Also, it is worth noting that even with interventions lasting for longer than 6-months, maintenance effects are only seen for psychological distress measures, highlighting again that these outcomes are the most amenable to change (20) and that if we want other outcomes to continue to be improved, then support beyond the intervention period is probably needed.

Future Research

Whilst the findings here suggest that interventions to improve psychological distress and emotional wellbeing outcomes in those with mental LTCs have been successfully applied to those with mental and physical LTCs, the results for those with two or more physical LTCs are less conclusive. Future research needs to explore why this might be, and whether different approaches are needed, with specific intervention components, to address the needs of people with multiple physical LTCs, alongside identifying which outcomes are most appropriate to measure this mechanism of action (29).

In addition, whilst we can see that interventions lasting for more than 6-months are more effective, the findings around maintenance effects are less conclusive. This suggests more research is needed to determine how to ensure effective interventions have long lasting effects and improvements can be maintained over time once intervention periods have ended.

Limitations

Double screening of eligible papers was only completed for a percentage of the identified studies, meaning some eligible papers could have been missed. However, through piloting good consensus was found and the lead reviewer (T.R.) was most familiar with the eligibility criteria, the papers of interest, and the process of screening.

Complex interventions are difficult to define and therefore, determining whether a study was eligible based on this criterion can be a challenge. To overcome this potential bias, if there was any doubt, studies were included and discussed with a second (D.N.) and sometimes a third (R.F.) reviewer to establish consensus about eligibility, to make this process as consistent as possible.

Many of the meta-analyses results had high heterogeneity, suggesting that they may not have been comparable. A random-effects model was used for all analyses, but the results should be viewed with this in mind.

Conclusions

In conclusion, effective complex interventions to improve the outcomes of people with M-LTCs are possible. For those with a combination of physical and mental LTCs, a collaborative care or a cognitive and/or behavioural activation approach should be considered, and emotional wellbeing and psychological distress outcomes should be measured to detect effectiveness. Evidence around those with multiple physical LTCs is

For peer review only

less clear, however a collaborative care approach is likely the most appropriate, but the intervention components and outcome to measure this still need to be identified to ensure the best outcomes for people with M-LTCs.

Key Words
Multimorbidity, multiple long-term conditions, randomised controlled trial, meta-analysis, systematic review, behaviour change.

Word Count 3434

Conflicts of interests
The authors have no conflicts of interest to declare

Author Statement
TAR: Principal Investigator for the study, developed the protocol, conducted the searches, screened all the abstracts and full texts, completed outcome measure data extraction, conducted meta-analysis and narrative synthesis, wrote and finalised the manuscript.
DN: Co-investigator for the study, second reviewer for screening and quality assessment, supported with analysis and interpretation and reviewed the final manuscript.
RF: Supervised TR through the process, supported with development of project and protocol, acted as third reviewer when consensus could not be agreed, supported with interpretation of meta-analysis, writing up the results, and reviewed the final manuscript.
MA: Supported with development of project and protocol and with the interpretation of findings and reviewed the final manuscript.
LD: Completed data extraction for all studies included and reviewed the final manuscript.
JR: Supported with development of project and protocol and reviewed the final manuscript.
JH: Public contributor assisting with project design, finalising the research questions and identifying outcome measures of importance.
MM: Public contributor assisting with project design, finalising the research questions and identifying outcome measures of importance.
SJCT: Supported with development of project and protocol and reviewed the final manuscript.
KW: Supervised TR through the process, supported with development of project, protocol, and analysis plan and reviewed the final manuscript.

Data Statement
All data is available by reasonable request from the corresponding author.

Funding
This study/project is funded by the National Institute for Health and Care Research (NIHR) School for Primary Care Research (572). The views expressed are those of the author(s) and not necessarily those of the NIHR or the Department of Health and Social Care.

References

1. NICE: National Institute for Health and Care Excellence. https://cks.nice.org.uk/topics/multimorbidity/background-information/prevalence/. 2023. Multimorbidity: How common is it?
2. Kingston A, Robinson L, Booth H, Knapp M, Jagger C, project M. Projections of multi-morbidity in the older population in England to 2035: estimates from the Population Ageing and Care Simulation (PACSim) model. Age Ageing. 2018;47(3):374-80.
3. Nunes BP, Flores TR, Mielke GI, Thume E, Facchini LA. Multimorbidity and mortality in older adults: A systematic review and meta-analysis. Arch Gerontol Geriatr. 2016;67:130-8.

For peer review only

4. Williams JS, Egede LE. The Association Between Multimorbidity and Quality of Life, Health Status and Functional Disability. Am J Med Sci. 2016;352(1):45-52.
5. Soley-Bori M, Ashworth M, Bisquera A, Dodhia H, Lynch R, Wang Y, et al. Impact of multimorbidity on healthcare costs and utilisation: a systematic review of the UK literature. Br J Gen Pract. 2021;71(702):e39-e46.
6. Afshar S, Roderick PJ, Kowal P, Dimitrov BD, Hill AG. Multimorbidity and the inequalities of global ageing: a cross-sectional study of 28 countries using the World Health Surveys. BMC Public Health. 2015;15:776.
7. Salisbury C, Johnson L, Purdy S, Valderas JM, Montgomery AA. Epidemiology and impact of multimorbidity in primary care: a retrospective cohort study. Br J Gen Pract. 2011;61(582):e12-21.
8. Skivington K, Matthews L, Simpson SA, Craig P, Baird J, Blazeby JM, et al. A new framework for developing and evaluating complex interventions: update of Medical Research Council guidance. BMJ. 2021;374:n2061.
9. Harrison M, Reeves D, Harkness E, Valderas J, Kennedy A, Rogers A, et al. A secondary analysis of the moderating effects of depression and multimorbidity on the effectiveness of a chronic disease self-management programme. Patient Educ Couns. 2012;87(1):67-73.
10. Zora S, Custodero C, Pers YM, Valsecchi V, Cella A, Ferri A, et al. Impact of the chronic disease self-management program (CDSMP) on self-perceived frailty condition: the EU-EFFICHRONIC project. Ther Adv Chronic Dis. 2021;12:20406223211056722.
11. Smith SM, Soubhi H, Fortin M, Hudon C, O'Dowd T. Managing patients with multimorbidity: systematic review of interventions in primary care and community settings. BMJ. 2012;345:e5205.
12. Smith SM, Wallace E, O'Dowd T, Fortin M. Interventions for improving outcomes in patients with multimorbidity in primary care and community settings. Cochrane Database Syst Rev. 2016;3(3):CD006560.
13. Bratzke LC, Muehrer RJ, Kehl KA, Lee KS, Ward EC, Kwekkeboom KL. Self-management priority setting and decision-making in adults with multimorbidity: a narrative review of literature. Int J Nurs Stud. 2015;52(3):744-55.
14. Smith SM, Wallace E, Clyne B, Boland F, Fortin M. Interventions for improving outcomes in patients with multimorbidity in primary care and community setting: a systematic review. Syst Rev. 2021;10(1):271.
15. Ouzzani M, Hammady H, Fedorowicz Z, Elmagarmid A. Rayyan-a web and mobile app for systematic reviews. Syst Rev. 2016;5(1):210.
16. Sterne JA-O, Savović J, Page MJ, Elbers RG, Blencowe NS, Boutron I, et al. RoB 2: a revised tool for assessing risk of bias in randomised trials. (1756-1833 (Electronic)).
17. Review Manager Web (RevMan Web). Available at revman.cochrane.org: The Cochrane Collaboration; 2020.
18. Higgins JP, Thompson Sg Fau - Deeks JJ, Deeks Jj Fau - Altman DG, Altman DG. Measuring inconsistency in meta-analyses. (1756-1833 (Electronic)).
19. Page MJ, McKenzie JE, Bossuyt PM, Boutron I, Hoffmann TC, Mulrow CD, et al. The PRISMA 2020 statement: an updated guideline for reporting systematic reviews. BMJ. 2021;372.
20. Heath G, Cooke R, Cameron E. A Theory-Based Approach for Developing Interventions to Change Patient Behaviours: A Medication Adherence Example from Paediatric Secondary Care. Healthcare (Basel). 2015;3(4):1228-42.
21. Michie S, van Stralen MM, West R. The behaviour change wheel: a new method for characterising and designing behaviour change interventions. Implement Sci. 2011;6:42.
22. Oades LG, Ozturk C, Hou H, Slemp GR. Wellbeing literacy: A language-use capability relevant to wellbeing outcomes of positive psychology intervention. The Journal of Positive Psychology. 2020;15(5):696-700.
23. Huguet A, Rao S, McGrath PJ, Wozney L, Wheaton M, Conrod J, et al. A Systematic Review of Cognitive Behavioral Therapy and Behavioral Activation Apps for Depression. PLoS One. 2016;11(5):e0154248.

For peer review only

24. Orgeta V, Brede J, Livingston G. Behavioural activation for depression in older people: systematic review and meta-analysis. Br J Psychiatry. 2017;211(5):274-9.
25. What are integrated care systems? Available at https://www.england.nhs.uk/integratedcare/what-is-integrated-care/: NHS England; 2022 [
26. Coventry P, Lovell K, Dickens C, Bower P, Chew-Graham C, McElvenny D, et al. Integrated primary care for patients with mental and physical multimorbidity: cluster randomised controlled trial of collaborative care for patients with depression comorbid with diabetes or cardiovascular disease. BMJ. 2015;350:h638.
27. Kappelin C, Carlsson AC, Wachtler C. Specific content for collaborative care: a systematic review of collaborative care interventions for patients with multimorbidity involving depression and/or anxiety in primary care. Fam Pract. 2022;39(4):725-34.
28. Hoffmann TC, Glasziou PP, Boutron I, Milne R, Perera R, Moher D, et al. Better reporting of interventions: template for intervention description and replication (TIDieR) checklist and guide. BMJ. 2014;348:g1687.
29. Smith SM, Bayliss EA, Mercer SW, Gunn J, Vestergaard M, Wyke S, et al. How to Design and Evaluate Interventions to Improve Outcomes for Patients with Multimorbidity. Journal of Comorbidity. 2013;3(1):10-7.

For peer review only

Example Medline (OVID) Search Strategy for Systematic Review

Multiple long-term conditions search strategy

Adapted from Smith et al. (2021)

1 Comorbidity/

2 (comorbid$ or co-morbid$).ti,ab.

3 (multimorbid$ or multi-morbid$).ti,ab.

4 (multidisease? or multi-disease? or (multiple adj (ill$ or disease? or condition? or syndrom$ or disorder?))).ti,ab.

5 Long term condition.ti,ab.

6 Chronic disease/

7 (multiple chronic$ adj3 (disease? or ill$ or care or condition? or disorder$ or health$ or syndrom$ or symptom$)).ti,ab.

8 ((coocur$ or co-ocur$ or coexist$ or co-exist$ or multipl$) adj3 (disease? or ill$ or care or condition? or disorder$ or health$ or medication$ or symptom$ or syndrom$)).ti,ab.

9 or/1-8

10 exp diabetes mellitus/ or diabet$.ti,ab.

11 exp hypertension/ or (hypertens$ or "high blood pressure?").ti,ab.

12 exp heart diseases/ or (((heart or cardiac or cardiovascular or coronary) adj (disease? or disorder? or failure)) or arrythmia?).ti,ab.

13 exp cerebrovascular disorders/ or ((cerebrovascular or vascular or carotoid$ or arter$) adj (disorder? or disease?)).ti,ab.

14 exp asthma/ or asthma$.ti,ab.

15 exp pulmonary disease chronic obstructive/ or (copd or (pulmonary adj2 (disease? or disorder?))).ti,ab.

16 exp hyperlipidemia/ or (hyperlipidem$ or Hypercholesterolemia$ or hypertriglyceridemia$).ti,ab.

17 exp Thyroid diseases/ or ((thyroid adj (disease? or disorder)) or hyperthyroid$ or hypothyroid$).ti,ab.

18 exp arthritis rheumatoid/ or rheumatoid arthritis.ti,ab.

19 exp mental disorders/ or (((mental or anxiety or panic or mood or psychological) adj (disease? or disorder?)) or (depression or bipolar or schizophren$ or psychos$)).ti,ab.

20 exp epilepsy/ or (epileps$ or seizure?).ti,ab.

21 exp hiv infections/ or (HIV or acquired immune$ deficiency syndrome? or (aids adj (associated or related or arteritis))).ti,ab.

22 exp neoplasms/ or (neoplasm? or cancer?).ti,ab.

For peer review only

23 exp kidney diseases/ or (kidney adj (disease? or disorder?)).ti,ab.

24 exp liver diseases/ or (liver adj (disease? or disorder?)).ti,ab.

25 exp osteoporosis/ or osteoporosis.ti,ab.

26 exp dementia/ or dementia.ab,ti.

27 exp stroke/ or stroke.ab,ti.

28 exp multiple sclerosis/ or (multiple sclerosis).ab,ti.

29 exp Parkinson disease/ or Parkinson*.ab,ti.

30 exp motor neuron disease/ or (motor neuron disease).ab,ti.

31 exp neurodegenerative diseases/ or neurodegenerative.ab,ti.

32 or/10-31

33 9 and 32

Behaviour change intervention search strategy

Adapted from Gardener et al. (2017)

1 (self car*) OR (self-manag*).mp.

2 behavio* adj3 chang*.mp.

3 (health education) OR (structure* education).mp.

4 secondary prevent*.mp.

5 (case manag*) OR (care manag*).mp.

6 (care navigat*) OR (community navigat*) OR (care co-ordinat*).mp.

7 (shared care) OR (collaborative care).mp.

8 (complex intervention*) OR (multicomponent intervention) OR (multiple intervention component).mp.

9 (integrated adj (model OR care)).mp.

10 ((Multiprofessional AND intervention$) OR (Interprofessional AND intervention$)).mp.

11 (comprehensive care) OR (comprehensive health care).mp.

12 (prevent* adj3 hospital*).mp.

13 or/1-12

RCT search strategy

For peer review only

Cochrane RCT from Smith et al. (2021)

1 randomi?ed controlled trial.pt.

2 controlled clinical trial.pt.

3 random$.ti,ab.

4 (control$ adj2 (trial? or study or studies)).ti,ab.

5 double-blind method/ or random allocation/ or single-blind method/

6 ((double or single or triple or treble) adj2 blind$).ti,ab.

7 or/1-6

8 exp animals/ not humans.sh.

9 7 not 8

For peer review only

1. Ali MK, Chwastiak L, Poongothai S, Emmert-Fees KMF, Patel SA, Anjana RM, et al. Effect of a Collaborative Care Model on Depressive Symptoms and Glycated Hemoglobin, Blood Pressure, and Serum Cholesterol among Patients with Depression and Diabetes in India: The INDEPENDENT Randomized Clinical Trial. JAMA - Journal of the American Medical Association. 2020 Aug 18;324(7):651–62.

2. Aragonès E, Rambla C, López-Cortacans G, Tomé-Pires C, Sánchez-Rodríguez E, Caballero A, et al. Effectiveness of a collaborative care intervention for managing major depression and chronic musculoskeletal pain in primary care: A cluster-randomised controlled trial. J Affect Disord. 2019 Jun 1;252:221–9.

3. Barley EA, Walters P, Haddad M, Phillips R, Achilla E, McCrone P, et al. The UPBEAT nurse-delivered personalized care intervention for people with coronary heart disease who report current chest pain and depression: A randomised controlled pilot study. PLoS One. 2014 Jun 5;9(6).

4. Barrowclough C, Haddock G, Tarrier N, Shôn Lewis FbpW, Jan Moring Frcp, Nichola Schofield B, et al. Article Randomized Controlled Trial of Motivational Interviewing, Cognitive Behavior Therapy, and Family Intervention for Patients With Comorbid Schizophrenia and Substance Use Disorders. Vol. 158, Am J Psychiatry. 2001.

5. Barr Taylor C, Houston Miller N, Reilly KR, Greenwald G, Cunning D, Deeter A, et al. Evaluation of a Nurse-Care Management System to Improve Outcomes in Patients With Complicated Diabetes [Internet]. 2003. Available from: http://diabetesjournals.org/care/article-pdf/26/4/1058/659413/dc0403001058.pdf

6. Battersby MW, Beattie J, Pols RG, Smith DP, Condon J, Blunden S. A randomised controlled trial of the Flinders ProgramTM of chronic condition management in Vietnam veterans with co-morbid alcohol misuse, and psychiatric and medical conditions. Australian and New Zealand Journal of Psychiatry. 2013 May;47(5):451–62.

7. Blank MB, Hanrahan NP, Fishbein M, Wu ES, Tennille JA, Ten Have TR, et al. A randomized trial of a nursing intervention for HIV disease management among persons with serious mental illness. Psychiatric Services. 2011 Nov 1;62(11):1318–24.

8. Boeschoten RE, Dekker J, Uitdehaag BMJ, Beekman ATF, Hoogendoorn AW, Collette EH, et al. Internet-based treatment for depression in multiple sclerosis: A randomized controlled trial. Multiple Sclerosis. 2017 Jul 1;23(8):1112–22.

9. Bogner HR, De Vries HF. Integration of depression and hypertension treatment: A pilot, randomized controlled trial. Ann Fam Med. 2008;6(4):295–301.

10. Chwastiak LA, Luongo M, Russo J, Johnson L, Lowe JM, Hoffman G, et al. Use of a mental health center collaborative care team to improve diabetes care and outcomes for patients with psychosis. Psychiatric Services. 2018 Mar 1;69(3):349–52.

11. Cummings DM, Lutes LD, Littlewood K, Solar C, Carraway M, Kirian K, et al. Randomized trial of a tailored cognitive behavioral intervention in type 2 diabetes with comorbid depressive and/or regimen-related distress symptoms: 12-month outcomes from. In: Diabetes Care. American Diabetes Association Inc.; 2019. p. 841–8.

12. Druss BG, Singh M, Von Esenwein SA, Glick GE, Tapscott S, Tucker SJ, et al. Peer-led self-management of general medical conditions for patients with serious mental illnesses: A randomized trial. Psychiatric Services. 2018 May 1;69(5):529–35.

For peer review only

13. Ell K, Katon W, Xie B, Lee PJ, Kapetanovic S, Guterman J, et al. Collaborative care management of major depression among low-income, predominantly hispanic subjects with diabetes: A randomized controlled trial. Diabetes Care. 2010 Apr;33(4):706–13.

14. Ell K, Aranda MP, Wu S, Oh H, Lee PJ, Guterman J. Promotora assisted depression and self-care management among predominantly Latinos with concurrent chronic illness: Safety net care system clinical trial results. Contemp Clin Trials. 2017 Oct 1;61:1–9.

15. Fisher K, Markle-Reid M, Ploeg J, Bartholomew A, Griffith LE, Gafni A, et al. Self-management program versus usual care for community-dwelling older adults with multimorbidity: A pragmatic randomized controlled trial in Ontario, Canada. J Comorb. 2020 Jan 1;10:2235042X2096339.

16. Garvey J, Connolly D, Boland F, Smith SM. OPTIMAL, an occupational therapy led self-management support programme for people with multimorbidity in primary care: A randomized controlled trial. BMC Fam Pract. 2015 Dec 12;16(1).

17. Goldberg RW, Dickerson F, Lucksted A, Brown CH, Weber E, Tenhula WN, et al. Living Well: An intervention to improve self-management of medical illness for individuals with serious mental illness. Psychiatric Services. 2013 Jan 1;64(1):51–7.

18. Goorden M, van der Feltz-Cornelis CM, van Steenbergen-Weijenburg KM, Horn EK, Beekman ATF, Hakkaar-Van Roijen L. Cost-utility of collaborative care for the treatment of comorbid major depressive disorder in outpatients with chronic physical conditions. A randomized controlled trial in the general hospital setting (CC-DIM). Neuropsychiatr Dis Treat. 2017 Jul 18;13:1881–93.

19. Griva K, Rajeswari M, Nandakumar M, Khoo EYH, Lee VYW, Chua CG, et al. The combined diabetes and renal control trial (C-DIRECT) - A feasibility randomised controlled trial to evaluate outcomes in multi-morbid patients with diabetes and on dialysis using a mixed methods approach. BMC Nephrol. 2019 Jan 3;20(1).

20. Guo Y, Lane DA, Wang L, Zhang H, Wang H, Zhang W, et al. Mobile Health Technology to Improve Care for Patients With Atrial Fibrillation. J Am Coll Cardiol. 2020 Apr 7;75(13):1523–34.

21. Hernandez-Quiles C, Bernabeu-Wittel M, Barón-Franco B, Palacios AA, Garcia-Serrano MR, Lopez-Jimeno W, et al. A randomized clinical trial of home telemonitoring in patients with advanced heart and lung diseases. J Telemed Telecare. 2021;

22. House A, Bryant L, Russell AM, Wright-Hughes A, Graham L, Walwyn R, et al. Randomized controlled feasibility trial of supported self-management in adults with Type 2 diabetes mellitus and an intellectual disability: OK Diabetes. Diabetic Medicine. 2018 Jun 1;35(6):776–88.

23. Jackson IL, Ukwe C V. Clinical outcomes of pharmaceutical care intervention in HIV positive patients with hypertension: A randomized controlled study. J Clin Pharm Ther. 2021 Aug 1;46(4):1083–94.

24. Kamradt M, Ose D, Krisam J, Jacke C, Salize HJ, Besier W, et al. Meeting the needs of multimorbid patients with Type 2 diabetes mellitus – A randomized controlled trial to assess the impact of a care management intervention aiming to improve self-care. In: Diabetes Research and Clinical Practice. Elsevier Ireland Ltd; 2019. p. 184–93.

25. Kanwal F, Pyne JM, Tavakoli-Tabasi S, Nicholson S, Dieckgraefe B, Storay E, et al. A Randomized Trial of Off-Site Collaborative Care for Depression in Chronic Hepatitis C Virus. Health Serv Res. 2018 Aug 1;53(4):2547–66.

For peer review only

26. Katon WJ, Michael ;, Korff V, Lin EHB, Simon G, Ludman E, et al. The Pathways Study A Randomized Trial of Collaborative Care in Patients With Diabetes and Depression. 2004.

27. Katon WJ, Lin EH, Von Korff M, Ciechanowski P, Ludman EJ, Young B, et al. Collaborative Care for Patients with Depression and Chronic Illnesses From the Departments of Psychiatry A bs t r ac t. Vol. 363, N Engl J Med. 2010.

28. Khunti K, Highton PJ, Waheed G, Dallosso H, Redman E, Batt ME, et al. Promoting physical activity with self-management support for those with multimorbidity: A randomised controlled trial. British Journal of General Practice. 2021 Dec 1;71(713):E921–30.

29. Koesoemadinata RC, McAllister SM, Soetedjo NNM, Santoso P, Ruslami R, Damayanti H, et al. Educational counselling of patients with combined TB and diabetes mellitus: a randomised trial. Public Health Action. 2021 Dec 18;11(4):202–8.

30. Kroenke K, Bair MJ, Damush TM, Wu J, Hoke S, Sutherland J, et al. Optimized Antidepressant Therapy and Pain Self-management in Primary Care Patients With Depression and Musculoskeletal Pain A Randomized Controlled Trial [Internet]. 2009. Available from: https://jamanetwork.com/

31. Lear SA, Norena M, Banner D, Whitehurst DGT, Gill S, Burns J, et al. Assessment of an Interactive Digital Health-Based Self-management Program to Reduce Hospitalizations among Patients with Multiple Chronic Diseases: A Randomized Clinical Trial. JAMA Netw Open. 2021 Dec 28;4(12).

32. Lenferink A, van der Palen J, van der Valk PDLPM, Cafarella P, van Veen A, Quinn S, et al. Exacerbation action plans for patients with COPD and comorbidities: A randomised controlled trial. European Respiratory Journal. 2019 Nov 1;54(5).

33. Markle-Reid M, Ploeg J, Fraser KD, Fisher KA, Bartholomew A, Griffith LE, et al. Community Program Improves Quality of Life and Self-Management in Older Adults with Diabetes Mellitus and Comorbidity. J Am Geriatr Soc. 2018 Feb 1;66(2):263–73.

34. McDermott RA, Schmidt B, Preece C, Owens V, Taylor S, Li M, et al. Community health workers improve diabetes care in remote Australian indigenous communities: Results of a pragmatic cluster randomized controlled trial. BMC Health Serv Res. 2015 Feb 19;15(1).

35. Merlin JS, Westfall AO, Long D, Davies S, Saag M, Demonte W, et al. A Randomized Pilot Trial of a Novel Behavioral Intervention for Chronic Pain Tailored to Individuals with HIV. AIDS Behav. 2018 Aug 1;22(8):2733–42.

36. Miklavcic JJ, Fraser KD, Ploeg J, Markle-Reid M, Fisher K, Gafni A, et al. Effectiveness of a community program for older adults with type 2 diabetes and multimorbidity: A pragmatic randomized controlled trial. BMC Geriatr. 2020 May 13;20(1).

37. Morgan MAJ, Coates MJ, Dunbar JA, Reddy P, Schlicht K, Fuller J. The TrueBlue model of collaborative care using practice nurses as case managers for depression alongside diabetes or heart disease: a randomised trial. BMJ Open [Internet]. 2013; Available from: http://bmjopen.bmj.com/

38. Naik AD, Hundt NE, Vaughan EM, Petersen NJ, Zeno D, Kunik ME, et al. Effect of Telephone-Delivered Collaborative Goal Setting and Behavioral Activation vs Enhanced Usual Care for Depression among Adults with Uncontrolled Diabetes: A Randomized Clinical Trial. JAMA Netw Open. 2019 Aug 7;2(8).

For peer review only

39. Ose D, Kamradt M, Kiel M, Freund T, Besier W, Mayer M, et al. Care management intervention to strengthen self-care of multimorbid patients with type 2 diabetes in a German primary care network: A randomized controlled trial. PLoS One. 2019 Jun 1;14(6).

40. O'Toole L, Connolly D, Boland F, Smith SM. Effect of the OPTIMAL programme on self-management of multimorbidity in primary care: A randomised controlled trial. British Journal of General Practice. 2021 Apr 1;71(705):E303–11.

41. Park YH, Chang HK. Effect of a health coaching self-management program for older adults with multimorbidity in nursing homes. Patient Prefer Adherence. 2014 Jul 4;8:959–70.

42. Pibernik-Okanovic M, Begic D, Ajdukovic D, Andrijasevic N, Metelko Z. Psychoeducation versus treatment as usual in diabetic patients with subthreshold depression: Preliminary results of a randomized controlled trial. Trials. 2009 Aug 26;10.

43. Rose L, Istanboulian L, Carriere L, Thomas A, Lee HB, Rezaie S, et al. Program of integrated care for patients with chronic obstructive pulmonary disease and multiple comorbidities (PIC COPD+): A randomised controlled trial. European Respiratory Journal. 2018;51(1).

44. Ross CJM, Davis TMA, MacDonald GF. Cognitive-behavioral treatment combined with asthma education for adults with asthma and coexisting panic disorder. Clin Nurs Res. 2005 May;14(2):131–57.

45. Sajatovic M, Gunzler DD, Kanuch SW, Cassidy KA, Tatsuoka C, McCormick R, et al. A 60-week prospective RCT of a self-management intervention for individuals with serious mental illness and diabetes mellitus. Psychiatric Services. 2017 Sep 1;68(9):883–90.

46. Salisbury C, Man MS, Bower P, Guthrie B, Chaplin K, Gaunt DM, et al. Management of multimorbidity using a patient-centred care model: a pragmatic cluster-randomised trial of the 3D approach. The Lancet. 2018 Jul 7;392(10141):41–50.

47. Schneider KL, Panza E, Handschin B, Ma Y, Busch AM, Waring ME, et al. Feasibility of Pairing Behavioral Activation With Exercise for Women With Type 2 Diabetes and Depression: The Get It Study Pilot Randomized Controlled Trial [Internet]. 2015. Available from: www.elsevier.com/locate/bt

48. Takahashi PY, Pecina JL, Upatising B, Chaudhry R, Shah ND, Van Houten H, et al. A randomized controlled trial of telemonitoring in older adults with multiple health issues to prevent hospitalizations and emergency department visits. Arch Intern Med. 2012 May 28;172(10):773–9.

49. Vera M, Perez-Pedrogo C, Huertas SE, Reyes-Rabanillo ML, Juarbe D, Huertas A, et al. Collaborative Care for Depressed Patients With Chronic Medical Conditions: A Randomized Trial in Puerto Rico. Psychiatric Services. 2010 Feb 1;61(2).

50. Wakefield BJ, Holman JE, Ray A, Scherubel M, Adams MR, Hillis SL, et al. Effectiveness of home telehealth in comorbid diabetes and hypertension: A randomized, controlled trial. Telemedicine and e-Health. 2011 May 1;17(4):254–61.

51. Williams A, Manias E, Walker R, Gorelik A. A multifactorial intervention to improve blood pressure control in co-existing diabetes and kidney disease: A feasibility randomized controlled trial. J Adv Nurs. 2012 Nov;68(11):2515–25.

For peer review only

52. Wilson M, Hewes C, Barbosa-Leiker C, Mason A, Wuestney KA, Shuen JA, et al. Engaging Adults With Chronic Disease in Online Depressive Symptom Self-Management. West J Nurs Res. 2018 Jun 1;40(6):834–53.

53. Yu C, Choi D, Bruno BA, Thorpe KE, Straus SE, Cantarutti P, et al. Impact of mydiabetesplan, a web-based patient decision aid on decisional conflict, diabetes distress, quality of life, and chronic illness care in patients with diabetes: Cluster randomized controlled trial. J Med Internet Res. 2020 Sep 29;22(9).

For peer review only

PRISMA 2020 Checklist

Section and Topic	Item #	Checklist item	Location where item is reported
**TITLE**			
Title	1	Identify the report as a systematic review.	Page 1
**ABSTRACT**			
Abstract	2	See the PRISMA 2020 for Abstracts checklist.	Page 1
**INTRODUCTION**			
Rationale	3	Describe the rationale for the review in the context of existing knowledge.	Page 1 and 2
Objectives	4	Provide an explicit statement of the objective(s) or question(s) the review addresses.	Page 2
**METHODS**			
Eligibility criteria	5	Specify the inclusion and exclusion criteria for the review and how studies were grouped for the syntheses.	Page 3
Information sources	6	Specify all databases, registers, websites, organisations, reference lists and other sources searched or consulted to identify studies. Specify the date when each source was last searched or consulted.	Page 2 and 3
Search strategy	7	Present the full search strategies for all databases, registers and websites, including any filters and limits used.	Supplementary figure 1
Selection process	8	Specify the methods used to decide whether a study met the inclusion criteria of the review, including how many reviewers screened each record and each report retrieved, whether they worked independently, and if applicable, details of automation tools used in the process.	Page 2 and 3
Data collection process	9	Specify the methods used to collect data from reports, including how many reviewers collected data from each report, whether they worked independently, any processes for obtaining or confirming data from study investigators, and if applicable, details of automation tools used in the process.	Page 3
Data items	10a	List and define all outcomes for which data were sought. Specify whether all results that were compatible with each outcome domain in each study were sought (e.g. for all measures, time points, analyses), and if not, the methods used to decide which results to collect.	Page 3
	10b	List and define all other variables for which data were sought (e.g. participant and intervention characteristics, funding sources). Describe any assumptions made about any missing or unclear information.	Page 3
Study risk of bias assessment	11	Specify the methods used to assess risk of bias in the included studies, including details of the tool(s) used, how many reviewers assessed each study and whether they worked independently, and if applicable, details of automation tools used in the process.	Page 3
Effect measures	12	Specify for each outcome the effect measure(s) (e.g. risk ratio, mean difference) used in the synthesis or presentation of results.	Page 3
Synthesis methods	13a	Describe the processes used to decide which studies were eligible for each synthesis (e.g. tabulating the study intervention characteristics and comparing against the planned groups for each synthesis (item #5)).	Page 3
	13b	Describe any methods required to prepare the data for presentation or synthesis, such as handling of missing summary statistics, or data conversions.	Page 3
	13c	Describe any methods used to tabulate or visually display results of individual studies and syntheses.	Supplementary table 2
	13d	Describe any methods used to synthesize results and provide a rationale for the choice(s). If meta-analysis was performed, describe the model(s), method(s) to identify the presence and extent of statistical heterogeneity, and software package(s) used.	Page 3
	13e	Describe any methods used to explore possible causes of heterogeneity among study results (e.g. subgroup analysis, meta-regression).	Page 3
	13f	Describe any sensitivity analyses conducted to assess robustness of the synthesized results.	N/A
Reporting bias assessment	14	Describe any methods used to assess risk of bias due to missing results in a synthesis (arising from reporting biases).	Page 3

For peer review only

For peer review only - http://bmjopen.bmj.com/site/about/guidelines.xhtml

PRISMA 2020 Checklist

Section and Topic	Item #	Checklist item	Location where item is reported
Certainty assessment	15	Describe any methods used to assess certainty (or confidence) in the body of evidence for an outcome.	Page 10
**RESULTS**			
Study selection	16a	Describe the results of the search and selection process, from the number of records identified in the search to the number of studies included in the review, ideally using a flow diagram.	Page 4
	16b	Cite studies that might appear to meet the inclusion criteria, but which were excluded, and explain why they were excluded.	Page 4
Study characteristics	17	Cite each included study and present its characteristics.	Supplementary figure 2 and supplementary table 2
Risk of bias in studies	18	Present assessments of risk of bias for each included study.	Supplementary table 2
Results of individual studies	19	For all outcomes, present, for each study: (a) summary statistics for each group (where appropriate) and (b) an effect estimate and its precision (e.g. confidence/credible interval), ideally using structured tables or plots.	Tables 2, 3, 4 and supplementary table 4
Results of syntheses	20a	For each synthesis, briefly summarise the characteristics and risk of bias among contributing studies.	Page 5
	20b	Present results of all statistical syntheses conducted. If meta-analysis was done, present for each the summary estimate and its precision (e.g. confidence/credible interval) and measures of statistical heterogeneity. If comparing groups, describe the direction of the effect.	Tables 2, 3, 4 and supplementary table 4
	20c	Present results of all investigations of possible causes of heterogeneity among study results.	Page 7, 8, 9
	20d	Present results of all sensitivity analyses conducted to assess the robustness of the synthesized results.	N/A
Reporting biases	21	Present assessments of risk of bias due to missing results (arising from reporting biases) for each synthesis assessed.	Page 7, 8, 9
Certainty of evidence	22	Present assessments of certainty (or confidence) in the body of evidence for each outcome assessed.	Page 10
**DISCUSSION**			
Discussion	23a	Provide a general interpretation of the results in the context of other evidence.	Page 9 and 10
	23b	Discuss any limitations of the evidence included in the review.	Page 10
	23c	Discuss any limitations of the review processes used.	Page 10
	23d	Discuss implications of the results for practice, policy, and future research.	Page 10
**OTHER INFORMATION**			
Registration and protocol	24a	Provide registration information for the review, including register name and registration number, or state that the review was not registered.	Page 2
	24b	Indicate where the review protocol can be accessed, or state that a protocol was not prepared.	Page 2
	24c	Describe and explain any amendments to information provided at registration or in the protocol.	N/A
Support	25	Describe sources of financial or non-financial support for the review, and the role of the funders or sponsors in the review.	Page 11

For peer review only

PRISMA 2020 Checklist

Section and Topic	Item #	Checklist item	Location where item is reported
Competing interests	26	Declare any competing interests of review authors.	Page 11
Availability of data, code and other materials	27	Report which of the following are publicly available and where they can be found: template data collection forms; data extracted from included studies; data used for all analyses; analytic code; any other materials used in the review.	Page 11

From: Page MJ, McKenzie JE, Bossuyt PM, Boutron I, Hoffmann TC, Mulrow CD, et al. The PRISMA 2020 statement: an updated guideline for reporting systematic reviews. BMJ 2021;372:n71. doi: 10.1136/bmj.n71

For more information, visit: http://www.prisma-statement.org/

For peer review only

Lead Author<br>Year<br>Country	Participant condition 1<br>Participant condition 2<br>Other conditions	LTC combination	Study n<br>Intervention n<br>Control n<br>Carers n	Intervention length<br>Follow-up timepoints	Primary outcome<br>Timepoint<br>Effectiveness
Ali<br>2020<br>India	Depression<br>Type 2 Diabetes	Physical-Mental	404<br>196<br>208	12 months<br>Baseline, 6, 12, 18, 24 months	Composite depression and clinical<br>24 months<br>Yes
Aragones<br>2019<br>Spain	Depression and Chronic Musculoskeletal Pain	Physical-Mental	41<br>21<br>20	3 months<br>Baseline, 3, 6, 12 months.	Depression<br>12 months<br>Yes
Barley, 2014, UK	Coronary heart disease<br>Depression	Physical-Mental	81<br>41<br>40	6 months.<br>Baseline, 1, 3, 6, 12 months.	N/A Feasibility and acceptability
Barrowclough, 2001, UK	Schizophrenia<br>Substance Abuse disorder	Mental-Mental	33 patient carer dyads	9 months.<br>Baseline, 9 months and 12 months.	Functioning<br>9 and 12 months<br>Yes

For peer review only

Barr Taylor, 2003, USA	Diabetes<br>And one or more of:<br>Hypertension<br>CVD<br>Dyslipidemia	Physical-Physical	169<br>84<br>85	12 months<br>Baseline, 12 months	Clincal outcome (HbA1c)<br>12 months<br>Yes
Battersby, 2013, Australia	Alcohol use disorder<br>AND one or more of:<br>-Cardiovascular<br>-Musculoskeletal<br>-Gastrointestinal<br>-Respiratory<br>-Diabetes<br>-Skin conditions<br>-Cancer<br>-Genitourinary<br>-Alcohol dependence<br>-PTSD<br>-Major depression<br>-Generalised Anxiety Disorder<br>-Panic Disorder	Physical-Mental	77<br>46<br>31<br>SCDSMP course = 22 veterans and 15 of their partners from intervention group	9-month intervention. Seen at 1, 3, 6 and 9 months, with telephone calls at 4.5 and 7.5 months<br>Follow-up measures at 18 months	Behaviour change (AUDIT)<br>9 months<br>Yes
Blank 2011, USA	HIV<br>Schizophrenia Spectrum Disorder/ Affective Disorder/ Other serious mental ilness	Physical-Mental	238<br>128<br>110	12 months. Baseline, 3 months, 6 months, 12 months<br>Follow-up at 24 months.	Clinical (Viral load)<br>12 months<br>No
Boeschoten, 2017, The Netherlands	MS<br>Depression<br>Anxiety	Physical-Mental	171<br>85<br>86	5 to 10 weeks.<br>Baseline, within a week of intervention, 4 months	Depression (BDI-II)<br>5-10 weeks and 4 months<br>No

For peer review only

Bogner, 2008, USA	Hypertension<br>Depression	Physical-Mental	64<br>32<br>32	4 weeks.<br>Baseline, 2, 4 and 6 weeks.	Depression (CES-D)<br>6 weeks<br>Yes
Chwastiak, 2018, USA	Type II diabetes<br>Schizophrenia<br>Schizoaffective disorder<br>Bipolar disorder<br>Major depressive disorder with psychosis	Physical-Mental	35<br>18<br>17	3 months.<br>Baseline, 3 months.	Clinical (HbA1c)<br>3 months<br>N/A test not completed
Cummings, 2019, USA	Type 2 diabetes<br>Diabetes-related distress or depression	Physical-Mental	139<br>67 (CBT=48; Lifestyle=19)<br>72	12 months.<br>Baseline, 6 months (to adjust intervention if necessary), 12 months.	Clinical (HbA1c)<br>12 months<br>No (approached p=0.06)

For peer review only

Druss, 2018, USA	Medical illness<br>Diabetes<br>Heart disease<br>Hyperlipidemia<br>Hypertension<br>Arthritis<br>Hepatitis<br>Asthma/COPD<br>HIV<br>SMI<br>Schizophrenia/ schizoaffective<br>Bipolar disorder<br>Depression<br>OCD<br>PTSD	Physical-Mental	400<br>198<br>202	3 months. Baseline, 3 months, 6 months.	Quality of life<br>6 months<br>Yes
Ell, 2010, USA	Type 1 or 2 diabetes<br>Depression	Physical-Mental	387<br>193<br>194	12 months. Baseline, 6, 12, and 18 months.	Depression (SLC-20)<br>6-12-18 months<br>Yes
Ell, 2017, USA	Depression<br>Diabetes OR heart disease OR both	Physical-Mental	348<br>178<br>170	6 weeks (plus 3 monthly boosters). Baseline, 6 months, 12 months.	Depression (PHQ-9)<br>12 months<br>No

For peer review only

Fisher, 2020, Canada	At least 3 chronic conditions<br>Most common:<br>Cardiovascular<br>Kidney/Urogenital<br>Arthritis<br>Gastrointestinal<br>Endocrine<br>Hearing/Vision	Physical-Physical	59<br>30<br>29	6 months.<br>Baseline and 6 months.	SF-12 Physical<br>6 months<br>No
Garvey, 2015, Ireland	43 chronic coniditions identified, most common:<br>Arthritis<br>Congestive cardiac failure<br>Diabetes<br>Depression<br>Hypertension	Physical-Physical	50<br>26<br>24	6 weeks.<br>Baseline and 6 weeks	N/A (participation)<br>6 weeks<br>yes
Goldberg, 2013, USA	Schizophrenia spectrum disorder OR bipolar disorder<br>AND one or more chronic condition, including:<br>Diabetes<br>Arthritis<br>Respiratory disease<br>Cardiovascular disease	Physical-Mental	63<br>32<br>31	3 months<br>Baseline, 3 months and 5 months	SF-12 Physical<br>3 months<br>Yes

For peer review only

Goorden, 2017, The Netherlands	Major depressive disorder<br>AND one or more chronic physical condition:<br>Diabetes<br>COPD<br>IBS<br>Heart failure	Physical-Mental	81<br>42<br>39	12 months. Baseline, 3, 6, 9, 12 months.	N/A cost effectiveness<br>12 months
Griva, 2019, Singapore	Diabetes<br>End stage renal disease	Physical-Mental	44<br>20<br>24	3 weeks. Baseline, 3 months	Clinical endpoint (HbA1c)<br>3 months<br>No
Guo, 2020, China	Atrial Fibrilliation<br>AND<br>Congestive heart failure<br>Hypertension<br>Diabetes<br>Prior ischemic stroke<br>Vascular disease<br>Coronary artery disease<br>Peripheral arterial disease<br>Renal dysfunction<br>Liver dysfunction<br>Prior bleeding<br>Prior brain bleeding<br>Hyperthyroidism<br>Cardiomyopathy	Physical-Physical	40 hospitals randomised (20 to each arm)<br>3324<br>1646<br>1678	12 months Baseline, 6 and 12 months.	N/A<br>composite hospital<br>12 months<br>Yes

For peer review only

Hernandez-Quiles, 2021, Spain	Advanced heart/lung failure/both.<br>AND one of:<br>Hypertension<br>Heart Failure<br>Atrial fibrillation<br>Dyslipemia<br>Diabetes<br>CHD<br>COPD<br>Chronic kidney disease<br>Obesity<br>Chronic anemia<br>Valvular heart disease<br>Pulmonary hypertension<br>Sleep apnea hypopnea syndrome<br>Hypothyroidism<br>Depression	Physical-Physical	510<br>255<br>255	180 days<br>0, 15, 45, 90, 180 days.	N/A hospital admissions<br>45, 90 and 180 days<br>Yes
House, 2018, UK	Type 2 diabetes<br>Obesity<br>Intellectual disability	Physical-Physical	82<br>41<br>41	4 months<br>Baseline, 6 month follow-up (reduced to 4 because of project deadlines)	Clinical endpoint (HbA1c)<br>4-6 months<br>No

For peer review only

Jackson, 2021, Nigeria	HIV<br>Hypertension<br>Other:<br>Diabetes<br>Peptic ulcer disease<br>Renal disease<br>Hypertensive heart disease<br>Osetoarthritis<br>Haemorrhoids<br>Benign prostatic hyperplasia	Physical-Physical	206<br>103<br>103	12 months. Baseline, 6 months, 12 months.	Clinical outcomes (Blood pressure)<br>12 months<br>Yes
Kamradt, 2019, Germany	Type 2 diabetes and Two or more of the following:<br>Coronary heart disease<br>Chronic pain<br>Depression<br>Chronic heart failure<br>COPD<br>Artherosclerosis<br>Cerebrovascular disease	Physical-Physical	495<br>252<br>243	9 months. Baseline, 9 months.	Quality of life<br>9 months<br>No
Kanwal, 2018, USA	HCV<br>Depression	Physical-Mental	309<br>156<br>153	6 months Baseline, 6 months, 12 months.	Depression (SLC-20)<br>12 months<br>Yes

For peer review only

Katon, 2004, USA	Diabetes<br>Depression	Physical-Mental	329<br>164<br>165	3-6 months. Baseline, 3 months, 6 months, 12 months.	Depression (SLC-90)<br>6 and 12 months<br>Yes
Katon, 2010, USA	Depression<br>Diabetes/Coronary heart disease/both	Physical-Mental	214<br>106<br>108	12 months. Baseline, 6 months, 12 months.	Composite depression and clinical<br>12 months<br>Yes
Khunti, 2021, UK	Conditions accepted in inclusion criteria:<br>Cardiovsacular<br>Asthma/COPD<br>High dependency/ LTC's<br>Depression<br>Musculoskeletal	Physical-Physical	353<br>180<br>173	6 months. Baseline, 6 months, 12 months.	Behaviour change (physical activity)<br>12 months<br>Yes

For peer review only

Koesoemadinata, 2021, Indonesia	Tuberculosis<br>Diabetes	Physical-Physical	108<br>60<br>48	6 months. Baseline, 6 months.	N/A patient knowledge<br>6 months<br>No
Kroenke, 2009, USA	Musculoskeletal pain<br>Depression	Physical-Mental	250<br>123<br>127	12 months. Baseline, 1, 3, 6, and 12 months.	Depression (SLC-20)<br>12 months<br>Yes
Lear, 2021, Canada	2 or more of:<br>Diabetes<br>Heart failure<br>Ischemic heart disease<br>Chronic kidney disease<br>COPD	Physical-Physical	230 (219 - one withdrew after randomisation)<br>117 (116 - see above)<br>113	Two years. Baseline, 12 months, 24 months.	N/A Hospital admissions<br>24 months<br>No

For peer review only

Lenferink, 2019, The Netherlands and Australia	COPD<br>and one or more of:<br>Ishemic heart disease<br>Chronic heart failure<br>Diabetes<br>Anxiety<br>Depression	Physical-Physical	201<br>102<br>99	4 weeks intensive and 3 follow up calls at weeks 8, 20 and 36<br>Baseline, 6 months, 12 months.	COPD exacerbation<br>12 months<br>No
Markle-Reid, 2018, Canada	Type 2 diabetes<br>Top 3 most common chronic conditions:<br>Hypertension<br>Cardiovascular<br>Arthritis	Physical-Physical	159<br>80<br>79	6 months.<br>Baseline, 6 months.	SF-12 physical and mental<br>6 months<br>Mental - yes<br>Physical - no
McDermott, 2015, Australia	Type 2 diabetes<br>One or more other significant comorbidity	Physical-Physical	213<br>100<br>113	18 months.<br>Baseline, 18 months.	Clinical (HbA1c)<br>18 months<br>yes

For peer review only

Merlin, 2018, USA	HIV<br>Chronic pain	Physical-Physical	44<br>22<br>22	12 weeks.<br>Baseline, 12 weeks	Pain (BPI)<br>12 weeks<br>no
Miklavcic, 2020, Canada	Type 2 diabetes<br>Two or more other chronic conditions	Physical-Physical	132<br>70<br>62	6 months.<br>Baseline, 6 months.	Physical functioning<br>6 months<br>No
Morgan, 2013, Australia	Depression<br>Type 2 diabetes/ coronary heart disease (or both)	Physical-Mental	400<br>206<br>194	6 months.<br>Baseline, 6 months, 12 months	Depression (PHQ-9)<br>6 months<br>Yes

For peer review only

Naik, 2019, USA	Diabetes<br>Depression	Physical-Mental	225<br>136<br>89	6 months<br>Baseline, 6 months, 12 months.	Depression (PHQ-9) and clinical (HbA1c)<br>6 and 12 months<br>No
Ose, 2019, USA	Type 2 diabetes<br>Two or more other chronic conditions including:<br>Artherosclerosis<br>Coronary heart disease<br>Chronic obstructive lung disease<br>Asthma<br>Cerebrovascular diseases<br>Depression<br>Heart failure<br>Parkinsons<br>Chronic pain	Physical-Physical	495<br>252<br>243	9 months<br>Baseline, 9 months.	Self-care (Summary of Diabetes Self-Care Activities Measure (SDSCA-G)<br>9 months<br>No
O'Toole, 2021, Ireland	Conditions not listed. Mean 4.5 per participant	Physical-Physical	149<br>78<br>71	6 weeks.<br>Baseline, 6 weeks, 6 months.	Quality of life (EQ-5D-3L) and behaviour change (participation)<br>6 weeks<br>No

For peer review only

Park, 2014, South Korea	Two or more of:<br>Stroke<br>Parkinsons<br>Dementia	Physical-Physical	43<br>21<br>22	8 weeks. Baseline, 8 weeks	self-management behaviors, self-efficacy, and health status<br>8 weeks<br>Yes
Pibernik-Okanovic, 2009, Croatia	Diabetes<br>Depression	Physical-Mental	50<br>25<br>25	5 weeks<br>Baseline, 6 months, 12 months	Depression (CES-D) and clinical (HbA1c)<br>6 and 12 months<br>No
Rose, 2018, Canada	COPD and two or more of:<br>Cardiovascular disease<br>Depression<br>Diabetes<br>Osteopenia and osteoporosis<br>Gastro-oesophageal reflux disease<br>Hypothyroidism<br>Osteoarthritis<br>Glaucoma and cataracts<br>Cachexia and malnutrition<br>Chrornic kidney disease<br>Anxiety<br>Peripheral muscle dysfunction<br>Obstructive sleep apnoea<br>Lung cancer<br>Cerebrovascular accident	Physical-Physical	470<br>236<br>234	12 months. Baseline, 3 months, 6 months, and 12 months.	Hospital admissions<br>12 months<br>No

For peer review only

Ross, 2005, Canada	Asthma<br>Panic disorder	Physical-Mental	48 (25 analysed)<br>25 (15 analsyed)<br>23 (10 analysed)	8 weeks.<br>Intervention group: Baseline, 8 weeks, and 6 month follow-up.<br>Wait-list control group: baseline (coincided with IG pre), 8 weeks (coincided with IG post), 16 weeks, and 6 month follow-up	Panic disorder and asthma outcomes<br>8 weeks<br>Yes
Sajatovic, 2017, USA	Serious mental illlness<br>Type 2 diabetes	Physical-Mental	200<br>100<br>100	60 weeks.<br>Baseline, 13 weeks, 30 weeks, 60 weeks	Psychiatric symptoms<br>60 weeks<br>yes
Salisbury, 2018, UK	Cardiovascular disease or chronic kidney disease<br>Stroke or transient ischaemic attack<br>Diabetes<br>COPD or asthma<br>Epilepsy<br>Atrial fibrillation<br>Serious mental illness<br>Depression<br>Dementia<br>Learning disability<br>Rheumatoid arthritis	Physical-Physical	33 practices randomised (17 to intervention, 16 to control).<br><br>1546<br>797<br>749	6 months.<br>Baseline, 9 months, 15 months	Quality of life (EQ-5D-3L)<br>15 months<br>No
Schneider, 2015, USA	Type 2 diabetes<br>Depression	Physical-Mental	29<br>15<br>14	6 months.<br>Baseline, 3 months, 6 months.	Clinical outcomes (HbA1c) and depression (BDI-II)<br>3 and 6 months<br>No

For peer review only

Takahashi, 2012, USA	Myocardial infarction<br>Congestive heart failure<br>COPD<br>Diabetes<br>Renal disease	Physical-Physical	205<br>102<br>103	12 months<br>Baseline, 12 months	Hospital admissions<br>12 months<br>No
Vera, 2010, USA	Depression<br>And one or more of:<br>Diabetes<br>Hypothryoidism<br>Asthma<br>Hypertension<br>Chronic bronchitis<br>Arthritis<br>Heart disease<br>High cholesterol<br>Stroke	Physical-Mental	179<br>89<br>90	6 months.<br>Baseline, 8, 16 and 24 weeks.	Depression (SCL-20) and Functioning (SF-36)<br>6 months<br>Yes
Wakefield, 2011, USA	Type 2 diabetes<br>Hypertension	Physical-Physical	302<br>102 (low intensity) / 93 (high intensity)<br>107	6 months.<br>Baseline, 6 months, 12 months	Clinical (HbA1c and SBP)<br>6 month<br>Yes
Williams, 2012, Australia	Diabetes<br>Chronic kidney disease/diabetic kidney disease<br>Hypertension	Physical-Physical	80<br>39<br>41	3 months.<br>Baseline, 3 months, 6 months, 9 months.	Medication adherence<br>3-6-9 months<br>No

For peer review only

Wilson, 2018, USA	Depression<br>Any other chronic disease (most common: chronic pain, multiple sclerosis, cardiovascular disease, mental disorder)	Physical-Mental	53<br>27<br>26	8 weeks.<br>Baseline, 4 weeks, 8 weeks.	Depression (PHQ-8)<br>8 weeks<br>Yes
Yu, 2020, Canada	Diabetes AND two of:<br>Heart disease<br>Stroke<br>Hypertension<br>Cancer<br>Chronic lung disease<br>Arthritis<br>Inflammatory bowel disorder<br>Urinary incontinence	Physical-Physical	10 primary care practices were randomsed (5 in each arm).<br>213<br>102<br>111	6 months<br>Baseline, 6 months, 12 months.	Behaviour change (Decisional conflict)<br>12 months<br>No

For peer review only

Category	Quality assessment (RoB-2)	Intervention name, description and modifications	Intervention components<br>Leader<br>Recipient<br>One-to-one or Group<br>Mode of delivery and location<br>Number/length sessions
Collaborative care	Low	Collaborative care intervention targetting patient-, clinician-, and system-level improvements. Administered PHQ-9 to monitor depression, reviewed glucose logs, and counseling toward achieving treatment goals. Intergrated decision making tool for HCPs within electronic health record system	Psychiatrist and diabetologist and care co-ordinators<br>Person with depression and T2DM<br>One-to-one<br>Face-to-face in clinic or telephone<br>Every 2-4 weeks for 12 months
Self-management	Low	DROP (DepRessiOn and Pain) programme:<br>1. Optimised management of major depression through computerised clinical guideline to aid decision making<br>2. Care management of patient pain through calls with psychologist<br>3. Psychoeducational intervention group programme, with discussions and homework covering: understanding pain; managing emotions and attention; basic relaxation techniques; cognitive restructuring strategies; problem-solving; setting life goals; relationships between pain and physical activity; healthy posture and sleep; maintenance of the strategies learned; and a relapse plan	Care co-ordinator (psychologist)<br>Primary care physicians and people with conditions<br>Primary Care Centres<br>Face-to-face - 9 weekly 2 hour group sessions<br>Telephone - Monthly for first 3 months and then every 3 months for next 9 months
Self-management	Low	Personalised Care: the nurse case manager conducts a standardised, face to face, biopsychosocial assessment. Patients then identify up to three problems which contribute to their depression and they want to address. The nurse-case managers provide information, sign-post to existing resources and use evidence based behaviour change techniques to help patients set and achieve goals. The underlying intention of the intervention is to increase the patient's self-efficacy to achieve their desired goals. The action plan is recorded in a 'personalised health plan' which the patient holds.	Nurse<br>Person with CHD and depression<br>One-to-one<br>Face-to-face and telephone<br>1 hour session<br>Weekly 15 minute phone calls for 6 months
Cognitive and/or Behavioural Activation	Some concerns	Allocated a family support worker from Making Space, providing information, giving advice on benefits, advocacy, emotional support, and practical help. The integrated treatment program attempted to combine three treatment approaches: motivational interviewing, individual cognitive behavior therapy, and family or caregiver intervention.	Family support worker<br>Patient and primary care giver dyad<br>Face-to-face<br>Home or clinic<br>23 weekly sessions followed by 6 biweekly sessions and 10-16 family sessions

For peer review only

Collaborative care	Low	Nurse care management system.<br>Initial consultation to review medical, lifestyle, and social status, and develop a self-management action plan. Group classes followed a workbook specifically created for the program and included some didactic material, although the focus was on group discussion, participation and problem-solving. Follow-up calls were used to review patient goals, medication use, glucose/BP monitoring, and self-management activities. Higher levels of depression and alcohol problems were also addressed. Algorithims were used throughout for medication managament.	Nurse care manager<br>Patient<br>Group classes once a week for 4 weeks (1-2 hours)<br>8 Follow-up telephone calls lasted 15 minutes, over 9 months.
Self-management	High	Flinders Programme<br>Targeted self-management support to assist the patient to achieve improved health outcomes over a period of 12 months. A health professional is trained to tailor the interventions to meet the persons needs whilst simultaneously motivating the patient to achieve their medical, psychosocial, or lifestyle risk factor goals. Veterans offered SCDSMP course (Stanford School of Medicine) if considered appropriate - this builds the person's self-efficacy by teaching skills in problem-solving, decision-making, resource utilisation, managing the patient–provider partnership, action planning and emotional management	Research nurse (RN)<br>Veterans<br>One-to-one<br>Face-to-face: begins with developing care plan, follow-up visits monitor outcome of this plan<br>SCDSMP: 6 weekly face-to-face group sessions (2.5 hours each) delivered jointly by research officer and veteran peer leader to veterans and their partners if desired
Self-management	Some concerns	Preventing Aids Through Health (PATH)<br>Driven by theory of reasoned action; helps patients actively cope with barriers to medication adherence and promote participants self-care abilities. The protocol includes one face-to-face meeting a week as a minimum with basic intervention (psychoeducation, pillboxes, beeping watches). The intervention was then cascaded to suit the needs of the individual if adherence fell - this was a gradual increase in intensity and included activation of social networks, followed by use of beepers with alphanumeric displays, then prepaid cellular phones to encourage participants to follow their regimen, then the final step was directly observed therapy.	Advanced Practice Nurse (same nurse each session where possible)<br>Patient<br>One-to-one<br>Community-based: weekly face-to-face home visits from the APN
Cognitive and/or Behavioural Activation	Low	"Minder Zorgen" (Worry Less) - a guided self help intervention using Cognitive Behavioural Therapy with a focus on coping skills. Five modules with texts, examples and assignments that patients could access online from their own computer. This intervention was an existing IPST and adapated for MS patients.	Psychologists and masters student<br>Patient<br>One-to-one<br>Online - digital<br>One module a week for 10 weeks

For peer review only

Collaborative care	Low	Integrated Care Intervention.<br>Involved education about depression and hypertension, emphasizing the importance of controlling depression to manage hypertension; encouragement and relief from stigma; helped to identify target symptoms for both conditions; explained the rationale for antidepressant and antihypertensive medication usage; assessed for side-effects and assisted in their management; assessed progress; assisted with referrals; and monitored and responded to life-threatening symptoms.	Integrated care manager (ICM)<br>Patient<br>One-to-one<br>3, 30-minute in-person sessions and 2, 15-minute telephone-monitoring contacts during the 4 weeks
Collaborative care	Low	CMHC-based collaborative care model, adapted from the primary care–based TEAMcare model and adapted for patients with psychosis.<br>Initial 60-min nurse care manager visit for a comprehensive health assessment and an individualized health plan and then 30-minute visits for the support of chronic illness self-management (including medication adherence, healthy nutrition, and regular physical activity) every other week for 12 weeks. Nurses used motivational interviewing and behavioural activation to address barriers to self management. Diabetic education materials were modified to address needs of patients with psychosis.	A CMHC nurse care manager, a CMHC psychiatrist, the advanced practice registered nurse who provided primary care onsite at the CMHC, and an endocrinologist consultant.<br>Patients<br>One-on-one<br>Hospital clinic
Cognitive and/or Behavioural Activation	Low	CBT (plus usual care): Session content used cognitive techniques to identify and challenge general and diabetes-specific cognitive distortions that result in maladaptive behavior, in combination with behavioral techniques, including behavioral activation and specific behavior change strategies related to diabetes and/or mood. Those with more intermediate concerns were also provided with problem solving therapy focusing on effective coping and adaptive problem-solving skills.<br>Small Changes Lifestyle Coaching (plus usual care): Focused on lifestyle modifications to improve diabetes and mood.<br>Patients were reevaluated at 6 months and either entered a maintenance phase, or switched to CBT or SCLC.	SCLC - nurse care manager; CBT - a psychologist and clinical health psychology doctoral student; and a community health worker provided navigation and social support.<br>Patient.<br>One-on-one.<br>12 tailored sessions 30-60 minutes long for the first 6 months of either:<br>CBT: Face-to-face or over the telephone if necessary.<br>Lifestyle: over telephone

For peer review only

Self-management	Some concerns	Health and Recovery Peer Programme (HARP)<br>The program builds on the Chronic Disease Self-Management Program (CDSMP), a 6-session health education program. It provides skills that help patients become more effective self-managers including modeling of healthy behaviors, problem-solving, reinterpretation of symptoms, and training in specific disease management techniques. Action plans help develop self-efficacy and build to long-term health goals.<br>Modifications of CDSMP to HARP addressed specific patient, provider, and community level determinates of health in patients with comorbid medical and serious mental disorders. Materials were added addressing the mental health impact of chronic conditions and dealing with social isolation.	Two certified peer specialists led each session.<br>Patient.<br>Six 2.5 hour face-to-face sessions in groups of 6-10, with one-on-one peer coaching meetings held in between sessions for reinforcement.
Collaborative care	Low	Multifaceted Diabetes and Depression Program (collaborative care intervention).<br>Patients chose problem-solving therapy or antidepressant, and a stepped-care algorithm was used to ensure patients received treatment consistent with their preference, clinical presentations and responses over time.<br>Usual care was enhanced by providing patients with depression educational pamphlets and a community resource list.	1) PST provided by bilingual graduate social work diabetes depression clinical specialists (DDCSs) and/or antidepressant medications prescribed by the treating primary care physician.<br>Patient.<br>One-to-one but support group sessions available.
Self-management	Some concerns	A-Helping-hand (AHH)<br>Psychoeducational intervention following a problem-solving framework, using self-care management of chronic care conditions as a specific skill-building focus with review of<br>healthy behaviors outlined in the written materials provided to patients. Intervention provided problem-solving modeling and opportunities for patients to practice problem solving skills: 6 tasks included engagement, problem formulation, education, action planning and evaluation.<br>Study participants in both groups were given depression educational brochures, depression photo novels, and materials on specific chronic illness treatments and self-care.	3 promotoras (trained and supervised by the research team) were added to a standard team of physician, nurse, and medical assistant.<br>Patient.<br>One-to-one.<br>Six sessions over six weeks administered face-to-face (or telephone if preferred) at the patients home/any other setting preferred by patient, followed by 3 monthly booster sessions for reinforcement.

For peer review only

Self-management	High	Self-management intervention.<br>An individualised care plan was developed to help self-management of chronic conditions, and caregivers were actively consulted in all aspects of care. Focus on client building on their strengths and abilities to enhance self-efficacy and achieve personal goals.<br>Adapted from two previous community-based self-management interventions for older adults (targeting diabetes and stroke) to target multi-morbidities.	Care Coordinator, Registered Nurse, Physiotherapist, Occupational Therapist, and Personal Support Worker<br>Patient and informal/family caregiver.<br>One-to-one<br>1. Minimum of one in-home visit from CC and 3 from PT/OT.<br>2. Monthly case conferences with provider team to discuss ongoing care plan.<br>3. Ongoing case management by CC's which involved system navigation and facilitating participant access to appropriate health and social services.
Self-management	Some concerns	Occupational therapy led self-management support programme (OPTIMAL).<br>Occupational therapy focus with peer support; goals were discussed and revised at each session, considering patient preference. Intervention components included: self-management; fatigue and energy management; managing stress/anxiety and maintaining mental health/wellbeing; keeping physically active; healthy eating; managing medications; effective communication strategies; goal setting.<br>Based on the Stanford Chronic Disease Self-Management Programme: key adaptations were an occupational therapy focus, groups being professionally led and a clear focus on the specific challenges of multimorbidity.	Facilitated and led by local community-based occupational therapists.<br>Patient.<br>Group.<br>Weekly 3-hour meetings for 6 weeks held in local community health centres.
Self-management	Some concerns	Living Well.<br>Sessions focused on action planning, peer feedback, support, modeling, problem solving, training in specific disease management techniques and the application of these skills to the topics of nutrition, exercise, sleep, medication management, addiction behaviours.<br>Modified from CDSMP for patients with serious mental illness as follows - delivery by two mental health peers OR mental health provider and peer; increased length of intervention; action plan tracker and health workbook; extra module on communication with HCP's; extra topics on mental illness; two monthly booster sessions after intervention.	Six groups - four were cofacilitated by two mental health peers who had at least one chronic condition, two were cofacilitated by a mental health professional and a peer.<br>Patients.<br>Group.<br>Weekly 60-75 minute face-to-face sessions and two monthly booster sessions post-intervention.<br>Clinics

For peer review only

Collaborative care	High	Collaborative Care<br>Guided self-help and problem-solving treatment, prescription of anti-depressant medications according to an algorithm, and consultations with psychiatrist if necessary.	Provided by a team consisting of patient, consultant psychiatrist nurse, and consultant-liaison psychiatrist.<br>Patient.<br>One-to-one.<br>Face to face at outpatient clinic of general hospital.
Self-management	Low	Combined Diabetes and Renal Control Trial (C-DIRECT) Intervention (plus usual care).<br>Centered around self-management strategies. Patients chose a topic to focus on at each session. Session 1: feedback on Hba1c; information/advice on chosen topic; goal setting; food record assignment. Session 2: review goal setting and problem solve any barriers; revise goals as needed; review food record and provide support if necessary; choose topic (information/advice and new goal setting). Session 3: review goals and problem solve barriers; revise goals as needed; choose topic (information/advice and new goal setting). Provide additional advice and resourse links to go away with.	DM link nurse.<br>Patient.<br>One-to-one.<br>3 weekly sessions 30-60 minutes long, delivered at patient bedside at dialysis centre upon cannulation and connection to haemodialysis machine.
Collaborative care	Low	mobile Atrial Fibrillation Application (mAFA).<br>Installed on smart phones. Provided clinical decision support tools for doctors to facilitate treatment recommendations, educational materials, patient self-care, and structured follow-up, to support AF management: Avoid stroke (tracking bleeding events/trends; managing treatment and anticoagulant use), Better symptom management (tracking symptoms, monitoring cardiac rhythm using smart watches), Cardiovascular and other comorbidities risk management (tracking BP and lifestyle factors). The app encouraged patient engagement through participation in educational programs, provision of informative articles, videos, game playing, etc.	Doctors.<br>Doctors and patients.<br>One-to-one.<br>Followed up at outpatient clinics.

For peer review only

Self-management	Low	TELECARE (plus usual care).<br>Usual care was frequent clinical and bio-parameters self-checks (BP, heart rate, oxygen saturation, weight, and blood glucose)Patients were provided with educational material and instructed how to recognize exacerbation symptoms. For the intervention group, a synchronous monitoring equipment was added for biological and medical questionnaires so data was available in real time for healthcare team. An alarm system was added to induce a healthcare response if there was variation in data or missing data.	Health care team<br>Patient tracked own data.<br>One-to-one.<br>Patients inserted data daily for first 15 days (frequency was then altered depending on stability and ocurrence of incidents).
Self-management	Some concerns	Supported self-management<br>The intervention emphasized realistic goal setting, identifying resources and barriers likely to influence success in reaching goals, and regular self-monitoring of goal attainment.<br>Four components: (1) Establishing the participant's daily routines and lifestyle; (2) Identifying all supporters and helpers and their roles; (3) Setting realistic goals for change; (4) Monitoring progress against agreed upon goals.<br>Materials: for nurses - weekly timetable, charts to record friends/family/helpers, charts to complete in collaboration with participant; for participants - OK Diabetes board to record goals and visual prompts, a written action plan, tear off slips to record daily actions; for supporters/helpers - information sheet on study, card summarising their role in helping the person with diabetes.<br>The intervention was modelled on existing self-management interventions, with adjustments made to respond to barriers to self-care in people with an intellectual disability.	Diabetes specialist nurse.<br>Patient (73% had their supporter present for at least one session).<br>One-to-one.<br>4 sessions, most (92%) took place in participants homes, ranging from 13-95 minutes (typical total intervention time was 2 hours).

For peer review only

Self-management	Low	Pharmaceutical care (PC) (plus routine care). Routine care - BP taken by nurse, physician prescribes medication/orders lab tests, meeting with adherence counsellor, then medication/ counselling from pharmacist. Intervention also exposed to PC from research pharmacist. This involved structured education/ counselling focused on self-monitoring of BP, healthy eating, maintaining an ideal body weight, proper foot and dental care, healthy coping, use and storage of medications; lifestyle modifications with emphasis on the dietary approaches to stop hypertension (DASH) diet, physical activity, smoking cessation, moderation of alcohol intake; reviewing date of the next appointment and prescription with the patient, expected side effects of the medications, and how to prevent/ manage such side effects.	Research pharmacist. Patient. One-to-one. Face-to-face bi-monthly appoointments at hospital, as well as two text messages per month.
Collaborative care	Some concerns	Care management intervention. Embedded in a regional network of primary care practices. It included two home visits with structured assessments of clinical and social needs, aiming to reduce barriers to patient's access of support and healthcare systems. Frequent telephone monitoring was also used to improve and sustain changes in self-care behaviour, and give feedback related to patients' control of diseases and current (self-care) activities to reinforce positive behavioral changes.	Specially trained medical assistants, physicians specialising in family/internal medicine, and net-care managers. Patient. One-to-one. Two home visits and 15 structured telephone monitoring contacts over 9 months.
Collaborative care	Some concerns	Hepatitis C Translating Initiatives for Depression Into Effective Solutions (HEPTIDES) The intervention included: participant education and activation, assessment of treatment barriers and possible resolutions, depression symptom and treatment monitoring, substance abuse monitoring, and instruction in self-management. The intervention used a stepped-care model for depression treatment, with five steps: self-management education, depression care team treatment suggestions, pharmacotherapy suggestions, combination pharmacotherapy and specialty mental health counseling, and referral to specialty mental health.	Depression care manager, pharmacist and psychiatrist. They convened once a week and communicated with HCV and mental health clinicians at each hospital site to discuss treatment and make decisions. Patient. One-to-one via telephone delivered by DCM, every 2 weeks during acute treatment and every 4 weeks during self-management education or continuation treatment.

For peer review only

Collaborative care	Low	Collaborative care.<br>Individualised, stepped-care depression treatment programme designed to improve quality of care and outcomes of depression. Patients were offerd an intial choice of two treatments: antidepressant medication or problem solving treatment (PST). If patients still had persistent depressive symptoms (< 50% decrease in severity based on the PHQ-9) 10 to 12 weeks after initial treatment, they could (1) switch to a second antidepressant; (2) switch to the alternative treatment (from PST to medication or vice versa); (3) receive augmentation with PST or medication with the first treatment they had received; or (4) receive a psychiatric consultation. If there was still no improvement after a further 8 to 12 weeks, referral to speciality care was made. Once patients reached a significant decrease in clinical symptoms (< 50% decrement in symptoms), the nurse began continuation phase treatment.	Depression clinical specialist nurse in collaboration with the primary care physician.<br>Patient.<br>One-to-one.<br>Initial 1-hour visit followed by twice-a-month, half-hour appointments (telephone and in person) for 12 weeks followed by monthly scheduled telephone contacts for continuation phase treatment.
Collaborative care	Low	Collaborative care management.<br>The intervention combined support for self-care with pharmacotherapy, aiming to manage depression and improve glycemic, BP and lipid control. Patients worked collaboratively with nurses and primary care physicians to establish individualized clinical and self-care goals. Using motivational and encouraging coaching, nurses helped patients solve problems and set goals for improved medication adherence and self-care. Patients also received self-care materials and self-monitoring devices. Nurses monitored the patient's progress and medications were adjusted where necessary according to protocol. Once target levels were achieved, a maintenance plan was developed that included stress reduction, behavioral goals, continued use of medications, and identification of prodromal symptoms associated with worsening depression and glycemic control.	Nurses, in collaboration with primary care physicians.<br>Patient.<br>One-to-one.<br>Visits every 2 to 3 weeks at the patients primary care clinic; once maintenance plan was in place. Patients whose disease control worsened were offered follow-up visits and telephone calls with increased treatment intensity.
Self-management	Low	Movement through Active Personalised engagement (MAP) programme.<br>Four sessions with person-centred self monitoring and goal setting. The sessions focused on increasing PA, and addressed key non-disease-specific self-management challenges and themes (mastering emotions, managing treatments, communication within health care). Increasing PA was further facilitated and encouraged by providing resistance bands in order to complete resistance training at home and pedometers to help track PA. Regular reminder and motivational texts were sent to encourage long-term behaviour change.	Trained facilitator.<br>Patient.<br>Group.<br>1.5 hour face-to-face sessions delivered at two week intervals in local community.<br>Texts were sent daily during the 8 weeks of sessions up until 2 weeks after the 6-month timepoint, then 3 times a week over the remainder of the 12 month period.

For peer review only

Cognitive and/or Behavioural Activation	Some concerns	Structured counselling/education. At each visit, blood glucose level was measured, and DM medication was adjusted according to a structured algorithm. Educational messages were discussed according to a specified checklist using flip charts. Topics included: TB: Cause, transmission, treatment compliance, possible side effects; What is DM? What are the symptoms? What can be done?; Blood glucose measurement and recording; Importance of glycaemic control; Metformin and insulin; Hyper- and hypoglycaemia; The importance of smoking cessation; DM and TB together; Healthy lifestyle: diet, exercise, and smoking cessation; Management after the TB treatment is completed. A leaflet was also provided to patients with information regarding recommended food types and amounts.	Doctor. Patient. One-on-one. Face-to-face in a research clinic at weeks 1, 2 and 4, then monthly until 6 months.
Collaborative care	Low	Stepped Care for Affective Disorders and Musculoskeletal Pain (SCAMP). Step 1 (weeks 1-12): Optimised antidepressant therapy. Adjustments in medication as per an algorithim to optimise antidepressant management. Step 2 (weeks 13-26): Pain self-management programme. Focus on increasing self-efficacy, social support to self-manage pain, problem solving techniques, behavioural change strategies, learning about chronic pain and receiving feedback from nurse. Step 3 (weeks 27-52): continuation phase. Evaluation. The care manager assessed current self-management strategies and assisted patients with new behavioral plans. Antidepressant dose could be increased, or if necessary a referral to a psychiatrist offered.	Nurse care manager and physician depression specialist. Patient. One-to-one. 5 inperson contacts (baseline, 6, 12, 16, and 20 weeks) and 8 telephone contacts (1, 3, 9, 14, 18, and 22 weeks, and at 8 and 10 months). Extra contacts could occur depending on treatment changes or a patient's clinical needs.
Self-management	Low	Internet chronic disease management (CDM). Participants used the CDM website to complete daily symptom reports which asked questions about disease-specific symptoms and biometric data, which generated alert for specific scenarios or targets not being met. The nurse would then call to discuss/readjust targets, and support patient self-management or recommend follow up with primary care practicioner/hospital referral if necessary. Every 8 weeks, participants answered a lifestyle questionnaire, and alerts were sent based on thresholds for these questions. If necessary the nurse gave a referral to a dietitian/exercise specialist or use of a psychosocial support workbook. Participants had access to a public forum, graphical presentations of their biometric data overlaid with relevant alerts, their action plan, and external online resources.	Coordinated care between participant, their primary care practicioner, and a nurse managing the CDM programme (supported by a dietitian and an exercise specialist). Patient. One-to-one. Telephone.

For peer review only

Self-management	Some concerns	Self-management intervention.<br>Week 1: knowledge on COPD/ comorbidities, symptom recognition/ monitoring, self-treatment action plan (linked to diary), breathing/relaxation exercises, how to check blood glucose. Week 2: record 'usual symptoms', diary training, exacerbation action plan training, mastery of skills (e.g. inhaler use). Week 3: importance of exericse, diet/lifestyle behaviours, re-iteration of diary/action plan use, reiteration of breathing/relaxation. Week 4: re-iteration of diary/action plan use, feedback on diary completion, feedback on actions.<br>Both intervention and usual care groups completed daily symptom diaries for the 12 month period. For the intervention group, self-management behaviours were reinforced, and exacerbation action plans for all co-morbidities were directly linked to their diaries.	Researchers<br>Patient.<br>Weeks 1 and 3: group. Weeks 2 and 4: individual. Phone calls at weeks 8, 20, and 36.<br>Location of face-to-face sessions unspecified.
Self-management	Low	Self-management programme.<br>The intervention focused on self-efficacy, self-management, holistic care, and individual and caregiver engagement. It was client-driven therefore fleixble to client needs; the indvidual was fully engaged in developing and tailoring their own care plan. The programme offered up to 3 in-home visits from nurse/dietitian/both, monthly group wellness sessions, monthly case conferences amongst the team, and ongoing nurse-led care coordination.	Nurses and dietitians, a program coordinator from a community partner, and peer volunteers.<br>Patient and carer (optional)<br>One-on-one sessions at patients home, group sessions at diabetes education centre.
Self-management	Low	"Getting better at chronic care".<br>Health-worker led case management intervention (one recruited at each site to work as part of primary care team and given caseload of 9 to 26 clients). The roles of the health workers included helping patients make and keep appointments, understand their medications and nutrition and the effects of smoking and work with the family to help support the patient in self-management. Home visits and out-of-clinic care were conducted according to patient preference. Specific training and practice in: chronic care model and evidence-based management and treatment goals for chronic conditions; "hands-on" case management (regular client home visits, including basic diabetes care); working in a primary care team with clear roles/ responsibilities; engaging with families and using local resources to support effective client self-management.	Indigenous healthcare worker (IHW) from community.<br>Health worker/client.<br>One-to-one.<br>Home visits OR out of clinc care (based on preference).

Self-management	Some concerns	Skills TO Manage Pain (STOMP) plus usual care. Incorporated self-regulation, self-efficacy, observational learning, and outcome expectations. Individual sessions: to build pain self-management skills. All participants received a pain education session, and then selected 5 of the remaining 9 sessions (physical activity and your pain, losing weight to improve your pain, relaxation skills to prevent your pain, sleeping better to help your pain, thinking diferently about your pain, building self-worth, talking with our family and friends about pain, taking opioid pain medications).<br>Group sessions: to enhance peer support related to chronic pain. Each session included sharing refections on lessons learned and goals set during one-on-one sessions, and challenges encountered.	Two peers with HIV and chronic pain ("pain pals") and two research staff ("pain coaches"), working in pal-coach pairs.<br>Participant.<br>Six (in-person) one-to-one sessions (led by pain coach) and six group sessions (led by pal-coach pair), alternating weekly.
Collaborative care	High	Aging, Community and Health Research Unit Community Partnership Program.<br>Focused on the role of self-efficacy in achieving self management behaviours, and tailored to patient preferences and needs.The program included up to 3 home visits by RN or RD; 6 monthly group sessions which included education, exercise, a light meal, and peer support; monthly intervention team case conferences (share observations about participants strengths, challenges and goals, identify needs related to other community-based service, and prepare for upcoming group sessions); care coordination to link the client to relevant health or social services.	Interprofessional team consisting of a Registered Nurse (RN), Registered Dietitian (RD), and Program Coordinator (PC).<br>Patient (family caregiver was also invited to attend).<br>One-to-one sessions at patients home, group sessions at community site once a month.
Cognitive and/or Behavioural Activation	Some concerns	TrueBlue<br>Collaborative care approach. Involved visit with PN followed by usual GP, in which stepped care (psychotherapy or pharmacotherapy) was offered if PHQ-9 scores had not improved or dropped below 5. The PN obtained physical measures and reviewed recent pathology results and lifestyle risk factors. They worked with the patient to identify barriers to achieving goals and discuss ways to overcome them. They assisted the patient with self-management by discussing available educational resources and setting personal goals for review at the next visit.	Practice Nurse (PN)<br>Patient<br>One-to-one.<br>Patient visited their practice every 3 months for 45 minutes (then 15 minutes with the GP).

For peer review only

Self-management	Low	Healthy Outcomes through Patient Empowerment (HOPE).<br>The first 2 sessions focused on building rapport, introducing and clarifying values, collaboratively setting initial goals, identifying potential skill sets to address goals, and empowering patients to advocate for their health through active communication with clinicians. Sessions 3-6 focused on discrete skill modules (increasing pleasant activities, using thoughts to improve wellness, diet, physical activity, medication management, and relaxation) customized to meet their goals. Sessions 7-9 focused on maintenance skills (reviewing action plans and overcoming barriers).	Trained health professional.<br>Participant.<br>One-to-one.<br>9 telephone sessions: biweekly (30-40 minutes) in months 1-3, and monthly (15 minutes) from months 4-6). No contact in following 6 months.
Self-management	Low	Care management intervention.<br>3 steps: (1) Structured, IT-supported assessment performed by net-care managers to capture medical/non-medical needs; (2) goal setting between patient and primary care physician based on results of assessment; (3) telephone monitoring by net-care managers to detect acute clinical needs and provide support.<br>Embedded in PCN (net-care manager links to responsible physician via a medical assistant).	Net Care Manager.<br>Patient.<br>One-to-one.<br>(1) at-home visits/face-to-face meetings 3 times within initial 2 weeks, then again at 6 months; (2) once at week 2; (3) every 2 weeks for the first 6 months then every month for the next 3 months.
Self-management	Some concerns - a lot of analyses were completed	Occupational therapy-led self-management support programme (OPTIMAL).<br>Intervention incorporated performance accomplishments, vicarious learning, social/verbal persuasion, reinterpretation of physiological and emotional states. Patients' programme goals were set in week 1 and reviewed weekly. They were also given educational and health-promoting resources/materials. Week 1: Introduction to self-management, activity and health, and goal-setting. Week 2: Fatigue management and healthy eating. Week 3: Maintaining physical activity. Week 4: Maintaining mental wellbeing. Week 5: Managing medications. Week 6: Communication and programme review.	Occupational therapist.<br>Patient.<br>Group.<br>2.5 hour face-to-face session once a week for 6 weeks, in a primary care centre or community resource centre.

For peer review only

Cognitive and/or Behavioural Activation	Low	Health coaching self management programme (HCSMP).<br>Group level approach: structured health education developed for older adults, regarding participants' diseases and self-management strategies, then a period of physical activity to enhance cognition and body movements.<br>Individual level approach: individual counselling and goal setting for self-management behaviours.<br>Facility-level approach: principal investigator met frequently with the director and chief manager of the nursing home to help support and facilitate patient progress.	Trained research team members who were geriatric nurse speciaists.<br>Participant.<br>Group sessions delivered face to face at nursing home for 8 weeks; education (Monday) and exercise (Thursday), both lasting 1 hour.<br>Individual sessions: 8 face-to-face coaching sessions for approx 20 minutes,
Self-management	Low	Psycho-education.<br>Four interactive meetings on the following topics: (1) symptoms of depression, interaction of depression and diabetes; (2) alleviaing burden of depression through activities and problem solving; (3) associations between depression and cognitive processes – thoughts, beliefs and attitudes that induce and maintain depression; (4) developing a personal plan for managing depression-related problems in the future. Patients were provided with a self-help manual and kept daily mood and activities diaries.	Not specified.<br>Patient.<br>Group.<br>Four 90 minute sessions - first two held within the first week and the third and fourth held at two week intervals
Cognitive and/or Behavioural Activation	Low	Multi-component, case manager led intervention. Components included: (1) standardised 40 minute COPD education session on enrolment; (2) individualised care and action plans for COPD exacerbation recognition, self-management and management of comorbidites; (3) telephone consultations containing standardised reinforcement/ motivational interviewing focusing on health behaviours, action plan teach-back sessions, symptom monitoring, and problem solving strategies; (4) ongoing case manager communication with family physicians and with hospital specialists including respirologists; and 5) priority access to ambulatory outpatient clinics.	Case manager.<br>Patient.<br>One-to-one.<br>12 weekly telephone calls (after initial education session at ED/hospital/outpatient clinic), followed by monthly calls for 9 months.

For peer review only

Cognitive and/or Behavioural Activation	Some Concerns - multiple scales used to measure the same outcome	CBT-AE.<br>CBT: (a) education about anxiety and panic; (b) cognitive therapy techniques aimed regarding anxiety and panic; (c) training in slow diaphragmatic breathing to aid physical symptoms of panic attacks; and (d) interoceptive exposure exercises designed to reduce participants' fear of anxiety/panic attack symptons. AE: (a) information about airway inflammation and bronchospasm; (b) rationale, correct use, and side effects of medications, (c) inhaler techniques; (d) methods of self-monitoring, symptoms, and peak flow; (e) asthma triggers and strategies for control; and (f) action plans. Asthma diaries kept by the participants were reviewed during each treatment session to reinforce the importance of the participant's role in self-management and self monitoring.	Two nurse clinicians (one Asthma Educator, one with postdoctoral training in cognitive therapy).<br>Participant.<br>Small groups.<br>12 face-to-face 90 minute sessions with both CBT and AE components. Sessions 1-8 conducted twice weekly, and sessions 9-12 spaced one week apart.
Self-management	Some Concerns	Targeted Training in Illness Management.<br>Group-based psychosocial treatment that blends psychoeducation, problem-identification, goal-setting, behavioral modeling, and care linkage.	Nurse and peer educators.<br>Participant.<br>Group/one-to-one.<br>12 weekly face-to-face sessions, followed by 15 minute one-to-one telephone calls every 2-4 weeks.
Collaborative care	Some concerns - self-completed measure for primary outcome and all were aware of group allocation	3D intervention<br>Replace separate disease focused review with one 6-monthly comprehensive multidisciplinary review. Done in 3 stages: (1) nurse review - identifies important health problems and how these affect life, and disease-specific care that the patient needs; (2) pharmacist reviews medication and makes recommendations for treatment; and (3) physician review - considers nurse and pharmacist reviews, then agrees on collaborative health plan with patient (which is printed off) specifying goals and how these will be achieved over the next 6 months of consultations.	Nurse, physician and pharmacist.<br>Patient.<br>One-to-one.<br>Two 6-monthly reviews consisting of two appointments (nurse and physician) and a medication review, then consultations over 6 months as per the health plan.
Cognitive and/or Behavioural Activation	Low	Behavioural Activation and Exercise Condition.<br>Classes contained 3 parts - (i) warm-up, (ii) exercise (different types for first 12 weeks then based on paricipant enjoyment for next 12, gradually increasing in intensity each week), and (iii) cool down and BA (activity and mood monitoring, exploration of ways to make exercise more enjoyable, a behavioural contract with friends/ family to promote exercise, a list of life values and how to connect exercise to them, and exercise counseling strategies including self-monitoring activity using a pedometer, monitoring exercise intensity via perceived exertion and heart rate, identifying facilitators to exercise and engaging in problem solving for exercise barriers).	Female group leader.<br>Participant.<br>Group.<br>One orientation session and 38 classes over 4 weeks at a medical school site. The 90-minute classes occurred 2 times per week for 16 weeks, once a week for 4 weeks, and then once every other week for 4 weeks.

For peer review only

Self-management	Some concerns - outcome assessors were not blind at follow-up, more deaths in intervention	Telemonitoring.<br>An FDA approved telemonitoring device was used in the patients home. Patients performed daily assessment of symptoms and biometrics, and data were downloaded and reviewed daily by the health care team. A registered nurse communicated with the individuals via phone or videoconference if alerts arose, communicating with their primary physician about treatment options if needed.	Registered nurse and (if required) primary physician oversaw the process.<br>Patient.<br>One-to-one.<br>Daily 5-10 minute sessions<br>Home or clinical
Collaborative care	Low	Collaborative care.<br>A multicomponent intervention, including program oversight and teamwork among physicians, mental health specialists, and care managers. Patients were provided education about depression and a choice of 13 CBT sessions or antidepressant medication as initial treatment. The care manager participated in the coordination of treatment initiation, as well as monitoring treatment adherence, side effects, and clinical response, while liaising with the psychiatrist and patients physician. Extra contacts were available to help patients overcome barriers and provide treatment adherence support.	Care manager.<br>Patient<br>One-to-one.<br>Patients were contacted by telephone or in person every 2 weeks initially, and then monthly up to 6 months.
Self-management	Low	Telehealth intervention.<br>In a home telehealth device, all intervention participants logged blood pressure, blood glucose and responses to standardised questions (which were reinforced when answered correctly and reviewed/explained when answered incorrectly), which were reviewed daily by a registered nurse to determine whether the participant needed a follow-up (e.g. if clinical parameters were out of range or the participant needed extra support). In the high intensity group, a disease management algorithm was used, with scheduled prompts for rotating questions and education focused on diet, exercise, smoking cessation, foot care, advice for sick days, medications, weight management, preventive care, and behavior modification and lifestyle adjustments. In the low intensity group, participants responded to a smaller subset of questions from the HI group and did not use the algorithm.	Nurse.<br>Participant.<br>One-to-one.<br>Telehealth device was in patient home - follow-ups were made via telephone, letter or directly through the device as and when needed.
Self-management	Low	Medication Self-Management Intervention (MESMI).<br>The intervention consisted of self-monitoring of blood pressure (participants were taught how to do this and recorded it daily for 3 months), an individualized medication review, a 20-minute DVD (using an interactive, psychosocial approach to motivate people to take their medications), and motivational interviewing to support blood pressure control and optimal medication self-management.	Intervention nurse.<br>Participant.<br>One-to-one.<br>Motivational interviewing was done fortnightly via telephone calls, starting 2 weeks after the intervention home visit until 3 months.

For peer review only

Cognitive and/or Behavioural Activation	Some concerns - as unblinded participants completing assessments, including primary outcome	Think Clearly About Depression. Computer based intervention to teach mildly/moderately depressed individuals about the role of thinking in the development and maintenance of depression, using videos, interactive activities and homework exercises to challenge depressive thoughts and practice hopeful and constructive thinking, as well as a daily mood tracker and a schedule for mood boosting self-management activities. To increase engagement, the researchers created a program guide to assist participants and sent weekly prompts for the first 4 weeks.	Research team (two psychiatric nurse practicioners and a psychiatrist). Participant. One-to-one. The program could be accessed at any time of day in any setting of the participants' choosing where they had Internet services and a personal computer or smartphone.
Self-management	Some concerns - as unblinded participants completing assessments, including primary outcome	MyDiabetesPlan. Web-based intervention where participants provide cardiometabolic and psychosocial profiles and general care priorities. An individualised action plan is then generated with diabetes-specific goals and strategies. For the first 6 months, MyDiabetesPlan was completed by a member of the clinical team with the patient, then their action plan was reviewed. At the 6-month mark patients were given a "how to" guide for MyDiabetesPlan and from then on logged the information themselves before their appointment.	Clinician (nurse, dietitian, physician, or pharmacist). Patient. One-to-one. Clinical encounters were done face-to-face at the participants primary care clinic.

For peer review only

Study	Outcome	Effective	Intervention type	Intervention length	M-LTCs combination
Blank, 2011	Clinical endpoint	No	Self-management	>6months	Physical-Mental
Chwastiak, 2018	Clinical endpoint	No	Collaborative care	3-6months	Physical-Mental
Ell, 2010	Psychological distress (depression)	Yes	Collaborative care	>6months	Physical-Mental
Fisher, 2020	Functioning	No	Self-management	3-6months	Physical-Physical
Guo, 2020	N/A	N/A	Collaborative care	>6months	Physical-Physical
Hernandez, 2021	N/A	N/A	Self-management	3-6months	Physical-Physical
Kamradt, 2019	Quality of life	No	Collaborative care	>6months	Physical-Physical
Katon, 2004	Psychological distress (depression)	Yes	Collaborative care	>6months	Physical-Mental
Khunti, 2021	Behaviour change	Yes	Self-management	>6months	Physical-Physical
Koesoemadinata, 2021	N/A	N/A	Cognitive and/or Behavioural activation	3-6months	Physical-Physical
Lear, 2021	N/A	N/A	Self-management	>6months	Physical-Physical
Lenferink, 2019	Clinical endpoint	No	Self-management	>6months	Physical-Physical
Merlin, 2018	Pain	No	Self-management	<3months	Physical-Physical
Ose, 2019	N/A	N/A	Self-management	>6months	Physical-Physical
O'Toole, 2021	Quality of life<br>Behaviour change	No<br>No	Self-management	<3months	Physical-Physical
Pibernik, 2009	Psychological distress (depression)<br>Clinical endpoint	No<br>No	Self-management	<3months	Physical-Mental
Rose, 2018	N/A	N/A	Cognitive and/or Behavioural activation	>6months	Physical-Physical
Takahashi, 2012	N/A	N/A	Self-management	>6months	Physical-Physical
Taylor, 2003	Clinical endpoint	Yes	Collaborative care	>6months	Physical-Physical
Vera 2010	Psychological disress (depression)<br>Functioning	Yes<br>Yes	Collaborative care	3-6months	Physical-Mental
Wakefield, 2011	Clinical endpoint	Yes	Self-management	3-6months	Physical-Physical
Williams, 2012	Behaviour change	No	Self-management	3-6months	Physical-Physical

For peer review only

	Physical-Physical			Physical-Mental		
**Post-intervention**	*n*	SMD [95% CI]	I^2	*n*	SMD [95% CI]	I^2
Quality of life	3	0.16 [-0.06, 0.39]	65%	5	0.10 [-0.22, 0.41]	65%
Clinical endpoints	3	-0.29 [-0.61, 0.02]	61%	8	-0.20 [-0.53, 0.12]	90%
Behaviour change	2	0.32 [-0.20, 0.84]	91%	4	-0.14 [-0.32, 0.05]	14%
Functioning	5	0.03 [-0.15, 0.20]	41%	8	0.04 [-0.09, 0.17]	45%
Psychological distress (depression)	6	-0.11 [-0.34, 0.12]	57%	15	**-0.58 [-0.94, -0.21]**	95%
Psychological distress (anxiety)	4	-0.10 [-0.19, 0.00]	0%	5	-0.17 [-0.48, 0.13]	63%
Emotional wellbeing	1	0.35 [-0.01, 0.71]	N/A	6	0.30 [-0.01, 0.61]	87%
**Maintenance**						
Functioning	1	0.08 [-0.19, 0.35]	N/A	6	0.03 [-0.07, 0.13]	0%
Psychological distress (depression)	1	**-0.15 [-0.26, -0.04]**	N/A	7	0.05 [-0.42, 0.51]	94%

For peer review only

BMJ Open

The effectiveness of complex behaviour change interventions tested in randomised controlled trials for people with multiple long-term conditions (M-LTCs): systematic review with meta-analysis

Journal:	*BMJ Open*
Manuscript ID	bmjopen-2023-081104.R1
Article Type:	Original research
Date Submitted by the Author:	20-Feb-2024

Complete List of Authors:	Rookes, Tasmin; University College London Research Department of Primary Care and Population Health<br>Nimmons, Danielle; University College London Research Department of Primary Care and Population Health<br>Frost, Rachael; University College London Research Department of Primary Care and Population Health, Research Department of Primary Care and Population Health<br>Armstrong, Megan; Queen Mary University of London Wolfson Institute of Population Health<br>Davies, Laura; University College London Research Department of Primary Care and Population Health<br>Ross, Jamie; Queen Mary University of London Wolfson Institute of Population Health<br>Hopkins, Jane; Public Contributor<br>Mistry, Manoj; Public Contributor<br>Taylor, Stephanie; Queen Mary University of London, Wolfson Institute of Population Health<br>Walters, Kate; University College London, Primary Care and Population Health

**Primary Subject Heading**:	Public health
Secondary Subject Heading:	General practice / Family practice, Research methods, Patient-centred medicine
Keywords:	Meta-Analysis, Systematic Review, Aging, Patient Reported Outcome Measures, Primary Care < Primary Health Care, PUBLIC HEALTH

SCHOLARONE™
Manuscripts

I, the Submitting Author has the right to grant and does grant on behalf of all authors of the Work (as defined in the below author licence), an exclusive licence and/or a non-exclusive licence for contributions from authors who are: i) UK Crown employees; ii) where BMJ has agreed a CC-BY licence shall apply, and/or iii) in accordance with the terms applicable for US Federal Government officers or employees acting as part of their official duties; on a worldwide, perpetual, irrevocable, royalty-free basis to BMJ Publishing Group Ltd ("BMJ") its licensees and where the relevant Journal is co-owned by BMJ to the co-owners of the Journal, to publish the Work in this journal and any other BMJ products and to exploit all rights, as set out in our licence.

The Submitting Author accepts and understands that any supply made under these terms is made by BMJ to the Submitting Author unless you are acting as an employee on behalf of your employer or a postgraduate student of an affiliated institution which is paying any applicable article publishing charge ("APC") for Open Access articles. Where the Submitting Author wishes to make the Work available on an Open Access basis (and intends to pay the relevant APC), the terms of reuse of such Open Access shall be governed by a Creative Commons licence – details of these licences and which Creative Commons licence will apply to this Work are set out in our licence referred to above.

Other than as permitted in any relevant BMJ Author's Self Archiving Policies, I confirm this Work has not been accepted for publication elsewhere, is not being considered for publication elsewhere and does not duplicate material already published. I confirm all authors consent to publication of this Work and authorise the granting of this licence.

For peer review only

The effectiveness of complex behaviour change interventions tested in randomised controlled trials for people with multiple long-term conditions (M-LTCs): systematic review with meta-analysis

Tasmin Rookes, Danielle Nimmons, Rachael Frost, Megan Armstrong, Laura Davies, Jamie Ross, Jane Hopkins, Manoj Mistry, Stephanie JC Taylor, and Kate Walters

Corresponding Author:
Tasmin Rookes: t.rookes@ucl.ac.uk

Abstract:

Introduction

Prevalence of multiple long-term conditions (M-LTCs) increases as adults age and impacts quality of life and health outcomes. To help people manage these conditions, complex behaviour change interventions are utilised, often based on research conducted in those with single LTCs. However, the needs of those with M-LTCs can differ due to complex health decision making and engagement with multiple health and care teams.

Objectives

The aim of this review is to identify whether current interventions are effective for people living with M-LTCs, and which outcomes are most appropriate to detect this change.

Methods

Five databases (MEDLINE, Embase, PsychInfo, CINAHL and Web of Science) were systematically searched, between January 1999 to January 2022, to identify RCTs evaluating effectiveness of behaviour change interventions in people with M-LTCs. Intervention characteristics, intervention effectiveness, and outcome measures were meta-analysed and narratively synthesised.

Results

Fifty-three eligible articles were included. Emotional wellbeing and psychological distress (depression and anxiety) outcomes were most amenable to change (emotional wellbeing: SMD 0.31(95% 0.04 to 0.58); depression psychological distress: SMD -0.45(95% -0.73 to -0.16); anxiety psychological distress: SMD -0.14(95% -0.28 to 0.00)), particularly for interventions with a collaborative care approach. Interventions targeting those with a physical and mental health condition and those with cognitive and/or behavioural activation approach saw larger reductions in psychological distress outcomes. Interventions that lasted for longer than 6-months significantly improved the widest variety of outcomes.

Conclusion

Complex interventions can be successfully delivered to those with M-LTCs. These are most effective at reducing psychological distress in those with physical and mental LTCs. Further research is needed to identify the effective components of interventions for people with two or more physical LTCs and which outcome is most appropriate for detecting this change.

Strengths and Limitations:

- Broad search terms across many key databases have enabled a summary of a large amount of evidence exploring the effectiveness of complex behaviour change interventions in people living with M-LTCs in a systematic review and meta-analysis.
- These findings impact on the development and implementation of such interventions in people with M-LTCs, which is a growing area of research and clinical practice.

For peer review only

- Double screening of eligible papers was only completed for a percentage of the identified studies, meaning some eligible papers could have been missed.
- Complex interventions are difficult to define and determining eligibility can be a challenge, so a conservative approach to exclusion was used.
- Some meta-analyses results had high heterogeneity, suggesting that they may not have been comparable.

Introduction:

Living with multiple long-term conditions (M-LTCs) is defined as an individual having two or more health conditions at the same time. Globally, the presence of M-LTCs is reported to be 27.2% in all adults, rising to 67.0% in those aged 74 and over (1). With an ageing population, this is predicted to rise in those over 65 in the UK, from 54% in 2015, to 68% in 2035 (2). In comparison with the general population, those with M-LTCs are found to have lower quality of life and higher mortality risk (3, 4), as well as greater healthcare costs and utilisation (5). As prevalence increases, nationally and globally, the pressure and reliance on healthcare services, people living with the conditions, and their carers also increases, making the challenge of managing M-LTCs a priority for current research agendas and healthcare systems, such as the NHS and the James Lind Alliance (1, 6, 7).

Despite this increased focus on managing M-LTCs, many of the beneficial complex interventions designed to help people with M-LTCs are based on findings from research and interventions aimed at people with single health conditions and applied more widely to those with M-LTCs. Complex interventions contain multiple interacting components and often depend on the behaviours of those delivering and receiving the intervention and need to be adapted to the specific participant group and setting (8). The chronic disease self-management program (CDSMP) has found mixed effectiveness in people with M-LTCs, depending on the combination and type of health conditions participants experience, being more effective for people with physical health conditions and probable depression (9, 10). In a recent systematic review and meta-analysis of 14 studies testing behavioural interventions in people with M-LTCs, there was little to no effect on physical activity and weight loss (11). Managing M-LTCs presents a different challenge to those with single LTCs due to the often-fragmented care across multiple primary and secondary care teams and the varied and competing needs of the individual (12, 13). These needs include complex priority setting and decision-making to adhere to multiple healthcare regimes and weighing up conflicting advice from different specialties (14). This is reflected in poorer outcomes when receiving interventions to help with the management of their M-LTCs, suggesting that the specific needs of these people are not being addressed by current interventions (15).

Systematic reviews, highlight that the effectiveness of complex interventions varies depending on the combination of LTCs, the type of intervention being used, and the outcome measure being used to assess effectiveness (9, 15). Determining the most appropriate outcome measure is particularly difficult in this population, given the wide variety of outcomes that may be relevant depending on the conditions and challenges faced by the population e.g., clinical outcomes, wellbeing, quality of life, and distress. Therefore, it is essential we first identify whether complex interventions currently being tested in people with M-LTCs are effective for this population and then explore which outcome measure is most sensitive to detecting a significant positive change in outcomes.

Aims:

1. To evaluate the evidence around the effectiveness of behaviour change interventions in people with M-LTCs.

For peer review only - http://bmjopen.bmj.com/site/about/guidelines.xhtml

2. To identify the outcome measures most sensitive to change, to identify effectiveness of complex behaviour change interventions in people with M-LTCs.

Methods:
This review was follows the PRISMA guidelines (supplementary table 1) and registered on PROSPERO (ID: CRD42021287847).

Search
The databases MEDLINE, Embase, PsychInfo, CINAHL and Web of Science were systematically searched from January 1999 to January 2022, and an example search term list can be seen in supplementary figure 1. This cutoff date was chosen, to align with the oldest dated paper identified in a previous Cochrane systematic review for complex interventions in people with M-LTCs (13) and to ensure that the findings were applicable to current healthcare settings. The identified articles were exported into Endnote and duplicates removed. 10% of identified titles and abstracts were independently screened by two reviewers (T.R. and D.N.) against the inclusion criteria, using Rayyan (16). As there was a less than 5% conflict (n=41/938; 4.37%), single screening was conducted by T.R. for the remaining titles and abstracts. For full text screening, 20% were independently reviewed by T.R. and D.N., and again, there was only a 5% discrepancy (n=2/40; 5%), and so the remainder were independently screened by T.R. for eligibility.

Inclusion Criteria
Participants: People with two or more physical and/or mental health conditions.
Intervention: A complex behaviour change intervention to help with the management of health conditions aiming to improve individual health outcomes, which could be targeted at the individual or organisational level.
Comparator: Treatment as usual.
Outcomes: Any individual health outcomes, such as quality of life, emotional wellbeing, clinical outcomes, psychological distress, behaviour change, and pain.
Setting: Interventions delivered in primary and community care settings.
Timeframe: published between 1st January 1999 and 17th January 2022.
Design: Randomised Controlled Trials

Data extraction and Outcome measures
Study characteristics were extracted by L.D. and checked by T.R. for completeness. Outcome measures were extracted by T.R. and checked by D.N. for completeness. For all studies, we extracted trial authors, year of publication, country, intervention description and length, study follow-up length, M-LTCs targeted, sample size at each timepoint, means and standard deviations for relevant outcome measures. Outcome measures were grouped into six categories, 1) quality of life, 2) clinical endpoints, 3) behaviour change, 4) pain, 5) wellbeing, and 6) psychological distress. Where data were not reported in the paper, we contacted the authors for further details (11 approached, 1 responded providing further data).

Study quality assessment
Trial result quality was assessed using the Risk of Bias 2 (RoB 2) tool (17). Initial assessment for 20% of eligible studies (n=12) was completed independently by two reviewers (T.R. and D.N.), with agreement for 8/12 trials (75%). Therefore, a further 10% (n=6) were independently reviewed, with 100% agreement between the two reviewers. The remaining 38 trials were assessed for quality by T.R. Study quality was used to inform narrative synthesis but was not used to exclude studies.

Patient and Public Involvement
Two public co-applicants living with M-LTCs were involved throughout the whole project. This included the review process, including refining the aims and inclusion criteria,

For peer review only

selecting the most important outcomes to focus on, analysing and providing feedback on findings, and in academic and lay dissemination of findings. They have been included as co-authors. A public engagement workshop, involving the two public co-applicants, and an additional six people with M-LTCs, was held to discuss relevant outcomes to focus on, effective ways to disseminate research findings with the public, and to develop future research questions.

Data analysis

Where there were 3 or more studies reporting the same outcome, we conducted a meta-analysis. RevMan 5.4 (18) was used to calculate, and pool mean differences or standardised mean differences and 95% confidence intervals, using inverse-variance random-effects models for all six continuous outcome categories. Psychological distress was separated into anxiety and depression, as many studies reported both outcomes and we felt the two outcomes differed. Random-effects model was selected due to the heterogenous populations and interventions present in this review, with differing conditions and intervention types being compared.

Interventions were grouped into three types, self-management, collaborative care, and cognitive and/or behavioural activation, based on their predominant features (table 1). Exploratory supplementary subgroup analyses were carried out to explore 1) Intervention type: self-management, collaborative care, and cognitive and/or behavioural activation), 2) Length of intervention: <3-months, 3-6-months, and 6+months, for outcomes immediately post-intervention delivery, 3) Maintenance of effect: Time since intervention was complete to final follow-up, grouped as <3 months, 3+months post intervention, and 4) Type of M-LTCs combinations: physical-physical, mental-physical and mental-mental.

Heterogeneity was assessed across studies using I^2 statistics, with proportions greater than 25%, 50%, and 75% considered to have low, moderate, and high heterogeneity, respectively (19). Studies for which data were not reported in a form that could be meta-analysed were synthesised narratively.

Table 1. Example interventions and their characteristics for allocation into the three types of intervention for supplementary subgroup analysis.

Intervention Type	Example of intervention and characteristics
Self-management	Psychoeducational interventions, with goal setting and problem solving, to help people manage the symptoms and improve their health outcomes related to their health conditions. Also included telemonitoring approaches, where participants received information and had their symptoms monitored over time.
Collaborative care	Interventions with behaviour change components which target the participants, the clinical team, and the system they are embedded within. Also included stepped care, where participants started with one treatment pathway and depending on progress, could be stepped up to more intensive treatment.
Cognitive and/or behavioural activation	Often use motivational interviewing, cognitive behavioural therapy, behavioural activation, and/or problem solving to empower people to help people understand the association between health conditions and cognitive processes and create healthier behaviours for future problems.

Results

For peer review only

After searching and de-duplication, 9,354 titles and abstracts were screened. 200 full text articles were screened and 53 included in the synthesis (see figure 1 and supplementary figure 2).

Study characteristics
Across 53 RCTs there were a total of 14,740 participants with study sizes ranging from 25 to 3324. The studies were published between 2001 and 2021, with a steady increase in publications from 2009 to the present day, with 58% published since 2017. Studies were conducted in the United States (n=22), Canada (n=7), Australia (n=5), UK (n=5), Netherlands (n=3), Spain (n=2), Ireland (n=2), and the remaining eight in Singapore, China, Nigeria, Germany, Indonesia, South Korea, Croatia, and India (one study was conducted across two countries). Interventions were aimed at people with a combination of physical and mental health conditions (n=27), two or more physical health conditions (n=25), and 1 study was for people with a combination of two mental health conditions. The health conditions included diabetes, depression, schizophrenia, coronary heart disease, chronic pain, HIV, and hypertension, as well as interventions which were open to people with multiple different health conditions.

The types of behaviour change interventions could be grouped into three categories: self-management (n=28), collaborative care (n=15), and cognitive and/or behavioural activation (n=10). Interventions were heterogeneous in terms of the person delivering them; they were delivered by individual or combinations of nurses, doctors, psychologists, other health-care professionals, and trained peer support leaders. The mode of delivery varied from face-to-face, in clinics or homes, telephone, and through digital platforms. The length of these interventions ranged from 3 weeks to 2 years, with most lasting between 3-12 months. Post-intervention, maintenance follow-up lengths varied from 6-weeks to 12-months. A summary of the studies and the interventions can be found in supplementary table 2.

Quality assessment
Overall trial result quality was very variable, 29 outcomes were rated as low risk of bias, 20 outcomes rated as having some concerns and four outcomes were assessed as having a high risk of bias. All the studies measuring quality of life and pain, half of the studies measuring clinical endpoints, 80% measuring functioning and 40% of those measuring psychological distress had at least some concerns with risk of bias (see supplementary table 2).

Outcome characteristics
For the primary outcome, 10 measured a clinical endpoint relevant to the included LTCs (e.g., HbA1c, blood pressure, cholesterol), 10 studies used a psychological distress measure, five used a behaviour change measure (e.g., alcohol use (AUDIT), five a functioning measure (e.g., physical functioning), four studies measured quality of life, one study measured pain, no studies used emotional wellbeing as the primary outcome, and six studies used a composite of these measures (often psychological distress combined with a clinical endpoint) and 12 studies used an outcome measure that did not fit into these categories e.g., acceptability of intervention.

For the secondary outcomes included in the meta-analyses, 11 studies measured functioning and psychological distress as an outcome, nine measure emotional wellbeing, seven measure quality of life, six behaviour change and four clinical endpoint outcomes.

Main analysis: Meta-analysis of individual outcomes
Outcome data assessed immediately post-intervention was available from 31 studies for meta-analysis across the seven outcome types. Effectiveness of interventions was detected when emotional wellbeing (SMD 0.31 [0.04, 0.58]) and psychological distress,

For peer review only

both depression (SMD -0.45 [-0.73, -0.16]) and anxiety (SMD -0.14 [-0.28, -0.00]), were measured. The effect sizes for psychological distress (depression) and emotional wellbeing (both post-intervention and maintenance) were moderate, but heterogeneity was high. For psychological distress (anxiety) the effect size was small and heterogeneity moderate. No other outcomes detected a significant effect from the interventions (see table 2; and supplementary figure 3 for forest plots).

Outcome data assessed at follow-up, to measure the effect of maintenance, was available from 15 studies for meta-analysis across all outcomes. Effectiveness of interventions was only detected when emotional wellbeing was measured (SMD 0.28 [0.07, 0.49]), with moderate heterogeneity. All other outcomes did not detect a significant effect (see table 2; and supplementary figure 3 for forest plots).

Table 2. Summary of pooled effects of outcome measures, for each outcome, assessed immediately post-intervention and at a follow-up/maintenance timepoint.

	**Overall effect**					
	**Post-intervention**			**Maintenance**		
**Outcome**	*n*	SMD [95% CI]	I^2	*n*	SMD [95% CI]	I^2
Quality of life	8	0.13 [-0.04, 0.30]	59%	3	0.61 [-1.54, 2.75]	99%
Clinical endpoints	11	-0.22 [-0.47, 0.03]	88%	5	-0.16 [-0.36, 0.04]	53%
Behaviour change	6	0.06 [-0.18, 0.29]	78%	3	0.02 [-0.10, 0.15]	23%
Pain	4	-0.32 [-0.70, 0.07]	77%	0	N/A	N/A
Functioning	14	0.05 [-0.06, 0.15]	42%	8	0.05 [-0.05, 0.16]	16%
Psychological distress (depression)	21	**-0.45 [-0.73, -0.16]**	95%	8	0.02 [-0.33, 0.36]	94%
Psychological distress (anxiety)	9	**-0.14 [-0.28, 0.00]**	40%	4	-0.08 [-0.18, 0.02]	0%
Emotional wellbeing	7	**0.31 [0.04, 0.58]**	85%	7	**0.28 [0.07, 0.49]**	72%

n is number of studies measuring the outcome. Positive results for psychological distress, indicated by a decrease in scores and for emotional wellbeing by an increase in scores.

Narrative synthesis:
For the 22 studies where meta-analysis was not possible due to data not being available, 15 measured at least one of the seven outcomes, and found results mostly consistent with the meta-analysis. Seven studies found positive intervention effects, which measured psychological distress (n=3), clinical endpoints (n=2), behaviour change (n=1), and functioning (n=1) (see supplementary table 3). Studies measuring quality of life (n=2) found no effects.

Supplementary analysis:
We conducted three supplementary analyses to explore outcomes by intervention type, duration of intervention and MLTC combination type:

1) *Intervention type*

As interventions were heterogeneous, a further supplementary meta-analysis was conducted, grouping interventions by type of approach.

For interventions with a self-management approach, none of the outcomes detected a significant benefit immediately post intervention and only emotional wellbeing at follow-up significantly improved (SMD 0.34 [0.03, 0.65]), but with high heterogeneity (see table 3; and supplementary figure 3 for forest plots).

For peer review only

For interventions with a collaborative care approach, significant differences post-intervention were detected for quality of life (SMD 0.15 [0.02, 0.27]), with low heterogeneity, emotional wellbeing (SMD 0.31 [0.11, 0.51]), and psychological distress (depression; SMD -0.82 [-1.40, -0.24]), with high heterogeneity, (table 3). When measuring maintenance effects, significant effects were found for emotional wellbeing (SMD 0.15 [0.00, 0.31]), with no heterogeneity, and psychological distress (depression; SMD -0.28 [-0.55, -0.01]), again with high heterogeneity.

For interventions with a cognitive and/or behavioural activation approach, post-intervention effects were seen for psychological distress (depression) only (SMD -0.24 [-0.38, -0.09]), with no heterogeneity. For maintenance effects, functioning was significantly improved (SMD 0.78 [0.05, 1.50]).

Table 3. Pooled effects of outcomes from meta-analysis, stratified by the three groups of complex behaviour change intervention types described in Table 1.

	Self-management			Collaborative care			Cognitive and/or behavioural activation		
**Post-intervention**	*n*	SMD [95% CI]	I^2	*n*	SMD [95% CI]	I^2	*n*	SMD [95% CI]	I^2
Quality of life	2	0.27 [-0.77, 1.30]	86%	4	**0.15 [0.02, 0.27]**	31%	2	0.14 [-0.69, 0.98]	72%
Clinical endpoints	3	-0.29 [-0.61, 0.02]	61%	4	-0.45 [-1.00, 0.11]	95%	4	0.06 [-0.10, 0.23]	0%
Behaviour change	3	0.01 [-0.60, 0.61]	91%	1	0.07 [-0.03, 0.17]	N/A	2	0.07 [-0.23, 0.38]	0%
Functioning	9	0.05 [-0.06, 0.16]	26%	3	0.08 [-0.20, 0.35]	74%	2	0.18 [-0.53, 0.89]	72%
Psychological distress (depression)	8	-0.30 [-0.91, 0.31]	96%	6	**-0.82 [-1.40, -0.24]**	97%	7	**-0.24 [-0.38, -0.09]**	0%
Psychological distress (anxiety)	5	-0.05 [-0.23, 0.14]	0%	2	-0.24 [-0.58, 0.10]	84%	2	-0.42 [-1.26, 0.42]	71%
Emotional wellbeing	5	0.33 [-0.10, 0.76]	90%	1	**0.31 [0.11, 0.51]**	N/A	1	0.22 [-0.06, 0.50]	N/A
**Maintenance**									
Clinical endpoints	1	-0.38 [-0.98, 0.21]	N/A	3	-0.18 [-0.48, 0.12]	74%	1	-0.11 [-0.40, 0.18]	N/A
Functioning	5	0.10 [-0.02, 0.22]	0%	2	-0.07 [-0.23, 0.08]	0%	1	**0.78 [0.05, 1.50]**	N/A
Psychological distress (depression)	4	0.28 [-0.48, 1.04]	96%	2	**-0.28 [-0.55, -0.01]**	82%	2	-0.16 [-0.59, 0.28]	71%
Emotional wellbeing	5	**0.34 [0.03, 0.65]**	76%	2	**0.15 [-0.00, 0.31]**	0%	0	N/A	N/A

n is number of studies measuring the outcome. *p* value is considered significant if ≤0.05.

In the 22 studies synthesised narratively, two of the nine self-management interventions (22%), and four of the six collaborative care interventions (67%) found significant effects on outcomes, three of which were psychological distress (depression) measures. These findings generally support the meta-analysis conclusions (see supplementary table 3).

For peer review only

2) *Duration of intervention*

For interventions lasting for less than 3-months, positive effects post-intervention were only detected with psychological distress (depression) measures (SMD -0.36 [-0.57, -0.14]), with very low heterogeneity. For behaviour change outcomes, a negative effect was found, with those in the control group doing better post-intervention than those receiving the intervention (SMD -0.22 [-0.41, -0.04]), with no heterogeneity. When exploring maintenance effects of interventions, clinical endpoints were the only outcome to detect a significant effect (SMD -0.60 [-0.99, -0.21]), with no heterogeneity.

Significant post-intervention effects for interventions lasting between 3- and 6-months were not found for any of the outcomes. However, significant differences at maintenance were detected when emotional wellbeing was measured (SMD 0.28 [0.02, 0.53]), but heterogeneity was high.

For interventions lasting for longer than 6-months, effects were seen post-intervention for clinical endpoints (SMD -0.15 [-0.28, -0.02]), with low heterogeneity, emotional wellbeing (SMD 0.28 [0.12, 0.44]), with no heterogeneity, and psychological distress (depression; -SMD 0.50 [-0.69, -0.30]) measures, with moderate heterogeneity, (see table 4). Maintenance effects in interventions lasting longer than 6-months were only identified with psychological distress (depression) measures (SMD -0.28 [-0.46, -0.10]), but heterogeneity was high (table 4; and supplementary figure 3 for forest plots).

Table 4. Pooled effects of outcomes, stratified by intervention length.

	<3-months			3-6months			>6months		
**Post-intervention**	*n*	SMD [95% CI]	I^2	*n*	SMD [95% CI]	I^2	*n*	SMD [95% CI]	I^2
Quality of life	3	0.38 [-0.38, 1.13]	81%	1	**0.13 [0.03, 0.24]**	N/A	4	0.11 [-0.13, 0.35]	55%
Clinical endpoints	0	N/A	N/A	4	-0.33 [-1.30, 0.65]	96%	7	**-0.15 [-0.28, -0.02]**	44%
Behaviour change	2	-0.22 [-0.41, -0.04]	0%	2	0.08 [-0.02, 0.17]	0%	2	0.34 [-0.20, 0.87]	83%
Functioning	1	-0.56 [-1.16, 0.04]	N/A	8	0.07 [-0.02, 0.16]	0%	5	0.07 [-0.15, 0.30]	65%
Psychological distress (depression)	6	**-0.36 [-0.57, -0.14]**	7%	10	-0.42 [-0.96, 0.12]	97%	**5**	**-0.50 [-0.69, -0.30]**	62%
Psychological distress (anxiety)	3	-0.20 [-0.65, 0.26]	50%	4	-0.09 [-0.19, 0.01]	0%	2	-0.17 [-0.75, 0.41]	79%
Emotional wellbeing	2	0.29 [-0.33, 0.91]	80%	3	0.34 [-0.28, 0.96]	90%	2	**0.28 [0.12, 0.44]**	0%
**Maintenance**									
Clinical endpoints	2	**-0.60 [-0.99, -0.21]**	0%	1	-0.11 [-0.40, 0.18]	N/A	2	-0.03 [-0.17, 0.11]	0%
Functioning	1	0.26 [-0.26, 0.79]	N/A	6	0.03 [-0.10, 0.17]	31%	1	0.11 [-0.10, 0.32]	N/A
Psychological distress (depression)	2	0.12 [-0.18, 0.42]	0%	3	0.26 [-0.67, 1.20]	96%	3	**-0.28 [-0.46, -0.10]**	94%

For peer review only

Emotional wellbeing	2	0.30 [-0.10, 0.69]	0%	5	**0.28 [0.02, 0.53]**	81%	0	N/A	N/A

n is number of studies measuring the outcome. *p* value is considered significant if ≤0.05.

Exploring the 22 studies without data for meta-analysis, none of the 3 studies lasting less than 3-months, two of the five studies lasting between 3- and 6-months (40%), and four out of the seven studies lasting longer than 6 months (57%) had significant primary outcome effects (see supplementary table 3).

3) *M-LTCs combination type*

Analysis around effectiveness of interventions depending on the combination of M-LTCs types, grouped as physical-physical, physical-mental, and mental-mental was explored post-hoc, following conversations about potential impact with public contributors. The only significant effect found was post-intervention for psychological distress (depression) measures in people with combined physical and mental health conditions (SMD -0.58 [-0.94, -0.21]), but heterogeneity was high (see supplementary table 4; and supplementary figure 3 for forest plots).

In the narrative synthesis, of those measuring a relevant outcome, three out of nine studies measuring interventions targeted at people with two physical health conditions found positive effects (33%), two of which were measuring clinical endpoints. Of those measuring effects in people with a physical and mental health condition, three out of six studies found positive effects (50%), and these were all measuring psychological distress (depression) (see supplementary table 3).

Discussion

Across the 53 included studies, types of complex interventions, LTCs targeted, and outcome measures used varied widely. Overall, there is evidence that complex interventions can improve the outcomes of people with M-LTCs, and the outcome measures most sensitive to change were emotional wellbeing and psychological distress.

In subgroup analyses, interventions that lasted for longer periods of time, and had a collaborative care approach were associated with greater beneficial outcomes than shorter interventions with a self-management focus. Interventions with a cognitive and/or behavioural activation approach and interventions targeted at people with a combination of a mental and physical health condition had better outcomes when measuring psychological distress (depression) measures.

Results in context

Developing interventions for people with single LTCs centred around psychological variables is not a new concept, with psychological distress and emotional wellbeing factors being the most amenable to change (21). This is particularly important when exploring the psychological factors, such as psychological capability, that enable a person with a health condition to believe they can engage in the necessary behaviour to improve their outcomes (22). In the absence of psychological capability, an intervention targeting this, through goal setting, problem solving, and self-efficacy, could improve wellbeing outcomes (23). These concepts for people with single LTCs appear to transfer over to those with M-LTCs, with psychological distress measures showing a significant improvement in those with a combination of physical and mental health conditions. This is perhaps unsurprising, given the likely higher psychological burden placed on those living with M-LTCs.

In people with single mental LTCs, cognitive and/or behavioural activation centred interventions have a strong evidence base from systematic reviews for reducing peoples' psychological distress and depression symptoms (24, 25). The findings from this review suggest these interventions have similar improvements for psychological distress in people

For peer review only

with M-LTCs. Despite this, often quality of life is chosen as the primary outcome to test the effectiveness of complex interventions, when single clinical outcomes cannot be used. However, here we have shown that emotional wellbeing or psychological distress may be the outcomes more able to detect a change in people with M-LTCs. We have further explored the intervention components and how they link to intervention effectiveness in a linked paper (26).

Looking across the outcomes, the most effective intervention type for people with M-LTCs appears to be collaborative care. Collaborative care involves integrating health and care services across specialties and services to improve the outcomes of the people they are caring for (27). Other reviews have found positive effects of collaborative care interventions for people with a combination of at least one physical LTC and depression (27, 28). The mechanisms that underpin this change are thought to be two-fold, at the individual level, with the person being able to manage their physical LTC better once the depressive symptoms have been reduced, and at the institutional level, with improved delivery of care with a patient centred focus (27).

Alongside the type of intervention, the length of the intervention also seems to be an important factor for effectiveness, with interventions lasting for longer than 6-months improving outcomes post-intervention. In line with guidance, when designing an intervention, the minimum clinically effective dose should be identified and empirically tested (8, 29). There is no evidence suggesting that length and effectiveness of interventions are linked in people with single LTCs. However, it may be that the complex and interacting needs of people with M-LTCs require longer interventions to ensure that the skills to self-manage and the systems to support the individual are in place (12). Also, it is worth noting that even with interventions lasting for longer than 6-months, maintenance effects are only seen for psychological distress measures, highlighting again that these outcomes are the most amenable to change (21) and that if we want other outcomes to continue to be improved, then support beyond the intervention period is probably needed.

Alongside these findings, it is worth highlighting the lack of studies exploring some of the outcomes of interest, including pain, clinical endpoints, and behaviour change. Therefore, the lack of significant effects for these may be due to the lack of studies measuring them as an outcome. This is even more apparent when looking at the maintenance data.

Future Research

The findings presented here highlight the intervention type and length that are associated with improved outcomes in people with M-LTCs. Therefore, future behaviour change interventions should explore a collaborative care approach and last for longer than 6-months. In addition to this, whilst post-intervention outcomes are effective in those lasting more than 6-months, findings around maintenance effects are less conclusive. This suggests more research is needed to determine how to ensure effective interventions have long lasting effects and improvements can be maintained over time once intervention periods have ended.

In addition, whilst the findings here suggest that interventions to improve psychological distress and emotional wellbeing outcomes in those with mental LTCs have been successfully applied to those with mental and physical LTCs, the results for those with two or more physical LTCs are less conclusive. Future research needs to explore why this might be, and whether different approaches are needed, with specific intervention components, to address the needs of people with multiple physical LTCs, alongside identifying which outcomes are most appropriate to measure this mechanism of action (30).

For peer review only

Limitations
Double screening of eligible papers was only completed for a percentage of the identified studies, meaning some eligible papers could have been missed. However, through piloting good consensus was found and the lead reviewer (T.R.) was most familiar with the eligibility criteria, the papers of interest, and the process of screening.

Complex interventions are difficult to define and therefore, determining whether a study was eligible based on this criterion can be a challenge. To overcome this potential bias, if there was any doubt, studies were included and discussed with a second (D.N.) and sometimes a third (R.F.) reviewer to establish consensus about eligibility, to make this process as consistent as possible.

Many of the meta-analyses results had high heterogeneity, suggesting that they may not have been comparable. A random-effects model was used for all analyses, but the results should be viewed with this in mind.

Searches were conducted over a year prior to submission. A full updated search was not possible, due to team resources and funding. The search was rerun in Medline and an additional 356 papers were identified and a brief screen found one paper which may have been eligible (31). This intervention focussed on behaviour change in people with depression and type 2 diabetes and found positive outcomes for clinical outcomes and psychological outcomes, which confirm the conclusions of this review and meta-analysis.

Conclusions
In conclusion, effective complex interventions to improve the outcomes of people with M-LTCs are possible. For those with a combination of physical and mental LTCs, a collaborative care or a cognitive and/or behavioural activation approach should be considered, and emotional wellbeing and psychological distress outcomes should be measured to detect effectiveness. Evidence around those with multiple physical LTCs is less clear, however a collaborative care approach is likely the most appropriate, but the intervention components and outcome to measure this still need to be identified to ensure the best outcomes for people with M-LTCs.

Key Words
Multimorbidity, multiple long-term conditions, randomised controlled trial, meta-analysis, systematic review, behaviour change.

Word Count 4136

Conflicts of interests
The authors have no conflicts of interest to declare.

Author Statement
TAR: Principal Investigator for the study, developed the protocol, conducted the searches, screened all the abstracts and full texts, completed outcome measure data extraction, conducted meta-analysis and narrative synthesis, wrote and finalised the manuscript.
DN: Co-investigator for the study, second reviewer for screening and quality assessment, supported with analysis and interpretation and reviewed the final manuscript.
RF: Supervised TR through the process, supported with development of project and protocol, acted as third reviewer when consensus could not be agreed, supported with interpretation of meta-analysis, writing up the results, and reviewed the final manuscript.
MA: Supported with development of project and protocol and with the interpretation of findings and reviewed the final manuscript.
LD: Completed data extraction for all studies included and reviewed the final manuscript.
JR: Supported with development of project and protocol and reviewed the final manuscript.

For peer review only

JH: Public contributor assisting with project design, finalising the research questions and identifying outcome measures of importance.
MM: Public contributor assisting with project design, finalising the research questions and identifying outcome measures of importance.
SJCT: Supported with development of project and protocol and reviewed the final manuscript.
KW: Supervised TR through the process, supported with development of project, protocol, and analysis plan and reviewed the final manuscript.

Ethical approval
Ethical approval was not obtained for this study, as no participants were directly involved and consented.

Data Statement
All data is available by reasonable request from the corresponding author.

Funding
This study/project is funded by the National Institute for Health and Care Research (NIHR) School for Primary Care Research (572). The views expressed are those of the author(s) and not necessarily those of the NIHR or the Department of Health and Social Care.

References
1. NICE: National Institute for Health and Care Excellence. https://cks.nice.org.uk/topics/multimorbidity/background-information/prevalence/. 2023. Multimorbidity: How common is it?
2. Kingston A, Robinson L, Booth H, Knapp M, Jagger C, project M. Projections of multi-morbidity in the older population in England to 2035: estimates from the Population Ageing and Care Simulation (PACSim) model. Age Ageing. 2018;47(3):374-80.
3. Nunes BP, Flores TR, Mielke GI, Thume E, Facchini LA. Multimorbidity and mortality in older adults: A systematic review and meta-analysis. Arch Gerontol Geriatr. 2016;67:130-8.
4. Williams JS, Egede LE. The Association Between Multimorbidity and Quality of Life, Health Status and Functional Disability. Am J Med Sci. 2016;352(1):45-52.
5. Soley-Bori M, Ashworth M, Bisquera A, Dodhia H, Lynch R, Wang Y, et al. Impact of multimorbidity on healthcare costs and utilisation: a systematic review of the UK literature. Br J Gen Pract. 2021;71(702):e39-e46.
6. Afshar S, Roderick PJ, Kowal P, Dimitrov BD, Hill AG. Multimorbidity and the inequalities of global ageing: a cross-sectional study of 28 countries using the World Health Surveys. BMC Public Health. 2015;15:776.
7. Salisbury C, Johnson L, Purdy S, Valderas JM, Montgomery AA. Epidemiology and impact of multimorbidity in primary care: a retrospective cohort study. Br J Gen Pract. 2011;61(582):e12-21.
8. Skivington K, Matthews L, Simpson SA, Craig P, Baird J, Blazeby JM, et al. A new framework for developing and evaluating complex interventions: update of Medical Research Council guidance. BMJ. 2021;374:n2061.
9. Harrison M, Reeves D, Harkness E, Valderas J, Kennedy A, Rogers A, et al. A secondary analysis of the moderating effects of depression and multimorbidity on the effectiveness of a chronic disease self-management programme. Patient Educ Couns. 2012;87(1):67-73.
10. Zora S, Custodero C, Pers YM, Valsecchi V, Cella A, Ferri A, et al. Impact of the chronic disease self-management program (CDSMP) on self-perceived frailty condition: the EU-EFFICHRONIC project. Ther Adv Chronic Dis. 2021;12:20406223211056722.
11. Bricca A, Jäger M, Johnston M, Zangger G, Harris LK, Midtgaard J, Skou ST. Effect of in-person delivered behavioural interventions in people with multimorbidity: systematic

For peer review only

review and meta-analysis. International Journal of Behavioral Medicine. 2023 Apr;30(2):167-89.
12. Smith SM, Soubhi H, Fortin M, Hudon C, O'Dowd T. Managing patients with multimorbidity: systematic review of interventions in primary care and community settings. BMJ. 2012;345:e5205.
13. Smith SM, Wallace E, O'Dowd T, Fortin M. Interventions for improving outcomes in patients with multimorbidity in primary care and community settings. Cochrane Database Syst Rev. 2016;3(3):CD006560.
14. Bratzke LC, Muehrer RJ, Kehl KA, Lee KS, Ward EC, Kwekkeboom KL. Self-management priority setting and decision-making in adults with multimorbidity: a narrative review of literature. Int J Nurs Stud. 2015;52(3):744-55.
15. Smith SM, Wallace E, Clyne B, Boland F, Fortin M. Interventions for improving outcomes in patients with multimorbidity in primary care and community setting: a systematic review. Syst Rev. 2021;10(1):271.
16. Ouzzani M, Hammady H, Fedorowicz Z, Elmagarmid A. Rayyan-a web and mobile app for systematic reviews. Syst Rev. 2016;5(1):210.
17. Sterne JA-O, Savović J, Page MJ, Elbers RG, Blencowe NS, Boutron I, et al. RoB 2: a revised tool for assessing risk of bias in randomised trials. (1756-1833 (Electronic)).
18. Review Manager Web (RevMan Web). Available at revman.cochrane.org: The Cochrane Collaboration; 2020.
19. Higgins JP, Thompson Sg Fau - Deeks JJ, Deeks Jj Fau - Altman DG, Altman DG. Measuring inconsistency in meta-analyses. (1756-1833 (Electronic)).
20. Page MJ, McKenzie JE, Bossuyt PM, Boutron I, Hoffmann TC, Mulrow CD, et al. The PRISMA 2020 statement: an updated guideline for reporting systematic reviews. BMJ. 2021;372.
21. Heath G, Cooke R, Cameron E. A Theory-Based Approach for Developing Interventions to Change Patient Behaviours: A Medication Adherence Example from Paediatric Secondary Care. Healthcare (Basel). 2015;3(4):1228-42.
22. Michie S, van Stralen MM, West R. The behaviour change wheel: a new method for characterising and designing behaviour change interventions. Implement Sci. 2011;6:42.
23. Oades LG, Ozturk C, Hou H, Slemp GR. Wellbeing literacy: A language-use capability relevant to wellbeing outcomes of positive psychology intervention. The Journal of Positive Psychology. 2020;15(5):696-700.
24. Huguet A, Rao S, McGrath PJ, Wozney L, Wheaton M, Conrod J, et al. A Systematic Review of Cognitive Behavioral Therapy and Behavioral Activation Apps for Depression. PLoS One. 2016;11(5):e0154248.
25. Orgeta V, Brede J, Livingston G. Behavioural activation for depression in older people: systematic review and meta-analysis. Br J Psychiatry. 2017;211(5):274-9.
26. Rookes TA, Nimmons D, Frost R, Armstrong M, Davies L, Ross, et al. Identifying the active components through the behaviour change techniques taxonomy in complex interventions for people with multiple long-term health conditions (M-LTCs): a systematic review. [Under review]. 2024.
27. Coventry P, Lovell K, Dickens C, Bower P, Chew-Graham C, McElvenny D, et al. Integrated primary care for patients with mental and physical multimorbidity: cluster randomised controlled trial of collaborative care for patients with depression comorbid with diabetes or cardiovascular disease. BMJ. 2015;350:h638.
28. Kappelin C, Carlsson AC, Wachtler C. Specific content for collaborative care: a systematic review of collaborative care interventions for patients with multimorbidity involving depression and/or anxiety in primary care. Fam Pract. 2022;39(4):725-34.
29. Hoffmann TC, Glasziou PP, Boutron I, Milne R, Perera R, Moher D, et al. Better reporting of interventions: template for intervention description and replication (TIDieR) checklist and guide. BMJ. 2014;348:g1687.
30. Smith SM, Bayliss EA, Mercer SW, Gunn J, Vestergaard M, Wyke S, et al. How to Design and Evaluate Interventions to Improve Outcomes for Patients with Multimorbidity. Journal of Comorbidity. 2013;3(1):10-7.

For peer review only

31. Hoyo ML, Rodrigo MT, Urcola-Pardo F, Monreal-Bartolomé A, Ruiz DC, Borao MG, et al. The TELE-DD Randomised Controlled Trial on Treatment Adherence in Patients with Type 2 Diabetes and Comorbid Depression: Clinical Outcomes after 18-Month Follow-Up. International Journal of Environmental Research and Public Health. 2022;20(1):328.

For peer review only

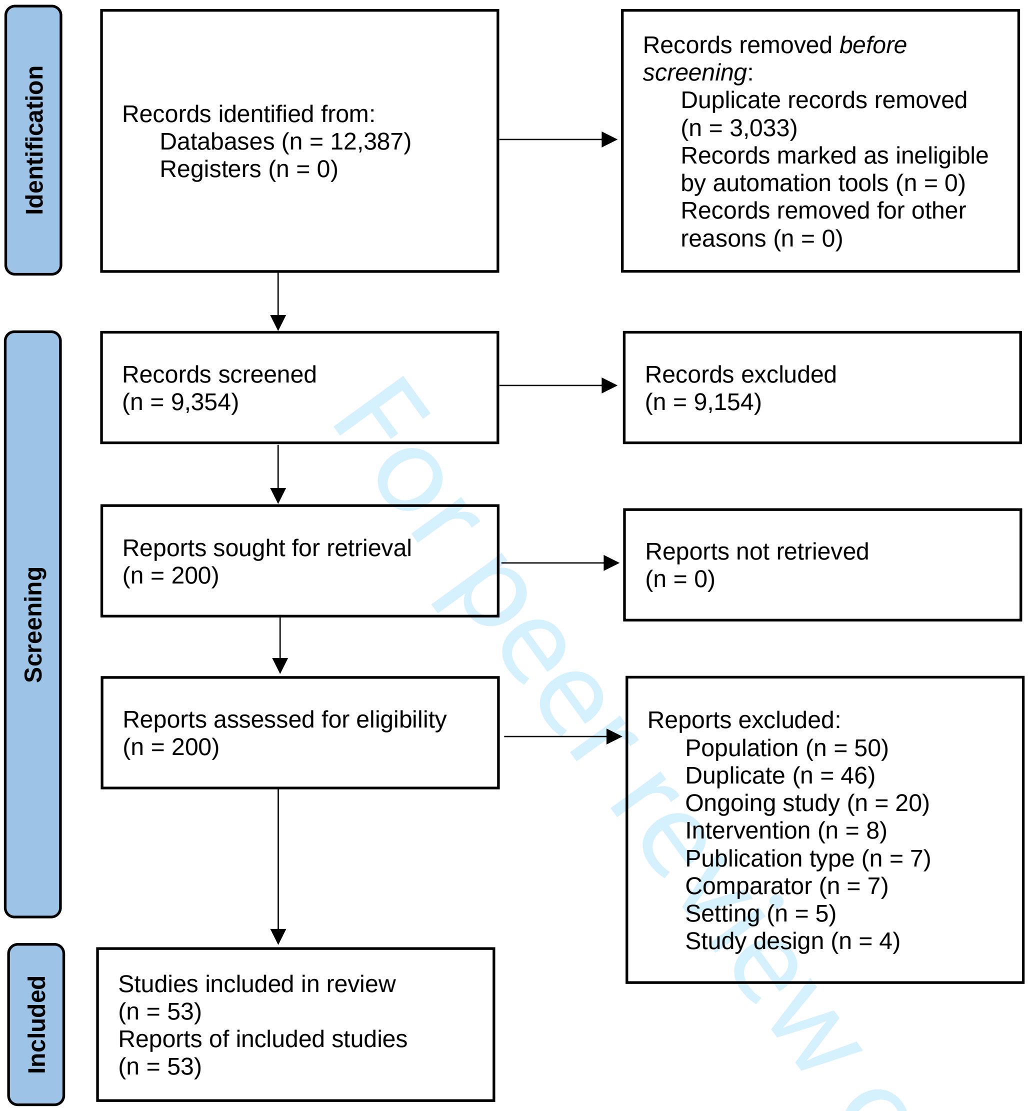

Figure 1. Prisma flow chart. (20)

For peer review only

PRISMA 2020 Checklist

Section and Topic	Item #	Checklist item	Location where item is reported
**TITLE**			
Title	1	Identify the report as a systematic review.	Page 1
**ABSTRACT**			
Abstract	2	See the PRISMA 2020 for Abstracts checklist.	Page 1
**INTRODUCTION**			
Rationale	3	Describe the rationale for the review in the context of existing knowledge.	Page 1 and 2
Objectives	4	Provide an explicit statement of the objective(s) or question(s) the review addresses.	Page 2
**METHODS**			
Eligibility criteria	5	Specify the inclusion and exclusion criteria for the review and how studies were grouped for the syntheses.	Page 3
Information sources	6	Specify all databases, registers, websites, organisations, reference lists and other sources searched or consulted to identify studies. Specify the date when each source was last searched or consulted.	Page 2 and 3
Search strategy	7	Present the full search strategies for all databases, registers and websites, including any filters and limits used.	Supplementary figure 1
Selection process	8	Specify the methods used to decide whether a study met the inclusion criteria of the review, including how many reviewers screened each record and each report retrieved, whether they worked independently, and if applicable, details of automation tools used in the process.	Page 2 and 3
Data collection process	9	Specify the methods used to collect data from reports, including how many reviewers collected data from each report, whether they worked independently, any processes for obtaining or confirming data from study investigators, and if applicable, details of automation tools used in the process.	Page 3
Data items	10a	List and define all outcomes for which data were sought. Specify whether all results that were compatible with each outcome domain in each study were sought (e.g. for all measures, time points, analyses), and if not, the methods used to decide which results to collect.	Page 3
	10b	List and define all other variables for which data were sought (e.g. participant and intervention characteristics, funding sources). Describe any assumptions made about any missing or unclear information.	Page 3
Study risk of bias assessment	11	Specify the methods used to assess risk of bias in the included studies, including details of the tool(s) used, how many reviewers assessed each study and whether they worked independently, and if applicable, details of automation tools used in the process.	Page 3
Effect measures	12	Specify for each outcome the effect measure(s) (e.g. risk ratio, mean difference) used in the synthesis or presentation of results.	Page 3
Synthesis methods	13a	Describe the processes used to decide which studies were eligible for each synthesis (e.g. tabulating the study intervention characteristics and comparing against the planned groups for each synthesis (item #5)).	Page 3
	13b	Describe any methods required to prepare the data for presentation or synthesis, such as handling of missing summary statistics, or data conversions.	Page 3
	13c	Describe any methods used to tabulate or visually display results of individual studies and syntheses.	Supplementary table 2
	13d	Describe any methods used to synthesize results and provide a rationale for the choice(s). If meta-analysis was performed, describe the model(s), method(s) to identify the presence and extent of statistical heterogeneity, and software package(s) used.	Page 3
	13e	Describe any methods used to explore possible causes of heterogeneity among study results (e.g. subgroup analysis, meta-regression).	Page 3
	13f	Describe any sensitivity analyses conducted to assess robustness of the synthesized results.	N/A
Reporting bias assessment	14	Describe any methods used to assess risk of bias due to missing results in a synthesis (arising from reporting biases).	Page 3

For peer review only - http://bmjopen.bmj.com/site/about/guidelines.xhtml

PRISMA 2020 Checklist

Section and Topic	Item #	Checklist item	Location where item is reported
Certainty assessment	15	Describe any methods used to assess certainty (or confidence) in the body of evidence for an outcome.	Page 10
**RESULTS**			
Study selection	16a	Describe the results of the search and selection process, from the number of records identified in the search to the number of studies included in the review, ideally using a flow diagram.	Page 4
	16b	Cite studies that might appear to meet the inclusion criteria, but which were excluded, and explain why they were excluded.	Page 4
Study characteristics	17	Cite each included study and present its characteristics.	Supplementary figure 2 and supplementary table 2
Risk of bias in studies	18	Present assessments of risk of bias for each included study.	Supplementary table 2
Results of individual studies	19	For all outcomes, present, for each study: (a) summary statistics for each group (where appropriate) and (b) an effect estimate and its precision (e.g. confidence/credible interval), ideally using structured tables or plots.	Tables 2, 3, 4 and supplementary table 4
Results of syntheses	20a	For each synthesis, briefly summarise the characteristics and risk of bias among contributing studies.	Page 5
	20b	Present results of all statistical syntheses conducted. If meta-analysis was done, present for each the summary estimate and its precision (e.g. confidence/credible interval) and measures of statistical heterogeneity. If comparing groups, describe the direction of the effect.	Tables 2, 3, 4 and supplementary table 4
	20c	Present results of all investigations of possible causes of heterogeneity among study results.	Page 7, 8, 9
	20d	Present results of all sensitivity analyses conducted to assess the robustness of the synthesized results.	N/A
Reporting biases	21	Present assessments of risk of bias due to missing results (arising from reporting biases) for each synthesis assessed.	Page 7, 8, 9
Certainty of evidence	22	Present assessments of certainty (or confidence) in the body of evidence for each outcome assessed.	Page 10
**DISCUSSION**			
Discussion	23a	Provide a general interpretation of the results in the context of other evidence.	Page 9 and 10
	23b	Discuss any limitations of the evidence included in the review.	Page 10
	23c	Discuss any limitations of the review processes used.	Page 10
	23d	Discuss implications of the results for practice, policy, and future research.	Page 10
**OTHER INFORMATION**			
Registration and protocol	24a	Provide registration information for the review, including register name and registration number, or state that the review was not registered.	Page 2
	24b	Indicate where the review protocol can be accessed, or state that a protocol was not prepared.	Page 2
	24c	Describe and explain any amendments to information provided at registration or in the protocol.	N/A
Support	25	Describe sources of financial or non-financial support for the review, and the role of the funders or sponsors in the review.	Page 11
Competing	26	Declare any competing interests of review authors.	Page 11

For peer review only - http://bmjopen.bmj.com/site/about/guidelines.xhtml

For peer review only

PRISMA 2020 Checklist

Section and Topic	Item #	Checklist item	Location where item is reported
interests			
Availability of data, code and other materials	27	Report which of the following are publicly available and where they can be found: template data collection forms; data extracted from included studies; data used for all analyses; analytic code; any other materials used in the review.	Page 11

From: Page MJ, McKenzie JE, Bossuyt PM, Boutron I, Hoffmann TC, Mulrow CD, et al. The PRISMA 2020 statement: an updated guideline for reporting systematic reviews. BMJ 2021;372:n71. doi: 10.1136/bmj.n71

For more information, visit: http://www.prisma-statement.org/

For peer review only

Lead Author<br>Year<br>Country	Participant condition 1<br>Participant condition 2<br>Other conditions	LTC combination	Study n<br>Intervention n<br>Control n<br>Carers n	Intervention length<br>Follow-up timepoints	Primary outcome<br>Timepoint<br>Effectiveness	Category	Quality assessment (RoB-2)	Intervention name, description and modifications	Intervention components<br>Leader<br>Recipient<br>One-to-one or Group<br>Mode of delivery and location<br>Number/length sessions
Ali<br>2020<br>India	Depression<br>Type 2 diabetes	Physical-Mental	404<br>196<br>208	12 months<br>Baseline, 6, 12, 18, 24 months	Composite depression and clinical<br>24 months<br>Yes	Collaborative care	Low	Collaborative care intervention targetting patient-, clinician-, and system-level improvements. Administered PHQ-9 to monitor depression, reviewed glucose logs, and counseling toward achieving treatment goals. Intergrated decision making tool for HCPs within electronic health record system	Psychiatrist and diabetologist and care co-ordinators<br>Person with depression and T2DM<br>One-to-one<br>Face-to-face in clinic or telephone<br>Every 2-4 weeks for 12 months

For peer review only

Aragones 2019 Spain	Depression and Chronic Musculoskeletal Pain	Physical-Mental	41<br>21<br>20		3 months<br>Baseline, 3, 6, 12 months.	Depression<br>12 months<br>Yes	Self-management	Low	DROP (DepRessiOn and Pain) programme:<br>1. Optimised management of major depression through computerised clinical guideline to aid decision making<br>2. Care management of patient pain through calls with psychologist<br>3. Psychoeducational intervention group programme, with discussions and homework covering: understanding pain; managing emotions and attention; basic relaxation techniques; cognitive restructuring strategies; problem-solving; setting life goals; relationships between pain and physical activity; healthy posture and sleep; maintenance of the strategies learned; and a relapse plan	Care co-ordinator (psychologist)<br>Primary care physicians and people with conditions<br>Primary Care Centres<br>Face-to-face - 9 weekly 2 hour group sessions<br>Telephone - Monthly for first 3 months and then every 3 months for next 9 months

For peer review only

Barley, 2014, UK	Coronary heart disease<br>Depression	Physical-Mental	81<br>41<br>40	6 months. Baseline, 1, 3, 6, 12 months.	N/A Feasibility and acceptability	Self-management	Low	Personalised Care: the nurse case manager conducts a standardised, face to face, biopsychosocial assessment. Patients then identify up to three problems which contribute to their depression and they want to address. The nurse-case managers provide information, sign-post to existing resources and use evidence based behaviour change techniques to help patients set and achieve goals. The underlying intention of the intervention is to increase the patient's self-efficacy to achieve their desired goals. The action plan is recorded in a 'personalised health plan' which the patient holds.	Nurse<br>Person with CHD and depression<br>One-to-one<br>Face-to-face and telephone<br>1 hour session<br>Weekly 15 minute phone calls for 6 months
Barrowclough, 2001, UK	Schizophrenia<br>Substance Abuse disorder	Mental-Mental	33 patient carer dyads	9 months. Baseline, 9 months and 12 months.	Functioning 9 and 12 months<br>Yes	Cognitive and/or Behavioural Activation	Some concerns	Allocated a family support worker from Making Space, providing information, giving advice on benefits, advocacy, emotional support, and practical help. The integrated treatment program attempted to combine three treatment approaches: motivational interviewing, individual cognitive behavior therapy, and family or caregiver intervention.	Family support worker<br>Patient and primary care giver dyad<br>Face-to-face<br>Home or clinic<br>23 weekly sessions followed by 6 biweekly sessions and 10-16 family sessions

For peer review only

Barr Taylor, 2003, USA	Diabetes<br>And one or more of:<br>Hypertension<br>CVD<br>Dyslipidemia	Physical-Physical	169<br>84<br>85	12 months<br>Baseline, 12 months	Clincal outcome (HbA1c)<br>12 months<br>Yes	Collaborative care	Low	Nurse care management system. Initial consultation to review medical, lifestyle, and social status, and develop a self-management action plan. Group classes followed a workbook specifically created for the program and included some didactic material, although the focus was on group discussion, participation and problem-solving. Follow-up calls were used to review patient goals, medication use, glucose/BP monitoring, and self-management activities. Higher levels of depression and alcohol problems were also addressed. Algorithims were used throughout for medication managament.	Nurse care manager<br>Patient<br>Group classes once a week for 4 weeks (1-2 hours)<br>8 Follow-up telephone calls lasted 15 minutes, over 9 months.

For peer review only

Battersby, 2013, Australia	Alcohol use disorder AND one or more of:<br>-Cardiovascular<br>-Musculoskeletal<br>-Gastrointestinal<br>-Respiratory<br>-Diabetes<br>-Skin conditions<br>-Cancer<br>-Genitourinary<br>-Alcohol dependence<br>-PTSD<br>-Major depression<br>-Generalised Anxiety Disorder<br>-Panic Disorder	Physical-Mental	77<br>46<br>31<br>SCDSMP course = 22 veterans and 15 of their partners from intervention group	9-month intervention. Seen at 1, 3, 6 and 9 months, with telephone calls at 4.5 and 7.5 months<br>Follow-up measures at 18 months	Behaviour change (AUDIT)<br>9 months<br>Yes	Self-management	High	Flinders Programme<br>Targeted self-management support to assist the patient to achieve improved health outcomes over a period of 12 months. A health professional is trained to tailor the interventions to meet the persons needs whilst simultaneously motivating the patient to achieve their medical, psychosocial, or lifestyle risk factor goals. Veterans offered SCDSMP course (Stanford School of Medicine) if considered appropriate - this builds the person's self-efficacy by teaching skills in problem-solving, decision-making, resource utilisation, managing the patient–provider partnership, action planning and emotional management	Research nurse (RN)<br>Veterans<br>One-to-one<br>Face-to-face: begins with developing care plan, follow-up visits monitor outcome of this plan<br>SCDSMP: 6 weekly face-to-face group sessions (2.5 hours each) delivered jointly by research officer and veteran peer leader to veterans and their partners if desired

For peer review only

Blank 2011, USA	HIV<br>Schizophrenia Spectrum Disorder/ Affective Disorder/ Other serious mental ilness	Physical-Mental	238<br>128<br>110	12 months. Baseline, 3 months, 6 months, 12 months<br>Follow-up at 24 months.	Clinical (Viral load)<br>12 months<br>No	Self-management	Some concerns	Preventing Aids Through Health (PATH)<br>Driven by theory of reasoned action; helps patients actively cope with barriers to medication adherence and promote participants self-care abilities. The protocol includes one face-to-face meeting a week as a minimum with basic intervention (psychoeducation, pillboxes, beeping watches). The intervention was then cascaded to suit the needs of the individual if adherence fell - this was a gradual increase in intensity and included activation of social networks, followed by use of beepers with alphanumeric displays, then prepaid cellular phones to encourage participants to follow their regimen, then the final step was directly observed therapy.	Advanced Practice Nurse (same nurse each session where possible)<br>Patient<br>One-to-one<br>Community-based: weekly face-to-face home visits from the APN
Boeschoten, 2017, The Netherlands	MS<br>Depression<br>Anxiety	Physical-Mental	171<br>85<br>86	5 to 10 weeks. Baseline, within a week of intervention, 4 months	Depression (BDI-II)<br>5-10 weeks and 4 months<br>No	Cognitive and/or Behavioural Activation	Low	"Minder Zorgen" (Worry Less) - a guided self help intervention using Cognitive Behavioural Therapy with a focus on coping skills. Five modules with texts, examples and assignments that patients could access online from their own computer. This intervention was an existing IPST and adapated for MS patients.	Psychologists and masters student<br>Patient<br>One-to-one<br>Online - digital<br>One module a week for 10 weeks

For peer review only

Bogner, 2008, USA	Hypertension Depression	Physical-Mental	64 32 32	4 weeks. Baseline, 2, 4 and 6 weeks.	Depression (CES-D) 6 weeks Yes	Collaborative care	Low	Integrated Care Intervention. Involved education about depression and hypertension, emphasizing the importance of controlling depression to manage hypertension; encouragement and relief from stigma; helped to identify target symptoms for both conditions; explained the rationale for antidepressant and antihypertensive medication usage; assessed for side-effects and assisted in their management; assessed progress; assisted with referrals; and monitored and responded to life-threatening symptoms.	Integrated care manager (ICM) Patient One-to-one 3, 30-minute in-person sessions and 2, 15-minute telephone-monitoring contacts during the 4 weeks

For peer review only

Chwastiak, 2018, USA	Type II diabetes<br>Schizophrenia<br>Schizoaffective disorder<br>Bipolar disorder<br>Major depressive disorder with psychosis	Physical-Mental	35<br>18<br>17	3 months. Baseline, 3 months.	Clinical (HbA1c)<br>3 months<br>N/A test not completed	Collaborative care	Low	CMHC-based collaborative care model, adapted from the primary care–based TEAMcare model and adapted for patients with psychosis.<br>Initial 60-min nurse care manager visit for a comprehensive health assessment and an individualized health plan and then 30-minute visits for the support of chronic illness self-management (including medication adherence,<br>healthy nutrition, and regular physical activity) every other week for 12 weeks. Nurses used motivational interviewing and behavioural activation to address barriers to self management.<br>Diabetic education materials were modified to address needs of patients with psychosis.	A CMHC nurse care manager, a CMHC psychiatrist, the advanced practice registered nurse who provided primary care onsite at the CMHC, and an endocrinologist consultant.<br>Patients<br>One-on-one<br>Hospital clinic

For peer review only

Cummings, 2019, USA	Type 2 diabetes<br>Diabetes-related distress or depression	Physical-Mental	139<br>67 (CBT=48; Lifestyle=19)<br>72	12 months. Baseline, 6 months (to adjust intervention if necessary), 12 months.	Clinical (HbA1c)<br>12 months<br>No (approached p=0.06)	Cognitive and/or Behavioural Activation	Low	CBT (plus usual care): Session content used cognitive techniques to identify and challenge general and diabetes-specific cognitive distortions that result in maladaptive behavior, in combination with behavioral techniques, including behavioral activation and specific behavior change strategies related to diabetes and/or mood. Those with more intermediate concerns were also provided with problem solving therapy focusing on effective coping and adaptive problem-solving skills.<br>Small Changes Lifestyle Coaching (plus usual care): Focused on lifestyle modifications to improve diabetes and mood.<br>Patients were reevaluated at 6 months and either entered a maintenance phase, or switched to CBT or SCLC.	SCLC - nurse care manager; CBT - a psychologist and clinical health psychology doctoral student; and a community health worker provided navigation and social support.<br>Patient.<br>One-on-one.<br>12 tailored sessions 30-60 minutes long for the first 6 months of either:<br>CBT: Face-to-face or over the telephone if necessary.<br>Lifestyle: over telephone

For peer review only

Druss, 2018, USA	Medical illness<br>Diabetes<br>Heart disease<br>Hyperlipidemia<br>Hypertension<br>Arthritis<br>Hepatitis<br>Asthma/COPD<br>HIV<br>SMI<br>Schizophrenia/ schizoaffective<br>Bipolar disorder<br>Depression<br>OCD<br>PTSD	Physical-Mental	400<br>198<br>202	3 months. Baseline, 3 months, 6 months.	Quality of life<br>6 months<br>Yes	Self-management	Some concerns	Health and Recovery Peer Programme (HARP)<br>The program builds on the Chronic Disease Self-Management Program (CDSMP), a 6-session health education program. It provides skills that help patients become more effective self-managers including modeling of healthy behaviors, problem-solving, reinterpretation of symptoms, and training in specific disease management techniques. Action plans help develop self-efficacy and build to long-term health goals.<br>Modifications of CDSMP to HARP addressed specific patient, provider, and community level determinates of health in patients with comorbid medical and serious mental disorders.<br>Materials were added addressing the mental health impact of chronic conditions and dealing with social isolation.	Two certified peer specialists led each session.<br>Patient.<br>Six 2.5 hour face-to-face sessions in groups of 6-10, with one-on-one peer coaching meetings held in between sessions for reinforcement.

For peer review only

Ell, 2010, USA	Type 1 or 2 diabetes Depression	Physical-Mental	387 193 194	12 months. Baseline, 6, 12, and 18 months.	Depression (SLC-20) 6-12-18 months Yes	Collaborative care	Low	Multifaceted Diabetes and Depression Program (collaborative care intervention). Patients chose problem-solving therapy or antidepressant, and a stepped-care algorithm was used to ensure patients received treatment consistent with their preference, clinical presentations and responses over time. Usual care was enhanced by providing patients with depression educational pamphlets and a community resource list.	1) PST provided by bilingual graduate social work diabetes depression clinical specialists (DDCSs) and/or antidepressant medications prescribed by the treating primary care physician. Patient. One-to-one but support group sessions available.

For peer review only

Ell, 2017, USA	Depression Diabetes OR heart disease OR both	Physical-Mental	348 178 170	6 weeks (plus 3 monthly boosters). Baseline, 6 months, 12 months.	Depression (PHQ-9) 12 months No	Self-management	Some concerns	A-Helping-hand (AHH) Psychoeducational intervention following a problem-solving framework, using self-care management of chronic care conditions as a specific skill-building focus with review of healthy behaviors outlined in the written materials provided to patients. Intervention provided problem-solving modeling and opportunities for patients to practice problem solving skills: 6 tasks included engagement, problem formulation, education, action planning and evaluation. Study participants in both groups were given depression educational brochures, depression photo novels, and materials on specific chronic illness treatments and self-care.	3 promotoras (trained and supervised by the research team) were added to a standard team of physician, nurse, and medical assistant. Patient. One-to-one. Six sessions over six weeks administered face-to-face (or telephone if preferred) at the patients home/any other setting preferred by patient, followed by 3 monthly booster sessions for reinforcement.

For peer review only

Fisher, 2020, Canada	At least 3 chronic conditions<br>Most common:<br>Cardiovascular<br>Kidney/Urogenital<br>Arthritis<br>Gastrointestinal<br>Endocrine<br>Hearing/Vision	Physical-Physical	59<br>30<br>29	6 months.<br>Baseline and 6 months.	SF-12 Physical<br>6 months<br>No	Self-management	High	Self-management intervention. An individualised care plan was developed to help self-management of chronic conditions, and caregivers were actively consulted in all aspects of care. Focus on client building on their strengths and abilities to enhance self-efficacy and achieve personal goals.<br>Adapted from two previous community-based self-management interventions for older adults (targeting diabetes and stroke) to target multi-morbidities.	Care Coordinator, Registered Nurse, Physiotherapist, Occupational Therapist, and Personal Support Worker<br>Patient and informal/family caregiver.<br>One-to-one<br>1. Minimum of one in-home visit from CC and 3 from PT/OT.<br>2. Monthly case conferences with provider team to discuss ongoing care plan.<br>3. Ongoing case management by CC's which involved system navigation and facilitating participant access to appropriate health and social services.

For peer review only

Garvey, 2015, Ireland	43 chronic coniditions identified, most common:<br>Arthritis<br>Congestive cardiac failure<br>Diabetes<br>Depression<br>Hypertension	Physical-Physical	50<br>26<br>24	6 weeks. Baseline and 6 weeks	N/A (participation) 6 weeks yes	Self-management	Some concerns	Occupational therapy led self-management support programme (OPTIMAL).<br>Occupational therapy focus with peer support; goals were discussed and revised at each session, considering patient preference. Intervention components included: self-management; fatigue and energy management; managing stress/anxiety and maintaining mental health/wellbeing; keeping physically active; healthy eating; managing medications; effective communication strategies; goal setting.<br>Based on the Stanford Chronic Disease Self-Management Programme: key adaptations were an occupational therapy focus, groups being professionally led and a clear focus on the specific challenges of multimorbidity.	Facilitated and led by local community-based occupational therapists.<br>Patient.<br>Group.<br>Weekly 3-hour meetings for 6 weeks held in local community health centres.

For peer review only

Goldberg, 2013, USA	Schizophrenia spectrum disorder OR bipolar disorder<br>AND one or more chronic condition, including:<br>Diabetes<br>Arthritis<br>Respiratory disease<br>Cardiovascular disease	Physical-Mental	63<br>32<br>31		3 months<br>Baseline, 3 months and 5 months	SF-12 Physical<br>3 months<br>Yes	Self-management	Some concerns	Living Well.<br>Sessions focused on action planning, peer feedback, support, modeling, problem solving, training in specific disease management techniques and the application of these skills to the topics of nutrition, exercise, sleep, medication management, addiction behaviours.<br>Modified from CDSMP for patients with serious mental illness as follows - delivery by two mental health peers OR mental health provider and peer; increased length of intervention; action plan tracker and health workbook; extra module on communication with HCP's; extra topics on mental illness; two monthly booster sessions after intervention.	Six groups - four were cofacilitated by two mental health peers who had at least one chronic condition, two were cofacilitated by a mental health professional and a peer.<br>Patients.<br>Group.<br>Weekly 60-75 minute face-to-face sessions and two monthly booster sessions post-intervention.<br>Clinics
Goorden, 2017, The Netherlands	Major depressive disorder<br>AND one or more chronic physical condition:<br>Diabetes<br>COPD<br>IBS<br>Heart failure	Physical-Mental	81<br>42<br>39		12 months.<br>Baseline, 3, 6, 9, 12 months.	N/A cost effectiveness<br>12 months	Collaborative care	High	Collaborative Care<br>Guided self-help and problem-solving treatment, prescription of anti-depressant medications according to an algorithm, and consultations with psychiatrist if necessary.	Provided by a team consisting of patient, consultant psychiatrist nurse, and consultant-liaison psychiatrist.<br>Patient.<br>One-to-one.<br>Face to face at outpatient clinic of general hospital.

For peer review only

Griva, 2019, Singapore	Diabetes<br>End stage renal disease	Physical-Mental	44<br>20<br>24	3 weeks. Baseline, 3 months	Clinical endpoint (HbA1c)<br>3 months<br>No	Self-management	Low	Combined Diabetes and Renal Control Trial (C-DIRECT) Intervention (plus usual care). Centered around self-management strategies. Patients chose a topic to focus on at each session. Session 1: feedback on Hba1c; information/advice on chosen topic; goal setting; food record assignment. Session 2: review goal setting and problem solve any barriers; revise goals as needed; review food record and provide support if necessary; choose topic (information/advice and new goal setting). Session 3: review goals and problem solve barriers; revise goals as needed; choose topic (information/advice and new goal setting). Provide additional advice and resourse links to go away with.	DM link nurse.<br>Patient.<br>One-to-one.<br>3 weekly sessions 30-60 minutes long, delivered at patient bedside at dialysis centre upon cannulation and connection to haemodialysis machine.

For peer review only

Guo, 2020, China	Atrial Fibrilliation AND Congestive heart failure Hypertension Diabetes Prior ischemic stroke Vascular disease Coronary artery disease Peripheral arterial disease Renal dysfunction Liver dysfunction Prior bleeding Prior brain bleeding Hyperthyroidism Cardiomyopathy	Physical-Physical	40 hopsitals randomised (20 to each arm) 3324 1646 1678	12 months Baseline, 6 and 12 months.	N/A composite hospital 12 months Yes	Collaborative care	Low	mobile Atrial Fibrillation Application (mAFA). Installed on smart phones. Provided clinical decision support tools for doctors to facilitate treatment recommendations, educational materials, patient self-care, and structured follow-up, to support AF management: Avoid stroke (tracking bleeding events/trends; managing treatment and anticoagulant use), Better symptom management (tracking symptoms, monitoring cardiac rhythm using smart watches), Cardiovascular and other comorbidities risk management (tracking BP and lifestyle factors). The app encouraged patient engagement through participation in educational programs, provision of informative articles, videos, game playing, etc.	Doctors. Doctors and patients. One-to-one. Followed up at outpatient clinics.

For peer review only

Hernandez-Quiles, 2021, Spain	Advanced heart/lung failure/both.<br>AND one of:<br>Hypertension<br>Heart Failure<br>Atrial fibrillation<br>Dyslipemia<br>Diabetes<br>CHD<br>COPD<br>Chronic kidney disease<br>Obesity<br>Chronic anemia<br>Valvular heart disease<br>Pulmonary hypertension<br>Sleep apnea hypopnea syndrome<br>Hypothyroidism<br>Depression	Physical-Physical	510<br>255<br>255	180 days<br>0, 15, 45, 90, 180 days.	N/A hospital admissions<br>45, 90 and 180 days<br>Yes	Self-management	Low	TELECARE (plus usual care).<br>Usual care was frequent clinical and bio-parameters self-checks (BP, heart rate, oxygen saturation, weight, and blood glucose)Patients were provided with educational material and instructed how to recognize exacerbation symptoms. For the intervention group, a synchronous monitoring equipment was added for biological and medical questionnaires so data was available in real time for healthcare team. An alarm system was added to induce a healthcare response if there was variation in data or missing data.	Health care team<br>Patient tracked own data.<br>One-to-one.<br>Patients inserted data daily for first 15 days (frequency was then altered depending on stability and ocurrence of incidents).

For peer review only

House, 2018, UK	Type 2 diabetes<br>Obesity<br>Intellectual disability	Physical-Physical	82<br>41<br>41	4 months<br>Baseline, 6 month follow-up (reduced to 4 because of project deadlines)	Clinical endpoint (HbA1c)<br>4-6 months<br>No	Self-management	Some concerns	Supported self-management<br>The intervention emphasized realistic goal setting, identifying resources and barriers likely to influence success in reaching goals, and regular self-monitoring of goal attainment.<br>Four components: (1) Establishing the participant's daily routines and lifestyle; (2) Identifying all supporters and helpers and their roles; (3) Setting realistic goals for change; (4) Monitoring progress against agreed upon goals.<br>Materials: for nurses - weekly timetable, charts to record friends/family/helpers, charts to complete in collaboration with participant; for participants - OK Diabetes board to record goals and visual prompts, a written action plan, tear off slips to record daily actions; for supporters/helpers - information sheet on study, card summarising their role in helping the person with diabetes.<br>The intervention was modelled on existing self-management interventions, with adjustments [illegible]	Diabetes specialist nurse.<br>Patient (73% had their supporter present for at least one session).<br>One-to-one.<br>4 sessions, most (92%) took place in participants homes, ranging from 13-95 minutes (typical total intervention time was 2 hours).

For peer review only

Jackson, 2021, Nigeria	HIV<br>Hypertension<br>Other:<br>Diabetes<br>Peptic ulcer disease<br>Renal disease<br>Hypertensive heart disease<br>Osetoarthritis<br>Haemorrhoids<br>Benign prostatic hyperplasia	Physical-Physical	206<br>103<br>103	12 months. Baseline, 6 months, 12 months.	Clinical outcomes (Blood pressure)<br>12 months<br>Yes	Self-management	Low	Pharmaceutical care (PC) (plus routine care).<br>Routine care - BP taken by nurse, physician prescribes medication/orders lab tests, meeting with adherence counsellor, then medication/ counselling from pharmacist.<br>Intervention also exposed to PC from research pharmacist. This involved structured education/ counselling focused on self-monitoring of BP, healthy eating, maintaining an ideal body weight, proper foot and dental care, healthy coping, use and storage of medications; lifestyle modifications with emphasis on the dietary approaches to stop hypertension (DASH) diet, physical activity, smoking cessation, moderation of alcohol intake; reviewing date of the next appointment and prescription with the patient, expected side effects of the medications, and how to prevent/ manage such side effects.	Research pharmacist.<br>Patient.<br>One-to-one.<br>Face-to-face bi-monthly appoointments at hospital, as well as two text messages per month.

For peer review only

Kamradt, 2019, Germany	Type 2 diabetes and<br>Two or more of the following:<br>Coronary heart disease<br>Chronic pain<br>Depression<br>Chronic heart failure<br>COPD<br>Artherosclerosis<br>Cerebrovascular disease	Physical-Physical	495<br>252<br>243	9 months.<br>Baseline, 9 months.	Quality of life<br>9 months<br>No	Collaborative care	Some concerns	Care management intervention. Embedded in a regional network of primary care practices. It included two home visits with structured assessments of clinical and social needs, aiming to reduce barriers to patient's access of support and healthcare systems. Frequent telephone monitoring was also used to improve and sustain changes in self-care behaviour, and give feedback related to patients' control of diseases and current (self-care) activities to reinforce positive behavioral changes.	Specially trained medical assistants, physicians specialising in family/internal medicine, and net-care managers.<br>Patient.<br>One-to-one.<br>Two home visits and 15 structured telephone monitoring contacts over 9 months.

For peer review only

Kanwal, 2018, USA	HCV Depression	Physical-Mental	309<br>156<br>153	6 months<br>Baseline, 6 months, 12 months.	Depression (SLC-20)<br>12 months<br>Yes	Collaborative care	Some concerns	Hepatitis C Translating Initiatives for Depression Into Effective Solutions (HEPTIDES)<br>The intervention included: participant education and activation, assessment of treatment barriers and possible resolutions, depression symptom and treatment monitoring, substance abuse monitoring, and instruction in self-management. The intervention used a stepped-care model for depression treatment, with five steps: self-management education, depression care team treatment suggestions, pharmacotherapy suggestions, combination pharmacotherapy and specialty mental health counseling, and referral to specialty mental health.	Depression care manager, pharmacist and psychiatrist. They convened once a week and communicated with HCV and mental health clinicians at each hospital site to discuss treatment and make decisions.<br>Patient.<br>One-to-one via telephone delivered by DCM, every 2 weeks during acute treatment and every 4 weeks during self-management education or continuation treatment.

For peer review only

Katon, 2004, USA	Diabetes Depression	Physical-Mental	329 164 165	3-6 months. Baseline, 3 months, 6 months, 12 months.	Depression (SLC-90) 6 and 12 months Yes	Collaborative care	Low	Collaborative care. Individualised, stepped-care depression treatment programme designed to improve quality of care and outcomes of depression. Patients were offerd an intial choice of two treatments: antidepressant medication or problem solving treatment (PST). If patients still had persistent depressive symptoms (< 50% decrease in severity based on the PHQ-9) 10 to 12 weeks after initial treatment, they could (1) switch to a second antidepressant; (2) switch to the alternative treatment (from PST to medication or vice versa); (3) receive augmentation with PST or medication with the first treatment they had received; or (4) receive a psychiatric consultation. If there was still no improvement after a further 8 to 12 weeks, referral to speciality care was made. Once patients reached a significant decrease in clinical symptoms (< 50% decrement in symptoms), the nurse began continuation phase	Depression clinical specialist nurse in collaboration with the primary care physician. Patient. One-to-one. Initial 1-hour visit followed by twice-a-month, half-hour appointments (telephone and in person) for 12 weeks followed by monthly scheduled telephone contacts for continuation phase treatment.

For peer review only

Katon, 2010, USA	Depression Diabetes/Coronary heart disease/both	Physical-Mental	214<br>106<br>108	12 months. Baseline, 6 months, 12 months.	Composite depression and clinical 12 months Yes	Collaborative care	Low	Collaborative care management. The intervention combined support for self-care with pharmacotherapy, aiming to manage depression and improve glycemic, BP and lipid control. Patients worked collaboratively with nurses and primary care physicians to establish individualized clinical and self-care goals. Using motivational and encouraging coaching, nurses helped patients solve problems and set goals for improved medication adherence and self-care. Patients also received self-care materials and self-monitoring devices. Nurses monitored the patient's progress and medications were adjusted where necessary according to protocol. Once target levels were achieved, a maintenance plan was developed that included stress reduction, behavioral goals, continued use of medications, and identification of prodromal symptoms associated with worsening depression and glycemic control.	Nurses, in collaboration with primary care physicians.<br>Patient.<br>One-to-one.<br>Visits every 2 to 3 weeks at the patients primary care clinic; once maintenance plan was in place. Patients whose disease control worsened were offered follow-up visits and telephone calls with increased treatment intensity.

For peer review only

Khunti, 2021, UK	Conditions accepted in inclusion criteria: Cardiovsacular Asthma/COPD High dependency/ LTC's Depression Musculoskeletal	Physical-Physical	353 180 173	6 months. Baseline, 6 months, 12 months.	Behaviour change (physical activity) 12 months Yes	Self-management	Low	Movement through Active Personalised engagement (MAP) programme. Four sessions with person-centred self monitoring and goal setting. The sessions focused on increasing PA, and addressed key non-disease-specific self-management challenges and themes (mastering emotions, managing treatments, communication within health care). Increasing PA was further facilitated and encouraged by providing resistance bands in order to complete resistance training at home and pedometers to help track PA. Regular reminder and motivational texts were sent to encourage long-term behaviour change.	Trained facilitator. Patient. Group. 1.5 hour face-to-face sessions delivered at two week intervals in local community. Texts were sent daily during the 8 weeks of sessions up until 2 weeks after the 6-month timepoint, then 3 times a week over the remainder of the 12 month period.

For peer review only

Koesoemadinata, 2021, Indonesia	Tuberculosis Diabetes	Physical-Physical	108 60 48	6 months. Baseline, 6 months.	N/A patient knowledge 6 months No	Cognitive and/or Behavioural Activation	Some concerns	Structured counselling/education. At each visit, blood glucose level was measured, and DM medication was adjusted according to a structured algorithm. Educational messages were discussed according to a specified checklist using flip charts. Topics included: TB: Cause, transmission, treatment compliance, possible side effects; What is DM? What are the symptoms? What can be done?; Blood glucose measurement and recording; Importance of glycaemic control; Metformin and insulin; Hyper- and hypoglycaemia; The importance of smoking cessation; DM and TB together; Healthy lifestyle: diet, exercise, and smoking cessation; Management after the TB treatment is completed. A leaflet was also provided to patients with information regarding recommended food types and amounts.	Doctor. Patient. One-on-one. Face-to-face in a research clinic at weeks 1, 2 and 4, then monthly until 6 months.

For peer review only

Kroenke, 2009, USA	Musculoskeletal pain Depression	Physical-Mental	250 123 127	12 months. Baseline, 1, 3, 6, and 12 months.	Depression (SLC-20) 12 months Yes	Collaborative care	Low	Stepped Care for Affective Disorders and Musculoskeletal Pain (SCAMP). Step 1 (weeks 1-12): Optimised antidepressant therapy. Adjustments in medication as per an algorithim to optimise antidepressant management. Step 2 (weeks 13-26): Pain self-management programme. Focus on increasing self-efficacy, social support to self-manage pain, problem solving techniques, behavioural change strategies, learning about chronic pain and receiving feedback from nurse. Step 3 (weeks 27-52): continuation phase. Evaluation. The care manager assessed current self-management strategies and assisted patients with new behavioral plans. Antidepressant dose could be increased, or if necessary a referral to a psychiatrist offered.	Nurse care manager and physician depression specialist. Patient. One-to-one. 5 inperson contacts (baseline, 6, 12, 16, and 20 weeks) and 8 telephone contacts (1, 3, 9, 14, 18, and 22 weeks, and at 8 and 10 months). Extra contacts could occur depending on treatment changes or a patient's clinical needs.

For peer review only

Lear, 2021, Canada	2 or more of: Diabetes Heart failure Ischemic heart disease Chronic kidney disease COPD	Physical-Physical	230 (219 - one withdrew after randomisation) 117 (116 - see above) 113	Two years. Baseline, 12 months, 24 months.	N/A Hospital admissions 24 months No	Self-management	Low	Internet chronic disease management (CDM). Participants used the CDM website to complete daily symptom reports which asked questions about disease-specific symptoms and biometric data, which generated alert for specific scenarios or targets not being met. The nurse would then call to discuss/readjust targets, and support patient self-management or recommend follow up with primary care practicioner/hospital referral if necessary. Every 8 weeks, participants answered a lifestyle questionnaire, and alerts were sent based on thresholds for these questions. If necessary the nurse gave a referral to a dietitian/exercise specialist or use of a psychosocial support workbook. Participants had access to a public forum, graphical presentations of their biometric data overlaid with relevant alerts, their action plan, and external online resources.	Coordinated care between participant, their primary care practicioner, and a nurse managing the CDM programme (supported by a dietitian and an exercise specialist). Patient. One-to-one. Telephone.

For peer review only

Lenferink, 2019, The Netherlands and Australia	COPD and one or more of: Ishemic heart disease Chronic heart failure Diabetes Anxiety Depression	Physical-Physical	201 102 99	4 weeks intensive and 3 follow up calls at weeks 8, 20 and 36 Baseline, 6 months, 12 months.	COPD exacerbation 12 months No	Self-management	Some concerns	Self-management intervention. Week 1: knowledge on COPD/ comorbidities, symptom recognition/ monitoring, self-treatment action plan (linked to diary), breathing/relaxation exercises, how to check blood glucose. Week 2: record 'usual symptoms', diary training, exacerbation action plan training, mastery of skills (e.g. inhaler use). Week 3: importance of exericse, diet/lifestyle behaviours, re-iteration of diary/action plan use, reiteration of breathing/relaxation. Week 4: re-iteration of diary/action plan use, feedback on diary completion, feedback on actions. Both intervention and usual care groups completed daily symptom diaries for the 12 month period. For the intervention group, self-management behaviours were reinforced, and exacerbation action plans for all co-morbidities were directly linked to their diaries.	Researchers Patient. Weeks 1 and 3: group. Weeks 2 and 4: individual. Phone calls at weeks 8, 20, and 36. Location of face-to-face sessions unspecified.

For peer review only

Markle-Reid, 2018, Canada	Type 2 diabetes<br>Top 3 most common chronic conditions:<br>Hypertension<br>Cardiovascular<br>Arthritis	Physical-Physical	159<br>80<br>79	6 months. Baseline, 6 months.	SF-12 physical and mental<br>6 months<br>Mental - yes<br>Physical - no	Self-management	Low	Self-management programme. The intervention focused on self-efficacy, self-management, holistic care, and individual and caregiver engagement. It was client-driven therefore fleixble to client needs; the indvidual was fully engaged in developing and tailoring their own care plan. The programme offered up to 3 in-home visits from nurse/dietitian/both, monthly group wellness sessions, monthly case conferences amongst the team, and ongoing nurse-led care coordination.	Nurses and dietitians, a program coordinator from a community partner, and peer volunteers.<br>Patient and carer (optional)<br>One-on-one sessions at patients home, group sessions at diabetes education centre.

For peer review only

McDermott, 2015, Australia	Type 2 diabetes One or more other significant comorbidity	Physical-Physical	213 100 113	18 months. Baseline, 18 months.	Clinical (HbA1c) 18 months yes	Self-management	Low	"Getting better at chronic care". Health-worker led case management intervention (one recruited at each site to work as part of primary care team and given caseload of 9 to 26 clients). The roles of the health workers included helping patients make and keep appointments, understand their medications and nutrition and the effects of smoking and work with the family to help support the patient in self-management. Home visits and out-of-clinic care were conducted according to patient preference. Specific training and practice in: chronic care model and evidence-based management and treatment goals for chronic conditions; "hands-on" case management (regular client home visits, including basic diabetes care); working in a primary care team with clear roles/ responsibilities; engaging with families and using local resources to support effective client self-management.	Indigenous healthcare worker (IHW) from community. Health worker/client. One-to-one. Home visits OR out of clinc care (based on preference).

For peer review only

Merlin, 2018, USA	HIV Chronic pain	Physical-Physical	44 22 22	12 weeks. Baseline, 12 weeks	Pain (BPI) 12 weeks no	Self-management	Some concerns	Skills TO Manage Pain (STOMP) plus usual care. Incorporated self-regulation, self-efficacy, observational learning, and outcome expectations. Individual sessions: to build pain self-management skills. All participants received a pain education session, and then selected 5 of the remaining 9 sessions (physical activity and your pain, losing weight to improve your pain, relaxation skills to prevent your pain, sleeping better to help your pain, thinking diferently about your pain, building self-worth, talking with our family and friends about pain, taking opioid pain medications). Group sessions: to enhance peer support related to chronic pain. Each session included sharing refections on lessons learned and goals set during one-on-one sessions, and challenges encountered.	Two peers with HIV and chronic pain ("pain pals") and two research staff ("pain coaches"), working in pal-coach pairs. Participant. Six (in-person) one-to-one sessions (led by pain coach) and six group sessions (led by pal-coach pair), alternating weekly.

For peer review only

Miklavcic, 2020, Canada	Type 2 diabetes Two or more other chronic conditions	Physical-Physical	132<br>70<br>62	6 months. Baseline, 6 months.	Physical functioning<br>6 months<br>No	Collaborative care	High	Aging, Community and Health Research Unit Community Partnership Program.<br>Focused on the role of self-efficacy in achieving self management behaviours, and tailored to patient preferences and needs.The program included up to 3 home visits by RN or RD; 6 monthly group sessions which included education, exercise, a light meal, and peer support; monthly intervention team case conferences (share observations about participants strengths, challenges and goals, identify needs related to other community-based service, and prepare for upcoming group sessions); care coordination to link the client to relevant health or social services.	Interprofessional team consisting of a Registered Nurse (RN), Registered Dietitian (RD), and Program Coordinator (PC).<br>Patient (family caregiver was also invited to attend).<br>One-to-one sessions at patients home, group sessions at community site once a month.

For peer review only

Morgan, 2013, Australia	Depression Type 2 diabetes/ coronary heart disease (or both)	Physical-Mental	400 206 194	6 months. Baseline, 6 months, 12 months	Depression (PHQ-9) 6 months Yes	Cognitive and/or Behavioural Activation	Some concerns	TrueBlue Collaborative care approach. Involved visit with PN followed by usual GP, in which stepped care (psychotherapy or pharmacotherapy) was offered if PHQ-9 scores had not improved or dropped below 5. The PN obtained physical measures and reviewed recent pathology results and lifestyle risk factors. They worked with the patient to identify barriers to achieving goals and discuss ways to overcome them. They assisted the patient with self-management by discussing available educational resources and setting personal goals for review at the next visit.	Practice Nurse (PN) Patient One-to-one. Patient visited their practice every 3 months for 45 minutes (then 15 minutes with the GP).

For peer review only

Naik, 2019, USA	Diabetes Depression	Physical-Mental	225 136 89	6 months Baseline, 6 months, 12 months.	Depression (PHQ-9) and clinical (HbA1c) 6 and 12 months No	Self-management	Low	Healthy Outcomes through Patient Empowerment (HOPE). The first 2 sessions focused on building rapport, introducing and clarifying values, collaboratively setting initial goals, identifying potential skill sets to address goals, and empowering patients to advocate for their health through active communication with clinicians. Sessions 3-6 focused on discrete skill modules (increasing pleasant activities, using thoughts to improve wellness, diet, physical activity, medication management, and relaxation) customized to meet their goals. Sessions 7-9 focused on maintenance skills (reviewing action plans and overcoming barriers).	Trained health professional. Participant. One-to-one. 9 telephone sessions: biweekly (30-40 minutes) in months 1-3, and monthly (15 minutes) from months 4-6). No contact in following 6 months.

For peer review only

Ose, 2019, USA	Type 2 diabetes<br>Two or more other chronic conditions including:<br>Artherosclerosis<br>Coronary heart disease<br>Chronic obstructive lung disease<br>Asthma<br>Cerebrovascular diseases<br>Depression<br>Heart failure<br>Parkinsons<br>Chronic pain	Physical-Physical	495<br>252<br>243	9 months<br>Baseline, 9 months.	Self-care (Summary of Diabetes Self-Care Activities Measure (SDSCA-G)<br>9 months<br>No	Self-management	Low	Care management intervention. 3 steps: (1) Structured, IT-supported assessment performed by net-care managers to capture medical/non-medical needs; (2) goal setting between patient and primary care physician based on results of assessment; (3) telephone monitoring by net-care managers to detect acute clinical needs and provide support. Embedded in PCN (net-care manager links to responsible physician via a medical assistant).	Net Care Manager.<br>Patient.<br>One-to-one.<br>(1) at-home visits/face-to-face meetings 3 times within initial 2 weeks, then again at 6 months; (2) once at week 2; (3) every 2 weeks for the first 6 months then every month for the next 3 months.

For peer review only

O'Toole, 2021, Ireland	Conditions not listed. Mean 4.5 per participant	Physical-Physical	149<br>78<br>71	6 weeks. Baseline, 6 weeks, 6 months.	Quality of life (EQ-5D-3L) and behaviour change (participation)<br>6 weeks<br>No	Self-management	Some concerns - a lot of analyses were completed	Occupational therapy-led self-management support programme (OPTIMAL).<br>Intervention incorporated performance accomplishments, vicarious learning, social/verbal persuasion, reinterpretation of physiological and emotional states. Patients' programme goals were set in week 1 and reviewed weekly. They were also given educational and health-promoting resources/materials. Week 1: Introduction to self-management, activity and health, and goal-setting. Week 2: Fatigue management and healthy eating. Week 3: Maintaining physical activity. Week 4: Maintaining mental wellbeing. Week 5: Managing medications. Week 6: Communication and programme review.	Occupational therapist.<br>Patient.<br>Group.<br>2.5 hour face-to-face session once a week for 6 weeks, in a primary care centre or community resource centre.

For peer review only

Park, 2014, South Korea	Two or more of: Stroke Parkinsons Dementia	Physical-Physical	43 21 22	8 weeks. Baseline, 8 weeks	self-management behaviors, self-efficacy, and health status 8 weeks Yes	Cognitive and/or Behavioural Activation	Low	Health coaching self management programme (HCSMP). Group level approach: structured health education developed for older adults, regarding participants' diseases and self-management strategies, then a period of physical activity to enhance cognition and body movements. Individual level approach: individual counselling and goal setting for self-management behaviours. Facility-level approach: principal investigator met frequently with the director and chief manager of the nursing home to help support and facilitate patient progress.	Trained research team members who were geriatric nurse speciaists. Participant. Group sessions delivered face to face at nursing home for 8 weeks; education (Monday) and exercise (Thursday), both lasting 1 hour. Individual sessions: 8 face-to-face coaching sessions for approx 20 minutes,

For peer review only

Pibernik-Okanovic, 2009, Croatia	Diabetes Depression	Physical-Mental	50 25 25	5 weeks Baseline, 6 months, 12 months	Depression (CES-D) and clinical (HbA1c) 6 and 12 months No	Self-management	Low	Psycho-education. Four interactive meetings on the following topics: (1) symptoms of depression, interaction of depression and diabetes; (2) alleviaing burden of depression through activities and problem solving; (3) associations between depression and cognitive processes – thoughts, beliefs and attitudes that induce and maintain depression; (4) developing a personal plan for managing depression-related problems in the future. Patients were provided with a self-help manual and kept daily mood and activities diaries.	Not specified. Patient. Group. Four 90 minute sessions - first two held within the first week and the third and fourth held at two week intervals

For peer review only

Rose, 2018, Canada	COPD and two or more of: Cardiovascular disease Depression Diabetes Osteopenia and osteoporosis Gastro-oesophageal reflux disease Hypothyroidism Osteoarthritis Glaucoma and cataracts Cachexia and malnutrition Chrornic kidney disease Anxiety Peripheral muscle dysfunction Obstructive sleep apnoea Lung cancer Cerebrovascular accident	Physical-Physical	470 236 234	12 months. Baseline, 3 months, 6 months, and 12 months.	Hospital admissions 12 months No	Cognitive and/or Behavioural Activation	Low	Multi-component, case manager led intervention. Components included: (1) standardised 40 minute COPD education session on enrolment; (2) individualised care and action plans for COPD exacerbation recognition, self-management and management of comorbidites; (3) telephone consultations containing standardised reinforcement/ motivational interviewing focusing on health behaviours, action plan teach-back sessions, symptom monitoring, and problem solving strategies; (4) ongoing case manager communication with family physicians and with hospital specialists including respirologists; and 5) priority access to ambulatory outpatient clinics.	Case manager. Patient. One-to-one. 12 weekly telephone calls (after initial education session at ED/hospital/outpatient clinic), followed by monthly calls for 9 months.

For peer review only

Ross, 2005, Canada	Asthma<br>Panic disorder	Physical-Mental	48 (25 analysed)<br>25 (15 analsyed)<br>23 (10 analysed)	8 weeks.<br>Intervention group: Baseline, 8 weeks, and 6 month follow-up.<br>Wait-list control group: baseline (coincided with IG pre), 8 weeks (coincided with IG post), 16 weeks, and 6 month follow-up	Panic disorder and asthma outcomes<br>8 weeks<br>Yes	Cognitive and/or Behavioural Activation	Some Concerns - multiple scales used to measure the same outcome	CBT-AE.<br>CBT: (a) education about anxiety and panic; (b) cognitive therapy techniques aimed regarding anxiety and panic; (c) training in slow diaphragmatic breathing to aid physical symptoms of panic attacks; and (d) interoceptive exposure exercises designed to reduce participants' fear of anxiety/panic attack symptons.<br>AE: (a) information about airway inflammation and bronchospasm; (b) rationale, correct use, and side effects of medications, (c) inhaler techniques; (d) methods of self-monitoring, symptoms, and peak flow; (e) asthma triggers and strategies for control; and (f) action plans. Asthma diaries kept by the participants were reviewed during each treatment session to reinforce the importance of the participant's role in self-management and self monitoring.	Two nurse clinicians (one Asthma Educator, one with postdoctoral training in cognitive therapy).<br>Participant.<br>Small groups.<br>12 face-to-face 90 minute sessions with both CBT and AE components. Sessions 1-8 conducted twice weekly, and sessions 9-12 spaced one week apart.
Sajatovic, 2017, USA	Serious mental illlness<br>Type 2 diabetes	Physical-Mental	200<br>100<br>100	60 weeks.<br>Baseline, 13 weeks, 30 weeks, 60 weeks	Psychiatric symptoms<br>60 weeks<br>yes	Self-management	Some Concerns	Targeted Training in Illness Management.<br>Group-based psychosocial treatment that blends psychoeducation, problem-identification, goal-setting, behavioral modeling, and care linkage.	Nurse and peer educators.<br>Participant.<br>Group/one-to-one.<br>12 weekly face-to-face sessions, followed by 15 minute one-to-one telephone calls every 2-4 weeks.

For peer review only

Salisbury, 2018, UK	Cardiovascular disease or chronic kidney disease<br>Stroke or transient ischaemic attack<br>Diabetes<br>COPD or asthma<br>Epilepsy<br>Atrial fibrillation<br>Serious mental illness<br>Depression<br>Dementia<br>Learning disability<br>Rheumatoid arthritis	Physical-Physical	33 practices randomised (17 to intervention, 16 to control).<br><br>1546<br>797<br>749	6 months. Baseline, 9 months, 15 months	Quality of life (EQ-5D-3L)<br>15 months<br>No	Collaborative care	Some concerns - self-completed measure for primary outcome and all were aware of group allocation	3D intervention<br>Replace separate disease focused review with one 6-monthly comprehensive multidisciplinary review. Done in 3 stages: (1) nurse review - identifies important health problems and how these affect life, and disease-specific care that the patient needs; (2) pharmacist reviews medication and makes recommendations for treatment; and (3) physician review - considers nurse and pharmacist reviews, then agrees on collaborative health plan with patient (which is printed off) specifying goals and how these will be achieved over the next 6 months of consultations.	Nurse, physician and pharmacist.<br>Patient.<br>One-to-one.<br>Two 6-monthly reviews consisting of two appointments (nurse and physician) and a medication review, then consultations over 6 months as per the health plan.

For peer review only

Schneider, 2015, USA	Type 2 diabetes Depression	Physical-Mental	29<br>15<br>14	6 months. Baseline, 3 months, 6 months.	Clinical outcomes (HbA1c) and depression (BDI-II)<br>3 and 6 months<br>No	Cognitive and/or Behavioural Activation	Low	Behavioural Activation and Exercise Condition.<br>Classes contained 3 parts - (i) warm-up, (ii) exercise (different types for first 12 weeks then based on paricipant enjoyment for next 12, gradually increasing in intensity each week), and (iii) cool down and BA (activity and mood monitoring, exploration of ways to make exercise more enjoyable, a behavioural contract with friends/ family to promote exercise, a list of life values and how to connect exercise to them, and exercise counseling strategies including self-monitoring activity using a pedometer, monitoring exercise intensity via perceived exertion and heart rate, identifying facilitators to exercise and engaging in problem solving for exercise barriers).	Female group leader.<br>Participant.<br>Group.<br>One orientation session and 38 classes over 4 weeks at a medical school site. The 90-minute classes occurred 2 times per week for 16 weeks, once a week for 4 weeks, and then once every other week for 4 weeks.

For peer review only

Takahashi, 2012, USA	Myocardial infarction<br>Congestive heart failure<br>COPD<br>Diabetes<br>Renal disease	Physical-Physical	205<br>102<br>103	12 months<br>Baseline, 12 months	Hospital admissions<br>12 months<br>No	Self-management	Some concerns - outcome assessors were not blind at follow-up, more deaths in intervention group	Telemonitoring.<br>An FDA approved telemonitoring device was used in the patients home. Patients performed daily assessment of symptoms and biometrics, and data were downloaded and reviewed daily by the health care team. A registered nurse communicated with the individuals via phone or videoconference if alerts arose, communicating with their primary physician about treatment options if needed.	Registered nurse and (if required) primary physician oversaw the process.<br>Patient.<br>One-to-one.<br>Daily 5-10 minute sessions<br>Home or clinical

For peer review only

Vera, 2010, USA	Depression<br>And one or more of:<br>Diabetes<br>Hypothryoidism<br>Asthma<br>Hypertension<br>Chronic bronchitis<br>Arthritis<br>Heart disease<br>High cholesterol<br>Stroke	Physical-Mental	179<br>89<br>90	6 months. Baseline, 8, 16 and 24 weeks.	Depression (SCL-20) and Functioning (SF-36)<br>6 months<br>Yes	Collaborative care	Low	Collaborative care.<br>A multicomponent intervention, including program oversight and teamwork among physicians, mental health specialists, and care managers. Patients were provided education about depression and a choice of 13 CBT sessions or antidepressant medication as initial treatment. The care manager participated in the coordination of treatment initiation, as well as monitoring treatment adherence, side effects, and clinical response, while liaising with the psychiatrist and patients physician. Extra contacts were available to help patients overcome barriers and provide treatment adherence support.	Care manager.<br>Patient<br>One-to-one.<br>Patients were contacted by telephone or in person every 2 weeks initially, and then monthly up to 6 months.

For peer review only

Wakefield, 2011, USA	Type 2 diabetes Hypertension	Physical-Physical	302<br>102 (low intensity) / 93 (high intensity)<br>107	6 months. Baseline, 6 months, 12 months	Clinical (HbA1c and SBP)<br>6 month<br>Yes	Self-management	Low	Telehealth intervention.<br>In a home telehealth device, all intervention participants logged blood pressure, blood glucose and responses to standardised questions (which were reinforced when answered correctly and reviewed/explained when answered incorrectly), which were reviewed daily by a registered nurse to determine whether the participant needed a follow-up (e.g. if clinical parameters were out of range or the participant needed extra support). In the high intensity group, a disease management algorithm was used, with scheduled prompts for rotating questions and education focused on diet, exercise, smoking cessation, foot care, advice for sick days, medications, weight management, preventive care, and behavior modification and lifestyle adjustments. In the low intensity group, participants responded to a smaller subset of questions from the HI group and did not use the algorithm.	Nurse.<br>Participant.<br>One-to-one.<br>Telehealth device was in patient home - follow-ups were made via telephone, letter or directly through the device as and when needed.

For peer review only

Williams, 2012, Australia	Diabetes<br>Chronic kidney disease/diabetic kidney disease<br>Hypertension	Physical-Physical	80<br>39<br>41	3 months.<br>Baseline, 3 months, 6 months, 9 months.	Medication adherence<br>3-6-9 months<br>No	Self-management	Low	Medication Self-Management Intervention (MESMI).<br>The intervention consisted of self-monitoring of blood pressure (participants were taught how to do this and recorded it daily for 3 months), an individualized medication review, a 20-minute DVD (using an interactive, psychosocial approach to motivate people to take their medications), and motivational interviewing to support blood pressure control and optimal medication self-management.	Intervention nurse.<br>Participant.<br>One-to-one.<br>Motivational interviewing was done fortnightly via telephone calls, starting 2 weeks after the intervention home visit until 3 months.
Wilson, 2018, USA	Depression<br>Any other chronic disease (most common: chronic pain, multiple sclerosis, cardiovascular disease, mental disorder)	Physical-Mental	53<br>27<br>26	8 weeks.<br>Baseline, 4 weeks, 8 weeks.	Depression (PHQ-8)<br>8 weeks<br>Yes	Cognitive and/or Behavioural Activation	Some concerns - as unblinded participants completing assessments, including primary outcome	Think Clearly About Depression.<br>Computer based intervention to teach mildly/moderately depressed individuals about the role of thinking in the development and maintenance of depression, using videos, interactive activities and homework exercises to challenge depressive thoughts and practice hopeful and constructive thinking, as well as a daily mood tracker and a schedule for mood boosting self-management activities. To increase engagement, the researchers created a program guide to assist participants and sent weekly prompts for the first 4 weeks.	Research team (two psychiatric nurse practicioners and a psychiatrist).<br>Participant.<br>One-to-one.<br>The program could be accessed at any time of day in any setting of the participants' choosing where they had Internet services and a personal computer or smartphone.

For peer review only

Yu, 2020, Canada	Diabetes AND two of:<br>Heart disease<br>Stroke<br>Hypertension<br>Cancer<br>Chronic lung disease<br>Arthritis<br>Inflammatory bowel disorder<br>Urinary incontinence	Physical-Physical	10 primary care practices were randomsed (5 in each arm).<br>213<br>102<br>111	6 months<br>Baseline, 6 months, 12 months.	Behaviour change (Decisional conflict)<br>12 months<br>No	Self-management	Some concerns - as unblinded participants completing assessments, including primary outcome	MyDiabetesPlan.<br>Web-based intervention where participants provide cardiometabolic and psychosocial profiles and general care priorities. An individualised action plan is then generated with diabetes-specific goals and strategies. For the first 6 months, MyDiabetesPlan was completed by a member of the clinical team with the patient, then their action plan was reviewed. At the 6-month mark patients were given a "how to" guide for MyDiabetesPlan and from then on logged the information themselves before their appointment.	Clinician (nurse, dietitian, physician, or pharmacist).<br>Patient.<br>One-to-one.<br>Clinical encounters were done face-to-face at the participants primary care clinic.

For peer review only

Study	Outcome	Effective	Intervention type	Intervention length	M-LTCs combination
Blank, 2011	Clinical endpoint	No	Self-management	>6months	Physical-Mental
Chwastiak, 2018	Clinical endpoint	No	Collaborative care	3-6months	Physical-Mental
Ell, 2010	Psychological distress (depression)	Yes	Collaborative care	>6months	Physical-Mental
Fisher, 2020	Functioning	No	Self-management	3-6months	Physical-Physical
Guo, 2020	N/A	N/A	Collaborative care	>6months	Physical-Physical
Hernandez, 2021	N/A	N/A	Self-management	3-6months	Physical-Physical
Kamradt, 2019	Quality of life	No	Collaborative care	>6months	Physical-Physical
Katon, 2004	Psychological distress (depression)	Yes	Collaborative care	>6months	Physical-Mental
Khunti, 2021	Behaviour change	Yes	Self-management	>6months	Physical-Physical
Koesoemadinata, 2021	N/A	N/A	Cognitive and/or Behavioural activation	3-6months	Physical-Physical
Lear, 2021	N/A	N/A	Self-management	>6months	Physical-Physical
Lenferink, 2019	Clinical endpoint	No	Self-management	>6months	Physical-Physical
Merlin, 2018	Pain	No	Self-management	<3months	Physical-Physical
Ose, 2019	N/A	N/A	Self-management	>6months	Physical-Physical
O'Toole, 2021	Quality of life<br>Behaviour change	No<br>No	Self-management	<3months	Physical-Physical
Pibernik, 2009	Psychological distress (depression)<br>Clinical endpoint	No<br>No	Self-management	<3months	Physical-Mental
Rose, 2018	N/A	N/A	Cognitive and/or Behavioural activation	>6months	Physical-Physical
Takahashi, 2012	N/A	N/A	Self-management	>6months	Physical-Physical
Taylor, 2003	Clinical endpoint	Yes	Collaborative care	>6months	Physical-Physical
Vera 2010	Psychological disress (depression)<br>Functioning	Yes<br>Yes	Collaborative care	3-6months	Physical-Mental
Wakefield, 2011	Clinical endpoint	Yes	Self-management	3-6months	Physical-Physical
Williams, 2012	Behaviour change	No	Self-management	3-6months	Physical-Physical

For peer review only

Supplementary Table 4. Pooled effects of outcomes, stratified by types of long-term condition combination types.

	Physical-Physical			Physical-Mental		
**Post-intervention**	*n*	SMD [95% CI]	I^2	*n*	SMD [95% CI]	I^2
Quality of life	3	0.16 [-0.06, 0.39]	65%	5	0.10 [-0.22, 0.41]	65%
Clinical endpoints	3	-0.29 [-0.61, 0.02]	61%	8	-0.20 [-0.53, 0.12]	90%
Behaviour change	2	0.32 [-0.20, 0.84]	91%	4	-0.14 [-0.32, 0.05]	14%
Functioning	5	0.03 [-0.15, 0.20]	41%	8	0.04 [-0.09, 0.17]	45%
Psychological distress (depression)	6	-0.11 [-0.34, 0.12]	57%	15	**-0.58 [-0.94, -0.21]**	95%
Psychological distress (anxiety)	4	-0.10 [-0.19, 0.00]	0%	5	-0.17 [-0.48, 0.13]	63%
Emotional wellbeing	1	0.35 [-0.01, 0.71]	N/A	6	0.30 [-0.01, 0.61]	87%
**Maintenance**						
Functioning	1	0.08 [-0.19, 0.35]	N/A	6	0.03 [-0.07, 0.13]	0%
Psychological distress (depression)	1	**-0.15 [-0.26, -0.04]**	N/A	7	0.05 [-0.42, 0.51]	94%

For peer review only

Database Search Strategy for Systematic Review (MEDLINE, Embase, PsychInfo, CINAHL)

Multiple long-term conditions search strategy

Adapted from Smith et al. (2021)

1 Comorbidity/

2 (comorbid$ or co-morbid$).ti,ab.

3 (multimorbid$ or multi-morbid$).ti,ab.

4 (multidisease? or multi-disease? or (multiple adj (ill$ or disease? or condition? or syndrom$ or disorder?))).ti,ab.

5 Long term condition.ti,ab.

6 Chronic disease/

7 (multiple chronic$ adj3 (disease? or ill$ or care or condition? or disorder$ or health$ or syndrom$ or symptom$)).ti,ab.

8 ((coocur$ or co-ocur$ or coexist$ or co-exist$ or multipl$) adj3 (disease? or ill$ or care or condition? or disorder$ or health$ or medication$ or symptom$ or syndrom$)).ti,ab.

9 or/1-8

10 exp diabetes mellitus/ or diabet$.ti,ab.

11 exp hypertension/ or (hypertens$ or "high blood pressure?").ti,ab.

12 exp heart diseases/ or (((heart or cardiac or cardiovascular or coronary) adj (disease? or disorder? or failure)) or arrythmia?).ti,ab.

13 exp cerebrovascular disorders/ or ((cerebrovascular or vascular or carotoid$ or arter$) adj (disorder? or disease?)).ti,ab.

14 exp asthma/ or asthma$.ti,ab.

15 exp pulmonary disease chronic obstructive/ or (copd or (pulmonary adj2 (disease? or disorder?))).ti,ab.

16 exp hyperlipidemia/ or (hyperlipidem$ or Hypercholesterolemia$ or hypertriglyceridemia$).ti,ab.

17 exp Thyroid diseases/ or ((thyroid adj (disease? or disorder)) or hyperthyroid$ or hypothyroid$).ti,ab.

18 exp arthritis rheumatoid/ or rheumatoid arthritis.ti,ab.

19 exp mental disorders/ or (((mental or anxiety or panic or mood or psychological) adj (disease? or disorder?)) or (depression or bipolar or schizophren$ or psychos$)).ti,ab.

20 exp epilepsy/ or (epileps$ or seizure?).ti,ab.

21 exp hiv infections/ or (HIV or acquired immune$ deficiency syndrome? or (aids adj (associated or related or arteritis))).ti,ab.

22 exp neoplasms/ or (neoplasm? or cancer?).ti,ab.

For peer review only

23 exp kidney diseases/ or (kidney adj (disease? or disorder?)).ti,ab.

24 exp liver diseases/ or (liver adj (disease? or disorder?)).ti,ab.

25 exp osteoporosis/ or osteoporosis.ti,ab.

26 exp dementia/ or dementia.ab,ti.

27 exp stroke/ or stroke.ab,ti.

28 exp multiple sclerosis/ or (multiple sclerosis).ab,ti.

29 exp Parkinson disease/ or Parkinson*.ab,ti.

30 exp motor neuron disease/ or (motor neuron disease).ab,ti.

31 exp neurodegenerative diseases/ or neurodegenerative.ab,ti.

32 or/10-31

33 9 and 32

Behaviour change intervention search strategy

Adapted from Gardener et al. (2017)

1 (self car*) OR (self-manag*).mp.

2 behavio* adj3 chang*.mp.

3 (health education) OR (structure* education).mp.

4 secondary prevent*.mp.

5 (case manag*) OR (care manag*).mp.

6 (care navigat*) OR (community navigat*) OR (care co-ordinat*).mp.

7 (shared care) OR (collaborative care).mp.

8 (complex intervention*) OR (multicomponent intervention) OR (multiple intervention component).mp.

9 (integrated adj (model OR care)).mp.

10 ((Multiprofessional AND intervention$) OR (Interprofessional AND intervention$)).mp.

11 (comprehensive care) OR (comprehensive health care).mp.

12 (prevent* adj3 hospital*).mp.

13 or/1-12

RCT search strategy

Cochrane RCT from Smith et al. (2021)

1 randomi?ed controlled trial.pt.

2 controlled clinical trial.pt.

For peer review only

3 random$.ti,ab.

4 (control$ adj2 (trial? or study or studies)).ti,ab.

5 double-blind method/ or random allocation/ or single-blind method/

6 ((double or single or triple or treble) adj2 blind$).ti,ab.

7 or/1-6

8 exp animals/ not humans.sh.

9 7 not 8

Web of Science Search Strategy for Systematic Review

1 comorbid* or co-morbid* or multimorbid* or multi-morbid* or multidisease? or multi-disease? or (multiple NEAR/1 (illness* or disease* or condition* or syndrome* or disorder*)) or “Long term condition*” or “chronic disease” or (“multiple chronic*” NEAR/3 (disease? or illness* or care or condition? or disorder* or health* or syndrome* or symptom*)) or ((coocur* or co-occur* or coexist* or co-exist* or multipl*) NEAR/3 (disease? or illness* or care or condition? or disorder* or health* or medication* or symptom* or syndrome*))

2 diabe* or hypertens* or “high blood pressure” or (((heart or cardiac or cardiovascular or coronary) NEAR/3 (disease? or disorder? or failure)) or arrythmia?) or ((cerebrovascular or vascular or carotoid* or arter*) NEAR/3 (disorder? or disease?)) or asthma* or copd or (pulmonary NEAR/2 (disease? or disorder?)) or hyperlipidem* or Hypercholesterolemia* or hypertriglyceridemia* or (thyroid NEAR/1 (disease? or disorder)) or hyperthyroid* or hypothyroid* or “rheumatoid arthritis” or (((mental or anxiety or panic or mood or psychological) NEAR/1 (disease? or disorder?)) or (depression or bipolar or schizophren* or psychos*)) or epilepsy* or seizure? or HIV or “acquired immune* deficiency syndrome?” or (aids NEAR/1 (associated or related or arteritis)) or neoplasm? or cancer? or (kidney NEAR/1 (disease? or disorder?)) or (liver NEAR/1 (disease? or disorder?)) or osteoporosis or dementia or stroke or “multiple sclerosis” or Parkinson* or “motor neuron disease” or neurodegenerative

3 1 AND 2

4 “self car*” OR self-manag* or behavio* NEAR/3 chang* or “health education” OR “structure* education” or “secondary prevent*” or “case manag*” OR “care manag*” or “care navigat*” OR “community navigat*” OR “care co-ordinat*” or “shared care” OR “collaborative care” or “complex intervention*” OR “multicomponent intervention” OR “multiple intervention component” or (integrated NEAR/1 (model OR care)) or ((Multiprofessional AND intervention*) OR (Interprofessional AND intervention*)) or “comprehensive care” OR “comprehensive health care” or (prevent* NEAR/3 hospital*)

5 ((randomised OR randomized OR randomisation OR randomisation OR (random* AND (allocat* OR assign*)) OR (blind* AND (single OR double OR treble OR triple))))

6 ((animal* AND model* OR mouse OR mice))

7 5 NOT 6

#3 AND #4 AND #7

For peer review only

1. Ali MK, Chwastiak L, Poongothai S, Emmert-Fees KMF, Patel SA, Anjana RM, et al. Effect of a Collaborative Care Model on Depressive Symptoms and Glycated Hemoglobin, Blood Pressure, and Serum Cholesterol among Patients with Depression and Diabetes in India: The INDEPENDENT Randomized Clinical Trial. JAMA - Journal of the American Medical Association. 2020 Aug 18;324(7):651–62.

2. Aragonès E, Rambla C, López-Cortacans G, Tomé-Pires C, Sánchez-Rodríguez E, Caballero A, et al. Effectiveness of a collaborative care intervention for managing major depression and chronic musculoskeletal pain in primary care: A cluster-randomised controlled trial. J Affect Disord. 2019 Jun 1;252:221–9.

3. Barley EA, Walters P, Haddad M, Phillips R, Achilla E, McCrone P, et al. The UPBEAT nurse-delivered personalized care intervention for people with coronary heart disease who report current chest pain and depression: A randomised controlled pilot study. PLoS One. 2014 Jun 5;9(6).

4. Barrowclough C, Haddock G, Tarrier N, Shôn Lewis FbpW, Jan Moring Frcp, Nichola Schofield B, et al. Article Randomized Controlled Trial of Motivational Interviewing, Cognitive Behavior Therapy, and Family Intervention for Patients With Comorbid Schizophrenia and Substance Use Disorders. Vol. 158, Am J Psychiatry. 2001.

5. Barr Taylor C, Houston Miller N, Reilly KR, Greenwald G, Cunning D, Deeter A, et al. Evaluation of a Nurse-Care Management System to Improve Outcomes in Patients With Complicated Diabetes [Internet]. 2003. Available from: http://diabetesjournals.org/care/article-pdf/26/4/1058/659413/dc0403001058.pdf

6. Battersby MW, Beattie J, Pols RG, Smith DP, Condon J, Blunden S. A randomised controlled trial of the Flinders ProgramTM of chronic condition management in Vietnam veterans with co-morbid alcohol misuse, and psychiatric and medical conditions. Australian and New Zealand Journal of Psychiatry. 2013 May;47(5):451–62.

7. Blank MB, Hanrahan NP, Fishbein M, Wu ES, Tennille JA, Ten Have TR, et al. A randomized trial of a nursing intervention for HIV disease management among persons with serious mental illness. Psychiatric Services. 2011 Nov 1;62(11):1318–24.

8. Boeschoten RE, Dekker J, Uitdehaag BMJ, Beekman ATF, Hoogendoorn AW, Collette EH, et al. Internet-based treatment for depression in multiple sclerosis: A randomized controlled trial. Multiple Sclerosis. 2017 Jul 1;23(8):1112–22.

9. Bogner HR, De Vries HF. Integration of depression and hypertension treatment: A pilot, randomized controlled trial. Ann Fam Med. 2008;6(4):295–301.

10. Chwastiak LA, Luongo M, Russo J, Johnson L, Lowe JM, Hoffman G, et al. Use of a mental health center collaborative care team to improve diabetes care and outcomes for patients with psychosis. Psychiatric Services. 2018 Mar 1;69(3):349–52.

11. Cummings DM, Lutes LD, Littlewood K, Solar C, Carraway M, Kirian K, et al. Randomized trial of a tailored cognitive behavioral intervention in type 2 diabetes with comorbid depressive and/or regimen-related distress symptoms: 12-month outcomes from. In: Diabetes Care. American Diabetes Association Inc.; 2019. p. 841–8.

12. Druss BG, Singh M, Von Esenwein SA, Glick GE, Tapscott S, Tucker SJ, et al. Peer-led self-management of general medical conditions for patients with serious mental illnesses: A randomized trial. Psychiatric Services. 2018 May 1;69(5):529–35.

For peer review only

13. Ell K, Katon W, Xie B, Lee PJ, Kapetanovic S, Guterman J, et al. Collaborative care management of major depression among low-income, predominantly hispanic subjects with diabetes: A randomized controlled trial. Diabetes Care. 2010 Apr;33(4):706–13.

14. Ell K, Aranda MP, Wu S, Oh H, Lee PJ, Guterman J. Promotora assisted depression and self-care management among predominantly Latinos with concurrent chronic illness: Safety net care system clinical trial results. Contemp Clin Trials. 2017 Oct 1;61:1–9.

15. Fisher K, Markle-Reid M, Ploeg J, Bartholomew A, Griffith LE, Gafni A, et al. Self-management program versus usual care for community-dwelling older adults with multimorbidity: A pragmatic randomized controlled trial in Ontario, Canada. J Comorb. 2020 Jan 1;10:2235042X2096339.

16. Garvey J, Connolly D, Boland F, Smith SM. OPTIMAL, an occupational therapy led self-management support programme for people with multimorbidity in primary care: A randomized controlled trial. BMC Fam Pract. 2015 Dec 12;16(1).

17. Goldberg RW, Dickerson F, Lucksted A, Brown CH, Weber E, Tenhula WN, et al. Living Well: An intervention to improve self-management of medical illness for individuals with serious mental illness. Psychiatric Services. 2013 Jan 1;64(1):51–7.

18. Goorden M, van der Feltz-Cornelis CM, van Steenbergen-Weijenburg KM, Horn EK, Beekman ATF, Hakkaar-Van Roijen L. Cost-utility of collaborative care for the treatment of comorbid major depressive disorder in outpatients with chronic physical conditions. A randomized controlled trial in the general hospital setting (CC-DIM). Neuropsychiatr Dis Treat. 2017 Jul 18;13:1881–93.

19. Griva K, Rajeswari M, Nandakumar M, Khoo EYH, Lee VYW, Chua CG, et al. The combined diabetes and renal control trial (C-DIRECT) - A feasibility randomised controlled trial to evaluate outcomes in multi-morbid patients with diabetes and on dialysis using a mixed methods approach. BMC Nephrol. 2019 Jan 3;20(1).

20. Guo Y, Lane DA, Wang L, Zhang H, Wang H, Zhang W, et al. Mobile Health Technology to Improve Care for Patients With Atrial Fibrillation. J Am Coll Cardiol. 2020 Apr 7;75(13):1523–34.

21. Hernandez-Quiles C, Bernabeu-Wittel M, Barón-Franco B, Palacios AA, Garcia-Serrano MR, Lopez-Jimeno W, et al. A randomized clinical trial of home telemonitoring in patients with advanced heart and lung diseases. J Telemed Telecare. 2021;

22. House A, Bryant L, Russell AM, Wright-Hughes A, Graham L, Walwyn R, et al. Randomized controlled feasibility trial of supported self-management in adults with Type 2 diabetes mellitus and an intellectual disability: OK Diabetes. Diabetic Medicine. 2018 Jun 1;35(6):776–88.

23. Jackson IL, Ukwe C V. Clinical outcomes of pharmaceutical care intervention in HIV positive patients with hypertension: A randomized controlled study. J Clin Pharm Ther. 2021 Aug 1;46(4):1083–94.

24. Kamradt M, Ose D, Krisam J, Jacke C, Salize HJ, Besier W, et al. Meeting the needs of multimorbid patients with Type 2 diabetes mellitus – A randomized controlled trial to assess the impact of a care management intervention aiming to improve self-care. In: Diabetes Research and Clinical Practice. Elsevier Ireland Ltd; 2019. p. 184–93.

25. Kanwal F, Pyne JM, Tavakoli-Tabasi S, Nicholson S, Dieckgraefe B, Storay E, et al. A Randomized Trial of Off-Site Collaborative Care for Depression in Chronic Hepatitis C Virus. Health Serv Res. 2018 Aug 1;53(4):2547–66.

For peer review only

26. Katon WJ, Michael ;, Korff V, Lin EHB, Simon G, Ludman E, et al. The Pathways Study A Randomized Trial of Collaborative Care in Patients With Diabetes and Depression. 2004.

27. Katon WJ, Lin EH, Von Korff M, Ciechanowski P, Ludman EJ, Young B, et al. Collaborative Care for Patients with Depression and Chronic Illnesses From the Departments of Psychiatry A bs t r ac t. Vol. 363, N Engl J Med. 2010.

28. Khunti K, Highton PJ, Waheed G, Dallosso H, Redman E, Batt ME, et al. Promoting physical activity with self-management support for those with multimorbidity: A randomised controlled trial. British Journal of General Practice. 2021 Dec 1;71(713):E921–30.

29. Koesoemadinata RC, McAllister SM, Soetedjo NNM, Santoso P, Ruslami R, Damayanti H, et al. Educational counselling of patients with combined TB and diabetes mellitus: a randomised trial. Public Health Action. 2021 Dec 18;11(4):202–8.

30. Kroenke K, Bair MJ, Damush TM, Wu J, Hoke S, Sutherland J, et al. Optimized Antidepressant Therapy and Pain Self-management in Primary Care Patients With Depression and Musculoskeletal Pain A Randomized Controlled Trial [Internet]. 2009. Available from: https://jamanetwork.com/

31. Lear SA, Norena M, Banner D, Whitehurst DGT, Gill S, Burns J, et al. Assessment of an Interactive Digital Health-Based Self-management Program to Reduce Hospitalizations among Patients with Multiple Chronic Diseases: A Randomized Clinical Trial. JAMA Netw Open. 2021 Dec 28;4(12).

32. Lenferink A, van der Palen J, van der Valk PDLPM, Cafarella P, van Veen A, Quinn S, et al. Exacerbation action plans for patients with COPD and comorbidities: A randomised controlled trial. European Respiratory Journal. 2019 Nov 1;54(5).

33. Markle-Reid M, Ploeg J, Fraser KD, Fisher KA, Bartholomew A, Griffith LE, et al. Community Program Improves Quality of Life and Self-Management in Older Adults with Diabetes Mellitus and Comorbidity. J Am Geriatr Soc. 2018 Feb 1;66(2):263–73.

34. McDermott RA, Schmidt B, Preece C, Owens V, Taylor S, Li M, et al. Community health workers improve diabetes care in remote Australian indigenous communities: Results of a pragmatic cluster randomized controlled trial. BMC Health Serv Res. 2015 Feb 19;15(1).

35. Merlin JS, Westfall AO, Long D, Davies S, Saag M, Demonte W, et al. A Randomized Pilot Trial of a Novel Behavioral Intervention for Chronic Pain Tailored to Individuals with HIV. AIDS Behav. 2018 Aug 1;22(8):2733–42.

36. Miklavcic JJ, Fraser KD, Ploeg J, Markle-Reid M, Fisher K, Gafni A, et al. Effectiveness of a community program for older adults with type 2 diabetes and multimorbidity: A pragmatic randomized controlled trial. BMC Geriatr. 2020 May 13;20(1).

37. Morgan MAJ, Coates MJ, Dunbar JA, Reddy P, Schlicht K, Fuller J. The TrueBlue model of collaborative care using practice nurses as case managers for depression alongside diabetes or heart disease: a randomised trial. BMJ Open [Internet]. 2013; Available from: http://bmjopen.bmj.com/

38. Naik AD, Hundt NE, Vaughan EM, Petersen NJ, Zeno D, Kunik ME, et al. Effect of Telephone-Delivered Collaborative Goal Setting and Behavioral Activation vs Enhanced Usual Care for Depression among Adults with Uncontrolled Diabetes: A Randomized Clinical Trial. JAMA Netw Open. 2019 Aug 7;2(8).

For peer review only

39. Ose D, Kamradt M, Kiel M, Freund T, Besier W, Mayer M, et al. Care management intervention to strengthen self-care of multimorbid patients with type 2 diabetes in a German primary care network: A randomized controlled trial. PLoS One. 2019 Jun 1;14(6).

40. O'Toole L, Connolly D, Boland F, Smith SM. Effect of the OPTIMAL programme on self-management of multimorbidity in primary care: A randomised controlled trial. British Journal of General Practice. 2021 Apr 1;71(705):E303–11.

41. Park YH, Chang HK. Effect of a health coaching self-management program for older adults with multimorbidity in nursing homes. Patient Prefer Adherence. 2014 Jul 4;8:959–70.

42. Pibernik-Okanovic M, Begic D, Ajdukovic D, Andrijasevic N, Metelko Z. Psychoeducation versus treatment as usual in diabetic patients with subthreshold depression: Preliminary results of a randomized controlled trial. Trials. 2009 Aug 26;10.

43. Rose L, Istanboulian L, Carriere L, Thomas A, Lee HB, Rezaie S, et al. Program of integrated care for patients with chronic obstructive pulmonary disease and multiple comorbidities (PIC COPD+): A randomised controlled trial. European Respiratory Journal. 2018;51(1).

44. Ross CJM, Davis TMA, MacDonald GF. Cognitive-behavioral treatment combined with asthma education for adults with asthma and coexisting panic disorder. Clin Nurs Res. 2005 May;14(2):131–57.

45. Sajatovic M, Gunzler DD, Kanuch SW, Cassidy KA, Tatsuoka C, McCormick R, et al. A 60-week prospective RCT of a self-management intervention for individuals with serious mental illness and diabetes mellitus. Psychiatric Services. 2017 Sep 1;68(9):883–90.

46. Salisbury C, Man MS, Bower P, Guthrie B, Chaplin K, Gaunt DM, et al. Management of multimorbidity using a patient-centred care model: a pragmatic cluster-randomised trial of the 3D approach. The Lancet. 2018 Jul 7;392(10141):41–50.

47. Schneider KL, Panza E, Handschin B, Ma Y, Busch AM, Waring ME, et al. Feasibility of Pairing Behavioral Activation With Exercise for Women With Type 2 Diabetes and Depression: The Get It Study Pilot Randomized Controlled Trial [Internet]. 2015. Available from: www.elsevier.com/locate/bt

48. Takahashi PY, Pecina JL, Upatising B, Chaudhry R, Shah ND, Van Houten H, et al. A randomized controlled trial of telemonitoring in older adults with multiple health issues to prevent hospitalizations and emergency department visits. Arch Intern Med. 2012 May 28;172(10):773–9.

49. Vera M, Perez-Pedrogo C, Huertas SE, Reyes-Rabanillo ML, Juarbe D, Huertas A, et al. Collaborative Care for Depressed Patients With Chronic Medical Conditions: A Randomized Trial in Puerto Rico. Psychiatric Services. 2010 Feb 1;61(2).

50. Wakefield BJ, Holman JE, Ray A, Scherubel M, Adams MR, Hillis SL, et al. Effectiveness of home telehealth in comorbid diabetes and hypertension: A randomized, controlled trial. Telemedicine and e-Health. 2011 May 1;17(4):254–61.

51. Williams A, Manias E, Walker R, Gorelik A. A multifactorial intervention to improve blood pressure control in co-existing diabetes and kidney disease: A feasibility randomized controlled trial. J Adv Nurs. 2012 Nov;68(11):2515–25.

52. Wilson M, Hewes C, Barbosa-Leiker C, Mason A, Wuestney KA, Shuen JA, et al. Engaging Adults With Chronic Disease in Online Depressive Symptom Self-Management. West J Nurs Res. 2018 Jun 1;40(6):834–53.

For peer review only

53. Yu C, Choi D, Bruno BA, Thorpe KE, Straus SE, Cantarutti P, et al. Impact of mydiabetesplan, a web-based patient decision aid on decisional conflict, diabetes distress, quality of life, and chronic illness care in patients with diabetes: Cluster randomized controlled trial. J Med Internet Res. 2020 Sep 29;22(9).

For peer review only

Supplementary figure 3

Table 2 significant effects forest plots

Psychological distress (depression) – post-intervention overall effect

Study or Subgroup	Intervention Mean	Intervention SD	Intervention Total	Control Mean	Control SD	Control Total	Weight	Std. Mean Difference IV, Random, 95% CI
Ali 2020	0.52	0.38	192	0.82	0.54	202	5.2%	-0.64 [-0.84, -0.44]
Aragones 2019	1.29	0.77	167	1.42	0.73	161	5.2%	-0.17 [-0.39, 0.04]
Barley 2014	10.3	3.8	35	9.2	4.6	39	4.7%	0.26 [-0.20, 0.71]
Boeschoten 2017	20.3	8.8	74	21	9.1	78	5.0%	-0.08 [-0.40, 0.24]
Bogner 2008	9.9	10.7	32	19.3	15.2	32	4.5%	-0.71 [-1.21, -0.20]
Cummings 2019	6.3	5.9	67	7.9	7	72	5.0%	-0.25 [-0.58, 0.09]
Ell 2017	1.02	0.07	178	1.15	0.07	170	5.1%	-1.85 [-2.10, -1.60]
Garvey 2015	6.32	4.19	22	7.82	3.83	22	4.3%	-0.37 [-0.96, 0.23]
House 2018	2.4	1.87	30	1.1	1.8	29	4.5%	0.70 [0.17, 1.23]
Katon 2010	0.83	0.68	105	1.14	0.66	106	5.1%	-0.46 [-0.73, -0.19]
Kroenke 2009	1.14	0.69	123	1.69	0.74	127	5.1%	-0.77 [-1.02, -0.51]
Markle-Reid 2018	4.67	4.83	71	5.71	5.24	65	5.0%	-0.21 [-0.54, 0.13]
Miklavcic 2020	4.94	4.46	63	5.82	4.93	50	4.9%	-0.19 [-0.56, 0.19]
Morgan 2013	7.1	0.8	164	9	0.9	146	5.1%	-2.23 [-2.52, -1.95]
Naik 2019	10.9	6.1	107	12.4	6	80	5.0%	-0.25 [-0.54, 0.04]
Park 2014	5.3	3.4	22	7.6	5.8	21	4.3%	-0.48 [-1.09, 0.13]
Ross 2005	11.8	9.93	15	16.78	7.89	9	3.6%	-0.52 [-1.36, 0.32]
Sajatovic 2017	15.92	10	100	18.55	8.8	100	5.1%	-0.28 [-0.56, 0.00]
Salisbury 2018	6.1	4.4	654	6.6	4.5	641	5.3%	-0.11 [-0.22, -0.00]
Schneider 2016	12.5	14.8	15	12.3	4.1	14	3.9%	0.02 [-0.71, 0.75]
Wilson 2018	17.46	6.16	22	20.5	5.3	25	4.3%	-0.52 [-1.11, 0.06]
**Total (95% CI)**			**2258**			**2189**	**100.0%**	**-0.45 [-0.73, -0.16]**

Heterogeneity: Tau² = 0.38; Chi² = 366.51, df = 20 (P < 0.00001); I² = 95%
Test for overall effect: Z = 3.11 (P = 0.002)

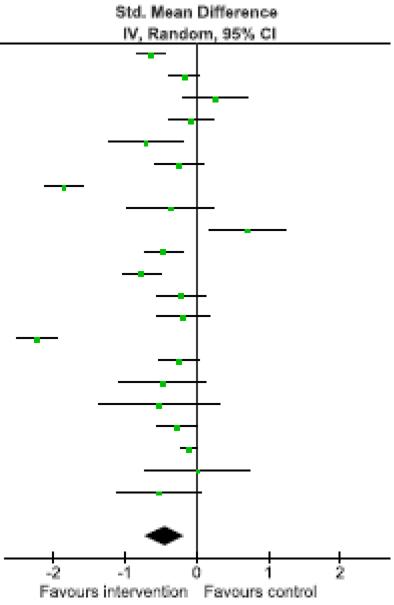

Psychological distress (anxiety) – post-intervention overall effect

Study or Subgroup	Intervention Mean	Intervention SD	Intervention Total	Control Mean	Control SD	Control Total	Weight	Std. Mean Difference IV, Random, 95% CI
Barley 2014	11	5	35	10.4	5	39	7.5%	0.12 [-0.34, 0.58]
Battersby 2013	48.95	10.39	44	47	13.75	28	7.1%	0.16 [-0.31, 0.64]
Boeschoten 2017	9	3.8	74	9.3	3.8	78	12.4%	-0.08 [-0.40, 0.24]
Garvey 2015	9.5	4.71	22	9.09	4.96	22	4.9%	0.08 [-0.51, 0.67]
Kroenke 2009	5.8	5	123	8	5.1	127	16.2%	-0.43 [-0.69, -0.18]
Markle-Reid 2018	2.34	2.92	77	2.71	3.27	66	11.9%	-0.12 [-0.45, 0.21]
Miklavcic 2020	2.37	3.32	63	3.24	3.68	50	10.1%	-0.25 [-0.62, 0.12]
Ross 2005	31.73	22.29	15	57.56	31.56	9	2.4%	-0.96 [-1.84, -0.08]
Salisbury 2018	5.7	4.6	652	6.1	4.7	638	27.4%	-0.09 [-0.20, 0.02]
**Total (95% CI)**			**1105**			**1057**	**100.0%**	**-0.14 [-0.28, 0.00]**

Heterogeneity: Tau² = 0.02; Chi² = 13.27, df = 8 (P = 0.10); I² = 40%
Test for overall effect: Z = 1.95 (P = 0.05)

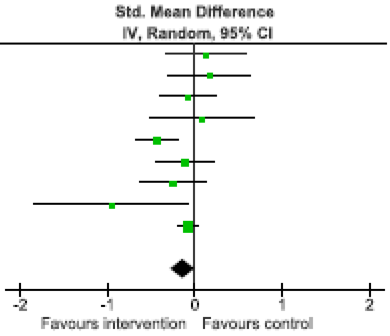

Emotional wellbeing – post-intervention overall effect

Study or Subgroup	Intervention Mean	Intervention SD	Intervention Total	Control Mean	Control SD	Control Total	Weight	Std. Mean Difference IV, Random, 95% CI
Barley 2014	32.4	11.2	35	35.4	11.8	39	11.9%	-0.26 [-0.72, 0.20]
Druss 2018	34.49	11.15	198	34.25	11.97	202	16.4%	0.02 [-0.18, 0.22]
Ell 2010	47.31	11.48	193	43.6	12.46	194	16.3%	0.31 [0.11, 0.51]
Ell 2017	44.54	1.03	178	43.67	1.02	170	16.0%	0.85 [0.63, 1.07]
Goldberg 2013	50.5	11.1	28	43.2	10.7	30	10.7%	0.66 [0.13, 1.19]
Miklavcic 2020	56.69	8.37	65	53.88	7.67	56	13.6%	0.35 [-0.01, 0.71]
Sajatovic 2017	42.05	11.1	100	39.58	11.4	100	15.1%	0.22 [-0.06, 0.50]
**Total (95% CI)**			**797**			**791**	**100.0%**	**0.31 [0.04, 0.58]**

Heterogeneity: Tau² = 0.11; Chi² = 39.32, df = 6 (P < 0.00001); I² = 85%
Test for overall effect: Z = 2.23 (P = 0.03)

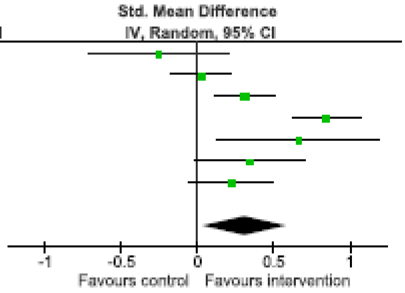

Emotional wellbeing – maintenance overall effect

Study or Subgroup	Intervention Mean	Intervention SD	Intervention Total	Control Mean	Control SD	Control Total	Weight	Std. Mean Difference IV, Random, 95% CI
Barley 2014	34.5	11.6	32	33.6	12.5	37	10.5%	0.07 [-0.40, 0.55]
Druss 2018	36.64	12.28	198	34.54	11.82	202	18.7%	0.17 [-0.02, 0.37]
Ell 2010	45.1	12.19	193	43.49	11.66	194	18.6%	0.13 [-0.06, 0.33]
Ell 2017	31.24	0.87	178	30.59	0.89	170	18.0%	0.74 [0.52, 0.95]
Goldberg 2013	46.9	10.7	28	43.9	11.5	29	9.4%	0.27 [-0.26, 0.79]
Griva 2019	2.38	0.72	20	2.15	0.63	24	7.9%	0.34 [-0.26, 0.93]
Kanwal 2018	41.8	13.8	114	39.4	11.9	128	16.9%	0.19 [-0.07, 0.44]
**Total (95% CI)**			**763**			**784**	**100.0%**	**0.28 [0.07, 0.49]**

Heterogeneity: Tau² = 0.05; Chi² = 21.42, df = 6 (P = 0.002); I² = 72%
Test for overall effect: Z = 2.63 (P = 0.009)

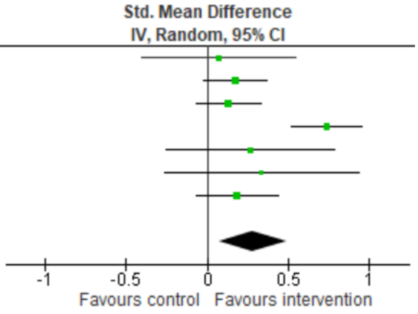

For peer review only

Table 3 significant effects forest plots

Quality of life – post-intervention by intervention type

Study or Subgroup	Intervention Mean	Intervention SD	Intervention Total	Control Mean	Control SD	Control Total	Weight	Std. Mean Difference IV, Random, 95% CI
**1.1.1 Self-management**								
Battersby 2013	0.38	0.19	44	0.43	0.22	28	8.6%	-0.24 [-0.72, 0.23]
Garvey 2015	65.73	20.18	22	50.5	16.3	22	5.9%	0.82 [0.20, 1.43]
**Subtotal (95% CI)**			**66**			**50**	**14.5%**	**0.27 [-0.77, 1.30]**
Heterogeneity: Tau² = 0.48; Chi² = 7.11, df = 1 (P = 0.008); I² = 86%								
Test for overall effect: Z = 0.50 (P = 0.62)								
**1.1.2 Collaborative care**								
Goorden 2017	0.54	0.33	42	0.5	0.3	39	9.6%	0.13 [-0.31, 0.56]
Kamradt 2019	0.715	0.22	195	0.708	0.23	187	19.2%	0.03 [-0.17, 0.23]
Katon 2010	6	2.2	105	5.2	1.9	106	15.7%	0.39 [0.12, 0.66]
Salisbury 2018	0.566	0.294	699	0.526	0.306	684	23.9%	0.13 [0.03, 0.24]
**Subtotal (95% CI)**			**1041**			**1016**	**68.4%**	**0.15 [0.02, 0.27]**
Heterogeneity: Tau² = 0.01; Chi² = 4.33, df = 3 (P = 0.23); I² = 31%								
Test for overall effect: Z = 2.33 (P = 0.02)								
**1.1.3 Cognitive and behavioural activation**								
Boeschoten 2017	0.52	0.3	74	0.58	0.3	78	13.7%	-0.20 [-0.52, 0.12]
Ross 2005	5.07	1.2	15	4.25	1.13	9	3.5%	0.67 [-0.18, 1.53]
**Subtotal (95% CI)**			**89**			**87**	**17.1%**	**0.14 [-0.69, 0.98]**
Heterogeneity: Tau² = 0.27; Chi² = 3.53, df = 1 (P = 0.06); I² = 72%								
Test for overall effect: Z = 0.34 (P = 0.74)								
**Total (95% CI)**			**1196**			**1153**	**100.0%**	**0.13 [-0.04, 0.30]**
Heterogeneity: Tau² = 0.03; Chi² = 17.13, df = 7 (P = 0.02); I² = 59%								
Test for overall effect: Z = 1.54 (P = 0.12)								
Test for subgroup differences: Chi² = 0.05, df = 2 (P = 0.98), I² = 0%								

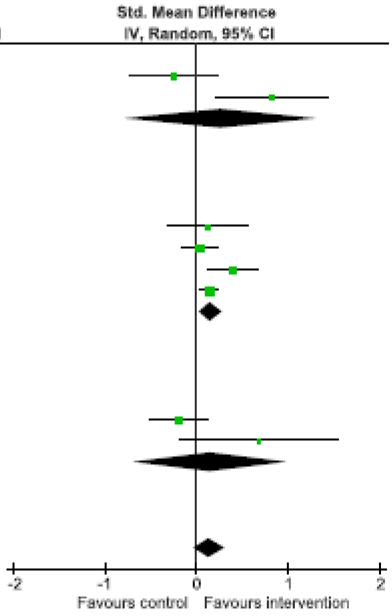

Psychological distress (depression) – post-intervention by intervention type

Study or Subgroup	Intervention Mean	Intervention SD	Intervention Total	Control Mean	Control SD	Control Total	Weight	Std. Mean Difference IV, Random, 95% CI
**1.10.1 Self-management**								
Aragones 2019	1.29	0.77	167	1.42	0.73	161	5.2%	-0.17 [-0.39, 0.04]
Barley 2014	10.3	3.8	35	9.2	4.6	39	4.7%	0.26 [-0.20, 0.71]
Ell 2017	1.02	0.07	178	1.15	0.07	170	5.1%	-1.85 [-2.10, -1.60]
Garvey 2015	6.32	4.19	22	7.82	3.83	22	4.3%	-0.37 [-0.96, 0.23]
House 2018	2.4	1.87	30	1.1	1.8	29	4.5%	0.70 [0.17, 1.23]
Markle-Reid 2018	4.67	4.83	71	5.71	5.24	65	5.0%	-0.21 [-0.54, 0.13]
Miklavcic 2020	4.94	4.46	63	5.82	4.93	50	4.9%	-0.19 [-0.56, 0.19]
Park 2014	5.3	3.4	22	7.6	5.8	21	4.3%	-0.48 [-1.09, 0.13]
**Subtotal (95% CI)**			**588**			**557**	**37.8%**	**-0.30 [-0.91, 0.31]**
Heterogeneity: Tau² = 0.72; Chi² = 155.89, df = 7 (P < 0.00001); I² = 96%								
Test for overall effect: Z = 0.96 (P = 0.34)								
**1.10.2 Collaborative care**								
Ali 2020	0.52	0.38	192	0.82	0.54	202	5.2%	-0.64 [-0.84, -0.44]
Bogner 2008	9.9	10.7	32	19.3	15.2	32	4.5%	-0.71 [-1.21, -0.20]
Katon 2010	0.83	0.68	105	1.14	0.66	106	5.1%	-0.46 [-0.73, -0.19]
Kroenke 2009	1.14	0.69	123	1.69	0.74	127	5.1%	-0.77 [-1.02, -0.51]
Morgan 2013	7.1	0.8	164	9	0.9	146	5.1%	-2.23 [-2.52, -1.95]
Salisbury 2018	6.1	4.4	654	6.6	4.5	641	5.3%	-0.11 [-0.22, -0.00]
**Subtotal (95% CI)**			**1270**			**1254**	**30.3%**	**-0.82 [-1.40, -0.24]**
Heterogeneity: Tau² = 0.50; Chi² = 197.36, df = 5 (P < 0.00001); I² = 97%								
Test for overall effect: Z = 2.76 (P = 0.006)								
**1.10.3 Cognitive and behavioural activation**								
Boeschoten 2017	20.3	8.8	74	21	9.1	78	5.0%	-0.08 [-0.40, 0.24]
Cummings 2019	6.3	5.9	67	7.9	7	72	5.0%	-0.25 [-0.58, 0.09]
Naik 2019	10.9	6.1	107	12.4	6	80	5.0%	-0.25 [-0.54, 0.04]
Ross 2005	11.8	9.93	15	16.78	7.89	9	3.6%	-0.52 [-1.36, 0.32]
Sajatovic 2017	15.92	10	100	18.55	8.8	100	5.1%	-0.28 [-0.56, 0.00]
Schneider 2016	12.5	14.8	15	12.3	4.1	14	3.9%	0.02 [-0.71, 0.75]
Wilson 2018	17.46	6.16	22	20.5	5.3	25	4.3%	-0.52 [-1.11, 0.06]
**Subtotal (95% CI)**			**400**			**378**	**31.9%**	**-0.24 [-0.38, -0.09]**
Heterogeneity: Tau² = 0.00; Chi² = 2.88, df = 6 (P = 0.82); I² = 0%								
Test for overall effect: Z = 3.25 (P = 0.001)								
**Total (95% CI)**			**2258**			**2189**	**100.0%**	**-0.45 [-0.73, -0.16]**
Heterogeneity: Tau² = 0.38; Chi² = 366.51, df = 20 (P < 0.00001); I² = 95%								
Test for overall effect: Z = 3.11 (P = 0.002)								
Test for subgroup differences: Chi² = 3.64, df = 2 (P = 0.16), I² = 45.1%								

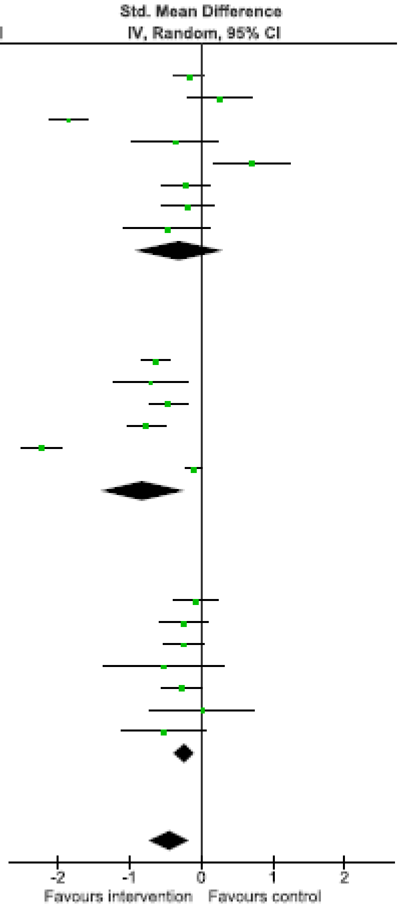

Emotional wellbeing – post-intervention by intervention type

Study or Subgroup	Intervention Mean	Intervention SD	Intervention Total	Control Mean	Control SD	Control Total	Weight	Std. Mean Difference IV, Random, 95% CI
**1.8.1 Self-management**								
Barley 2014	32.4	11.2	35	35.4	11.8	39	11.9%	-0.26 [-0.72, 0.20]
Druss 2018	34.49	11.15	198	34.25	11.97	202	16.4%	0.02 [-0.18, 0.22]
Ell 2017	44.54	1.03	178	43.67	1.02	170	16.0%	0.85 [0.63, 1.07]
Goldberg 2013	50.5	11.1	28	43.2	10.7	30	10.7%	0.66 [0.13, 1.19]
Miklavcic 2020	56.69	8.37	65	53.88	7.67	56	13.6%	0.35 [-0.01, 0.71]
**Subtotal (95% CI)**			**504**			**497**	**68.6%**	**0.33 [-0.10, 0.76]**
Heterogeneity: Tau² = 0.21; Chi² = 38.60, df = 4 (P < 0.00001); I² = 90%								
Test for overall effect: Z = 1.49 (P = 0.14)								
**1.8.2 Collaborative Care**								
Ell 2010	47.31	11.48	193	43.6	12.46	194	16.3%	0.31 [0.11, 0.51]
**Subtotal (95% CI)**			**193**			**194**	**16.3%**	**0.31 [0.11, 0.51]**
Heterogeneity: Not applicable								
Test for overall effect: Z = 3.02 (P = 0.003)								
**1.8.3 Cognitive and behavioural activiation**								
Sajatovic 2017	42.05	11.1	100	39.58	11.4	100	15.1%	0.22 [-0.06, 0.50]
**Subtotal (95% CI)**			**100**			**100**	**15.1%**	**0.22 [-0.06, 0.50]**
Heterogeneity: Not applicable								
Test for overall effect: Z = 1.54 (P = 0.12)								
**Total (95% CI)**			**797**			**791**	**100.0%**	**0.31 [0.04, 0.58]**
Heterogeneity: Tau² = 0.11; Chi² = 39.32, df = 6 (P < 0.00001); I² = 85%								
Test for overall effect: Z = 2.23 (P = 0.03)								
Test for subgroup differences: Chi² = 0.31, df = 2 (P = 0.86), I² = 0%								

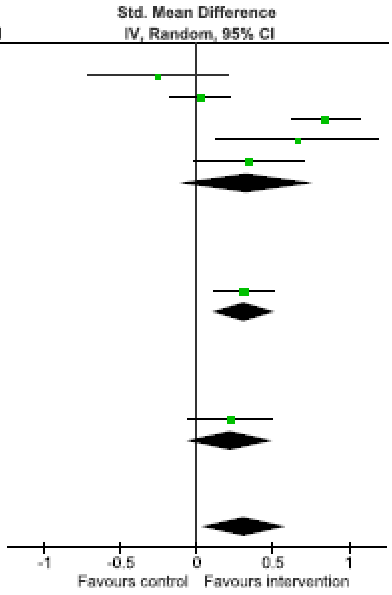

Emotional wellbeing – maintenance by intervention type

Study or Subgroup	Intervention Mean	Intervention SD	Intervention Total	Control Mean	Control SD	Control Total	Weight	Std. Mean Difference IV, Random, 95% CI
**1.9.1 Self-management**								
Barley 2014	34.5	11.6	32	33.6	12.5	37	10.5%	0.07 [-0.40, 0.55]
Druss 2018	36.64	12.28	198	34.54	11.82	202	18.7%	0.17 [-0.02, 0.37]
Ell 2017	31.24	0.87	178	30.59	0.89	170	18.0%	0.74 [0.52, 0.95]
Goldberg 2013	46.9	10.7	28	43.9	11.5	29	9.4%	0.27 [-0.26, 0.79]
Griva 2019	2.38	0.72	20	2.15	0.63	24	7.9%	0.34 [-0.26, 0.93]
**Subtotal (95% CI)**			**456**			**462**	**64.5%**	**0.34 [0.03, 0.65]**
Heterogeneity: Tau² = 0.09; Chi² = 16.42, df = 4 (P = 0.003); I² = 76%								
Test for overall effect: Z = 2.14 (P = 0.03)								
**1.9.2 Collaborative care**								
Ell 2010	45.1	12.19	193	43.49	11.66	194	18.6%	0.13 [-0.06, 0.33]
Kanwal 2018	41.8	13.8	114	39.4	11.9	128	16.9%	0.19 [-0.07, 0.44]
**Subtotal (95% CI)**			**307**			**322**	**35.5%**	**0.15 [-0.00, 0.31]**
Heterogeneity: Tau² = 0.00; Chi² = 0.10, df = 1 (P = 0.75); I² = 0%								
Test for overall effect: Z = 1.93 (P = 0.05)								
**1.9.3 Cognitive and behavioural activiation**								
**Subtotal (95% CI)**			**0**			**0**		**Not estimable**
Heterogeneity: Not applicable								
Test for overall effect: Not applicable								
**Total (95% CI)**			**763**			**784**	**100.0%**	**0.28 [0.07, 0.49]**
Heterogeneity: Tau² = 0.05; Chi² = 21.42, df = 6 (P = 0.002); I² = 72%								
Test for overall effect: Z = 2.63 (P = 0.009)								
Test for subgroup differences: Chi² = 1.09, df = 1 (P = 0.30), I² = 7.9%								

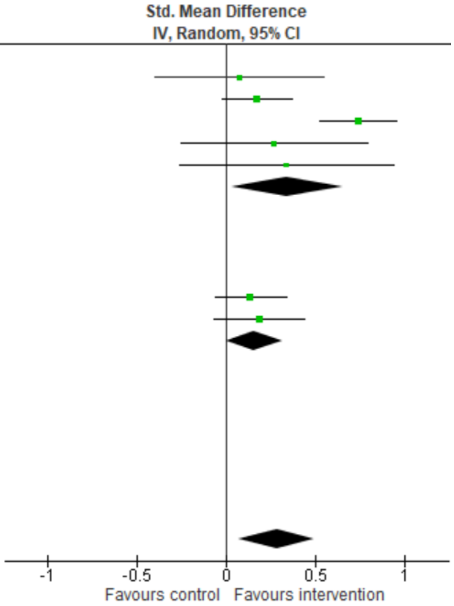

Psychological distress (depression) – maintenance by intervention type

Study or Subgroup	Intervention Mean	Intervention SD	Intervention Total	Control Mean	Control SD	Control Total	Weight	Std. Mean Difference IV, Random, 95% CI
**1.11.1 Self-management**								
Aragones 2019	1.11	0.72	167	1.34	0.76	161	13.3%	-0.31 [-0.53, -0.09]
Barley 2014	9.5	4.6	32	8.8	4.8	37	11.1%	0.15 [-0.33, 0.62]
Ell 2017	1.69	0.06	178	1.63	0.06	170	13.3%	1.00 [0.77, 1.22]
Griva 2019	5.3	4.22	20	4.22	3.88	24	9.9%	0.26 [-0.33, 0.86]
**Subtotal (95% CI)**			**397**			**392**	**47.7%**	**0.28 [-0.48, 1.04]**
Heterogeneity: Tau² = 0.56; Chi² = 68.16, df = 3 (P < 0.00001); I² = 96%								
Test for overall effect: Z = 0.71 (P = 0.47)								
**1.11.2 Collaborative care**								
Ali 2020	0.39	0.27	185	0.53	0.37	193	13.4%	-0.43 [-0.63, -0.23]
Salisbury 2018	6.1	4.6	630	6.8	4.6	625	13.9%	-0.15 [-0.26, -0.04]
**Subtotal (95% CI)**			**815**			**818**	**27.3%**	**-0.28 [-0.55, -0.01]**
Heterogeneity: Tau² = 0.03; Chi² = 5.50, df = 1 (P = 0.02); I² = 82%								
Test for overall effect: Z = 2.01 (P = 0.04)								
**1.11.3 Cognitive and behavioural activiation**								
Boeschoten 2017	20.8	10.4	64	20.1	9.2	67	12.4%	0.07 [-0.27, 0.41]
Naik 2019	10.1	6.9	96	12.6	6.5	70	12.6%	-0.37 [-0.68, -0.06]
**Subtotal (95% CI)**			**160**			**137**	**25.0%**	**-0.16 [-0.59, 0.28]**
Heterogeneity: Tau² = 0.07; Chi² = 3.48, df = 1 (P = 0.06); I² = 71%								
Test for overall effect: Z = 0.71 (P = 0.48)								
**Total (95% CI)**			**1372**			**1347**	**100.0%**	**0.02 [-0.33, 0.36]**
Heterogeneity: Tau² = 0.22; Chi² = 113.01, df = 7 (P < 0.00001); I² = 94%								
Test for overall effect: Z = 0.10 (P = 0.92)								
Test for subgroup differences: Chi² = 1.87, df = 2 (P = 0.39), I² = 0%								

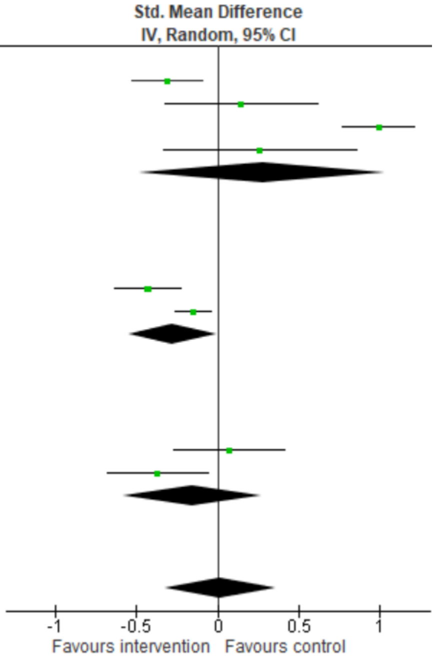

For peer review only

Table 4 significant effects forest plots

Clinical endpoints – post-intervention by intervention length

	Intervention			Control				Std. Mean Difference
**Study or Subgroup**	**Mean**	**SD**	**Total**	**Mean**	**SD**	**Total**	**Weight**	**IV, Random, 95% CI**
**1.3.1 <3months**								
**Subtotal (95% CI)**			**0**			**0**		**Not estimable**
Heterogeneity: Not applicable								
Test for overall effect: Not applicable								
**1.3.2 3-6months**								
House 2018	7.1	1.24	37	7.1	1.79	38	8.1%	0.00 [-0.45, 0.45]
Morgan 2013	6.9	0.26	89	7.4	0.36	67	8.9%	-1.62 [-1.99, -1.26]
Naik 2019	9.1	1.7	119	8.7	1.7	82	9.6%	0.23 [-0.05, 0.52]
Schneider 2016	7.8	0.9	15	7.7	0.8	14	5.7%	0.11 [-0.62, 0.84]
**Subtotal (95% CI)**			**260**			**201**	**32.2%**	**-0.33 [-1.30, 0.65]**
Heterogeneity: $Tau^2 = 0.93$; $Chi^2 = 66.74$, df = 3 ($P < 0.00001$); $I^2 = 96\%$								
Test for overall effect: Z = 0.66 (P = 0.51)								
**1.3.3 >6months**								
Ali 2020	8.19	1.8	192	8.44	1.82	202	10.2%	-0.14 [-0.34, 0.06]
Cummings 2019	8.96	2.1	67	9.04	2.2	72	9.1%	-0.04 [-0.37, 0.30]
Ell 2010	8.52	2.01	193	8.59	2.26	194	10.2%	-0.03 [-0.23, 0.17]
Jackson 2021	137.8	17.4	91	148.6	20.1	91	9.5%	-0.57 [-0.87, -0.28]
Katon 2010	131	18.2	105	132.3	17.4	106	9.7%	-0.07 [-0.34, 0.20]
McDermott 2015	9.8	2.3	84	10.3	2.2	105	9.5%	-0.22 [-0.51, 0.07]
Sajatovic 2017	7.69	1.9	100	7.77	2	100	9.6%	-0.04 [-0.32, 0.24]
**Subtotal (95% CI)**			**832**			**870**	**67.8%**	**-0.15 [-0.28, -0.02]**
Heterogeneity: $Tau^2 = 0.01$; $Chi^2 = 10.68$, df = 6 ($P = 0.10$); $I^2 = 44\%$								
Test for overall effect: Z = 2.25 (P = 0.02)								
**Total (95% CI)**			**1092**			**1071**	**100.0%**	**-0.22 [-0.47, 0.03]**
Heterogeneity: $Tau^2 = 0.15$; $Chi^2 = 80.56$, df = 10 ($P < 0.00001$); $I^2 = 88\%$								
Test for overall effect: Z = 1.72 (P = 0.09)								
Test for subgroup differences: $Chi^2 = 0.13$, df = 1 ($P = 0.72$), $I^2 = 0\%$								

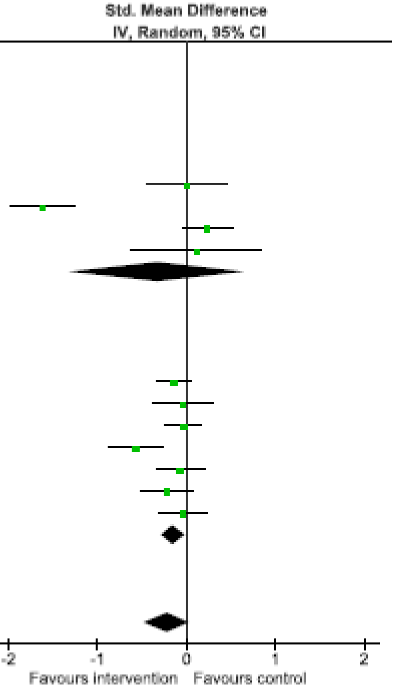

review only

Psychological distress (depression) – post-intervention by intervention length

Study or Subgroup	Intervention Mean	Intervention SD	Intervention Total	Control Mean	Control SD	Control Total	Weight	Std. Mean Difference IV, Random, 95% CI
**1.10.1 <3months**								
Boeschoten 2017	20.3	8.8	74	21	9.1	78	5.0%	-0.08 [-0.40, 0.24]
Bogner 2008	9.9	10.7	32	19.3	15.2	32	4.5%	-0.71 [-1.21, -0.20]
Garvey 2015	6.32	4.19	22	7.82	3.83	22	4.3%	-0.37 [-0.96, 0.23]
Park 2014	5.3	3.4	22	7.6	5.8	21	4.3%	-0.48 [-1.09, 0.13]
Ross 2005	11.8	9.93	15	16.78	7.89	9	3.6%	-0.52 [-1.36, 0.32]
Wilson 2018	17.46	6.16	22	20.5	5.3	25	4.3%	-0.52 [-1.11, 0.06]
**Subtotal (95% CI)**			**187**			**187**	**26.0%**	**-0.36 [-0.57, -0.14]**
Heterogeneity: Tau² = 0.01; Chi² = 5.38, df = 5 (P = 0.37); I² = 7%								
Test for overall effect: Z = 3.21 (P = 0.001)								
**1.10.2 3-6months**								
Aragones 2019	1.29	0.77	167	1.42	0.73	161	5.2%	-0.17 [-0.39, 0.04]
Barley 2014	10.3	3.8	35	9.2	4.6	39	4.7%	0.26 [-0.20, 0.71]
Ell 2017	1.02	0.07	178	1.15	0.07	170	5.1%	-1.85 [-2.10, -1.60]
House 2018	2.4	1.87	30	1.1	1.8	29	4.5%	0.70 [0.17, 1.23]
Markle-Reid 2018	4.67	4.83	71	5.71	5.24	65	5.0%	-0.21 [-0.54, 0.13]
Miklavcic 2020	4.94	4.46	63	5.82	4.93	50	4.9%	-0.19 [-0.56, 0.19]
Morgan 2013	7.1	0.8	164	9	0.9	146	5.1%	-2.23 [-2.52, -1.95]
Naik 2019	10.9	6.1	107	12.4	6	80	5.0%	-0.25 [-0.54, 0.04]
Salisbury 2018	6.1	4.4	654	6.6	4.5	641	5.3%	-0.11 [-0.22, -0.00]
Schneider 2016	12.5	14.8	15	12.3	4.1	14	3.9%	0.02 [-0.71, 0.75]
**Subtotal (95% CI)**			**1484**			**1395**	**48.6%**	**-0.42 [-0.96, 0.12]**
Heterogeneity: Tau² = 0.71; Chi² = 347.55, df = 9 (P < 0.00001); I² = 97%								
Test for overall effect: Z = 1.53 (P = 0.13)								
**1.10.3 >6months**								
Ali 2020	0.52	0.38	192	0.82	0.54	202	5.2%	-0.64 [-0.84, -0.44]
Cummings 2019	6.3	5.9	67	7.9	7	72	5.0%	-0.25 [-0.58, 0.09]
Katon 2010	0.83	0.68	105	1.14	0.66	106	5.1%	-0.46 [-0.73, -0.19]
Kroenke 2009	1.14	0.69	123	1.69	0.74	127	5.1%	-0.77 [-1.02, -0.51]
Sajatovic 2017	15.92	10	100	18.55	8.8	100	5.1%	-0.28 [-0.56, 0.00]
**Subtotal (95% CI)**			**587**			**607**	**25.4%**	**-0.50 [-0.69, -0.30]**
Heterogeneity: Tau² = 0.03; Chi² = 10.51, df = 4 (P = 0.03); I² = 62%								
Test for overall effect: Z = 5.07 (P < 0.00001)								
**Total (95% CI)**			**2258**			**2189**	**100.0%**	**-0.45 [-0.73, -0.16]**
Heterogeneity: Tau² = 0.38; Chi² = 366.51, df = 20 (P < 0.00001); I² = 95%								
Test for overall effect: Z = 3.11 (P = 0.002)								
Test for subgroup differences: Chi² = 0.88, df = 2 (P = 0.64), I² = 0%								

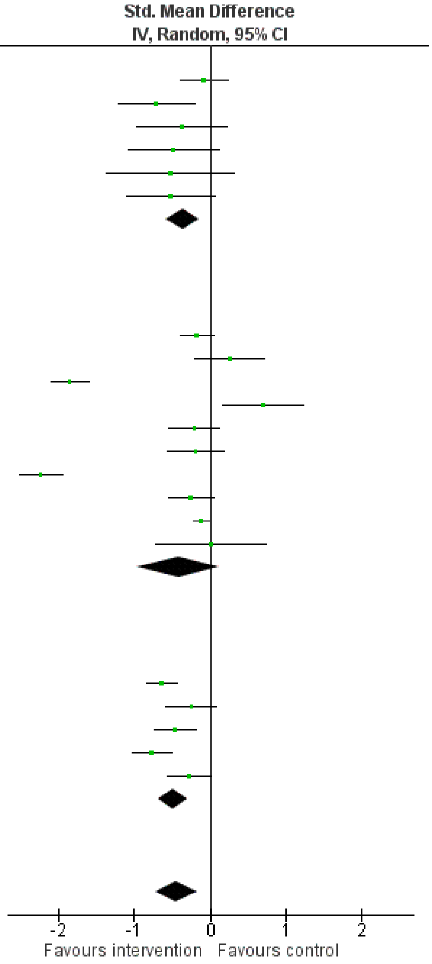

Emotional wellbeing – post-intervention by intervention length

Study or Subgroup	Intervention Mean	Intervention SD	Intervention Total	Control Mean	Control SD	Control Total	Weight	Std. Mean Difference IV, Random, 95% CI
**1.8.1 <3months**								
Druss 2018	34.49	11.15	198	34.25	11.97	202	16.4%	0.02 [-0.18, 0.22]
Goldberg 2013	50.5	11.1	28	43.2	10.7	30	10.7%	0.66 [0.13, 1.19]
**Subtotal (95% CI)**			**226**			**232**	**27.0%**	**0.29 [-0.33, 0.91]**
Heterogeneity: Tau² = 0.16; Chi² = 4.93, df = 1 (P = 0.03); I² = 80%								
Test for overall effect: Z = 0.92 (P = 0.36)								
**1.8.2 3-6months**								
Barley 2014	32.4	11.2	35	35.4	11.8	39	11.9%	-0.26 [-0.72, 0.20]
Ell 2017	44.54	1.03	178	43.67	1.02	170	16.0%	0.85 [0.63, 1.07]
Miklavcic 2020	56.69	8.37	65	53.88	7.67	56	13.6%	0.35 [-0.01, 0.71]
**Subtotal (95% CI)**			**278**			**265**	**41.5%**	**0.34 [-0.28, 0.96]**
Heterogeneity: Tau² = 0.27; Chi² = 20.11, df = 2 (P < 0.0001); I² = 90%								
Test for overall effect: Z = 1.07 (P = 0.28)								
**1.8.3 >6months**								
Ell 2010	47.31	11.48	193	43.6	12.46	194	16.3%	0.31 [0.11, 0.51]
Sajatovic 2017	42.05	11.1	100	39.58	11.4	100	15.1%	0.22 [-0.06, 0.50]
**Subtotal (95% CI)**			**293**			**294**	**31.4%**	**0.28 [0.12, 0.44]**
Heterogeneity: Tau² = 0.00; Chi² = 0.27, df = 1 (P = 0.61); I² = 0%								
Test for overall effect: Z = 3.35 (P = 0.0008)								
**Total (95% CI)**			**797**			**791**	**100.0%**	**0.31 [0.04, 0.58]**
Heterogeneity: Tau² = 0.11; Chi² = 39.32, df = 6 (P < 0.00001); I² = 85%								
Test for overall effect: Z = 2.23 (P = 0.03)								
Test for subgroup differences: Chi² = 0.03, df = 2 (P = 0.98), I² = 0%								

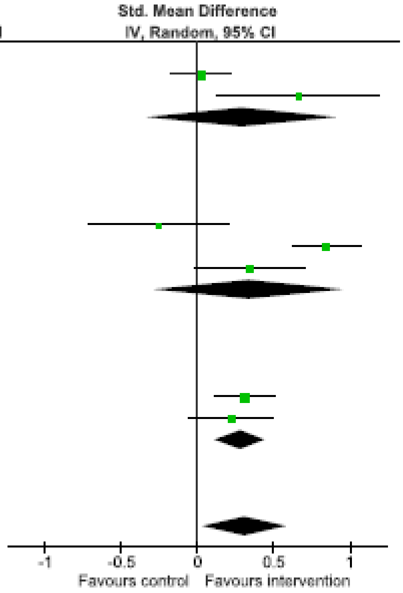

Clinical endpoints – maintenance by intervention length

Study or Subgroup	Intervention Mean	Intervention SD	Intervention Total	Control Mean	Control SD	Control Total	Weight	Std. Mean Difference IV, Random, 95% CI
**1.4.1 <3months**								
Bogner 2008	127.3	17.7	32	141.3	18.8	32	11.1%	-0.76 [-1.27, -0.25]
Griva 2019	8.78	1.54	20	9.5	2.05	24	8.7%	-0.38 [-0.98, 0.21]
**Subtotal (95% CI)**			**52**			**56**	**19.8%**	**-0.60 [-0.99, -0.21]**
Heterogeneity: Tau² = 0.00; Chi² = 0.86, df = 1 (P = 0.35); I² = 0%								
Test for overall effect: Z = 3.04 (P = 0.002)								
**1.4.2 3-6months**								
Naik 2019	8.7	1.6	108	8.9	2	78	21.9%	-0.11 [-0.40, 0.18]
**Subtotal (95% CI)**			**108**			**78**	**21.9%**	**-0.11 [-0.40, 0.18]**
Heterogeneity: Not applicable								
Test for overall effect: Z = 0.75 (P = 0.45)								
**1.4.3 >6months**								
Ali 2020	8.73	1.79	185	8.71	1.69	193	29.0%	0.01 [-0.19, 0.21]
Ell 2010	8.34	2.04	193	8.5	2.17	194	29.2%	-0.08 [-0.28, 0.12]
**Subtotal (95% CI)**			**378**			**387**	**58.3%**	**-0.03 [-0.17, 0.11]**
Heterogeneity: Tau² = 0.00; Chi² = 0.36, df = 1 (P = 0.55); I² = 0%								
Test for overall effect: Z = 0.45 (P = 0.65)								
**Total (95% CI)**			**538**			**521**	**100.0%**	**-0.16 [-0.36, 0.04]**
Heterogeneity: Tau² = 0.02; Chi² = 8.53, df = 4 (P = 0.07); I² = 53%								
Test for overall effect: Z = 1.59 (P = 0.11)								
Test for subgroup differences: Chi² = 7.30, df = 2 (P = 0.03), I² = 72.6%								

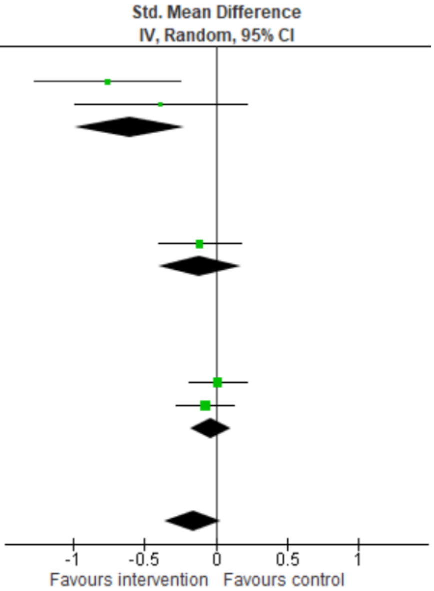

Psychological distress (depression) – maintenance by intervention length

Study or Subgroup	Intervention Mean	Intervention SD	Intervention Total	Control Mean	Control SD	Control Total	Weight	Std. Mean Difference IV, Random, 95% CI
**1.11.1 <3months**								
Boeschoten 2017	20.8	10.4	64	20.1	9.2	67	12.4%	0.07 [-0.27, 0.41]
Griva 2019	5.3	4.22	20	4.22	3.88	24	9.9%	0.26 [-0.33, 0.86]
**Subtotal (95% CI)**			**84**			**91**	**22.3%**	**0.12 [-0.18, 0.42]**
Heterogeneity: Tau² = 0.00; Chi² = 0.30, df = 1 (P = 0.58); I² = 0%								
Test for overall effect: Z = 0.78 (P = 0.43)								
**1.11.2 3-6months**								
Barley 2014	9.5	4.6	32	8.8	4.8	37	11.1%	0.15 [-0.33, 0.62]
Ell 2017	1.69	0.06	178	1.63	0.06	170	13.3%	1.00 [0.77, 1.22]
Naik 2019	10.1	6.9	96	12.6	6.5	70	12.6%	-0.37 [-0.68, -0.06]
**Subtotal (95% CI)**			**306**			**277**	**37.1%**	**0.26 [-0.67, 1.20]**
Heterogeneity: Tau² = 0.65; Chi² = 51.33, df = 2 (P < 0.00001); I² = 96%								
Test for overall effect: Z = 0.55 (P = 0.58)								
**1.11.3 >6months**								
Ali 2020	0.39	0.27	185	0.53	0.37	193	13.4%	-0.43 [-0.63, -0.23]
Aragones 2019	1.11	0.72	167	1.34	0.76	161	13.3%	-0.31 [-0.53, -0.09]
Salisbury 2018	6.1	4.6	630	6.8	4.6	625	13.9%	-0.15 [-0.26, -0.04]
**Subtotal (95% CI)**			**982**			**979**	**40.6%**	**-0.28 [-0.46, -0.10]**
Heterogeneity: Tau² = 0.02; Chi² = 6.11, df = 2 (P = 0.05); I² = 67%								
Test for overall effect: Z = 3.11 (P = 0.002)								
**Total (95% CI)**			**1372**			**1347**	**100.0%**	**0.02 [-0.33, 0.36]**
Heterogeneity: Tau² = 0.22; Chi² = 113.01, df = 7 (P < 0.00001); I² = 94%								
Test for overall effect: Z = 0.10 (P = 0.92)								
Test for subgroup differences: Chi² = 5.95, df = 2 (P = 0.05), I² = 66.4%								

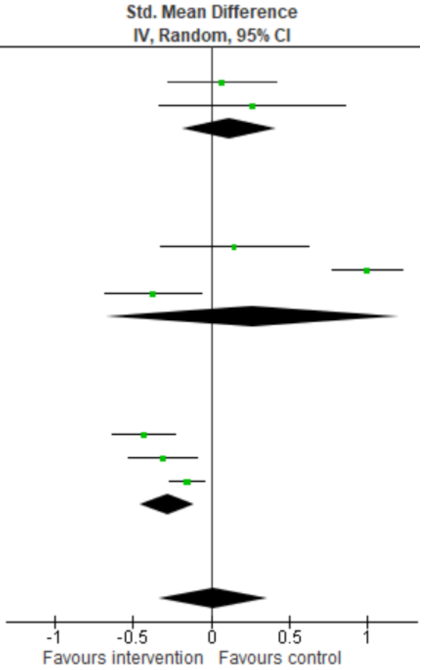

Emotional wellbeing – maintenance by intervention length

Study or Subgroup	Intervention Mean	Intervention SD	Intervention Total	Control Mean	Control SD	Control Total	Weight	Std. Mean Difference IV, Random, 95% CI
**1.9.1 <3months**								
Goldberg 2013	46.9	10.7	28	43.9	11.5	29	9.4%	0.27 [-0.26, 0.79]
Griva 2019	2.38	0.72	20	2.15	0.63	24	7.9%	0.34 [-0.26, 0.93]
**Subtotal (95% CI)**			**48**			**53**	**17.3%**	**0.30 [-0.10, 0.69]**
Heterogeneity: Tau² = 0.00; Chi² = 0.03, df = 1 (P = 0.86); I² = 0%								
Test for overall effect: Z = 1.48 (P = 0.14)								
**1.9.2 3-6months**								
Barley 2014	34.5	11.6	32	33.6	12.5	37	10.5%	0.07 [-0.40, 0.55]
Druss 2018	36.64	12.28	198	34.54	11.82	202	18.7%	0.17 [-0.02, 0.37]
Ell 2010	45.1	12.19	193	43.49	11.66	194	18.6%	0.13 [-0.06, 0.33]
Ell 2017	31.24	0.87	178	30.59	0.89	170	18.0%	0.74 [0.52, 0.95]
Kanwal 2018	41.8	13.8	114	39.4	11.9	128	16.9%	0.19 [-0.07, 0.44]
**Subtotal (95% CI)**			**715**			**731**	**82.7%**	**0.28 [0.02, 0.53]**
Heterogeneity: Tau² = 0.06; Chi² = 21.39, df = 4 (P = 0.0003); I² = 81%								
Test for overall effect: Z = 2.15 (P = 0.03)								
**1.9.3 >6months**								
**Subtotal (95% CI)**			**0**			**0**		**Not estimable**
Heterogeneity: Not applicable								
Test for overall effect: Not applicable								
**Total (95% CI)**			**763**			**784**	**100.0%**	**0.28 [0.07, 0.49]**
Heterogeneity: Tau² = 0.05; Chi² = 21.42, df = 6 (P = 0.002); I² = 72%								
Test for overall effect: Z = 2.63 (P = 0.009)								
Test for subgroup differences: Chi² = 0.01, df = 1 (P = 0.93), I² = 0%								

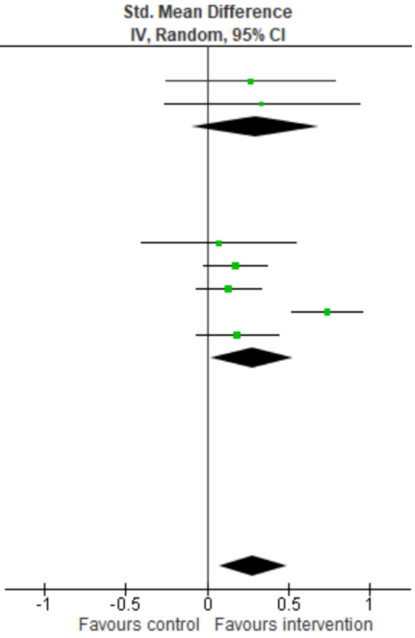

For peer review only - http://bmjopen.bmj.com/site/about/guidelines.xhtml

Supplementary table 4 significant effects forest plots

Psychological distress (depression) – post-intervention MLTCs combination type

Study or Subgroup	Intervention Mean	Intervention SD	Intervention Total	Control Mean	Control SD	Control Total	Weight	Std. Mean Difference IV, Random, 95% CI
**1.10.1 Physical-Physical**								
Garvey 2015	6.32	4.19	22	7.82	3.83	22	4.3%	-0.37 [-0.96, 0.23]
House 2018	2.4	1.87	30	1.1	1.8	29	4.5%	0.70 [0.17, 1.23]
Markle-Reid 2018	4.67	4.83	71	5.71	5.24	65	5.0%	-0.21 [-0.54, 0.13]
Miklavcic 2020	4.94	4.46	63	5.82	4.93	50	4.9%	-0.19 [-0.56, 0.19]
Park 2014	5.3	3.4	22	7.6	5.8	21	4.3%	-0.48 [-1.09, 0.13]
Salisbury 2018	6.1	4.4	654	6.6	4.5	641	5.3%	-0.11 [-0.22, -0.00]
**Subtotal (95% CI)**			**862**			**828**	**28.2%**	**-0.11 [-0.34, 0.12]**
Heterogeneity: $Tau^2 = 0.04$; $Chi^2 = 11.64$, df = 5 (P = 0.04); $I^2 = 57\%$								
Test for overall effect: Z = 0.93 (P = 0.35)								
**1.10.2 Physical-Mental**								
Ali 2020	0.52	0.38	192	0.82	0.54	202	5.2%	-0.64 [-0.84, -0.44]
Aragones 2019	1.29	0.77	167	1.42	0.73	161	5.2%	-0.17 [-0.39, 0.04]
Barley 2014	10.3	3.8	35	9.2	4.6	39	4.7%	0.26 [-0.20, 0.71]
Boeschoten 2017	20.3	8.8	74	21	9.1	78	5.0%	-0.08 [-0.40, 0.24]
Bogner 2008	9.9	10.7	32	19.3	15.2	32	4.5%	-0.71 [-1.21, -0.20]
Cummings 2019	6.3	5.9	67	7.9	7	72	5.0%	-0.25 [-0.58, 0.09]
Ell 2017	1.02	0.07	178	1.15	0.07	170	5.1%	-1.85 [-2.10, -1.60]
Katon 2010	0.83	0.68	105	1.14	0.66	106	5.1%	-0.46 [-0.73, -0.19]
Kroenke 2009	1.14	0.69	123	1.69	0.74	127	5.1%	-0.77 [-1.02, -0.51]
Morgan 2013	7.1	0.8	164	9	0.9	146	5.1%	-2.23 [-2.52, -1.95]
Naik 2019	10.9	6.1	107	12.4	6	80	5.0%	-0.25 [-0.54, 0.04]
Ross 2005	11.8	9.93	15	16.78	7.89	9	3.6%	-0.52 [-1.36, 0.32]
Sajatovic 2017	15.92	10	100	18.55	8.8	100	5.1%	-0.28 [-0.56, 0.00]
Schneider 2016	12.5	14.8	15	12.3	4.1	14	3.9%	0.02 [-0.71, 0.75]
Wilson 2018	17.46	6.16	22	20.5	5.3	25	4.3%	-0.52 [-1.11, 0.06]
**Subtotal (95% CI)**			**1396**			**1361**	**71.8%**	**-0.58 [-0.94, -0.21]**
Heterogeneity: $Tau^2 = 0.47$; $Chi^2 = 278.61$, df = 14 (P < 0.00001); $I^2 = 95\%$								
Test for overall effect: Z = 3.10 (P = 0.002)								
**1.10.3 Mental-Mental**								
**Subtotal (95% CI)**			**0**			**0**		**Not estimable**
Heterogeneity: Not applicable								
Test for overall effect: Not applicable								
**Total (95% CI)**			**2258**			**2189**	**100.0%**	**-0.45 [-0.73, -0.16]**
Heterogeneity: $Tau^2 = 0.38$; $Chi^2 = 366.51$, df = 20 (P < 0.00001); $I^2 = 95\%$								
Test for overall effect: Z = 3.11 (P = 0.002)								
Test for subgroup differences: $Chi^2 = 4.57$, df = 1 (P = 0.03), $I^2 = 78.1\%$								

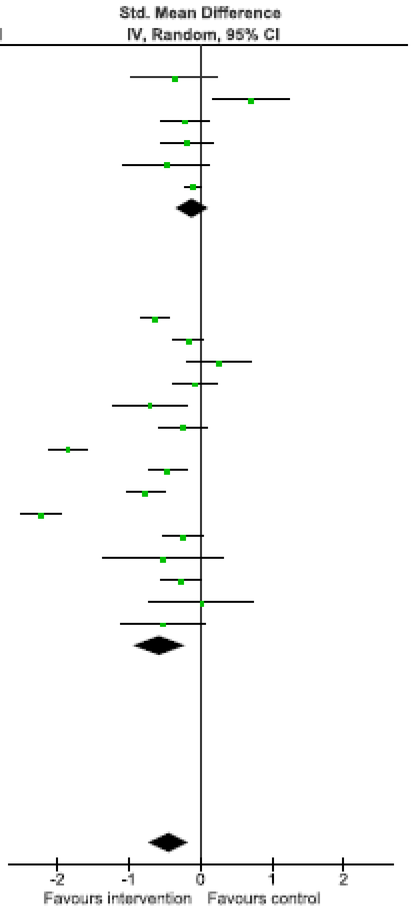

For peer review only - http://bmjopen.bmj.com/site/about/guidelines.xhtml

BMJ Open

The effectiveness of complex behaviour change interventions tested in randomised controlled trials for people with multiple long-term conditions (M-LTCs): systematic review with meta-analysis

Journal:	*BMJ Open*
Manuscript ID	bmjopen-2023-081104.R2
Article Type:	Original research
Date Submitted by the Author:	13-May-2024
Complete List of Authors:	Rookes, Tasmin; University College London Research Department of Primary Care and Population Health<br>Nimmons, Danielle; University College London Research Department of Primary Care and Population Health<br>Frost, Rachael; University College London Research Department of Primary Care and Population Health, Research Department of Primary Care and Population Health<br>Armstrong, Megan; Queen Mary University of London Wolfson Institute of Population Health<br>Davies, Laura; University College London Research Department of Primary Care and Population Health<br>Ross, Jamie; Queen Mary University of London Wolfson Institute of Population Health<br>Hopkins, Jane; Public Contributor<br>Mistry, Manoj; Public Contributor<br>Taylor, Stephanie; Queen Mary University of London, Wolfson Institute of Population Health<br>Walters, Kate; University College London, Primary Care and Population Health
**Primary Subject Heading**:	Public health
Secondary Subject Heading:	General practice / Family practice, Research methods, Patient-centred medicine
Keywords:	Meta-Analysis, Systematic Review, Aging, Patient Reported Outcome Measures, Primary Care < Primary Health Care, PUBLIC HEALTH

SCHOLARONE™
Manuscripts

I, the Submitting Author has the right to grant and does grant on behalf of all authors of the Work (as defined in the below author licence), an exclusive licence and/or a non-exclusive licence for contributions from authors who are: i) UK Crown employees; ii) where BMJ has agreed a CC-BY licence shall apply, and/or iii) in accordance with the terms applicable for US Federal Government officers or employees acting as part of their official duties; on a worldwide, perpetual, irrevocable, royalty-free basis to BMJ Publishing Group Ltd ("BMJ") its licensees and where the relevant Journal is co-owned by BMJ to the co-owners of the Journal, to publish the Work in this journal and any other BMJ products and to exploit all rights, as set out in our licence.

The Submitting Author accepts and understands that any supply made under these terms is made by BMJ to the Submitting Author unless you are acting as an employee on behalf of your employer or a postgraduate student of an affiliated institution which is paying any applicable article publishing charge ("APC") for Open Access articles. Where the Submitting Author wishes to make the Work available on an Open Access basis (and intends to pay the relevant APC), the terms of reuse of such Open Access shall be governed by a Creative Commons licence – details of these licences and which Creative Commons licence will apply to this Work are set out in our licence referred to above.

Other than as permitted in any relevant BMJ Author's Self Archiving Policies, I confirm this Work has not been accepted for publication elsewhere, is not being considered for publication elsewhere and does not duplicate material already published. I confirm all authors consent to publication of this Work and authorise the granting of this licence.

For peer review only

The effectiveness of complex behaviour change interventions tested in randomised controlled trials for people with multiple long-term conditions (M-LTCs): systematic review with meta-analysis

Tasmin Rookes[1], Danielle Nimmons[1], Rachael Frost[2], Megan Armstrong[3], Laura Davies[1], Jamie Ross[3], Jane Hopkins[4], Manoj Mistry[4], Stephanie JC Taylor[3], and Kate Walters[1]

Corresponding Author:
Ms Tasmin Rookes (MSc), Centre for Ageing Population Studies, Research Department of Primary Care and Population Health, University College London, London, UK; t.rookes@ucl.ac.uk; 02076799727

[1]Research Department of Primary Care and Population Health, University College London, London, UK
[2]School of Public and Allied Health, Liverpool John Moores University, Liverpool, UK
[3]Wolfson Institute of Population Health, Queen Mary University of London, London, UK
[4]Patient and Public Involvement Contributor, London, UK

Key Words
Multiple long-term conditions; randomised controlled trial; meta-analysis; systematic review; behaviour change.

Word Count 4166

For peer review only

Abstract:

Introduction

Prevalence of multiple long-term conditions (M-LTCs) increases as adults age and impacts quality of life and health outcomes. To help people manage these conditions, complex behaviour change interventions are utilised, often based on research conducted in those with single LTCs. However, the needs of those with M-LTCs can differ due to complex health decision making and engagement with multiple health and care teams.

Objectives

The aim of this review is to identify whether current interventions are effective for people living with M-LTCs, and which outcomes are most appropriate to detect this change.

Methods

Five databases (MEDLINE, Embase, PsychInfo, CINAHL and Web of Science) were systematically searched, between January 1999 to January 2022, to identify RCTs evaluating effectiveness of behaviour change interventions in people with M-LTCs. Intervention characteristics, intervention effectiveness, and outcome measures were meta-analysed and narratively synthesised.

Results

Fifty-three eligible articles were included. Emotional wellbeing and psychological distress (depression and anxiety) outcomes were most amenable to change (emotional wellbeing: SMD 0.31(95% 0.04 to 0.58); depression psychological distress: SMD -0.45(95% -0.73 to -0.16); anxiety psychological distress: SMD -0.14(95% -0.28 to 0.00)), particularly for interventions with a collaborative care approach. Interventions targeting those with a physical and mental health condition and those with cognitive and/or behavioural activation approach saw larger reductions in psychological distress outcomes. Interventions that lasted for longer than 6-months significantly improved the widest variety of outcomes.

Conclusion

Complex interventions can be successfully delivered to those with M-LTCs. These are most effective at reducing psychological distress in those with physical and mental LTCs. Further research is needed to identify the effective components of interventions for people with two or more physical LTCs and which outcome is most appropriate for detecting this change.

Strengths and Limitations:

- Broad search terms across many key databases have enabled a summary of a large amount of evidence exploring the effectiveness of complex behaviour change interventions in people living with M-LTCs in a systematic review and meta-analysis.
- These findings impact on the development and implementation of such interventions in people with M-LTCs, which is a growing area of research and clinical practice.
- Double screening of eligible papers was only completed for a percentage of the identified studies, meaning some eligible papers could have been missed.
- Complex interventions are difficult to define and determining eligibility can be a challenge, so a conservative approach to exclusion was used.
- Some meta-analyses results had high heterogeneity, suggesting that they may not have been comparable.

Introduction:

For peer review only

Living with multiple long-term conditions (M-LTCs) is defined as an individual having two or more health conditions at the same time. Globally, the presence of M-LTCs is reported to be 27.2% in all adults, rising to 67.0% in those aged 74 and over (1). With an ageing population, this is predicted to rise in those over 65 in the UK, from 54% in 2015, to 68% in 2035 (2). In comparison with the general population, those with M-LTCs are found to have lower quality of life and higher mortality risk (3, 4), as well as greater healthcare costs and utilisation (5). As prevalence increases, nationally and globally, the pressure and reliance on healthcare services, people living with the conditions, and their carers also increases, making the challenge of managing M-LTCs a priority for current research agendas and healthcare systems, such as the NHS and the James Lind Alliance (1, 6, 7).

Despite this increased focus on managing M-LTCs, many of the beneficial complex interventions designed to help people with M-LTCs are based on findings from research and interventions aimed at people with single health conditions and applied more widely to those with M-LTCs. Complex interventions contain multiple interacting components and often depend on the behaviours of those delivering and receiving the intervention and need to be adapted to the specific participant group and setting (8). The chronic disease self-management program (CDSMP) has found mixed effectiveness in people with M-LTCs, depending on the combination and type of health conditions participants experience, being more effective for people with physical health conditions and probable depression (9, 10). In a recent systematic review and meta-analysis of 14 studies testing behavioural interventions in people with M-LTCs, there was little to no effect on physical activity and weight loss (11). Managing M-LTCs presents a different challenge to those with single LTCs due to the often-fragmented care across multiple primary and secondary care teams and the varied and competing needs of the individual (12, 13). These needs include complex priority setting and decision-making to adhere to multiple healthcare regimes and weighing up conflicting advice from different specialties (14). This is reflected in poorer outcomes when receiving interventions to help with the management of their M-LTCs, suggesting that the specific needs of these people are not being addressed by current interventions (15).

Systematic reviews, highlight that the effectiveness of complex interventions varies depending on the combination of LTCs, the type of intervention being used, and the outcome measure being used to assess effectiveness (9, 15). Determining the most appropriate outcome measure is particularly difficult in this population, given the wide variety of outcomes that may be relevant depending on the conditions and challenges faced by the population e.g., clinical outcomes, wellbeing, quality of life, and distress. Therefore, it is essential we first identify whether complex interventions currently being tested in people with M-LTCs are effective for this population and then explore which outcome measure is most sensitive to detecting a significant positive change in outcomes.

Aims:

1. To evaluate the evidence around the effectiveness of behaviour change interventions in people with M-LTCs.
2. To identify the outcome measures most sensitive to change, to identify effectiveness of complex behaviour change interventions in people with M-LTCs.

Methods:

This review is reported in line with the PRISMA guidelines (16) (supplementary table 1) and registered on PROSPERO (ID: CRD42021287847).

Search

The databases MEDLINE, Embase, PsychInfo, CINAHL and Web of Science were systematically searched from January 1999 to January 2022, and an example search term list can be seen in supplementary figure 1. This cutoff date was chosen, to align with the

For peer review only

oldest dated paper identified in a previous Cochrane systematic review for complex interventions in people with M-LTCs (13) and to ensure that the findings were applicable to current healthcare settings. The identified articles were exported into Endnote and duplicates removed. 10% of identified titles and abstracts were independently screened by two reviewers (T.R. and D.N.) against the inclusion criteria, using Rayyan (17). As there was a less than 5% conflict (n=41/938; 4.37%), single screening was conducted by T.R. for the remaining titles and abstracts. For full text screening, 20% were independently reviewed by T.R. and D.N., and again, there was only a 5% discrepancy (n=2/40; 5%), and so the remainder were independently screened by T.R. for eligibility.

Inclusion Criteria
Participants: People with two or more physical and/or mental health conditions.
Intervention: A complex behaviour change intervention to help with the management of health conditions aiming to improve individual health outcomes, which could be targeted at the individual or organisational level.
Comparator: Treatment as usual.
Outcomes: Any individual health outcomes, such as quality of life, emotional wellbeing, clinical outcomes, psychological distress, behaviour change, and pain.
Setting: Interventions delivered in primary and community care settings.
Timeframe: published between 1st January 1999 and 17th January 2022.
Design: Randomised Controlled Trials

Data extraction and Outcome measures
Study characteristics were extracted by L.D. and checked by T.R. for completeness. Outcome measures were extracted by T.R. and checked by D.N. for completeness. For all studies, we extracted trial authors, year of publication, country, intervention description and length, study follow-up length, M-LTCs targeted, sample size at each timepoint, means and standard deviations for relevant outcome measures. Outcome measures were grouped into six categories, 1) quality of life, 2) clinical endpoints, 3) behaviour change, 4) pain, 5) wellbeing, and 6) psychological distress. Where data were not reported in the paper, we contacted the authors for further details (11 approached, 1 responded providing further data). For the 10 who did not respond, and for outcomes which could not be synthesised using meta-analysis, the available data were summarised using narrative synthesis.

Study quality assessment
Trial result quality was assessed using the Risk of Bias 2 (RoB 2) tool (18). Initial assessment for 20% of eligible studies (n=12) was completed independently by two reviewers (T.R. and D.N.), with agreement for 8/12 trials (75%). Therefore, a further 10% (n=6) were independently reviewed, with 100% agreement between the two reviewers. The remaining 38 trials findings were assessed for risk of bias by T.R. Risk of bias was used to inform narrative synthesis but not to exclude studies.

Patient and Public Involvement
Two public co-applicants living with M-LTCs were involved throughout the whole project. This included the review process, including refining the aims and inclusion criteria, selecting the most important outcomes to focus on, analysing and providing feedback on findings, and in academic and lay dissemination of findings. They have been included as co-authors. A public engagement workshop, involving the two public co-applicants, and an additional six people with M-LTCs, was held to discuss relevant outcomes to focus on, effective ways to disseminate research findings with the public, and to develop future research questions.

Data analysis

For peer review only

Where there were 3 or more studies reporting the same outcome, we conducted a meta-analysis. RevMan 5.4 (19) was used to calculate standardised mean differences and 95% confidence intervals. Standardised mean differences were used due to varying outcome measures being used to measure the same outcomes. To decide which outcome measures to pool together under outcome categories, three reviewers (T.R., D.N., and R.F.) met and discussed the appropriateness of each measure in the analyses. If there was more than one measures for the same outcome our decision on which to include was based on if one was the primary outcome, the measure most closely related to the outcome, and the extent of missing data. Psychological distress was separated into anxiety and depression, as many studies reported both outcomes and we felt the two constructs differed.

Inverse-variance random effects meta-analysis models was chosen, a priori, as the types of interventions and populations studied were expected to be somewhat heterogeneous due to the diverse populations living with M-LTCs and the different approaches to intervention and settings. To explore this expected variation, preplanned sub-group analyses were conducted, including by types of interventions, length of intervention. Post hoc analysis of combination of LTCs (physical-physical; physical-mental; and mental-mental) were explored following discussions with public contributors. Interventions were grouped into three intervention types through discussions between three reviewers (T.R. D.N. R.F.), which are well established in the literature, self-management, collaborative care, and cognitive and/or behavioural activation, based on their predominant features (table 1). Exploratory supplementary subgroup analyses were carried out to explore 1) Intervention type: self-management, collaborative care, and cognitive and/or behavioural activation), 2) Length of intervention: <3-months, 3-6-months, and 6+months, for outcomes immediately post-intervention delivery, 3) Maintenance of effect: Time since intervention was complete to final follow-up, grouped as <3 months, 3+months post intervention, and 4) Type of M-LTCs combinations: physical-physical, mental-physical and mental-mental.

Heterogeneity was assessed across studies using I^2 statistics, with proportions greater than 25%, 50%, and 75% considered to have low, moderate, and high heterogeneity, respectively (20).

Table 1. Example interventions and their characteristics for allocation into the three types of intervention for supplementary subgroup analysis.

Intervention Type	Example of intervention and characteristics
Self-management	Psychoeducational interventions, with goal setting and problem solving, to help people manage the symptoms and improve their health outcomes related to their health conditions. Also included telemonitoring approaches, where participants received information and had their symptoms monitored over time.
Collaborative care	Interventions with behaviour change components which target the participants, the clinical team, and the system they are embedded within. Also included stepped care, where participants started with one treatment pathway and depending on progress, could be stepped up to more intensive treatment.
Cognitive and/or behavioural activation	Often use motivational interviewing, cognitive behavioural therapy, behavioural activation, and/or problem solving to empower people to help people understand the association between health conditions and cognitive processes and create healthier behaviours for future problems.

Results

For peer review only

After searching and de-duplication, 9,354 titles and abstracts were screened. 200 full text articles were screened and 53 included in the synthesis (see figure 1 and supplementary figure 2).

Study characteristics

Across 53 RCTs there were a total of 14,740 participants with study sizes ranging from 25 to 3324. The studies were published between 2001 and 2021, with a steady increase in publications from 2009 to the present day, with 58% published since 2017. Studies were conducted in the United States (n=22), Canada (n=7), Australia (n=5), UK (n=5), Netherlands (n=3), Spain (n=2), Ireland (n=2), and the remaining eight in Singapore, China, Nigeria, Germany, Indonesia, South Korea, Croatia, and India (one study was conducted across two countries). Interventions were aimed at people with a combination of physical and mental health conditions (n=27), two or more physical health conditions (n=25), and one study was for people with a combination of two mental health conditions. The health conditions included diabetes, depression, schizophrenia, coronary heart disease, chronic pain, HIV, and hypertension, as well as interventions which were open to people with multiple different health conditions.

Most interventions were self-management interventions (n=28), with 15 collaborative care interventions and 10 cognitive and/or behavioural activation interventions. Interventions were heterogeneous in terms of the person delivering them; they were delivered by individual or combinations of nurses, doctors, psychologists, other health-care professionals, and trained peer support leaders. The mode of delivery varied from face-to-face, in clinics or homes, telephone, and through digital platforms. The length of these interventions ranged from 3 weeks to 2 years, with most lasting between 3-12 months. Post-intervention, maintenance follow-up lengths varied from 6-weeks to 12-months. A summary of the studies and the interventions can be found in supplementary table 2.

Risk of Bias

Judgement of the overall risk of bias of the trial findings was variable, 29 results were rated as low risk, 20 as having some concerns and four as having a high risk of bias. All the studies measuring quality of life and pain, half of the studies measuring clinical endpoints, 80% measuring functioning and 40% of those measuring psychological distress had at least some concerns with risk of bias (see supplementary table 2).

Outcome characteristics

For the primary outcome, 10 measured a clinical endpoint relevant to the included LTCs (e.g., HbA1c, blood pressure, cholesterol), 10 studies used a psychological distress measure, five used a behaviour change measure (e.g., alcohol use (AUDIT), five a functioning measure (e.g., physical functioning), four studies measured quality of life, one study measured pain, no studies used emotional wellbeing as the primary outcome, and six studies used a composite of these measures (often psychological distress combined with a clinical endpoint) and 12 studies used an outcome measure that did not fit into these categories e.g., acceptability of intervention.

For the secondary outcomes included in the meta-analyses, 11 studies measured functioning and psychological distress as an outcome, nine measured emotional wellbeing, seven measured quality of life, six behaviour change and four clinical endpoint outcomes.

Main analysis: Meta-analysis of individual outcomes

Outcome data (means and SD for each group) assessed immediately post-intervention was available from 31/53 studies for meta-analysis across the seven outcome types. Effectiveness of interventions was detected when emotional wellbeing (SMD 0.31 [0.04, 0.58]) and psychological distress, both depression (SMD -0.45 [-0.73, -0.16]) and anxiety (SMD -0.14 [-0.28, -0.00]), were measured. The effect sizes for psychological distress

For peer review only

(depression) and emotional wellbeing (both post-intervention and maintenance) were moderate, but heterogeneity was high. For psychological distress (anxiety) the effect size was small and heterogeneity moderate. No other outcomes detected a significant effect from the interventions (see table 2; and supplementary figure 3 for forest plots).

Outcome data assessed at follow-up, to measure the effect of maintenance, was available for meta-analysis from 15 studies in total across outcomes. Effectiveness of interventions was only detected when emotional wellbeing was measured (SMD 0.28 [0.07, 0.49]), with moderate heterogeneity. All other outcomes did not detect a significant effect (see table 2; and supplementary figure 3 for forest plots).

Table 2. Summary of pooled effects of outcome measures, for each outcome, assessed immediately post-intervention and at a follow-up/maintenance timepoint.

	**Overall effect**					
	**Post-intervention**			**Maintenance**		
**Outcome**	*n*	SMD [95% CI]	I^2	*n*	SMD [95% CI]	I^2
Quality of life	8	0.13 [-0.04, 0.30]	59%	3	0.61 [-1.54, 2.75]	99%
Clinical endpoints	11	-0.22 [-0.47, 0.03]	88%	5	-0.16 [-0.36, 0.04]	53%
Behaviour change	6	0.06 [-0.18, 0.29]	78%	3	0.02 [-0.10, 0.15]	23%
Pain	4	-0.32 [-0.70, 0.07]	77%	0	N/A	N/A
Functioning	14	0.05 [-0.06, 0.15]	42%	8	0.05 [-0.05, 0.16]	16%
Psychological distress (depression)	21	**-0.45 [-0.73, -0.16]**	95%	8	0.02 [-0.33, 0.36]	94%
Psychological distress (anxiety)	9	**-0.14 [-0.28, 0.00]**	40%	4	-0.08 [-0.18, 0.02]	0%
Emotional wellbeing	7	**0.31 [0.04, 0.58]**	85%	7	**0.28 [0.07, 0.49]**	72%

n is number of studies measuring the outcome. Positive results for psychological distress, indicated by a decrease in scores and for emotional wellbeing by an increase in scores.

Narrative synthesis:
For the 22 studies, where meta-analysis was not possible due to data not being available in the required format, 15 reported at least one of the seven outcomes, and found results mostly consistent with the meta-analysis. Seven studies found positive intervention effects, which measured psychological distress (n=3), clinical endpoints (n=2), behaviour change (n=1), and functioning (n=1) (see supplementary table 3). Studies measuring quality of life (n=2) found no effects.

Subgroup Analyses:
We conducted three supplementary subgroup analyses to explore the heterogeneity within the results by intervention type, duration of intervention and MLTC combination type:

1) *Intervention type*

For interventions with a self-management approach, none of the outcomes detected a significant benefit immediately post intervention and only emotional wellbeing at follow-up significantly improved (SMD 0.34 [0.03, 0.65]), but high heterogeneity remained for most outcomes (see table 3; and supplementary figure 3 for forest plots).

For interventions with a collaborative care approach, significant differences post-intervention were detected for quality of life (SMD 0.15 [0.02, 0.27]), with low heterogeneity and psychological distress (depression; SMD -0.82 [-1.40, -0.24]), with high heterogeneity,

For peer review only

(table 3). This high heterogeneity may be due to the different outcome measures used to measure depression across the studies, whereas quality of life measures were similar. When measuring maintenance effects, only one outcome was measured by enough studies to conduct meta-analyses and no effects were found.

For interventions with a cognitive and/or behavioural activation approach, post-intervention effects were seen for psychological distress (depression) only (SMD -0.24 [-0.38, -0.09]), with no heterogeneity.

Table 3. Pooled effects of outcomes from meta-analysis, stratified by the three groups of complex behaviour change intervention types described in Table 1.

	Self-management			Collaborative care			Cognitive and/or behavioural activation		
**Post-intervention**	*n*	SMD [95% CI]	I^2	*n*	SMD [95% CI]	I^2	*n*	SMD [95% CI]	I^2
Quality of life	2	N/A	N/A	4	**0.15 [0.02, 0.27]**	31%	2	N/A	N/A
Clinical endpoints	3	-0.29 [-0.61, 0.02]	61%	4	-0.45 [-1.00, 0.11]	95%	4	0.06 [-0.10, 0.23]	0%
Behaviour change	3	0.01 [-0.60, 0.61]	91%	1	N/A	N/A	2	N/A	N/A
Functioning	9	0.05 [-0.06, 0.16]	26%	3	0.08 [-0.20, 0.35]	74%	2	N/A	N/A
Psychological distress (depression)	8	-0.30 [-0.91, 0.31]	96%	6	**-0.82 [-1.40, -0.24]**	97%	7	**-0.24 [-0.38, -0.09]**	0%
Psychological distress (anxiety)	5	-0.05 [-0.23, 0.14]	0%	2	N/A	N/A	2	N/A	N/A
Emotional wellbeing	5	0.33 [-0.10, 0.76]	90%	1	N/A	N/A	1	N/A	N/A
**Maintenance**									
Clinical endpoints	1	N/A	N/A	3	-0.18 [-0.48, 0.12]	74%	1	N/A	N/A
Functioning	5	0.10 [-0.02, 0.22]	0%	2	N/A	N/A	1	N/A	N/A
Psychological distress (depression)	4	0.28 [-0.48, 1.04]	96%	2	N/A	N/A	2	N/A	N/A
Emotional wellbeing	5	**0.34 [0.03, 0.65]**	76%	2	N/A	N/A	0	N/A	N/A

n is number of studies measuring the outcome. *p* value is considered significant if ≤0.05.

In the 22 studies synthesised narratively, two of the nine self-management interventions (22%), and four of the six collaborative care interventions (67%) found significant effects on outcomes, three of which were psychological distress (depression) measures. These findings generally support the meta-analysis conclusions (see supplementary table 3).

2) Duration of intervention

For interventions lasting for less than 3-months, positive effects post-intervention were only detected with psychological distress (depression) measures (SMD -0.36 [-0.57, -

For peer review only

0.14]), with very low heterogeneity. Maintenance effects could not be explored, as not enough studies measured any of the outcomes.

Significant post-intervention effects for interventions lasting between 3- and 6-months were not found for any of the outcomes. However, significant differences at maintenance were detected when emotional wellbeing was measured (SMD 0.28 [0.02, 0.53]), but heterogeneity was high.

For interventions lasting for longer than 6-months, effects were seen post-intervention for clinical endpoints (SMD -0.15 [-0.28, -0.02]), with low heterogeneity and psychological distress (depression; -SMD 0.50 [-0.69, -0.30]) measures, with moderate heterogeneity, (see table 4). Maintenance effects in interventions lasting longer than 6-months were only identified with psychological distress (depression) measures (SMD -0.28 [-0.46, -0.10]), but heterogeneity was high (table 4; and supplementary figure 3 for forest plots).

Table 4. Pooled effects of outcomes, stratified by intervention length.

	<3-months			3-6months			>6months		
**Post-intervention**	*n*	SMD [95% CI]	I^2	*n*	SMD [95% CI]	I^2	*n*	SMD [95% CI]	I^2
Quality of life	3	0.38 [-0.38, 1.13]	81%	1	N/A	N/A	4	0.11 [-0.13, 0.35]	55%
Clinical endpoints	0	N/A	N/A	4	-0.33 [-1.30, 0.65]	96%	7	**-0.15 [-0.28, -0.02]**	44%
Behaviour change	2	N/A	N/A	2	N/A	N/A	2	N/A	N/A
Functioning	1	N/A	N/A	8	0.07 [-0.02, 0.16]	0%	5	0.07 [-0.15, 0.30]	65%
Psychological distress (depression)	6	**-0.36 [-0.57, -0.14]**	7%	10	-0.42 [-0.96, 0.12]	97%	**5**	**-0.50 [-0.69, -0.30]**	62%
Psychological distress (anxiety)	3	-0.20 [-0.65, 0.26]	50%	4	-0.09 [-0.19, 0.01]	0%	2	N/A	N/A
Emotional wellbeing	2	N/A	N/A	3	0.34 [-0.28, 0.96]	90%	2	N/A	N/A
**Maintenance**									
Functioning	1	N/A	N/A	6	0.03 [-0.10, 0.17]	31%	1	N/A	N/A
Psychological distress (depression)	2	N/A	N/A	3	0.26 [-0.67, 1.20]	96%	3	**-0.28 [-0.46, -0.10]**	94%
Emotional wellbeing	2	N/A	N/A	5	**0.28 [0.02, 0.53]**	81%	0	N/A	N/A

n is number of studies measuring the outcome. *p* value is considered significant if ≤0.05.

Exploring the 22 studies without data for meta-analysis, none of the 3 studies lasting less than 3-months, two of the five studies lasting between 3- and 6-months (40%), and four out of the seven studies lasting longer than 6 months (57%) had significant primary outcome effects (see supplementary table 3).

3) *M-LTCs combination type*

Analysis around effectiveness of interventions depending on the combination of M-LTCs types, grouped as physical-physical, physical-mental, and mental-mental was explored post-hoc. The only significant effect found was post-intervention for psychological distress (depression) measures in people with combined physical and mental health conditions (SMD -0.58 [-0.94, -0.21]), but heterogeneity was high (see supplementary table 4; and supplementary figure 3 for forest plots).

In the narrative synthesis, of those measuring a relevant outcome, three out of nine studies measuring interventions targeted at people with two physical health conditions found positive effects (33%), two of which were measuring clinical endpoints. Of those measuring effects in people with a physical and mental health condition, three out of six studies found positive effects (50%), and these were all measuring psychological distress (depression) (see supplementary table 3).

Discussion

Across the 53 included studies, types of complex interventions, LTCs targeted, and outcome measures used varied widely. Overall, there is evidence that complex interventions can improve the outcomes of people with M-LTCs, and the outcome measures most sensitive to change were emotional wellbeing and psychological distress.

In subgroup analyses, interventions that lasted for longer periods of time, and had a collaborative care approach were associated with greater beneficial outcomes than shorter interventions with a self-management focus. Interventions with a cognitive and/or behavioural activation approach and interventions targeted at people with a combination of a mental and physical health condition had better outcomes when measuring psychological distress (depression) measures.

Results in context

Developing interventions for people with single LTCs centred around psychological variables is not a new concept, with psychological distress and emotional wellbeing factors being the most amenable to change (21). This is particularly important when exploring the psychological factors, such as psychological capability, which enable a person with a health condition to believe they can engage in the necessary behaviour to improve their outcomes (22). In the absence of psychological capability, an intervention targeting this, through goal setting, problem solving, and self-efficacy, could improve wellbeing outcomes (23). These concepts for people with single LTCs appear to transfer over to those with M-LTCs, with psychological distress measures showing a significant improvement in those with a combination of physical and mental health conditions. This is perhaps unsurprising, given the likely higher psychological burden placed on those living with M-LTCs.

In people with single mental LTCs, cognitive and/or behavioural activation centred interventions have a strong evidence base from systematic reviews for reducing peoples' psychological distress and depression symptoms (24, 25). The findings from this review suggest these interventions have similar improvements for psychological distress in people with M-LTCs. The outcomes for this intervention type showed low heterogeneity, which may be due to greater standardised of psychological therapy approaches, which are often manualised with regular supervision. Despite this, often quality of life is chosen as the primary outcome to test the effectiveness of complex interventions, when single clinical outcomes cannot be used. However, here we have shown that emotional wellbeing or psychological distress may be the outcomes more able to detect a change in people with M-LTCs. We have further explored the intervention components and how they link to intervention effectiveness, which is currently under review for publication.

Looking across the outcomes, the most effective intervention type for people with M-LTCs appears to be collaborative care. Collaborative care involves integrating health and care

For peer review only

services across specialties and services to improve the outcomes of the people they are caring for (26). Other reviews have found positive effects of collaborative care interventions for people with a combination of at least one physical LTC and depression (26, 27). The mechanisms that underpin this change are thought to be two-fold, at the individual level, with the person being able to manage their physical LTC better once the depressive symptoms have been reduced, and at the institutional level, with improved delivery of care with a patient centred focus (26). However, heterogeneity was still substantial for this outcome, possibly as collaborative care interventions can involve differing configurations of healthcare professionals and contact frequencies which may impact effectiveness. Further work needs to explore the most effective components of collaborative care interventions when greater numbers of trials are available.

Alongside the type of intervention, the length of the intervention also seems to be an important factor for effectiveness, with interventions lasting for longer than 6-months improving outcomes post-intervention. In line with guidance, when designing an intervention, the minimum clinically effective dose should be identified and empirically tested (8, 28). There is no evidence suggesting that length and effectiveness of interventions are linked in people with single LTCs. However, it may be that the complex and interacting needs of people with M-LTCs require longer interventions to ensure that the skills to self-manage and the systems to support the individual are in place (12). Also, it is worth noting that even with interventions lasting for longer than 6-months, maintenance effects are only seen for psychological distress measures, highlighting again that these outcomes are the most amenable to change (21) and that if we want other outcomes to continue to be improved, then support beyond the intervention period is probably needed. The variable heterogeneity in the intervention length analysis may be partly due to differing intervention types; however, we lacked sufficient studies to explore the effect of length within the different intervention types.

Alongside these findings, it is worth highlighting the lack of studies exploring some of the outcomes of interest, including pain, clinical endpoints, and behaviour change. Therefore, the lack of significant effects for these may be due to the lack of studies measuring them as an outcome. This is even more apparent when looking at the maintenance data.

Future Research

The findings presented here highlight the intervention type and length that are associated with improved outcomes in people with M-LTCs. Therefore, future behaviour change interventions should explore a collaborative care approach and last for longer than 6-months. In addition to this, whilst post-intervention outcomes are effective in those lasting more than 6-months, findings around maintenance effects are less conclusive. This suggests more research is needed to determine how to ensure effective interventions have long lasting effects and improvements can be maintained over time once intervention periods have ended. As collaborative care and self-management intervention types had high heterogeneity, future work should explore the most effective components within these, perhaps utilising methods such as component network meta-analysis.

In addition, whilst the findings here suggest that interventions to improve psychological distress and emotional wellbeing outcomes in those with mental LTCs have been successfully applied to those with mental and physical LTCs, the results for those with two or more physical LTCs are less conclusive. Future research needs to explore why this might be, and whether different approaches are needed, with specific intervention components, to address the needs of people with multiple physical LTCs, alongside identifying which outcomes are most appropriate to measure this mechanism of action (29).

For peer review only

Limitations

Double screening of eligible papers was completed for a percentage of the identified studies, meaning some eligible papers could have been missed. However, through piloting good consensus was found and the lead reviewer (T.R.) was most familiar with the eligibility criteria, the papers of interest, and the process of screening.

Complex interventions are difficult to define and therefore, determining whether a study was eligible based on this criterion and grouping interventions for subgroup meta-analysis can be a challenge. To overcome this potential bias, if there was any doubt, studies were included and discussed with a second (D.N.) and sometimes a third (R.F.) reviewer to establish consensus about eligibility and grouping, to make this process as consistent as possible.

Despite using a random-effects model, many of the meta-analyses results had high heterogeneity, suggesting that they may not have been comparable. Pre-planned sub-group analysis was conducted to explore this. For psychological distress, the cognitive and/or behavioural activation intervention type and grouping interventions lasting for longer than 6-months and into physical-physical health conditions appeared to lower heterogeneity. Other sources of heterogeneity may be the different outcome measures used for each outcome across the studies. Therefore, the analyses pooling other intervention types and conditions should be viewed with caution, and indicative of areas for further research, rather than as definitive.

Searches were conducted over a year prior to submission. A full updated search was not possible, due to team resources and funding. The search was rerun in Medline and an additional 356 papers were identified and a brief screen found one paper which may have been eligible (30). This intervention focussed on behaviour change in people with depression and type 2 diabetes and found positive outcomes for clinical outcomes and psychological outcomes, which confirm the conclusions of this review and meta-analysis.

Conclusions

In conclusion, effective complex interventions to improve one or more outcomes of people with M-LTCs are possible. For those with a combination of physical and mental LTCs, a collaborative care or a cognitive and/or behavioural activation approach may be the best approach, and emotional wellbeing and psychological distress outcomes are most likely to detect effectiveness. Evidence around those with multiple physical LTCs is less clear, however a collaborative care approach is likely the most appropriate, but the intervention components and outcome to measure this still need to be identified to ensure the best outcomes for people with M-LTCs.

Conflicts of interests

The authors have no conflicts of interest to declare.

Author Statement

TAR: Principal Investigator for the study, developed the protocol, conducted the searches, screened all the abstracts and full texts, completed outcome measure data extraction, conducted meta-analysis and narrative synthesis, wrote and finalised the manuscript.
DN: Co-investigator for the study, second reviewer for screening and quality assessment, supported with analysis and interpretation and reviewed the final manuscript.
RF: Supervised TR through the process, supported with development of project and protocol, acted as third reviewer when consensus could not be agreed, supported with interpretation of meta-analysis, writing up the results, and reviewed the final manuscript.
MA: Supported with development of project and protocol and with the interpretation of findings and reviewed the final manuscript.
LD: Completed data extraction for all studies included and reviewed the final manuscript.

JR: Supported with development of project and protocol and reviewed the final manuscript.
JH: Public contributor assisting with project design, finalising the research questions and identifying outcome measures of importance.
MM: Public contributor assisting with project design, finalising the research questions and identifying outcome measures of importance.
SJCT: Supported with development of project and protocol and reviewed the final manuscript.
KW: Supervised TR through the process, supported with development of project, protocol, and analysis plan and reviewed the final manuscript.

Ethical approval
Ethical approval was not obtained for this study, as no participants were directly involved and consented.

Data Statement
All data relevant to the study are included in the article or uploaded as supplementary information.

Funding
This study/project is funded by the National Institute for Health and Care Research (NIHR) School for Primary Care Research (572). The views expressed are those of the author(s) and not necessarily those of the NIHR or the Department of Health and Social Care.

References
1. NICE: National Institute for Health and Care Excellence. https://cks.nice.org.uk/topics/multimorbidity/background-information/prevalence/. 2023. Multimorbidity: How common is it?
2. Kingston A, Robinson L, Booth H, Knapp M, Jagger C, project M. Projections of multi-morbidity in the older population in England to 2035: estimates from the Population Ageing and Care Simulation (PACSim) model. Age Ageing. 2018;47(3):374-80.
3. Nunes BP, Flores TR, Mielke GI, Thume E, Facchini LA. Multimorbidity and mortality in older adults: A systematic review and meta-analysis. Arch Gerontol Geriatr. 2016;67:130-8.
4. Williams JS, Egede LE. The Association Between Multimorbidity and Quality of Life, Health Status and Functional Disability. Am J Med Sci. 2016;352(1):45-52.
5. Soley-Bori M, Ashworth M, Bisquera A, Dodhia H, Lynch R, Wang Y, et al. Impact of multimorbidity on healthcare costs and utilisation: a systematic review of the UK literature. Br J Gen Pract. 2021;71(702):e39-e46.
6. Afshar S, Roderick PJ, Kowal P, Dimitrov BD, Hill AG. Multimorbidity and the inequalities of global ageing: a cross-sectional study of 28 countries using the World Health Surveys. BMC Public Health. 2015;15:776.
7. Salisbury C, Johnson L, Purdy S, Valderas JM, Montgomery AA. Epidemiology and impact of multimorbidity in primary care: a retrospective cohort study. Br J Gen Pract. 2011;61(582):e12-21.
8. Skivington K, Matthews L, Simpson SA, Craig P, Baird J, Blazeby JM, et al. A new framework for developing and evaluating complex interventions: update of Medical Research Council guidance. BMJ. 2021;374:n2061.
9. Harrison M, Reeves D, Harkness E, Valderas J, Kennedy A, Rogers A, et al. A secondary analysis of the moderating effects of depression and multimorbidity on the effectiveness of a chronic disease self-management programme. Patient Educ Couns. 2012;87(1):67-73.
10. Zora S, Custodero C, Pers YM, Valsecchi V, Cella A, Ferri A, et al. Impact of the chronic disease self-management program (CDSMP) on self-perceived frailty condition: the EU-EFFICHRONIC project. Ther Adv Chronic Dis. 2021;12:20406223211056722.

For peer review only

11. Bricca A, Jäger M, Johnston M, Zangger G, Harris LK, Midtgaard J, Skou ST. Effect of in-person delivered behavioural interventions in people with multimorbidity: systematic review and meta-analysis. International Journal of Behavioral Medicine. 2023 Apr;30(2):167-89.
12. Smith SM, Soubhi H, Fortin M, Hudon C, O'Dowd T. Managing patients with multimorbidity: systematic review of interventions in primary care and community settings. BMJ. 2012;345:e5205.
13. Smith SM, Wallace E, O'Dowd T, Fortin M. Interventions for improving outcomes in patients with multimorbidity in primary care and community settings. Cochrane Database Syst Rev. 2016;3(3):CD006560.
14. Bratzke LC, Muehrer RJ, Kehl KA, Lee KS, Ward EC, Kwekkeboom KL. Self-management priority setting and decision-making in adults with multimorbidity: a narrative review of literature. Int J Nurs Stud. 2015;52(3):744-55.
15. Smith SM, Wallace E, Clyne B, Boland F, Fortin M. Interventions for improving outcomes in patients with multimorbidity in primary care and community setting: a systematic review. Syst Rev. 2021;10(1):271.
16. Page MJ, McKenzie JE, Bossuyt PM, Boutron I, Hoffmann TC, Mulrow CD, et al. The PRISMA 2020 statement: an updated guideline for reporting systematic reviews. BMJ. 2021;372.
17. Ouzzani M, Hammady H, Fedorowicz Z, Elmagarmid A. Rayyan-a web and mobile app for systematic reviews. Syst Rev. 2016;5(1):210.
18. Sterne JA-O, Savović J, Page MJ, Elbers RG, Blencowe NS, Boutron I, et al. RoB 2: a revised tool for assessing risk of bias in randomised trials. (1756-1833 (Electronic)).
19. Review Manager Web (RevMan Web). Available at revman.cochrane.org: The Cochrane Collaboration; 2020.
20. Higgins JP, Thompson Sg Fau - Deeks JJ, Deeks Jj Fau - Altman DG, Altman DG. Measuring inconsistency in meta-analyses. (1756-1833 (Electronic)).
21. Heath G, Cooke R, Cameron E. A Theory-Based Approach for Developing Interventions to Change Patient Behaviours: A Medication Adherence Example from Paediatric Secondary Care. Healthcare (Basel). 2015;3(4):1228-42.
22. Michie S, van Stralen MM, West R. The behaviour change wheel: a new method for characterising and designing behaviour change interventions. Implement Sci. 2011;6:42.
23. Oades LG, Ozturk C, Hou H, Slemp GR. Wellbeing literacy: A language-use capability relevant to wellbeing outcomes of positive psychology intervention. The Journal of Positive Psychology. 2020;15(5):696-700.
24. Huguet A, Rao S, McGrath PJ, Wozney L, Wheaton M, Conrod J, et al. A Systematic Review of Cognitive Behavioral Therapy and Behavioral Activation Apps for Depression. PLoS One. 2016;11(5):e0154248.
25. Orgeta V, Brede J, Livingston G. Behavioural activation for depression in older people: systematic review and meta-analysis. Br J Psychiatry. 2017;211(5):274-9.
26. Coventry P, Lovell K, Dickens C, Bower P, Chew-Graham C, McElvenny D, et al. Integrated primary care for patients with mental and physical multimorbidity: cluster randomised controlled trial of collaborative care for patients with depression comorbid with diabetes or cardiovascular disease. BMJ. 2015;350:h638.
27. Kappelin C, Carlsson AC, Wachtler C. Specific content for collaborative care: a systematic review of collaborative care interventions for patients with multimorbidity involving depression and/or anxiety in primary care. Fam Pract. 2022;39(4):725-34.
28. Hoffmann TC, Glasziou PP, Boutron I, Milne R, Perera R, Moher D, et al. Better reporting of interventions: template for intervention description and replication (TIDieR) checklist and guide. BMJ. 2014;348:g1687.
29. Smith SM, Bayliss EA, Mercer SW, Gunn J, Vestergaard M, Wyke S, et al. How to Design and Evaluate Interventions to Improve Outcomes for Patients with Multimorbidity. Journal of Comorbidity. 2013;3(1):10-7.
30. Hoyo ML, Rodrigo MT, Urcola-Pardo F, Monreal-Bartolomé A, Ruiz DC, Borao MG, et al. The TELE-DD Randomised Controlled Trial on Treatment Adherence in Patients with

For peer review only

Type 2 Diabetes and Comorbid Depression: Clinical Outcomes after 18-Month Follow-Up. International Journal of Environmental Research and Public Health. 2022;20(1):328.

For peer review only

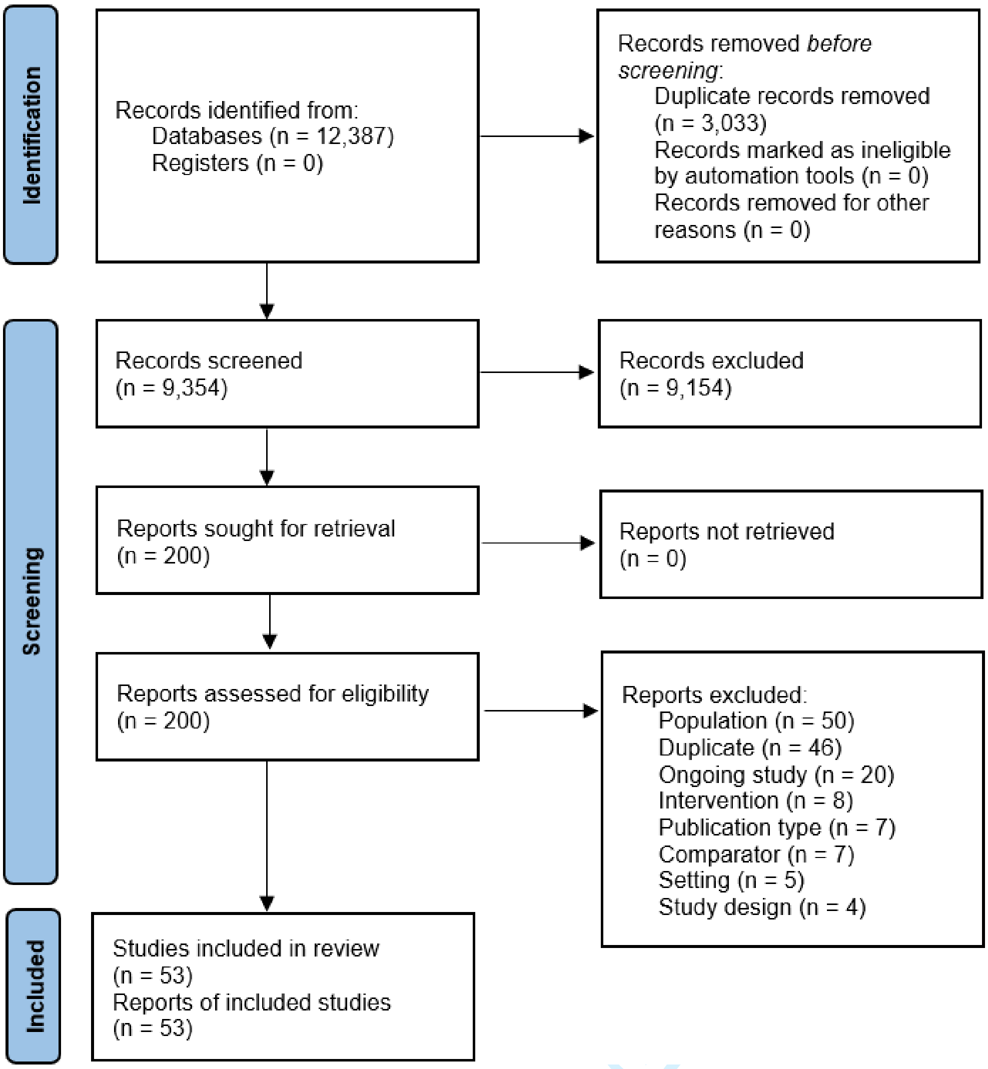

Figure 1. Prisma flow chart. (16)

PRISMA 2020 Checklist

Section and Topic	Item #	Checklist item	Location where item is reported
**TITLE**			
Title	1	Identify the report as a systematic review.	Page 1
**ABSTRACT**			
Abstract	2	See the PRISMA 2020 for Abstracts checklist.	Page 1
**INTRODUCTION**			
Rationale	3	Describe the rationale for the review in the context of existing knowledge.	Page 1 and 2
Objectives	4	Provide an explicit statement of the objective(s) or question(s) the review addresses.	Page 2
**METHODS**			
Eligibility criteria	5	Specify the inclusion and exclusion criteria for the review and how studies were grouped for the syntheses.	Page 3
Information sources	6	Specify all databases, registers, websites, organisations, reference lists and other sources searched or consulted to identify studies. Specify the date when each source was last searched or consulted.	Page 2 and 3
Search strategy	7	Present the full search strategies for all databases, registers and websites, including any filters and limits used.	Supplementary figure 1
Selection process	8	Specify the methods used to decide whether a study met the inclusion criteria of the review, including how many reviewers screened each record and each report retrieved, whether they worked independently, and if applicable, details of automation tools used in the process.	Page 2 and 3
Data collection process	9	Specify the methods used to collect data from reports, including how many reviewers collected data from each report, whether they worked independently, any processes for obtaining or confirming data from study investigators, and if applicable, details of automation tools used in the process.	Page 3
Data items	10a	List and define all outcomes for which data were sought. Specify whether all results that were compatible with each outcome domain in each study were sought (e.g. for all measures, time points, analyses), and if not, the methods used to decide which results to collect.	Page 3
	10b	List and define all other variables for which data were sought (e.g. participant and intervention characteristics, funding sources). Describe any assumptions made about any missing or unclear information.	Page 3
Study risk of bias assessment	11	Specify the methods used to assess risk of bias in the included studies, including details of the tool(s) used, how many reviewers assessed each study and whether they worked independently, and if applicable, details of automation tools used in the process.	Page 3
Effect measures	12	Specify for each outcome the effect measure(s) (e.g. risk ratio, mean difference) used in the synthesis or presentation of results.	Page 3
Synthesis methods	13a	Describe the processes used to decide which studies were eligible for each synthesis (e.g. tabulating the study intervention characteristics and comparing against the planned groups for each synthesis (item #5)).	Page 3
	13b	Describe any methods required to prepare the data for presentation or synthesis, such as handling of missing summary statistics, or data conversions.	Page 3
	13c	Describe any methods used to tabulate or visually display results of individual studies and syntheses.	Supplementary table 2
	13d	Describe any methods used to synthesize results and provide a rationale for the choice(s). If meta-analysis was performed, describe the model(s), method(s) to identify the presence and extent of statistical heterogeneity, and software package(s) used.	Page 3
	13e	Describe any methods used to explore possible causes of heterogeneity among study results (e.g. subgroup analysis, meta-regression).	Page 3
	13f	Describe any sensitivity analyses conducted to assess robustness of the synthesized results.	N/A
Reporting bias assessment	14	Describe any methods used to assess risk of bias due to missing results in a synthesis (arising from reporting biases).	Page 3

For peer review only - http://bmjopen.bmj.com/site/about/guidelines.xhtml

PRISMA 2020 Checklist

Section and Topic	Item #	Checklist item	Location where item is reported
Certainty assessment	15	Describe any methods used to assess certainty (or confidence) in the body of evidence for an outcome.	Page 10
**RESULTS**			
Study selection	16a	Describe the results of the search and selection process, from the number of records identified in the search to the number of studies included in the review, ideally using a flow diagram.	Page 4
	16b	Cite studies that might appear to meet the inclusion criteria, but which were excluded, and explain why they were excluded.	Page 4
Study characteristics	17	Cite each included study and present its characteristics.	Supplementary figure 2 and supplementary table 2
Risk of bias in studies	18	Present assessments of risk of bias for each included study.	Supplementary table 2
Results of individual studies	19	For all outcomes, present, for each study: (a) summary statistics for each group (where appropriate) and (b) an effect estimate and its precision (e.g. confidence/credible interval), ideally using structured tables or plots.	Tables 2, 3, 4 and supplementary table 4
Results of syntheses	20a	For each synthesis, briefly summarise the characteristics and risk of bias among contributing studies.	Page 5
	20b	Present results of all statistical syntheses conducted. If meta-analysis was done, present for each the summary estimate and its precision (e.g. confidence/credible interval) and measures of statistical heterogeneity. If comparing groups, describe the direction of the effect.	Tables 2, 3, 4 and supplementary table 4
	20c	Present results of all investigations of possible causes of heterogeneity among study results.	Page 7, 8, 9
	20d	Present results of all sensitivity analyses conducted to assess the robustness of the synthesized results.	N/A
Reporting biases	21	Present assessments of risk of bias due to missing results (arising from reporting biases) for each synthesis assessed.	Page 7, 8, 9
Certainty of evidence	22	Present assessments of certainty (or confidence) in the body of evidence for each outcome assessed.	Page 10
**DISCUSSION**			
Discussion	23a	Provide a general interpretation of the results in the context of other evidence.	Page 9 and 10
	23b	Discuss any limitations of the evidence included in the review.	Page 10
	23c	Discuss any limitations of the review processes used.	Page 10
	23d	Discuss implications of the results for practice, policy, and future research.	Page 10
**OTHER INFORMATION**			
Registration and protocol	24a	Provide registration information for the review, including register name and registration number, or state that the review was not registered.	Page 2
	24b	Indicate where the review protocol can be accessed, or state that a protocol was not prepared.	Page 2
	24c	Describe and explain any amendments to information provided at registration or in the protocol.	N/A
Support	25	Describe sources of financial or non-financial support for the review, and the role of the funders or sponsors in the review.	Page 11
Competing	26	Declare any competing interests of review authors.	Page 11

For peer review only

For peer review only - http://bmjopen.bmj.com/site/about/guidelines.xhtml

PRISMA 2020 Checklist

Section and Topic	Item #	Checklist item	Location where item is reported
interests			
Availability of data, code and other materials	27	Report which of the following are publicly available and where they can be found: template data collection forms; data extracted from included studies; data used for all analyses; analytic code; any other materials used in the review.	Page 11

From: Page MJ, McKenzie JE, Bossuyt PM, Boutron I, Hoffmann TC, Mulrow CD, et al. The PRISMA 2020 statement: an updated guideline for reporting systematic reviews. BMJ 2021;372:n71. doi: 10.1136/bmj.n71

For more information, visit: http://www.prisma-statement.org/

For peer review only

Lead Author<br>Year<br>Country	Participant condition 1<br>Participant condition 2<br>Other conditions	LTC combination	Study n<br>Intervention n<br>Control n<br>Carers n	Intervention length<br>Follow-up timepoints	Primary outcome<br>Timepoint<br>Effectiveness	Category	Quality assessment (RoB-2)	Intervention name, description and modifications	Intervention components<br>Leader<br>Recipient<br>One-to-one or Group<br>Mode of delivery and location<br>Number/length sessions
Ali<br>2020<br>India	Depression<br>Type 2 diabetes	Physical-Mental	404<br>196<br>208	12 months<br>Baseline, 6, 12, 18, 24 months	Composite depression and clinical<br>24 months<br>Yes	Collaborative care	Low	Collaborative care intervention targetting patient-, clinician-, and system-level improvements. Administered PHQ-9 to monitor depression, reviewed glucose logs, and counseling toward achieving treatment goals. Intergrated decision making tool for HCPs within electronic health record system	Psychiatrist and diabetologist and care co-ordinators<br>Person with depression and T2DM<br>One-to-one<br>Face-to-face in clinic or telephone<br>Every 2-4 weeks for 12 months

For peer review only

Aragones 2019 Spain	Depression and Chronic Musculoskeletal Pain	Physical-Mental	41<br>21<br>20	3 months Baseline, 3, 6, 12 months.	Depression 12 months Yes	Self-management	Low	DROP (DepRessiOn and Pain) programme:<br>1. Optimised management of major depression through computerised clinical guideline to aid decision making<br>2. Care management of patient pain through calls with psychologist<br>3. Psychoeducational intervention group programme, with discussions and homework covering: understanding pain; managing emotions and attention; basic relaxation techniques; cognitive restructuring strategies; problem-solving; setting life goals; relationships between pain and physical activity; healthy posture and sleep; maintenance of the strategies learned; and a relapse plan	Care co-ordinator (psychologist)<br>Primary care physicians and people with conditions<br>Primary Care Centres<br>Face-to-face - 9 weekly 2 hour group sessions<br>Telephone - Monthly for first 3 months and then every 3 months for next 9 months

For peer review only

Barley, 2014, UK	Coronary heart disease<br>Depression	Physical-Mental	81<br>41<br>40	6 months. Baseline, 1, 3, 6, 12 months.	N/A Feasibility and acceptability	Self-management	Low	Personalised Care: the nurse case manager conducts a standardised, face to face, biopsychosocial assessment. Patients then identify up to three problems which contribute to their depression and they want to address. The nurse-case managers provide information, sign-post to existing resources and use evidence based behaviour change techniques to help patients set and achieve goals. The underlying intention of the intervention is to increase the patient's self-efficacy to achieve their desired goals. The action plan is recorded in a 'personalised health plan' which the patient holds.	Nurse<br>Person with CHD and depression<br>One-to-one<br>Face-to-face and telephone<br>1 hour session<br>Weekly 15 minute phone calls for 6 months
Barrowclough, 2001, UK	Schizophrenia<br>Substance Abuse disorder	Mental-Mental	33 patient carer dyads	9 months. Baseline, 9 months and 12 months.	Functioning 9 and 12 months<br>Yes	Cognitive and/or Behavioural Activation	Some concerns	Allocated a family support worker from Making Space, providing information, giving advice on benefits, advocacy, emotional support, and practical help. The integrated treatment program attempted to combine three treatment approaches: motivational interviewing, individual cognitive behavior therapy, and family or caregiver intervention.	Family support worker<br>Patient and primary care giver dyad<br>Face-to-face<br>Home or clinic<br>23 weekly sessions followed by 6 biweekly sessions and 10-16 family sessions

For peer review only

Barr Taylor, 2003, USA	Diabetes<br>And one or more of:<br>Hypertension<br>CVD<br>Dyslipidemia	Physical-Physical	169<br>84<br>85	12 months<br>Baseline, 12 months	Clincal outcome (HbA1c)<br>12 months<br>Yes	Collaborative care	Low	Nurse care management system. Initial consultation to review medical, lifestyle, and social status, and develop a self-management action plan. Group classes followed a workbook specifically created for the program and included some didactic material, although the focus was on group discussion, participation and problem-solving. Follow-up calls were used to review patient goals, medication use, glucose/BP monitoring, and self-management activities. Higher levels of depression and alcohol problems were also addressed. Algorithims were used throughout for medication managament.	Nurse care manager<br>Patient<br>Group classes once a week for 4 weeks (1-2 hours)<br>8 Follow-up telephone calls lasted 15 minutes, over 9 months.

For peer review only

Battersby, 2013, Australia	Alcohol use disorder AND one or more of:<br>-Cardiovascular<br>-Musculoskeletal<br>-Gastrointestinal<br>-Respiratory<br>-Diabetes<br>-Skin conditions<br>-Cancer<br>-Genitourinary<br>-Alcohol dependence<br>-PTSD<br>-Major depression<br>-Generalised Anxiety Disorder<br>-Panic Disorder	Physical-Mental	77<br>46<br>31<br>SCDSMP course = 22 veterans and 15 of their partners from intervention group	9-month intervention. Seen at 1, 3, 6 and 9 months, with telephone calls at 4.5 and 7.5 months<br>Follow-up measures at 18 months	Behaviour change (AUDIT)<br>9 months<br>Yes	Self-management	High	Flinders Programme<br>Targeted self-management support to assist the patient to achieve improved health outcomes over a period of 12 months. A health professional is trained to tailor the interventions to meet the persons needs whilst simultaneously motivating the patient to achieve their medical, psychosocial, or lifestyle risk factor goals. Veterans offered SCDSMP course (Stanford School of Medicine) if considered appropriate - this builds the person's self-efficacy by teaching skills in problem-solving, decision-making, resource utilisation, managing the patient–provider partnership, action planning and emotional management	Research nurse (RN)<br>Veterans<br>One-to-one<br>Face-to-face: begins with developing care plan, follow-up visits monitor outcome of this plan<br>SCDSMP: 6 weekly face-to-face group sessions (2.5 hours each) delivered jointly by research officer and veteran peer leader to veterans and their partners if desired

For peer review only

Blank 2011, USA	HIV<br>Schizophrenia Spectrum Disorder/ Affective Disorder/ Other serious mental ilness	Physical-Mental	238<br>128<br>110	12 months. Baseline, 3 months, 6 months, 12 months<br>Follow-up at 24 months.	Clinical (Viral load)<br>12 months<br>No	Self-management	Some concerns	Preventing Aids Through Health (PATH)<br>Driven by theory of reasoned action; helps patients actively cope with barriers to medication adherence and promote participants self-care abilities. The protocol includes one face-to-face meeting a week as a minimum with basic intervention (psychoeducation, pillboxes, beeping watches). The intervention was then cascaded to suit the needs of the individual if adherence fell - this was a gradual increase in intensity and included activation of social networks, followed by use of beepers with alphanumeric displays, then prepaid cellular phones to encourage participants to follow their regimen, then the final step was directly observed therapy.	Advanced Practice Nurse (same nurse each session where possible)<br>Patient<br>One-to-one<br>Community-based: weekly face-to-face home visits from the APN
Boeschoten, 2017, The Netherlands	MS<br>Depression<br>Anxiety	Physical-Mental	171<br>85<br>86	5 to 10 weeks.<br>Baseline, within a week of intervention, 4 months	Depression (BDI-II)<br>5-10 weeks and 4 months<br>No	Cognitive and/or Behavioural Activation	Low	"Minder Zorgen" (Worry Less) - a guided self help intervention using Cognitive Behavioural Therapy with a focus on coping skills. Five modules with texts, examples and assignments that patients could access online from their own computer. This intervention was an existing IPST and adapated for MS patients.	Psychologists and masters student<br>Patient<br>One-to-one<br>Online - digital<br>One module a week for 10 weeks

For peer review only

Bogner, 2008, USA	Hypertension Depression	Physical-Mental	64 32 32	4 weeks. Baseline, 2, 4 and 6 weeks.	Depression (CES-D) 6 weeks Yes	Collaborative care	Low	Integrated Care Intervention. Involved education about depression and hypertension, emphasizing the importance of controlling depression to manage hypertension; encouragement and relief from stigma; helped to identify target symptoms for both conditions; explained the rationale for antidepressant and antihypertensive medication usage; assessed for side-effects and assisted in their management; assessed progress; assisted with referrals; and monitored and responded to life-threatening symptoms.	Integrated care manager (ICM) Patient One-to-one 3, 30-minute in-person sessions and 2, 15-minute telephone-monitoring contacts during the 4 weeks

For peer review only

Chwastiak, 2018, USA	Type II diabetes<br>Schizophrenia<br>Schizoaffective disorder<br>Bipolar disorder<br>Major depressive disorder with psychosis	Physical-Mental	35<br>18<br>17	3 months. Baseline, 3 months.	Clinical (HbA1c)<br>3 months<br>N/A test not completed	Collaborative care	Low	CMHC-based collaborative care model, adapted from the primary care–based TEAMcare model and adapted for patients with psychosis.<br>Initial 60-min nurse care manager visit for a comprehensive health assessment and an individualized health plan and then 30-minute visits for the support of chronic illness self-management (including medication adherence,<br>healthy nutrition, and regular physical activity) every other week for 12 weeks. Nurses used motivational interviewing and behavioural activation to address barriers to self management.<br>Diabetic education materials were modified to address needs of patients with psychosis.	A CMHC nurse care manager, a CMHC psychiatrist, the advanced practice registered nurse who provided primary care onsite at the CMHC, and an endocrinologist consultant.<br>Patients<br>One-on-one<br>Hospital clinic

For peer review only

Cummings, 2019, USA	Type 2 diabetes<br>Diabetes-related distress or depression	Physical-Mental	139<br>67 (CBT=48; Lifestyle=19)<br>72	12 months. Baseline, 6 months (to adjust intervention if necessary), 12 months.	Clinical (HbA1c)<br>12 months<br>No (approached p=0.06)	Cognitive and/or Behavioural Activation	Low	CBT (plus usual care): Session content used cognitive techniques to identify and challenge general and diabetes-specific cognitive distortions that result in maladaptive behavior, in combination with behavioral techniques, including behavioral activation and specific behavior change strategies related to diabetes and/or mood. Those with more intermediate concerns were also provided with problem solving therapy focusing on effective coping and adaptive problem-solving skills.<br>Small Changes Lifestyle Coaching (plus usual care): Focused on lifestyle modifications to improve diabetes and mood.<br>Patients were reevaluated at 6 months and either entered a maintenance phase, or switched to CBT or SCLC.	SCLC - nurse care manager; CBT - a psychologist and clinical health psychology doctoral student; and a community health worker provided navigation and social support.<br>Patient.<br>One-on-one.<br>12 tailored sessions 30-60 minutes long for the first 6 months of either:<br>CBT: Face-to-face or over the telephone if necessary.<br>Lifestyle: over telephone

For peer review only

Druss, 2018, USA	Medical illness<br>Diabetes<br>Heart disease<br>Hyperlipidemia<br>Hypertension<br>Arthritis<br>Hepatitis<br>Asthma/COPD<br>HIV<br>SMI<br>Schizophrenia/ schizoaffective<br>Bipolar disorder<br>Depression<br>OCD<br>PTSD	Physical-Mental	400<br>198<br>202	3 months. Baseline, 3 months, 6 months.	Quality of life<br>6 months<br>Yes	Self-management	Some concerns	Health and Recovery Peer Programme (HARP)<br>The program builds on the Chronic Disease Self-Management Program (CDSMP), a 6-session health education program. It provides skills that help patients become more effective self-managers including modeling of healthy behaviors, problem-solving, reinterpretation of symptoms, and training in specific disease management techniques. Action plans help develop self-efficacy and build to long-term health goals.<br>Modifications of CDSMP to HARP addressed specific patient, provider, and community level determinates of health in patients with comorbid medical and serious mental disorders.<br>Materials were added addressing the mental health impact of chronic conditions and dealing with social isolation.	Two certified peer specialists led each session.<br>Patient.<br>Six 2.5 hour face-to-face sessions in groups of 6-10, with one-on-one peer coaching meetings held in between sessions for reinforcement.

For peer review only

Ell, 2010, USA	Type 1 or 2 diabetes Depression	Physical-Mental	387 193 194	12 months. Baseline, 6, 12, and 18 months.	Depression (SLC-20) 6-12-18 months Yes	Collaborative care	Low	Multifaceted Diabetes and Depression Program (collaborative care intervention). Patients chose problem-solving therapy or antidepressant, and a stepped-care algorithm was used to ensure patients received treatment consistent with their preference, clinical presentations and responses over time. Usual care was enhanced by providing patients with depression educational pamphlets and a community resource list.	1) PST provided by bilingual graduate social work diabetes depression clinical specialists (DDCSs) and/or antidepressant medications prescribed by the treating primary care physician. Patient. One-to-one but support group sessions available.

For peer review only

Ell, 2017, USA	Depression Diabetes OR heart disease OR both	Physical-Mental	348 178 170	6 weeks (plus 3 monthly boosters). Baseline, 6 months, 12 months.	Depression (PHQ-9) 12 months No	Self-management	Some concerns	A-Helping-hand (AHH) Psychoeducational intervention following a problem-solving framework, using self-care management of chronic care conditions as a specific skill-building focus with review of healthy behaviors outlined in the written materials provided to patients. Intervention provided problem-solving modeling and opportunities for patients to practice problem solving skills: 6 tasks included engagement, problem formulation, education, action planning and evaluation. Study participants in both groups were given depression educational brochures, depression photo novels, and materials on specific chronic illness treatments and self-care.	3 promotoras (trained and supervised by the research team) were added to a standard team of physician, nurse, and medical assistant. Patient. One-to-one. Six sessions over six weeks administered face-to-face (or telephone if preferred) at the patients home/any other setting preferred by patient, followed by 3 monthly booster sessions for reinforcement.

For peer review only

Fisher, 2020, Canada	At least 3 chronic conditions<br>Most common:<br>Cardiovascular<br>Kidney/Urogenital<br>Arthritis<br>Gastrointestinal<br>Endocrine<br>Hearing/Vision	Physical-Physical	59<br>30<br>29	6 months. Baseline and 6 months.	SF-12 Physical<br>6 months<br>No	Self-management	High	Self-management intervention. An individualised care plan was developed to help self-management of chronic conditions, and caregivers were actively consulted in all aspects of care. Focus on client building on their strengths and abilities to enhance self-efficacy and achieve personal goals.<br>Adapted from two previous community-based self-management interventions for older adults (targeting diabetes and stroke) to target multi-morbidities.	Care Coordinator, Registered Nurse, Physiotherapist, Occupational Therapist, and Personal Support Worker<br>Patient and informal/family caregiver.<br>One-to-one<br>1. Minimum of one in-home visit from CC and 3 from PT/OT.<br>2. Monthly case conferences with provider team to discuss ongoing care plan.<br>3. Ongoing case management by CC's which involved system navigation and facilitating participant access to appropriate health and social services.

For peer review only

Garvey, 2015, Ireland	43 chronic coniditions identified, most common: Arthritis Congestive cardiac failure Diabetes Depression Hypertension	Physical-Physical	50 26 24	6 weeks. Baseline and 6 weeks	N/A (participation) 6 weeks yes	Self-management	Some concerns	Occupational therapy led self-management support programme (OPTIMAL). Occupational therapy focus with peer support; goals were discussed and revised at each session, considering patient preference. Intervention components included: self-management; fatigue and energy management; managing stress/anxiety and maintaining mental health/wellbeing; keeping physically active; healthy eating; managing medications; effective communication strategies; goal setting. Based on the Stanford Chronic Disease Self-Management Programme: key adaptations were an occupational therapy focus, groups being professionally led and a clear focus on the specific challenges of multimorbidity.	Facilitated and led by local community-based occupational therapists. Patient. Group. Weekly 3-hour meetings for 6 weeks held in local community health centres.

For peer review only

Goldberg, 2013, USA	Schizophrenia spectrum disorder OR bipolar disorder<br>AND one or more chronic condition, including:<br>Diabetes<br>Arthritis<br>Respiratory disease<br>Cardiovascular disease	Physical-Mental	63<br>32<br>31	3 months<br>Baseline, 3 months and 5 months	SF-12 Physical<br>3 months<br>Yes	Self-management	Some concerns	Living Well.<br>Sessions focused on action planning, peer feedback, support, modeling, problem solving, training in specific disease management techniques and the application of these skills to the topics of nutrition, exercise, sleep, medication management, addiction behaviours.<br>Modified from CDSMP for patients with serious mental illness as follows - delivery by two mental health peers OR mental health provider and peer; increased length of intervention; action plan tracker and health workbook; extra module on communication with HCP's; extra topics on mental illness; two monthly booster sessions after intervention.	Six groups - four were cofacilitated by two mental health peers who had at least one chronic condition, two were cofacilitated by a mental health professional and a peer.<br>Patients.<br>Group.<br>Weekly 60-75 minute face-to-face sessions and two monthly booster sessions post-intervention.<br>Clinics
Goorden, 2017, The Netherlands	Major depressive disorder<br>AND one or more chronic physical condition:<br>Diabetes<br>COPD<br>IBS<br>Heart failure	Physical-Mental	81<br>42<br>39	12 months.<br>Baseline, 3, 6, 9, 12 months.	N/A cost effectiveness<br>12 months	Collaborative care	High	Collaborative Care<br>Guided self-help and problem-solving treatment, prescription of anti-depressant medications according to an algorithm, and consultations with psychiatrist if necessary.	Provided by a team consisting of patient, consultant psychiatrist nurse, and consultant-liaison psychiatrist.<br>Patient.<br>One-to-one.<br>Face to face at outpatient clinic of general hospital.

For peer review only

Griva, 2019, Singapore	Diabetes<br>End stage renal disease	Physical-Mental	44<br>20<br>24	3 weeks. Baseline, 3 months	Clinical endpoint (HbA1c)<br>3 months<br>No	Self-management	Low	Combined Diabetes and Renal Control Trial (C-DIRECT) Intervention (plus usual care). Centered around self-management strategies. Patients chose a topic to focus on at each session. Session 1: feedback on Hba1c; information/advice on chosen topic; goal setting; food record assignment. Session 2: review goal setting and problem solve any barriers; revise goals as needed; review food record and provide support if necessary; choose topic (information/advice and new goal setting). Session 3: review goals and problem solve barriers; revise goals as needed; choose topic (information/advice and new goal setting). Provide additional advice and resourse links to go away with.	DM link nurse.<br>Patient.<br>One-to-one.<br>3 weekly sessions 30-60 minutes long, delivered at patient bedside at dialysis centre upon cannulation and connection to haemodialysis machine.

For peer review only

Guo, 2020, China	Atrial Fibrilliation AND Congestive heart failure Hypertension Diabetes Prior ischemic stroke Vascular disease Coronary artery disease Peripheral arterial disease Renal dysfunction Liver dysfunction Prior bleeding Prior brain bleeding Hyperthyroidism Cardiomyopathy	Physical-Physical	40 hopsitals randomised (20 to each arm) 3324 1646 1678	12 months Baseline, 6 and 12 months.	N/A composite hospital 12 months Yes	Collaborative care	Low	mobile Atrial Fibrillation Application (mAFA). Installed on smart phones. Provided clinical decision support tools for doctors to facilitate treatment recommendations, educational materials, patient self-care, and structured follow-up, to support AF management: Avoid stroke (tracking bleeding events/trends; managing treatment and anticoagulant use), Better symptom management (tracking symptoms, monitoring cardiac rhythm using smart watches), Cardiovascular and other comorbidities risk management (tracking BP and lifestyle factors). The app encouraged patient engagement through participation in educational programs, provision of informative articles, videos, game playing, etc.	Doctors. Doctors and patients. One-to-one. Followed up at outpatient clinics.

For peer review only

Hernandez-Quiles, 2021, Spain	Advanced heart/lung failure/both.<br>AND one of:<br>Hypertension<br>Heart Failure<br>Atrial fibrillation<br>Dyslipemia<br>Diabetes<br>CHD<br>COPD<br>Chronic kidney disease<br>Obesity<br>Chronic anemia<br>Valvular heart disease<br>Pulmonary hypertension<br>Sleep apnea hypopnea syndrome<br>Hypothyroidism<br>Depression	Physical-Physical	510<br>255<br>255	180 days<br>0, 15, 45, 90, 180 days.	N/A hospital admissions<br>45, 90 and 180 days<br>Yes	Self-management	Low	TELECARE (plus usual care).<br>Usual care was frequent clinical and bio-parameters self-checks (BP, heart rate, oxygen saturation, weight, and blood glucose)Patients were provided with educational material and instructed how to recognize exacerbation symptoms. For the intervention group, a synchronous monitoring equipment was added for biological and medical questionnaires so data was available in real time for healthcare team. An alarm system was added to induce a healthcare response if there was variation in data or missing data.	Health care team<br>Patient tracked own data.<br>One-to-one.<br>Patients inserted data daily for first 15 days (frequency was then altered depending on stability and ocurrence of incidents).

For peer review only

House, 2018, UK	Type 2 diabetes<br>Obesity<br>Intellectual disability	Physical-Physical	82<br>41<br>41	4 months<br>Baseline, 6 month follow-up (reduced to 4 because of project deadlines)	Clinical endpoint (HbA1c)<br>4-6 months<br>No	Self-management	Some concerns	Supported self-management<br>The intervention emphasized realistic goal setting, identifying resources and barriers likely to influence success in reaching goals, and regular self-monitoring of goal attainment.<br>Four components: (1) Establishing the participant's daily routines and lifestyle; (2) Identifying all supporters and helpers and their roles; (3) Setting realistic goals for change; (4) Monitoring progress against agreed upon goals.<br>Materials: for nurses - weekly timetable, charts to record friends/family/helpers, charts to complete in collaboration with participant; for participants - OK Diabetes board to record goals and visual prompts, a written action plan, tear off slips to record daily actions; for supporters/helpers - information sheet on study, card summarising their role in helping the person with diabetes.<br>The intervention was modelled on existing self-management interventions, with adjustments [illegible]	Diabetes specialist nurse.<br>Patient (73% had their supporter present for at least one session).<br>One-to-one.<br>4 sessions, most (92%) took place in participants homes, ranging from 13-95 minutes (typical total intervention time was 2 hours).

For peer review only

Jackson, 2021, Nigeria	HIV<br>Hypertension<br>Other:<br>Diabetes<br>Peptic ulcer disease<br>Renal disease<br>Hypertensive heart disease<br>Osetoarthritis<br>Haemorrhoids<br>Benign prostatic hyperplasia	Physical-Physical	206<br>103<br>103	12 months. Baseline, 6 months, 12 months.	Clinical outcomes (Blood pressure)<br>12 months<br>Yes	Self-management	Low	Pharmaceutical care (PC) (plus routine care).<br>Routine care - BP taken by nurse, physician prescribes medication/orders lab tests, meeting with adherence counsellor, then medication/ counselling from pharmacist.<br>Intervention also exposed to PC from research pharmacist. This involved structured education/ counselling focused on self-monitoring of BP, healthy eating, maintaining an ideal body weight, proper foot and dental care, healthy coping, use and storage of medications; lifestyle modifications with emphasis on the dietary approaches to stop hypertension (DASH) diet, physical activity, smoking cessation, moderation of alcohol intake; reviewing date of the next appointment and prescription with the patient, expected side effects of the medications, and how to prevent/ manage such side effects.	Research pharmacist.<br>Patient.<br>One-to-one.<br>Face-to-face bi-monthly appoointments at hospital, as well as two text messages per month.

For peer review only

Kamradt, 2019, Germany	Type 2 diabetes and<br>Two or more of the following:<br>Coronary heart disease<br>Chronic pain<br>Depression<br>Chronic heart failure<br>COPD<br>Artherosclerosis<br>Cerebrovascular disease	Physical-Physical	495<br>252<br>243	9 months. Baseline, 9 months.	Quality of life<br>9 months<br>No	Collaborative care	Some concerns	Care management intervention. Embedded in a regional network of primary care practices. It included two home visits with structured assessments of clinical and social needs, aiming to reduce barriers to patient's access of support and healthcare systems. Frequent telephone monitoring was also used to improve and sustain changes in self-care behaviour, and give feedback related to patients' control of diseases and current (self-care) activities to reinforce positive behavioral changes.	Specially trained medical assistants, physicians specialising in family/internal medicine, and net-care managers.<br>Patient.<br>One-to-one.<br>Two home visits and 15 structured telephone monitoring contacts over 9 months.

For peer review only

Kanwal, 2018, USA	HCV Depression	Physical-Mental	309<br>156<br>153	6 months<br>Baseline, 6 months, 12 months.	Depression (SLC-20)<br>12 months<br>Yes	Collaborative care	Some concerns	Hepatitis C Translating Initiatives for Depression Into Effective Solutions (HEPTIDES)<br>The intervention included: participant education and activation, assessment of treatment barriers and possible resolutions, depression symptom and treatment monitoring, substance abuse monitoring, and instruction in self-management. The intervention used a stepped-care model for depression treatment, with five steps: self-management education, depression care team treatment suggestions, pharmacotherapy suggestions, combination pharmacotherapy and specialty mental health counseling, and referral to specialty mental health.	Depression care manager, pharmacist and psychiatrist. They convened once a week and communicated with HCV and mental health clinicians at each hospital site to discuss treatment and make decisions.<br>Patient.<br>One-to-one via telephone delivered by DCM, every 2 weeks during acute treatment and every 4 weeks during self-management education or continuation treatment.

For peer review only

Katon, 2004, USA	Diabetes Depression	Physical-Mental	329<br>164<br>165	3-6 months. Baseline, 3 months, 6 months, 12 months.	Depression (SLC-90)<br>6 and 12 months<br>Yes	Collaborative care	Low	Collaborative care.<br>Individualised, stepped-care depression treatment programme designed to improve quality of care and outcomes of depression. Patients were offerd an intial choice of two treatments: antidepressant medication or problem solving treatment (PST). If patients still had persistent depressive symptoms (< 50% decrease in severity based on the PHQ-9) 10 to 12 weeks after initial treatment, they could (1) switch to a second antidepressant; (2) switch to the alternative treatment (from PST to medication or vice versa); (3) receive augmentation with PST or medication with the first treatment they had received; or (4) receive a psychiatric consultation. If there was still no improvement after a further 8 to 12 weeks, referral to speciality care was made. Once patients reached a significant decrease in clinical symptoms (< 50% decrement in symptoms), the nurse began continuation phase	Depression clinical specialist nurse in collaboration with the primary care physician.<br>Patient.<br>One-to-one.<br>Initial 1-hour visit followed by twice-a-month, half-hour appointments (telephone and in person) for 12 weeks followed by monthly scheduled telephone contacts for continuation phase treatment.

For peer review only

Katon, 2010, USA	Depression Diabetes/Coronary heart disease/both	Physical-Mental	214 106 108	12 months. Baseline, 6 months, 12 months.	Composite depression and clinical 12 months Yes	Collaborative care	Low	Collaborative care management. The intervention combined support for self-care with pharmacotherapy, aiming to manage depression and improve glycemic, BP and lipid control. Patients worked collaboratively with nurses and primary care physicians to establish individualized clinical and self-care goals. Using motivational and encouraging coaching, nurses helped patients solve problems and set goals for improved medication adherence and self-care. Patients also received self-care materials and self-monitoring devices. Nurses monitored the patient's progress and medications were adjusted where necessary according to protocol. Once target levels were achieved, a maintenance plan was developed that included stress reduction, behavioral goals, continued use of medications, and identification of prodromal symptoms associated with worsening depression and glycemic control.	Nurses, in collaboration with primary care physicians. Patient. One-to-one. Visits every 2 to 3 weeks at the patients primary care clinic; once maintenance plan was in place. Patients whose disease control worsened were offered follow-up visits and telephone calls with increased treatment intensity.

For peer review only

Khunti, 2021, UK	Conditions accepted in inclusion criteria: Cardiovsacular Asthma/COPD High dependency/ LTC's Depression Musculoskeletal	Physical-Physical	353 180 173	6 months. Baseline, 6 months, 12 months.	Behaviour change (physical activity) 12 months Yes	Self-management	Low	Movement through Active Personalised engagement (MAP) programme. Four sessions with person-centred self monitoring and goal setting. The sessions focused on increasing PA, and addressed key non-disease-specific self-management challenges and themes (mastering emotions, managing treatments, communication within health care). Increasing PA was further facilitated and encouraged by providing resistance bands in order to complete resistance training at home and pedometers to help track PA. Regular reminder and motivational texts were sent to encourage long-term behaviour change.	Trained facilitator. Patient. Group. 1.5 hour face-to-face sessions delivered at two week intervals in local community. Texts were sent daily during the 8 weeks of sessions up until 2 weeks after the 6-month timepoint, then 3 times a week over the remainder of the 12 month period.

For peer review only

Koesoemadinata, 2021, Indonesia	Tuberculosis Diabetes	Physical-Physical	108 60 48	6 months. Baseline, 6 months.	N/A patient knowledge 6 months No	Cognitive and/or Behavioural Activation	Some concerns	Structured counselling/education. At each visit, blood glucose level was measured, and DM medication was adjusted according to a structured algorithm. Educational messages were discussed according to a specified checklist using flip charts. Topics included: TB: Cause, transmission, treatment compliance, possible side effects; What is DM? What are the symptoms? What can be done?; Blood glucose measurement and recording; Importance of glycaemic control; Metformin and insulin; Hyper- and hypoglycaemia; The importance of smoking cessation; DM and TB together; Healthy lifestyle: diet, exercise, and smoking cessation; Management after the TB treatment is completed. A leaflet was also provided to patients with information regarding recommended food types and amounts.	Doctor. Patient. One-on-one. Face-to-face in a research clinic at weeks 1, 2 and 4, then monthly until 6 months.

For peer review only

Kroenke, 2009, USA	Musculoskeletal pain Depression	Physical-Mental	250 123 127	12 months. Baseline, 1, 3, 6, and 12 months.	Depression (SLC-20) 12 months Yes	Collaborative care	Low	Stepped Care for Affective Disorders and Musculoskeletal Pain (SCAMP).<br>Step 1 (weeks 1-12): Optimised antidepressant therapy. Adjustments in medication as per an algorithim to optimise antidepressant management.<br>Step 2 (weeks 13-26): Pain self-management programme. Focus on increasing self-efficacy, social support to self-manage pain, problem solving techniques, behavioural change strategies, learning about chronic pain and receiving feedback from nurse.<br>Step 3 (weeks 27-52): continuation phase. Evaluation. The care manager assessed current self-management strategies and assisted patients with new behavioral plans. Antidepressant dose could be increased, or if necessary a referral to a psychiatrist offered.	Nurse care manager and physician depression specialist.<br>Patient.<br>One-to-one.<br>5 inperson contacts (baseline, 6, 12, 16, and 20 weeks) and 8 telephone contacts (1, 3, 9, 14, 18, and 22 weeks, and at 8 and 10 months). Extra contacts could occur depending on treatment changes or a patient's clinical needs.

For peer review only

Lear, 2021, Canada	2 or more of:<br>Diabetes<br>Heart failure<br>Ischemic heart disease<br>Chronic kidney disease<br>COPD	Physical-Physical	230 (219 - one withdrew after randomisation)<br>117 (116 - see above)<br>113	Two years. Baseline, 12 months, 24 months.	N/A Hospital admissions<br>24 months<br>No	Self-management	Low	Internet chronic disease management (CDM). Participants used the CDM website to complete daily symptom reports which asked questions about disease-specific symptoms and biometric data, which generated alert for specific scenarios or targets not being met. The nurse would then call to discuss/readjust targets, and support patient self-management or recommend follow up with primary care practicioner/hospital referral if necessary. Every 8 weeks, participants answered a lifestyle questionnaire, and alerts were sent based on thresholds for these questions. If necessary the nurse gave a referral to a dietitian/exercise specialist or use of a psychosocial support workbook. Participants had access to a public forum, graphical presentations of their biometric data overlaid with relevant alerts, their action plan, and external online resources.	Coordinated care between participant, their primary care practicioner, and a nurse managing the CDM programme (supported by a dietitian and an exercise specialist).<br>Patient.<br>One-to-one.<br>Telephone.

For peer review only

Lenferink, 2019, The Netherlands and Australia	COPD and one or more of:<br>Ishemic heart disease<br>Chronic heart failure<br>Diabetes<br>Anxiety<br>Depression	Physical-Physical	201<br>102<br>99	4 weeks intensive and 3 follow up calls at weeks 8, 20 and 36<br>Baseline, 6 months, 12 months.	COPD exacerbation<br>12 months<br>No	Self-management	Some concerns	Self-management intervention. Week 1: knowledge on COPD/ comorbidities, symptom recognition/ monitoring, self-treatment action plan (linked to diary), breathing/relaxation exercises, how to check blood glucose. Week 2: record 'usual symptoms', diary training, exacerbation action plan training, mastery of skills (e.g. inhaler use). Week 3: importance of exericse, diet/lifestyle behaviours, re-iteration of diary/action plan use, reiteration of breathing/relaxation. Week 4: re-iteration of diary/action plan use, feedback on diary completion, feedback on actions.<br>Both intervention and usual care groups completed daily symptom diaries for the 12 month period. For the intervention group, self-management behaviours were reinforced, and exacerbation action plans for all co-morbidities were directly linked to their diaries.	Researchers<br>Patient.<br>Weeks 1 and 3: group. Weeks 2 and 4: individual. Phone calls at weeks 8, 20, and 36.<br>Location of face-to-face sessions unspecified.

For peer review only

Markle-Reid, 2018, Canada	Type 2 diabetes<br>Top 3 most common chronic conditions:<br>Hypertension<br>Cardiovascular<br>Arthritis	Physical-Physical	159<br>80<br>79	6 months. Baseline, 6 months.	SF-12 physical and mental<br>6 months<br>Mental - yes<br>Physical - no	Self-management	Low	Self-management programme. The intervention focused on self-efficacy, self-management, holistic care, and individual and caregiver engagement. It was client-driven therefore fleixble to client needs; the indvidual was fully engaged in developing and tailoring their own care plan. The programme offered up to 3 in-home visits from nurse/dietitian/both, monthly group wellness sessions, monthly case conferences amongst the team, and ongoing nurse-led care coordination.	Nurses and dietitians, a program coordinator from a community partner, and peer volunteers.<br>Patient and carer (optional)<br>One-on-one sessions at patients home, group sessions at diabetes education centre.

For peer review only

McDermott, 2015, Australia	Type 2 diabetes One or more other significant comorbidity	Physical-Physical	213 100 113	18 months. Baseline, 18 months.	Clinical (HbA1c) 18 months yes	Self-management	Low	"Getting better at chronic care". Health-worker led case management intervention (one recruited at each site to work as part of primary care team and given caseload of 9 to 26 clients). The roles of the health workers included helping patients make and keep appointments, understand their medications and nutrition and the effects of smoking and work with the family to help support the patient in self-management. Home visits and out-of-clinic care were conducted according to patient preference. Specific training and practice in: chronic care model and evidence-based management and treatment goals for chronic conditions; "hands-on" case management (regular client home visits, including basic diabetes care); working in a primary care team with clear roles/ responsibilities; engaging with families and using local resources to support effective client self-management.	Indigenous healthcare worker (IHW) from community. Health worker/client. One-to-one. Home visits OR out of clinc care (based on preference).

For peer review only

Merlin, 2018, USA	HIV Chronic pain	Physical-Physical	44 22 22	12 weeks. Baseline, 12 weeks	Pain (BPI) 12 weeks no	Self-management	Some concerns	Skills TO Manage Pain (STOMP) plus usual care. Incorporated self-regulation, self-efficacy, observational learning, and outcome expectations. Individual sessions: to build pain self-management skills. All participants received a pain education session, and then selected 5 of the remaining 9 sessions (physical activity and your pain, losing weight to improve your pain, relaxation skills to prevent your pain, sleeping better to help your pain, thinking diferently about your pain, building self-worth, talking with our family and friends about pain, taking opioid pain medications). Group sessions: to enhance peer support related to chronic pain. Each session included sharing refections on lessons learned and goals set during one-on-one sessions, and challenges encountered.	Two peers with HIV and chronic pain ("pain pals") and two research staff ("pain coaches"), working in pal-coach pairs. Participant. Six (in-person) one-to-one sessions (led by pain coach) and six group sessions (led by pal-coach pair), alternating weekly.

For peer review only

Miklavcic, 2020, Canada	Type 2 diabetes<br>Two or more other chronic conditions	Physical-Physical	132<br>70<br>62	6 months. Baseline, 6 months.	Physical functioning<br>6 months<br>No	Collaborative care	High	Aging, Community and Health Research Unit Community Partnership Program.<br>Focused on the role of self-efficacy in achieving self management behaviours, and tailored to patient preferences and needs.The program included up to 3 home visits by RN or RD; 6 monthly group sessions which included education, exercise, a light meal, and peer support; monthly intervention team case conferences (share observations about participants strengths, challenges and goals, identify needs related to other community-based service, and prepare for upcoming group sessions); care coordination to link the client to relevant health or social services.	Interprofessional team consisting of a Registered Nurse (RN), Registered Dietitian (RD), and Program Coordinator (PC).<br>Patient (family caregiver was also invited to attend).<br>One-to-one sessions at patients home, group sessions at community site once a month.

For peer review only

Morgan, 2013, Australia	Depression Type 2 diabetes/ coronary heart disease (or both)	Physical-Mental	400 206 194	6 months. Baseline, 6 months, 12 months	Depression (PHQ-9) 6 months Yes	Cognitive and/or Behavioural Activation	Some concerns	TrueBlue Collaborative care approach. Involved visit with PN followed by usual GP, in which stepped care (psychotherapy or pharmacotherapy) was offered if PHQ-9 scores had not improved or dropped below 5. The PN obtained physical measures and reviewed recent pathology results and lifestyle risk factors. They worked with the patient to identify barriers to achieving goals and discuss ways to overcome them. They assisted the patient with self-management by discussing available educational resources and setting personal goals for review at the next visit.	Practice Nurse (PN) Patient One-to-one. Patient visited their practice every 3 months for 45 minutes (then 15 minutes with the GP).

For peer review only

Naik, 2019, USA	Diabetes Depression	Physical-Mental	225<br>136<br>89	6 months<br>Baseline, 6 months, 12 months.	Depression (PHQ-9) and clinical (HbA1c)<br>6 and 12 months<br>No	Self-management	Low	Healthy Outcomes through Patient Empowerment (HOPE).<br>The first 2 sessions focused on building rapport, introducing and clarifying values, collaboratively setting initial goals, identifying potential skill sets to address goals, and empowering patients to advocate for their health through active communication with clinicians. Sessions 3-6 focused on discrete skill modules (increasing pleasant activities, using thoughts to improve wellness, diet, physical activity, medication management, and relaxation) customized to meet their goals. Sessions 7-9 focused on maintenance skills (reviewing action plans and overcoming barriers).	Trained health professional.<br>Participant.<br>One-to-one.<br>9 telephone sessions: biweekly (30-40 minutes) in months 1-3, and monthly (15 minutes) from months 4-6). No contact in following 6 months.

For peer review only

Ose, 2019, USA	Type 2 diabetes<br>Two or more other chronic conditions including:<br>Artherosclerosis<br>Coronary heart disease<br>Chronic obstructive lung disease<br>Asthma<br>Cerebrovascular diseases<br>Depression<br>Heart failure<br>Parkinsons<br>Chronic pain	Physical-Physical	495<br>252<br>243	9 months<br>Baseline, 9 months.	Self-care (Summary of Diabetes Self-Care Activities Measure (SDSCA-G)<br>9 months<br>No	Self-management	Low	Care management intervention. 3 steps: (1) Structured, IT-supported assessment performed by net-care managers to capture medical/non-medical needs; (2) goal setting between patient and primary care physician based on results of assessment; (3) telephone monitoring by net-care managers to detect acute clinical needs and provide support. Embedded in PCN (net-care manager links to responsible physician via a medical assistant).	Net Care Manager.<br>Patient.<br>One-to-one.<br>(1) at-home visits/face-to-face meetings 3 times within initial 2 weeks, then again at 6 months; (2) once at week 2; (3) every 2 weeks for the first 6 months then every month for the next 3 months.

O'Toole, 2021, Ireland	Conditions not listed. Mean 4.5 per participant	Physical-Physical	149<br>78<br>71	6 weeks. Baseline, 6 weeks, 6 months.	Quality of life (EQ-5D-3L) and behaviour change (participation)<br>6 weeks<br>No	Self-management	Some concerns - a lot of analyses were completed	Occupational therapy-led self-management support programme (OPTIMAL).<br>Intervention incorporated performance accomplishments, vicarious learning, social/verbal persuasion, reinterpretation of physiological and emotional states. Patients' programme goals were set in week 1 and reviewed weekly. They were also given educational and health-promoting resources/materials. Week 1: Introduction to self-management, activity and health, and goal-setting. Week 2: Fatigue management and healthy eating. Week 3: Maintaining physical activity. Week 4: Maintaining mental wellbeing. Week 5: Managing medications. Week 6: Communication and programme review.	Occupational therapist.<br>Patient.<br>Group.<br>2.5 hour face-to-face session once a week for 6 weeks, in a primary care centre or community resource centre.

For peer review only

Park, 2014,<br>South Korea	Two or more of:<br>Stroke<br>Parkinsons<br>Dementia	Physical-<br>Physical	43<br>21<br>22	8 weeks.<br>Baseline, 8 weeks	self-management behaviors, self-efficacy, and health status<br>8 weeks<br>Yes	Cognitive and/or Behavioural Activation	Low	Health coaching self management programme (HCSMP).<br>Group level approach: structured health education developed for older adults, regarding participants' diseases and self-management strategies, then a period of physical activity to enhance cognition and body movements.<br>Individual level approach: individual counselling and goal setting for self-management behaviours.<br>Facility-level approach: principal investigator met frequently with the director and chief manager of the nursing home to help support and facilitate patient progress.	Trained research team members who were geriatric nurse speciaists.<br>Participant.<br>Group sessions delivered face to face at nursing home for 8 weeks; education (Monday) and exercise (Thursday), both lasting 1 hour.<br>Individual sessions: 8 face-to-face coaching sessions for approx 20 minutes,

For peer review only

Pibernik-Okanovic, 2009, Croatia	Diabetes Depression	Physical-Mental	50<br>25<br>25	5 weeks<br>Baseline, 6 months, 12 months	Depression (CES-D) and clinical (HbA1c)<br>6 and 12 months<br>No	Self-management	Low	Psycho-education.<br>Four interactive meetings on the following topics: (1) symptoms of depression, interaction of depression and diabetes; (2) alleviaing burden of depression through activities and problem solving; (3) associations between depression and cognitive processes – thoughts, beliefs and attitudes that induce and maintain depression; (4) developing a personal plan for managing depression-related problems in the future. Patients were provided with a self-help manual and kept daily mood and activities diaries.	Not specified.<br>Patient.<br>Group.<br>Four 90 minute sessions - first two held within the first week and the third and fourth held at two week intervals

For peer review only

Rose, 2018, Canada	COPD and two or more of: Cardiovascular disease Depression Diabetes Osteopenia and osteoporosis Gastro-oesophageal reflux disease Hypothyroidism Osteoarthritis Glaucoma and cataracts Cachexia and malnutrition Chrornic kidney disease Anxiety Peripheral muscle dysfunction Obstructive sleep apnoea Lung cancer Cerebrovascular accident	Physical-Physical	470 236 234		12 months. Baseline, 3 months, 6 months, and 12 months.	Hospital admissions 12 months No	Cognitive and/or Behavioural Activation	Low	Multi-component, case manager led intervention. Components included: (1) standardised 40 minute COPD education session on enrolment; (2) individualised care and action plans for COPD exacerbation recognition, self-management and management of comorbidites; (3) telephone consultations containing standardised reinforcement/ motivational interviewing focusing on health behaviours, action plan teach-back sessions, symptom monitoring, and problem solving strategies; (4) ongoing case manager communication with family physicians and with hospital specialists including respirologists; and 5) priority access to ambulatory outpatient clinics.	Case manager. Patient. One-to-one. 12 weekly telephone calls (after initial education session at ED/hospital/outpatient clinic), followed by monthly calls for 9 months.

For peer review only

Ross, 2005, Canada	Asthma<br>Panic disorder	Physical-Mental	48 (25 analysed)<br>25 (15 analsyed)<br>23 (10 analysed)	8 weeks.<br>Intervention group: Baseline, 8 weeks, and 6 month follow-up.<br>Wait-list control group: baseline (coincided with IG pre), 8 weeks (coincided with IG post), 16 weeks, and 6 month follow-up	Panic disorder and asthma outcomes<br>8 weeks<br>Yes	Cognitive and/or Behavioural Activation	Some Concerns - multiple scales used to measure the same outcome	CBT-AE.<br>CBT: (a) education about anxiety and panic; (b) cognitive therapy techniques aimed regarding anxiety and panic; (c) training in slow diaphragmatic breathing to aid physical symptoms of panic attacks; and (d) interoceptive exposure exercises designed to reduce participants' fear of anxiety/panic attack symptons.<br>AE: (a) information about airway inflammation and bronchospasm; (b) rationale, correct use, and side effects of medications, (c) inhaler techniques; (d) methods of self-monitoring, symptoms, and peak flow; (e) asthma triggers and strategies for control; and (f) action plans. Asthma diaries kept by the participants were reviewed during each treatment session to reinforce the importance of the participant's role in self-management and self monitoring.	Two nurse clinicians (one Asthma Educator, one with postdoctoral training in cognitive therapy).<br>Participant.<br>Small groups.<br>12 face-to-face 90 minute sessions with both CBT and AE components. Sessions 1-8 conducted twice weekly, and sessions 9-12 spaced one week apart.
Sajatovic, 2017, USA	Serious mental illlness<br>Type 2 diabetes	Physical-Mental	200<br>100<br>100	60 weeks.<br>Baseline, 13 weeks, 30 weeks, 60 weeks	Psychiatric symptoms<br>60 weeks<br>yes	Self-management	Some Concerns	Targeted Training in Illness Management.<br>Group-based psychosocial treatment that blends psychoeducation, problem-identification, goal-setting, behavioral modeling, and care linkage	Nurse and peer educators.<br>Participant.<br>Group/one-to-one.<br>12 weekly face-to-face sessions, followed by 15 minute one-to-one telephone calls every 2-4 weeks.

For peer review only

Salisbury, 2018, UK	Cardiovascular disease or chronic kidney disease<br>Stroke or transient ischaemic attack<br>Diabetes<br>COPD or asthma<br>Epilepsy<br>Atrial fibrillation<br>Serious mental illness<br>Depression<br>Dementia<br>Learning disability<br>Rheumatoid arthritis	Physical-Physical	33 practices randomised (17 to intervention, 16 to control).<br><br>1546<br>797<br>749	6 months. Baseline, 9 months, 15 months	Quality of life (EQ-5D-3L)<br>15 months<br>No	Collaborative care	Some concerns - self-completed measure for primary outcome and all were aware of group allocation	3D intervention<br>Replace separate disease focused review with one 6-monthly comprehensive multidisciplinary review. Done in 3 stages: (1) nurse review - identifies important health problems and how these affect life, and disease-specific care that the patient needs; (2) pharmacist reviews medication and makes recommendations for treatment; and (3) physician review - considers nurse and pharmacist reviews, then agrees on collaborative health plan with patient (which is printed off) specifying goals and how these will be achieved over the next 6 months of consultations.	Nurse, physician and pharmacist.<br>Patient.<br>One-to-one.<br>Two 6-monthly reviews consisting of two appointments (nurse and physician) and a medication review, then consultations over 6 months as per the health plan.

For peer review only

Schneider, 2015, USA	Type 2 diabetes Depression	Physical-Mental	29<br>15<br>14	6 months. Baseline, 3 months, 6 months.	Clinical outcomes (HbA1c) and depression (BDI-II)<br>3 and 6 months<br>No	Cognitive and/or Behavioural Activation	Low	Behavioural Activation and Exercise Condition.<br>Classes contained 3 parts - (i) warm-up, (ii) exercise (different types for first 12 weeks then based on paricipant enjoyment for next 12, gradually increasing in intensity each week), and (iii) cool down and BA (activity and mood monitoring, exploration of ways to make exercise more enjoyable, a behavioural contract with friends/ family to promote exercise, a list of life values and how to connect exercise to them, and exercise counseling strategies including self-monitoring activity using a pedometer, monitoring exercise intensity via perceived exertion and heart rate, identifying facilitators to exercise and engaging in problem solving for exercise barriers).	Female group leader.<br>Participant.<br>Group.<br>One orientation session and 38 classes over 4 weeks at a medical school site. The 90-minute classes occurred 2 times per week for 16 weeks, once a week for 4 weeks, and then once every other week for 4 weeks.

For peer review only

Takahashi, 2012, USA	Myocardial infarction<br>Congestive heart failure<br>COPD<br>Diabetes<br>Renal disease	Physical-Physical	205<br>102<br>103	12 months<br>Baseline, 12 months	Hospital admissions<br>12 months<br>No	Self-management	Some concerns - outcome assessors were not blind at follow-up, more deaths in intervention group	Telemonitoring.<br>An FDA approved telemonitoring device was used in the patients home. Patients performed daily assessment of symptoms and biometrics, and data were downloaded and reviewed daily by the health care team. A registered nurse communicated with the individuals via phone or videoconference if alerts arose, communicating with their primary physician about treatment options if needed.	Registered nurse and (if required) primary physician oversaw the process.<br>Patient.<br>One-to-one.<br>Daily 5-10 minute sessions<br>Home or clinical

For peer review only

Vera, 2010, USA	Depression<br>And one or more of:<br>Diabetes<br>Hypothryoidism<br>Asthma<br>Hypertension<br>Chronic bronchitis<br>Arthritis<br>Heart disease<br>High cholesterol<br>Stroke	Physical-Mental	179<br>89<br>90	6 months. Baseline, 8, 16 and 24 weeks.	Depression (SCL-20) and Functioning (SF-36)<br>6 months<br>Yes	Collaborative care	Low	Collaborative care.<br>A multicomponent intervention, including program oversight and teamwork among physicians, mental health specialists, and care managers. Patients were provided education about depression and a choice of 13 CBT sessions or antidepressant medication as initial treatment. The care manager participated in the coordination of treatment initiation, as well as monitoring treatment adherence, side effects, and clinical response, while liaising with the psychiatrist and patients physician. Extra contacts were available to help patients overcome barriers and provide treatment adherence support.	Care manager.<br>Patient<br>One-to-one.<br>Patients were contacted by telephone or in person every 2 weeks initially, and then monthly up to 6 months.

For peer review only

Wakefield, 2011, USA	Type 2 diabetes Hypertension	Physical-Physical	302<br>102 (low intensity) / 93 (high intensity)<br>107	6 months. Baseline, 6 months, 12 months	Clinical (HbA1c and SBP)<br>6 month<br>Yes	Self-management	Low	Telehealth intervention.<br>In a home telehealth device, all intervention participants logged blood pressure, blood glucose and responses to standardised questions (which were reinforced when answered correctly and reviewed/explained when answered incorrectly), which were reviewed daily by a registered nurse to determine whether the participant needed a follow-up (e.g. if clinical parameters were out of range or the participant needed extra support). In the high intensity group, a disease management algorithm was used, with scheduled prompts for rotating questions and education focused on diet, exercise, smoking cessation, foot care, advice for sick days, medications, weight management, preventive care, and behavior modification and lifestyle adjustments. In the low intensity group, participants responded to a smaller subset of questions from the HI group and did not use the algorithm.	Nurse.<br>Participant.<br>One-to-one.<br>Telehealth device was in patient home - follow-ups were made via telephone, letter or directly through the device as and when needed.

For peer review only

Williams, 2012, Australia	Diabetes<br>Chronic kidney disease/diabetic kidney disease<br>Hypertension	Physical-Physical	80<br>39<br>41	3 months.<br>Baseline, 3 months, 6 months, 9 months.	Medication adherence<br>3-6-9 months<br>No	Self-management	Low	Medication Self-Management Intervention (MESMI).<br>The intervention consisted of self-monitoring of blood pressure (participants were taught how to do this and recorded it daily for 3 months), an individualized medication review, a 20-minute DVD (using an interactive, psychosocial approach to motivate people to take their medications), and motivational interviewing to support blood pressure control and optimal medication self-management.	Intervention nurse.<br>Participant.<br>One-to-one.<br>Motivational interviewing was done fortnightly via telephone calls, starting 2 weeks after the intervention home visit until 3 months.
Wilson, 2018, USA	Depression<br>Any other chronic disease (most common: chronic pain, multiple sclerosis, cardiovascular disease, mental disorder)	Physical-Mental	53<br>27<br>26	8 weeks.<br>Baseline, 4 weeks, 8 weeks.	Depression (PHQ-8)<br>8 weeks<br>Yes	Cognitive and/or Behavioural Activation	Some concerns - as unblinded participants completing assessments, including primary outcome	Think Clearly About Depression.<br>Computer based intervention to teach mildly/moderately depressed individuals about the role of thinking in the development and maintenance of depression, using videos, interactive activities and homework exercises to challenge depressive thoughts and practice hopeful and constructive thinking, as well as a daily mood tracker and a schedule for mood boosting self-management activities. To increase engagement, the researchers created a program guide to assist participants and sent weekly prompts for the first 4 weeks.	Research team (two psychiatric nurse practicioners and a psychiatrist).<br>Participant.<br>One-to-one.<br>The program could be accessed at any time of day in any setting of the participants' choosing where they had Internet services and a personal computer or smartphone.

For peer review only

Yu, 2020, Canada	Diabetes AND two of:<br>Heart disease<br>Stroke<br>Hypertension<br>Cancer<br>Chronic lung disease<br>Arthritis<br>Inflammatory bowel disorder<br>Urinary incontinence	Physical-Physical	10 primary care practices were randomsed (5 in each arm).<br>213<br>102<br>111	6 months<br>Baseline, 6 months, 12 months.	Behaviour change (Decisional conflict)<br>12 months<br>No	Self-management	Some concerns - as unblinded participants completing assessments, including primary outcome	MyDiabetesPlan.<br>Web-based intervention where participants provide cardiometabolic and psychosocial profiles and general care priorities. An individualised action plan is then generated with diabetes-specific goals and strategies. For the first 6 months, MyDiabetesPlan was completed by a member of the clinical team with the patient, then their action plan was reviewed. At the 6-month mark patients were given a "how to" guide for MyDiabetesPlan and from then on logged the information themselves before their appointment.	Clinician (nurse, dietitian, physician, or pharmacist).<br>Patient.<br>One-to-one.<br>Clinical encounters were done face-to-face at the participants primary care clinic.

For peer review only

Study	Outcome	Effective	Intervention type	Intervention length	M-LTCs combination
Blank, 2011	Clinical endpoint	No	Self-management	>6months	Physical-Mental
Chwastiak, 2018	Clinical endpoint	No	Collaborative care	3-6months	Physical-Mental
Ell, 2010	Psychological distress (depression)	Yes	Collaborative care	>6months	Physical-Mental
Fisher, 2020	Functioning	No	Self-management	3-6months	Physical-Physical
Guo, 2020	N/A	N/A	Collaborative care	>6months	Physical-Physical
Hernandez, 2021	N/A	N/A	Self-management	3-6months	Physical-Physical
Kamradt, 2019	Quality of life	No	Collaborative care	>6months	Physical-Physical
Katon, 2004	Psychological distress (depression)	Yes	Collaborative care	>6months	Physical-Mental
Khunti, 2021	Behaviour change	Yes	Self-management	>6months	Physical-Physical
Koesoemadinata, 2021	N/A	N/A	Cognitive and/or Behavioural activation	3-6months	Physical-Physical
Lear, 2021	N/A	N/A	Self-management	>6months	Physical-Physical
Lenferink, 2019	Clinical endpoint	No	Self-management	>6months	Physical-Physical
Merlin, 2018	Pain	No	Self-management	<3months	Physical-Physical
Ose, 2019	N/A	N/A	Self-management	>6months	Physical-Physical
O'Toole, 2021	Quality of life<br>Behaviour change	No<br>No	Self-management	<3months	Physical-Physical
Pibernik, 2009	Psychological distress (depression)<br>Clinical endpoint	No<br>No	Self-management	<3months	Physical-Mental
Rose, 2018	N/A	N/A	Cognitive and/or Behavioural activation	>6months	Physical-Physical
Takahashi, 2012	N/A	N/A	Self-management	>6months	Physical-Physical
Taylor, 2003	Clinical endpoint	Yes	Collaborative care	>6months	Physical-Physical
Vera 2010	Psychological disress (depression)<br>Functioning	Yes<br>Yes	Collaborative care	3-6months	Physical-Mental
Wakefield, 2011	Clinical endpoint	Yes	Self-management	3-6months	Physical-Physical
Williams, 2012	Behaviour change	No	Self-management	3-6months	Physical-Physical

For peer review only

Supplementary Table 4. Pooled effects of outcomes, stratified by types of long-term condition combination types.

	Physical-Physical			Physical-Mental		
**Post-intervention**	*n*	SMD [95% CI]	I^2	*n*	SMD [95% CI]	I^2
Quality of life	3	0.16 [-0.06, 0.39]	65%	5	0.10 [-0.22, 0.41]	65%
Clinical endpoints	3	-0.29 [-0.61, 0.02]	61%	8	-0.20 [-0.53, 0.12]	90%
Behaviour change	2	N/A	N/A	4	-0.14 [-0.32, 0.05]	14%
Functioning	5	0.03 [-0.15, 0.20]	41%	8	0.04 [-0.09, 0.17]	45%
Psychological distress (depression)	6	-0.11 [-0.34, 0.12]	57%	15	**-0.58 [-0.94, -0.21]**	95%
Psychological distress (anxiety)	4	-0.10 [-0.19, 0.00]	0%	5	-0.17 [-0.48, 0.13]	63%
Emotional wellbeing	1	N/A	N/A	6	0.30 [-0.01, 0.61]	87%
**Maintenance**						
Functioning	1	N/A	N/A	6	0.03 [-0.07, 0.13]	0%
Psychological distress (depression)	1	N/A	N/A	7	0.05 [-0.42, 0.51]	94%

For peer review only

Database Search Strategy for Systematic Review (MEDLINE, Embase, PsychInfo, CINAHL)

Multiple long-term conditions search strategy

Adapted from Smith et al. (2021)

1 Comorbidity/

2 (comorbid$ or co-morbid$).ti,ab.

3 (multimorbid$ or multi-morbid$).ti,ab.

4 (multidisease? or multi-disease? or (multiple adj (ill$ or disease? or condition? or syndrom$ or disorder?))).ti,ab.

5 Long term condition.ti,ab.

6 Chronic disease/

7 (multiple chronic$ adj3 (disease? or ill$ or care or condition? or disorder$ or health$ or syndrom$ or symptom$)).ti,ab.

8 ((coocur$ or co-ocur$ or coexist$ or co-exist$ or multipl$) adj3 (disease? or ill$ or care or condition? or disorder$ or health$ or medication$ or symptom$ or syndrom$)).ti,ab.

9 or/1-8

10 exp diabetes mellitus/ or diabet$.ti,ab.

11 exp hypertension/ or (hypertens$ or "high blood pressure?").ti,ab.

12 exp heart diseases/ or (((heart or cardiac or cardiovascular or coronary) adj (disease? or disorder? or failure)) or arrythmia?).ti,ab.

13 exp cerebrovascular disorders/ or ((cerebrovascular or vascular or carotoid$ or arter$) adj (disorder? or disease?)).ti,ab.

14 exp asthma/ or asthma$.ti,ab.

15 exp pulmonary disease chronic obstructive/ or (copd or (pulmonary adj2 (disease? or disorder?))).ti,ab.

16 exp hyperlipidemia/ or (hyperlipidem$ or Hypercholesterolemia$ or hypertriglyceridemia$).ti,ab.

17 exp Thyroid diseases/ or ((thyroid adj (disease? or disorder)) or hyperthyroid$ or hypothyroid$).ti,ab.

18 exp arthritis rheumatoid/ or rheumatoid arthritis.ti,ab.

19 exp mental disorders/ or (((mental or anxiety or panic or mood or psychological) adj (disease? or disorder?)) or (depression or bipolar or schizophren$ or psychos$)).ti,ab.

20 exp epilepsy/ or (epileps$ or seizure?).ti,ab.

21 exp hiv infections/ or (HIV or acquired immune$ deficiency syndrome? or (aids adj (associated or related or arteritis))).ti,ab.

22 exp neoplasms/ or (neoplasm? or cancer?).ti,ab.

For peer review only

23 exp kidney diseases/ or (kidney adj (disease? or disorder?)).ti,ab.

24 exp liver diseases/ or (liver adj (disease? or disorder?)).ti,ab.

25 exp osteoporosis/ or osteoporosis.ti,ab.

26 exp dementia/ or dementia.ab,ti.

27 exp stroke/ or stroke.ab,ti.

28 exp multiple sclerosis/ or (multiple sclerosis).ab,ti.

29 exp Parkinson disease/ or Parkinson*.ab,ti.

30 exp motor neuron disease/ or (motor neuron disease).ab,ti.

31 exp neurodegenerative diseases/ or neurodegenerative.ab,ti.

32 or/10-31

33 9 and 32

Behaviour change intervention search strategy

Adapted from Gardener et al. (2017)

1 (self car*) OR (self-manag*).mp.

2 behavio* adj3 chang*.mp.

3 (health education) OR (structure* education).mp.

4 secondary prevent*.mp.

5 (case manag*) OR (care manag*).mp.

6 (care navigat*) OR (community navigat*) OR (care co-ordinat*).mp.

7 (shared care) OR (collaborative care).mp.

8 (complex intervention*) OR (multicomponent intervention) OR (multiple intervention component).mp.

9 (integrated adj (model OR care)).mp.

10 ((Multiprofessional AND intervention$) OR (Interprofessional AND intervention$)).mp.

11 (comprehensive care) OR (comprehensive health care).mp.

12 (prevent* adj3 hospital*).mp.

13 or/1-12

RCT search strategy

Cochrane RCT from Smith et al. (2021)

1 randomi?ed controlled trial.pt.

2 controlled clinical trial.pt.

For peer review only

3 random$.ti,ab.

4 (control$ adj2 (trial? or study or studies)).ti,ab.

5 double-blind method/ or random allocation/ or single-blind method/

6 ((double or single or triple or treble) adj2 blind$).ti,ab.

7 or/1-6

8 exp animals/ not humans.sh.

9 7 not 8

Web of Science Search Strategy for Systematic Review

1 comorbid* or co-morbid* or multimorbid* or multi-morbid* or multidisease? or multi-disease? or (multiple NEAR/1 (illness* or disease* or condition* or syndrome* or disorder*)) or "Long term condition*" or "chronic disease" or ("multiple chronic*" NEAR/3 (disease? or illness* or care or condition? or disorder* or health* or syndrome* or symptom*)) or ((coocur* or co-occur* or coexist* or co-exist* or multipl*) NEAR/3 (disease? or illness* or care or condition? or disorder* or health* or medication* or symptom* or syndrome*))

2 diabe* or hypertens* or "high blood pressure" or (((heart or cardiac or cardiovascular or coronary) NEAR/3 (disease? or disorder? or failure)) or arrythmia?) or ((cerebrovascular or vascular or carotoid* or arter*) NEAR/3 (disorder? or disease?)) or asthma* or copd or (pulmonary NEAR/2 (disease? or disorder?)) or hyperlipidem* or Hypercholesterolemia* or hypertriglyceridemia* or (thyroid NEAR/1 (disease? or disorder)) or hyperthyroid* or hypothyroid* or "rheumatoid arthritis" or (((mental or anxiety or panic or mood or psychological) NEAR/1 (disease? or disorder?)) or (depression or bipolar or schizophren* or psychos*)) or epilepsy* or seizure? or HIV or "acquired immune* deficiency syndrome?" or (aids NEAR/1 (associated or related or arteritis)) or neoplasm? or cancer? or (kidney NEAR/1 (disease? or disorder?)) or (liver NEAR/1 (disease? or disorder?)) or osteoporosis or dementia or stroke or "multiple sclerosis" or Parkinson* or "motor neuron disease" or neurodegenerative

3 1 AND 2

4 "self car*" OR self-manag* or behavio* NEAR/3 chang* or "health education" OR "structure* education" or "secondary prevent*" or "case manag*" OR "care manag*" or "care navigat*" OR "community navigat*" OR "care co-ordinat*" or "shared care" OR "collaborative care" or "complex intervention*" OR "multicomponent intervention" OR "multiple intervention component" or (integrated NEAR/1 (model OR care)) or ((Multiprofessional AND intervention*) OR (Interprofessional AND intervention*)) or "comprehensive care" OR "comprehensive health care" or (prevent* NEAR/3 hospital*)

5 ((randomised OR randomized OR randomisation OR randomisation OR (random* AND (allocat* OR assign*)) OR (blind* AND (single OR double OR treble OR triple))))

6 ((animal* AND model* OR mouse OR mice))

7 5 NOT 6

#3 AND #4 AND #7

For peer review only

1. Ali MK, Chwastiak L, Poongothai S, Emmert-Fees KMF, Patel SA, Anjana RM, et al. Effect of a Collaborative Care Model on Depressive Symptoms and Glycated Hemoglobin, Blood Pressure, and Serum Cholesterol among Patients with Depression and Diabetes in India: The INDEPENDENT Randomized Clinical Trial. JAMA - Journal of the American Medical Association. 2020 Aug 18;324(7):651–62.

2. Aragonès E, Rambla C, López-Cortacans G, Tomé-Pires C, Sánchez-Rodríguez E, Caballero A, et al. Effectiveness of a collaborative care intervention for managing major depression and chronic musculoskeletal pain in primary care: A cluster-randomised controlled trial. J Affect Disord. 2019 Jun 1;252:221–9.

3. Barley EA, Walters P, Haddad M, Phillips R, Achilla E, McCrone P, et al. The UPBEAT nurse-delivered personalized care intervention for people with coronary heart disease who report current chest pain and depression: A randomised controlled pilot study. PLoS One. 2014 Jun 5;9(6).

4. Barrowclough C, Haddock G, Tarrier N, Shôn Lewis FbpW, Jan Moring Frcp, Nichola Schofield B, et al. Article Randomized Controlled Trial of Motivational Interviewing, Cognitive Behavior Therapy, and Family Intervention for Patients With Comorbid Schizophrenia and Substance Use Disorders. Vol. 158, Am J Psychiatry. 2001.

5. Barr Taylor C, Houston Miller N, Reilly KR, Greenwald G, Cunning D, Deeter A, et al. Evaluation of a Nurse-Care Management System to Improve Outcomes in Patients With Complicated Diabetes [Internet]. 2003. Available from: http://diabetesjournals.org/care/article-pdf/26/4/1058/659413/dc0403001058.pdf

6. Battersby MW, Beattie J, Pols RG, Smith DP, Condon J, Blunden S. A randomised controlled trial of the Flinders ProgramTM of chronic condition management in Vietnam veterans with co-morbid alcohol misuse, and psychiatric and medical conditions. Australian and New Zealand Journal of Psychiatry. 2013 May;47(5):451–62.

7. Blank MB, Hanrahan NP, Fishbein M, Wu ES, Tennille JA, Ten Have TR, et al. A randomized trial of a nursing intervention for HIV disease management among persons with serious mental illness. Psychiatric Services. 2011 Nov 1;62(11):1318–24.

8. Boeschoten RE, Dekker J, Uitdehaag BMJ, Beekman ATF, Hoogendoorn AW, Collette EH, et al. Internet-based treatment for depression in multiple sclerosis: A randomized controlled trial. Multiple Sclerosis. 2017 Jul 1;23(8):1112–22.

9. Bogner HR, De Vries HF. Integration of depression and hypertension treatment: A pilot, randomized controlled trial. Ann Fam Med. 2008;6(4):295–301.

10. Chwastiak LA, Luongo M, Russo J, Johnson L, Lowe JM, Hoffman G, et al. Use of a mental health center collaborative care team to improve diabetes care and outcomes for patients with psychosis. Psychiatric Services. 2018 Mar 1;69(3):349–52.

11. Cummings DM, Lutes LD, Littlewood K, Solar C, Carraway M, Kirian K, et al. Randomized trial of a tailored cognitive behavioral intervention in type 2 diabetes with comorbid depressive and/or regimen-related distress symptoms: 12-month outcomes from. In: Diabetes Care. American Diabetes Association Inc.; 2019. p. 841–8.

12. Druss BG, Singh M, Von Esenwein SA, Glick GE, Tapscott S, Tucker SJ, et al. Peer-led self-management of general medical conditions for patients with serious mental illnesses: A randomized trial. Psychiatric Services. 2018 May 1;69(5):529–35.

For peer review only

13. Ell K, Katon W, Xie B, Lee PJ, Kapetanovic S, Guterman J, et al. Collaborative care management of major depression among low-income, predominantly hispanic subjects with diabetes: A randomized controlled trial. Diabetes Care. 2010 Apr;33(4):706–13.

14. Ell K, Aranda MP, Wu S, Oh H, Lee PJ, Guterman J. Promotora assisted depression and self-care management among predominantly Latinos with concurrent chronic illness: Safety net care system clinical trial results. Contemp Clin Trials. 2017 Oct 1;61:1–9.

15. Fisher K, Markle-Reid M, Ploeg J, Bartholomew A, Griffith LE, Gafni A, et al. Self-management program versus usual care for community-dwelling older adults with multimorbidity: A pragmatic randomized controlled trial in Ontario, Canada. J Comorb. 2020 Jan 1;10:2235042X2096339.

16. Garvey J, Connolly D, Boland F, Smith SM. OPTIMAL, an occupational therapy led self-management support programme for people with multimorbidity in primary care: A randomized controlled trial. BMC Fam Pract. 2015 Dec 12;16(1).

17. Goldberg RW, Dickerson F, Lucksted A, Brown CH, Weber E, Tenhula WN, et al. Living Well: An intervention to improve self-management of medical illness for individuals with serious mental illness. Psychiatric Services. 2013 Jan 1;64(1):51–7.

18. Goorden M, van der Feltz-Cornelis CM, van Steenbergen-Weijenburg KM, Horn EK, Beekman ATF, Hakkaar-Van Roijen L. Cost-utility of collaborative care for the treatment of comorbid major depressive disorder in outpatients with chronic physical conditions. A randomized controlled trial in the general hospital setting (CC-DIM). Neuropsychiatr Dis Treat. 2017 Jul 18;13:1881–93.

19. Griva K, Rajeswari M, Nandakumar M, Khoo EYH, Lee VYW, Chua CG, et al. The combined diabetes and renal control trial (C-DIRECT) - A feasibility randomised controlled trial to evaluate outcomes in multi-morbid patients with diabetes and on dialysis using a mixed methods approach. BMC Nephrol. 2019 Jan 3;20(1).

20. Guo Y, Lane DA, Wang L, Zhang H, Wang H, Zhang W, et al. Mobile Health Technology to Improve Care for Patients With Atrial Fibrillation. J Am Coll Cardiol. 2020 Apr 7;75(13):1523–34.

21. Hernandez-Quiles C, Bernabeu-Wittel M, Barón-Franco B, Palacios AA, Garcia-Serrano MR, Lopez-Jimeno W, et al. A randomized clinical trial of home telemonitoring in patients with advanced heart and lung diseases. J Telemed Telecare. 2021;

22. House A, Bryant L, Russell AM, Wright-Hughes A, Graham L, Walwyn R, et al. Randomized controlled feasibility trial of supported self-management in adults with Type 2 diabetes mellitus and an intellectual disability: OK Diabetes. Diabetic Medicine. 2018 Jun 1;35(6):776–88.

23. Jackson IL, Ukwe C V. Clinical outcomes of pharmaceutical care intervention in HIV positive patients with hypertension: A randomized controlled study. J Clin Pharm Ther. 2021 Aug 1;46(4):1083–94.

24. Kamradt M, Ose D, Krisam J, Jacke C, Salize HJ, Besier W, et al. Meeting the needs of multimorbid patients with Type 2 diabetes mellitus – A randomized controlled trial to assess the impact of a care management intervention aiming to improve self-care. In: Diabetes Research and Clinical Practice. Elsevier Ireland Ltd; 2019. p. 184–93.

25. Kanwal F, Pyne JM, Tavakoli-Tabasi S, Nicholson S, Dieckgraefe B, Storay E, et al. A Randomized Trial of Off-Site Collaborative Care for Depression in Chronic Hepatitis C Virus. Health Serv Res. 2018 Aug 1;53(4):2547–66.

For peer review only

26. Katon WJ, Michael ;, Korff V, Lin EHB, Simon G, Ludman E, et al. The Pathways Study A Randomized Trial of Collaborative Care in Patients With Diabetes and Depression. 2004.

27. Katon WJ, Lin EH, Von Korff M, Ciechanowski P, Ludman EJ, Young B, et al. Collaborative Care for Patients with Depression and Chronic Illnesses From the Departments of Psychiatry A bs t r ac t. Vol. 363, N Engl J Med. 2010.

28. Khunti K, Highton PJ, Waheed G, Dallosso H, Redman E, Batt ME, et al. Promoting physical activity with self-management support for those with multimorbidity: A randomised controlled trial. British Journal of General Practice. 2021 Dec 1;71(713):E921–30.

29. Koesoemadinata RC, McAllister SM, Soetedjo NNM, Santoso P, Ruslami R, Damayanti H, et al. Educational counselling of patients with combined TB and diabetes mellitus: a randomised trial. Public Health Action. 2021 Dec 18;11(4):202–8.

30. Kroenke K, Bair MJ, Damush TM, Wu J, Hoke S, Sutherland J, et al. Optimized Antidepressant Therapy and Pain Self-management in Primary Care Patients With Depression and Musculoskeletal Pain A Randomized Controlled Trial [Internet]. 2009. Available from: https://jamanetwork.com/

31. Lear SA, Norena M, Banner D, Whitehurst DGT, Gill S, Burns J, et al. Assessment of an Interactive Digital Health-Based Self-management Program to Reduce Hospitalizations among Patients with Multiple Chronic Diseases: A Randomized Clinical Trial. JAMA Netw Open. 2021 Dec 28;4(12).

32. Lenferink A, van der Palen J, van der Valk PDLPM, Cafarella P, van Veen A, Quinn S, et al. Exacerbation action plans for patients with COPD and comorbidities: A randomised controlled trial. European Respiratory Journal. 2019 Nov 1;54(5).

33. Markle-Reid M, Ploeg J, Fraser KD, Fisher KA, Bartholomew A, Griffith LE, et al. Community Program Improves Quality of Life and Self-Management in Older Adults with Diabetes Mellitus and Comorbidity. J Am Geriatr Soc. 2018 Feb 1;66(2):263–73.

34. McDermott RA, Schmidt B, Preece C, Owens V, Taylor S, Li M, et al. Community health workers improve diabetes care in remote Australian indigenous communities: Results of a pragmatic cluster randomized controlled trial. BMC Health Serv Res. 2015 Feb 19;15(1).

35. Merlin JS, Westfall AO, Long D, Davies S, Saag M, Demonte W, et al. A Randomized Pilot Trial of a Novel Behavioral Intervention for Chronic Pain Tailored to Individuals with HIV. AIDS Behav. 2018 Aug 1;22(8):2733–42.

36. Miklavcic JJ, Fraser KD, Ploeg J, Markle-Reid M, Fisher K, Gafni A, et al. Effectiveness of a community program for older adults with type 2 diabetes and multimorbidity: A pragmatic randomized controlled trial. BMC Geriatr. 2020 May 13;20(1).

37. Morgan MAJ, Coates MJ, Dunbar JA, Reddy P, Schlicht K, Fuller J. The TrueBlue model of collaborative care using practice nurses as case managers for depression alongside diabetes or heart disease: a randomised trial. BMJ Open [Internet]. 2013; Available from: http://bmjopen.bmj.com/

38. Naik AD, Hundt NE, Vaughan EM, Petersen NJ, Zeno D, Kunik ME, et al. Effect of Telephone-Delivered Collaborative Goal Setting and Behavioral Activation vs Enhanced Usual Care for Depression among Adults with Uncontrolled Diabetes: A Randomized Clinical Trial. JAMA Netw Open. 2019 Aug 7;2(8).

39. Ose D, Kamradt M, Kiel M, Freund T, Besier W, Mayer M, et al. Care management intervention to strengthen self-care of multimorbid patients with type 2 diabetes in a German primary care network: A randomized controlled trial. PLoS One. 2019 Jun 1;14(6).

40. O'Toole L, Connolly D, Boland F, Smith SM. Effect of the OPTIMAL programme on self-management of multimorbidity in primary care: A randomised controlled trial. British Journal of General Practice. 2021 Apr 1;71(705):E303–11.

41. Park YH, Chang HK. Effect of a health coaching self-management program for older adults with multimorbidity in nursing homes. Patient Prefer Adherence. 2014 Jul 4;8:959–70.

42. Pibernik-Okanovic M, Begic D, Ajdukovic D, Andrijasevic N, Metelko Z. Psychoeducation versus treatment as usual in diabetic patients with subthreshold depression: Preliminary results of a randomized controlled trial. Trials. 2009 Aug 26;10.

43. Rose L, Istanboulian L, Carriere L, Thomas A, Lee HB, Rezaie S, et al. Program of integrated care for patients with chronic obstructive pulmonary disease and multiple comorbidities (PIC COPD+): A randomised controlled trial. European Respiratory Journal. 2018;51(1).

44. Ross CJM, Davis TMA, MacDonald GF. Cognitive-behavioral treatment combined with asthma education for adults with asthma and coexisting panic disorder. Clin Nurs Res. 2005 May;14(2):131–57.

45. Sajatovic M, Gunzler DD, Kanuch SW, Cassidy KA, Tatsuoka C, McCormick R, et al. A 60-week prospective RCT of a self-management intervention for individuals with serious mental illness and diabetes mellitus. Psychiatric Services. 2017 Sep 1;68(9):883–90.

46. Salisbury C, Man MS, Bower P, Guthrie B, Chaplin K, Gaunt DM, et al. Management of multimorbidity using a patient-centred care model: a pragmatic cluster-randomised trial of the 3D approach. The Lancet. 2018 Jul 7;392(10141):41–50.

47. Schneider KL, Panza E, Handschin B, Ma Y, Busch AM, Waring ME, et al. Feasibility of Pairing Behavioral Activation With Exercise for Women With Type 2 Diabetes and Depression: The Get It Study Pilot Randomized Controlled Trial [Internet]. 2015. Available from: www.elsevier.com/locate/bt

48. Takahashi PY, Pecina JL, Upatising B, Chaudhry R, Shah ND, Van Houten H, et al. A randomized controlled trial of telemonitoring in older adults with multiple health issues to prevent hospitalizations and emergency department visits. Arch Intern Med. 2012 May 28;172(10):773–9.

49. Vera M, Perez-Pedrogo C, Huertas SE, Reyes-Rabanillo ML, Juarbe D, Huertas A, et al. Collaborative Care for Depressed Patients With Chronic Medical Conditions: A Randomized Trial in Puerto Rico. Psychiatric Services. 2010 Feb 1;61(2).

50. Wakefield BJ, Holman JE, Ray A, Scherubel M, Adams MR, Hillis SL, et al. Effectiveness of home telehealth in comorbid diabetes and hypertension: A randomized, controlled trial. Telemedicine and e-Health. 2011 May 1;17(4):254–61.

51. Williams A, Manias E, Walker R, Gorelik A. A multifactorial intervention to improve blood pressure control in co-existing diabetes and kidney disease: A feasibility randomized controlled trial. J Adv Nurs. 2012 Nov;68(11):2515–25.

52. Wilson M, Hewes C, Barbosa-Leiker C, Mason A, Wuestney KA, Shuen JA, et al. Engaging Adults With Chronic Disease in Online Depressive Symptom Self-Management. West J Nurs Res. 2018 Jun 1;40(6):834–53.

For peer review only

53. Yu C, Choi D, Bruno BA, Thorpe KE, Straus SE, Cantarutti P, et al. Impact of mydiabetesplan, a web-based patient decision aid on decisional conflict, diabetes distress, quality of life, and chronic illness care in patients with diabetes: Cluster randomized controlled trial. J Med Internet Res. 2020 Sep 29;22(9).

For peer review only

Supplementary figure 3

Table 2 significant effects forest plots

Psychological distress (depression) – post-intervention overall effect

Study or Subgroup	Intervention Mean	Intervention SD	Intervention Total	Control Mean	Control SD	Control Total	Weight	Std. Mean Difference IV, Random, 95% CI
Ali 2020	0.52	0.38	192	0.82	0.54	202	5.2%	-0.64 [-0.84, -0.44]
Aragones 2019	1.29	0.77	167	1.42	0.73	161	5.2%	-0.17 [-0.39, 0.04]
Barley 2014	10.3	3.8	35	9.2	4.6	39	4.7%	0.26 [-0.20, 0.71]
Boeschoten 2017	20.3	8.8	74	21	9.1	78	5.0%	-0.08 [-0.40, 0.24]
Bogner 2008	9.9	10.7	32	19.3	15.2	32	4.5%	-0.71 [-1.21, -0.20]
Cummings 2019	6.3	5.9	67	7.9	7	72	5.0%	-0.25 [-0.58, 0.09]
Ell 2017	1.02	0.07	178	1.15	0.07	170	5.1%	-1.85 [-2.10, -1.60]
Garvey 2015	6.32	4.19	22	7.82	3.83	22	4.3%	-0.37 [-0.96, 0.23]
House 2018	2.4	1.87	30	1.1	1.8	29	4.5%	0.70 [0.17, 1.23]
Katon 2010	0.83	0.68	105	1.14	0.66	106	5.1%	-0.46 [-0.73, -0.19]
Kroenke 2009	1.14	0.69	123	1.69	0.74	127	5.1%	-0.77 [-1.02, -0.51]
Markle-Reid 2018	4.67	4.83	71	5.71	5.24	65	5.0%	-0.21 [-0.54, 0.13]
Miklavcic 2020	4.94	4.46	63	5.82	4.93	50	4.9%	-0.19 [-0.56, 0.19]
Morgan 2013	7.1	0.8	164	9	0.9	146	5.1%	-2.23 [-2.52, -1.95]
Naik 2019	10.9	6.1	107	12.4	6	80	5.0%	-0.25 [-0.54, 0.04]
Park 2014	5.3	3.4	22	7.6	5.8	21	4.3%	-0.48 [-1.09, 0.13]
Ross 2005	11.8	9.93	15	16.78	7.89	9	3.6%	-0.52 [-1.36, 0.32]
Sajatovic 2017	15.92	10	100	18.55	8.8	100	5.1%	-0.28 [-0.56, 0.00]
Salisbury 2018	6.1	4.4	654	6.6	4.5	641	5.3%	-0.11 [-0.22, -0.00]
Schneider 2016	12.5	14.8	15	12.3	4.1	14	3.9%	0.02 [-0.71, 0.75]
Wilson 2018	17.46	6.16	22	20.5	5.3	25	4.3%	-0.52 [-1.11, 0.06]
**Total (95% CI)**			**2258**			**2189**	**100.0%**	**-0.45 [-0.73, -0.16]**

Heterogeneity: $Tau^2 = 0.38$; $Chi^2 = 366.51$, df = 20 ($P < 0.00001$); $I^2 = 95\%$
Test for overall effect: Z = 3.11 (P = 0.002)

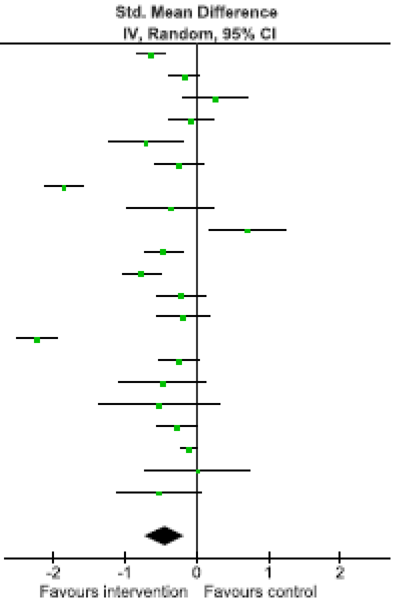

Psychological distress (anxiety) – post-intervention overall effect

Study or Subgroup	Intervention Mean	Intervention SD	Intervention Total	Control Mean	Control SD	Control Total	Weight	Std. Mean Difference IV, Random, 95% CI
Barley 2014	11	5	35	10.4	5	39	7.5%	0.12 [-0.34, 0.58]
Battersby 2013	48.95	10.39	44	47	13.75	28	7.1%	0.16 [-0.31, 0.64]
Boeschoten 2017	9	3.8	74	9.3	3.8	78	12.4%	-0.08 [-0.40, 0.24]
Garvey 2015	9.5	4.71	22	9.09	4.96	22	4.9%	0.08 [-0.51, 0.67]
Kroenke 2009	5.8	5	123	8	5.1	127	16.2%	-0.43 [-0.69, -0.18]
Markle-Reid 2018	2.34	2.92	77	2.71	3.27	66	11.9%	-0.12 [-0.45, 0.21]
Miklavcic 2020	2.37	3.32	63	3.24	3.68	50	10.1%	-0.25 [-0.62, 0.12]
Ross 2005	31.73	22.29	15	57.56	31.56	9	2.4%	-0.96 [-1.84, -0.08]
Salisbury 2018	5.7	4.6	652	6.1	4.7	638	27.4%	-0.09 [-0.20, 0.02]
**Total (95% CI)**			**1105**			**1057**	**100.0%**	**-0.14 [-0.28, 0.00]**

Heterogeneity: $Tau^2 = 0.02$; $Chi^2 = 13.27$, df = 8 ($P = 0.10$); $I^2 = 40\%$
Test for overall effect: Z = 1.95 (P = 0.05)

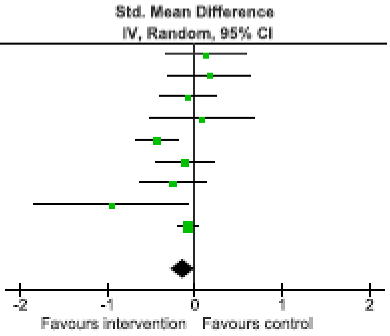

Emotional wellbeing – post-intervention overall effect

Study or Subgroup	Intervention Mean	Intervention SD	Intervention Total	Control Mean	Control SD	Control Total	Weight	Std. Mean Difference IV, Random, 95% CI
Barley 2014	32.4	11.2	35	35.4	11.8	39	11.9%	-0.26 [-0.72, 0.20]
Druss 2018	34.49	11.15	198	34.25	11.97	202	16.4%	0.02 [-0.18, 0.22]
Ell 2010	47.31	11.48	193	43.6	12.46	194	16.3%	0.31 [0.11, 0.51]
Ell 2017	44.54	1.03	178	43.67	1.02	170	16.0%	0.85 [0.63, 1.07]
Goldberg 2013	50.5	11.1	28	43.2	10.7	30	10.7%	0.66 [0.13, 1.19]
Miklavcic 2020	56.69	8.37	65	53.88	7.67	56	13.6%	0.35 [-0.01, 0.71]
Sajatovic 2017	42.05	11.1	100	39.58	11.4	100	15.1%	0.22 [-0.06, 0.50]
**Total (95% CI)**			**797**			**791**	**100.0%**	**0.31 [0.04, 0.58]**

Heterogeneity: $Tau^2 = 0.11$; $Chi^2 = 39.32$, df = 6 ($P < 0.00001$); $I^2 = 85\%$
Test for overall effect: Z = 2.23 (P = 0.03)

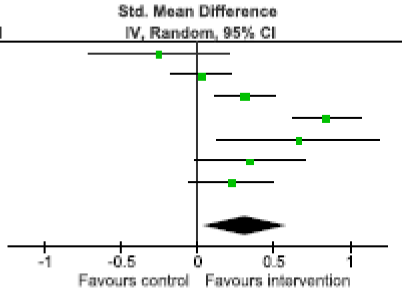

Emotional wellbeing – maintenance overall effect

	Intervention			Control				Std. Mean Difference
Study or Subgroup	Mean	SD	Total	Mean	SD	Total	Weight	IV, Random, 95% CI
Barley 2014	34.5	11.6	32	33.6	12.5	37	10.5%	0.07 [-0.40, 0.55]
Druss 2018	36.64	12.28	198	34.54	11.82	202	18.7%	0.17 [-0.02, 0.37]
Ell 2010	45.1	12.19	193	43.49	11.66	194	18.6%	0.13 [-0.06, 0.33]
Ell 2017	31.24	0.87	178	30.59	0.89	170	18.0%	0.74 [0.52, 0.95]
Goldberg 2013	46.9	10.7	28	43.9	11.5	29	9.4%	0.27 [-0.26, 0.79]
Griva 2019	2.38	0.72	20	2.15	0.63	24	7.9%	0.34 [-0.26, 0.93]
Kanwal 2018	41.8	13.8	114	39.4	11.9	128	16.9%	0.19 [-0.07, 0.44]
**Total (95% CI)**			**763**			**784**	**100.0%**	**0.28 [0.07, 0.49]**

Heterogeneity: Tau² = 0.05; Chi² = 21.42, df = 6 (P = 0.002); I² = 72%
Test for overall effect: Z = 2.63 (P = 0.009)

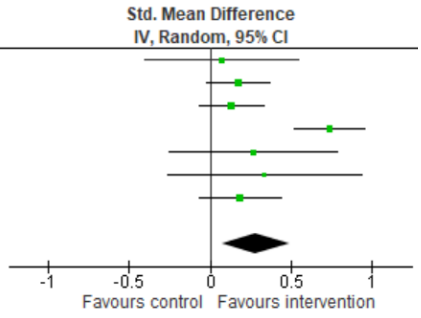

For peer review only

Table 3 significant effects forest plots

Quality of life – post-intervention by intervention type

Study or Subgroup	Intervention Mean	Intervention SD	Intervention Total	Control Mean	Control SD	Control Total	Weight	Std. Mean Difference IV, Random, 95% CI
**1.1.1 Self-management**								
Battersby 2013	0.38	0.19	44	0.43	0.22	28	8.6%	-0.24 [-0.72, 0.23]
Garvey 2015	65.73	20.18	22	50.5	16.3	22	5.9%	0.82 [0.20, 1.43]
**Subtotal (95% CI)**			**66**			**50**	**14.5%**	**0.27 [-0.77, 1.30]**
Heterogeneity: $Tau^2 = 0.48$; $Chi^2 = 7.11$, df = 1 (P = 0.008); $I^2 = 86\%$								
Test for overall effect: Z = 0.50 (P = 0.62)								
**1.1.2 Collaborative care**								
Goorden 2017	0.54	0.33	42	0.5	0.3	39	9.6%	0.13 [-0.31, 0.56]
Kamradt 2019	0.715	0.22	195	0.708	0.23	187	19.2%	0.03 [-0.17, 0.23]
Katon 2010	6	2.2	105	5.2	1.9	106	15.7%	0.39 [0.12, 0.66]
Salisbury 2018	0.566	0.294	699	0.526	0.306	684	23.9%	0.13 [0.03, 0.24]
**Subtotal (95% CI)**			**1041**			**1016**	**68.4%**	**0.15 [0.02, 0.27]**
Heterogeneity: $Tau^2 = 0.01$; $Chi^2 = 4.33$, df = 3 (P = 0.23); $I^2 = 31\%$								
Test for overall effect: Z = 2.33 (P = 0.02)								
**1.1.3 Cognitive and behavioural activation**								
Boeschoten 2017	0.52	0.3	74	0.58	0.3	78	13.7%	-0.20 [-0.52, 0.12]
Ross 2005	5.07	1.2	15	4.25	1.13	9	3.5%	0.67 [-0.18, 1.53]
**Subtotal (95% CI)**			**89**			**87**	**17.1%**	**0.14 [-0.69, 0.98]**
Heterogeneity: $Tau^2 = 0.27$; $Chi^2 = 3.53$, df = 1 (P = 0.06); $I^2 = 72\%$								
Test for overall effect: Z = 0.34 (P = 0.74)								
**Total (95% CI)**			**1196**			**1153**	**100.0%**	**0.13 [-0.04, 0.30]**
Heterogeneity: $Tau^2 = 0.03$; $Chi^2 = 17.13$, df = 7 (P = 0.02); $I^2 = 59\%$								
Test for overall effect: Z = 1.54 (P = 0.12)								
Test for subgroup differences: $Chi^2 = 0.05$, df = 2 (P = 0.98), $I^2 = 0\%$								

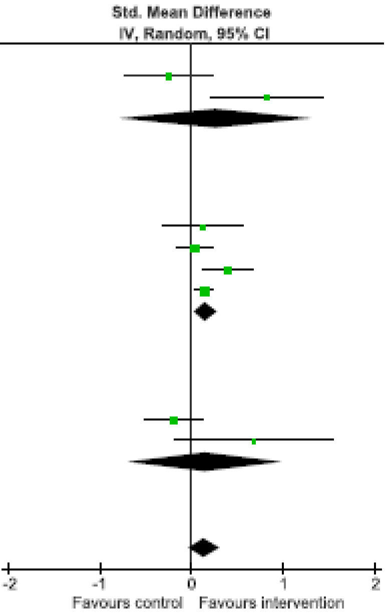

Psychological distress (depression) – post-intervention by intervention type

Study or Subgroup	Intervention Mean	Intervention SD	Intervention Total	Control Mean	Control SD	Control Total	Weight	Std. Mean Difference IV, Random, 95% CI
**1.10.1 Self-management**								
Aragones 2019	1.29	0.77	167	1.42	0.73	161	5.2%	-0.17 [-0.39, 0.04]
Barley 2014	10.3	3.8	35	9.2	4.6	39	4.7%	0.26 [-0.20, 0.71]
Ell 2017	1.02	0.07	178	1.15	0.07	170	5.1%	-1.85 [-2.10, -1.60]
Garvey 2015	6.32	4.19	22	7.82	3.83	22	4.3%	-0.37 [-0.96, 0.23]
House 2018	2.4	1.87	30	1.1	1.8	29	4.5%	0.70 [0.17, 1.23]
Markle-Reid 2018	4.67	4.83	71	5.71	5.24	65	5.0%	-0.21 [-0.54, 0.13]
Miklavcic 2020	4.94	4.46	63	5.82	4.93	50	4.9%	-0.19 [-0.56, 0.19]
Park 2014	5.3	3.4	22	7.6	5.8	21	4.3%	-0.48 [-1.09, 0.13]
**Subtotal (95% CI)**			**588**			**557**	**37.8%**	**-0.30 [-0.91, 0.31]**
Heterogeneity: $Tau^2 = 0.72$; $Chi^2 = 155.89$, df = 7 (P < 0.00001); $I^2 = 96\%$								
Test for overall effect: Z = 0.96 (P = 0.34)								
**1.10.2 Collaborative care**								
Ali 2020	0.52	0.38	192	0.82	0.54	202	5.2%	-0.64 [-0.84, -0.44]
Bogner 2008	9.9	10.7	32	19.3	15.2	32	4.5%	-0.71 [-1.21, -0.20]
Katon 2010	0.83	0.68	105	1.14	0.66	106	5.1%	-0.46 [-0.73, -0.19]
Kroenke 2009	1.14	0.69	123	1.69	0.74	127	5.1%	-0.77 [-1.02, -0.51]
Morgan 2013	7.1	0.8	164	9	0.9	146	5.1%	-2.23 [-2.52, -1.95]
Salisbury 2018	6.1	4.4	654	6.6	4.5	641	5.3%	-0.11 [-0.22, -0.00]
**Subtotal (95% CI)**			**1270**			**1254**	**30.3%**	**-0.82 [-1.40, -0.24]**
Heterogeneity: $Tau^2 = 0.50$; $Chi^2 = 197.36$, df = 5 (P < 0.00001); $I^2 = 97\%$								
Test for overall effect: Z = 2.76 (P = 0.006)								
**1.10.3 Cognitive and behavioural activation**								
Boeschoten 2017	20.3	8.8	74	21	9.1	78	5.0%	-0.08 [-0.40, 0.24]
Cummings 2019	6.3	5.9	67	7.9	7	72	5.0%	-0.25 [-0.58, 0.09]
Naik 2019	10.9	6.1	107	12.4	6	80	5.0%	-0.25 [-0.54, 0.04]
Ross 2005	11.8	9.93	15	16.78	7.89	9	3.6%	-0.52 [-1.36, 0.32]
Sajatovic 2017	15.92	10	100	18.55	8.8	100	5.1%	-0.28 [-0.56, 0.00]
Schneider 2016	12.5	14.8	15	12.3	4.1	14	3.9%	0.02 [-0.71, 0.75]
Wilson 2018	17.46	6.16	22	20.5	5.3	25	4.3%	-0.52 [-1.11, 0.06]
**Subtotal (95% CI)**			**400**			**378**	**31.9%**	**-0.24 [-0.38, -0.09]**
Heterogeneity: $Tau^2 = 0.00$; $Chi^2 = 2.88$, df = 6 (P = 0.82); $I^2 = 0\%$								
Test for overall effect: Z = 3.25 (P = 0.001)								
**Total (95% CI)**			**2258**			**2189**	**100.0%**	**-0.45 [-0.73, -0.16]**
Heterogeneity: $Tau^2 = 0.38$; $Chi^2 = 366.51$, df = 20 (P < 0.00001); $I^2 = 95\%$								
Test for overall effect: Z = 3.11 (P = 0.002)								
Test for subgroup differences: $Chi^2 = 3.64$, df = 2 (P = 0.16), $I^2 = 45.1\%$								

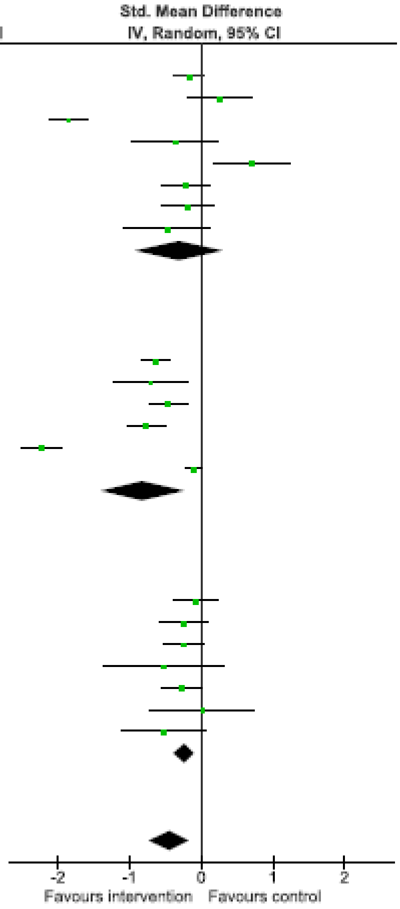

Emotional wellbeing – post-intervention by intervention type

Study or Subgroup	Intervention Mean	Intervention SD	Intervention Total	Control Mean	Control SD	Control Total	Weight	Std. Mean Difference IV, Random, 95% CI
**1.8.1 Self-management**								
Barley 2014	32.4	11.2	35	35.4	11.8	39	11.9%	-0.26 [-0.72, 0.20]
Druss 2018	34.49	11.15	198	34.25	11.97	202	16.4%	0.02 [-0.18, 0.22]
Ell 2017	44.54	1.03	178	43.67	1.02	170	16.0%	0.85 [0.63, 1.07]
Goldberg 2013	50.5	11.1	28	43.2	10.7	30	10.7%	0.66 [0.13, 1.19]
Miklavcic 2020	56.69	8.37	65	53.88	7.67	56	13.6%	0.35 [-0.01, 0.71]
**Subtotal (95% CI)**			**504**			**497**	**68.6%**	**0.33 [-0.10, 0.76]**
Heterogeneity: Tau² = 0.21; Chi² = 38.60, df = 4 (P < 0.00001); I² = 90%								
Test for overall effect: Z = 1.49 (P = 0.14)								
**1.8.2 Collaborative Care**								
Ell 2010	47.31	11.48	193	43.6	12.46	194	16.3%	0.31 [0.11, 0.51]
**Subtotal (95% CI)**			**193**			**194**	**16.3%**	**0.31 [0.11, 0.51]**
Heterogeneity: Not applicable								
Test for overall effect: Z = 3.02 (P = 0.003)								
**1.8.3 Cognitive and behavioural activiation**								
Sajatovic 2017	42.05	11.1	100	39.58	11.4	100	15.1%	0.22 [-0.06, 0.50]
**Subtotal (95% CI)**			**100**			**100**	**15.1%**	**0.22 [-0.06, 0.50]**
Heterogeneity: Not applicable								
Test for overall effect: Z = 1.54 (P = 0.12)								
**Total (95% CI)**			**797**			**791**	**100.0%**	**0.31 [0.04, 0.58]**
Heterogeneity: Tau² = 0.11; Chi² = 39.32, df = 6 (P < 0.00001); I² = 85%								
Test for overall effect: Z = 2.23 (P = 0.03)								
Test for subgroup differences: Chi² = 0.31, df = 2 (P = 0.86), I² = 0%								

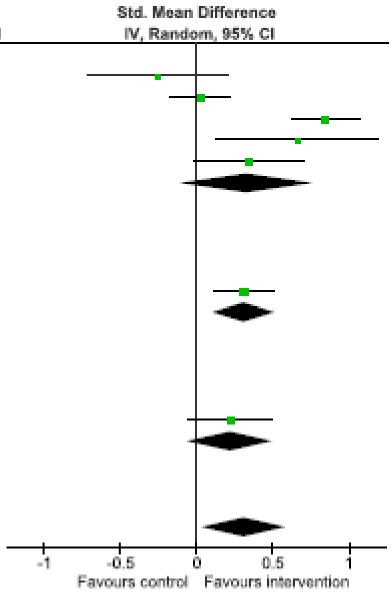

Emotional wellbeing – maintenance by intervention type

Study or Subgroup	Intervention Mean	Intervention SD	Intervention Total	Control Mean	Control SD	Control Total	Weight	Std. Mean Difference IV, Random, 95% CI
**1.9.1 Self-management**								
Barley 2014	34.5	11.6	32	33.6	12.5	37	10.5%	0.07 [-0.40, 0.55]
Druss 2018	36.64	12.28	198	34.54	11.82	202	18.7%	0.17 [-0.02, 0.37]
Ell 2017	31.24	0.87	178	30.59	0.89	170	18.0%	0.74 [0.52, 0.95]
Goldberg 2013	46.9	10.7	28	43.9	11.5	29	9.4%	0.27 [-0.26, 0.79]
Griva 2019	2.38	0.72	20	2.15	0.63	24	7.9%	0.34 [-0.26, 0.93]
**Subtotal (95% CI)**			**456**			**462**	**64.5%**	**0.34 [0.03, 0.65]**
Heterogeneity: Tau² = 0.09; Chi² = 16.42, df = 4 (P = 0.003); I² = 76%								
Test for overall effect: Z = 2.14 (P = 0.03)								
**1.9.2 Collaborative care**								
Ell 2010	45.1	12.19	193	43.49	11.66	194	18.6%	0.13 [-0.06, 0.33]
Kanwal 2018	41.8	13.8	114	39.4	11.9	128	16.9%	0.19 [-0.07, 0.44]
**Subtotal (95% CI)**			**307**			**322**	**35.5%**	**0.15 [-0.00, 0.31]**
Heterogeneity: Tau² = 0.00; Chi² = 0.10, df = 1 (P = 0.75); I² = 0%								
Test for overall effect: Z = 1.93 (P = 0.05)								
**1.9.3 Cognitive and behavioural activiation**								
**Subtotal (95% CI)**			**0**			**0**		**Not estimable**
Heterogeneity: Not applicable								
Test for overall effect: Not applicable								
**Total (95% CI)**			**763**			**784**	**100.0%**	**0.28 [0.07, 0.49]**
Heterogeneity: Tau² = 0.05; Chi² = 21.42, df = 6 (P = 0.002); I² = 72%								
Test for overall effect: Z = 2.63 (P = 0.009)								
Test for subgroup differences: Chi² = 1.09, df = 1 (P = 0.30), I² = 7.9%								

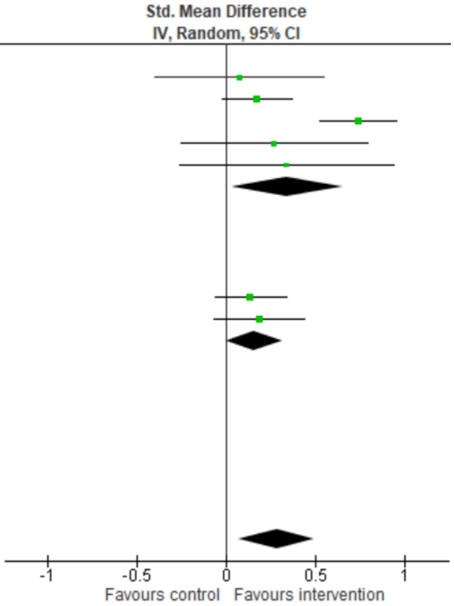

Psychological distress (depression) – maintenance by intervention type

Study or Subgroup	Intervention Mean	Intervention SD	Intervention Total	Control Mean	Control SD	Control Total	Weight	Std. Mean Difference IV, Random, 95% CI
**1.11.1 Self-management**								
Aragones 2019	1.11	0.72	167	1.34	0.76	161	13.3%	-0.31 [-0.53, -0.09]
Barley 2014	9.5	4.6	32	8.8	4.8	37	11.1%	0.15 [-0.33, 0.62]
Ell 2017	1.69	0.06	178	1.63	0.06	170	13.3%	1.00 [0.77, 1.22]
Griva 2019	5.3	4.22	20	4.22	3.88	24	9.9%	0.26 [-0.33, 0.86]
**Subtotal (95% CI)**			**397**			**392**	**47.7%**	**0.28 [-0.48, 1.04]**
Heterogeneity: Tau² = 0.56; Chi² = 68.16, df = 3 (P < 0.00001); I² = 96%								
Test for overall effect: Z = 0.71 (P = 0.47)								
**1.11.2 Collaborative care**								
Ali 2020	0.39	0.27	185	0.53	0.37	193	13.4%	-0.43 [-0.63, -0.23]
Salisbury 2018	6.1	4.6	630	6.8	4.6	625	13.9%	-0.15 [-0.26, -0.04]
**Subtotal (95% CI)**			**815**			**818**	**27.3%**	**-0.28 [-0.55, -0.01]**
Heterogeneity: Tau² = 0.03; Chi² = 5.50, df = 1 (P = 0.02); I² = 82%								
Test for overall effect: Z = 2.01 (P = 0.04)								
**1.11.3 Cognitive and behavioural activiation**								
Boeschoten 2017	20.8	10.4	64	20.1	9.2	67	12.4%	0.07 [-0.27, 0.41]
Naik 2019	10.1	6.9	96	12.6	6.5	70	12.6%	-0.37 [-0.68, -0.06]
**Subtotal (95% CI)**			**160**			**137**	**25.0%**	**-0.16 [-0.59, 0.28]**
Heterogeneity: Tau² = 0.07; Chi² = 3.48, df = 1 (P = 0.06); I² = 71%								
Test for overall effect: Z = 0.71 (P = 0.48)								
**Total (95% CI)**			**1372**			**1347**	**100.0%**	**0.02 [-0.33, 0.36]**
Heterogeneity: Tau² = 0.22; Chi² = 113.01, df = 7 (P < 0.00001); I² = 94%								
Test for overall effect: Z = 0.10 (P = 0.92)								
Test for subgroup differences: Chi² = 1.87, df = 2 (P = 0.39), I² = 0%								

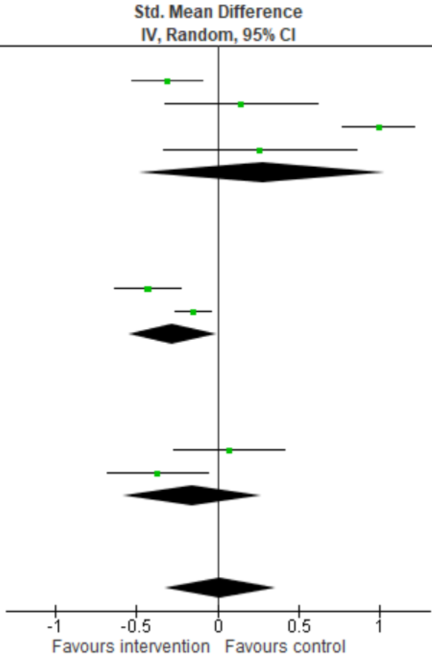

For peer review only

Table 4 significant effects forest plots

Clinical endpoints – post-intervention by intervention length

Study or Subgroup	Intervention Mean	Intervention SD	Intervention Total	Control Mean	Control SD	Control Total	Weight	Std. Mean Difference IV, Random, 95% CI
**1.3.1 <3months**								
**Subtotal (95% CI)**			**0**			**0**		**Not estimable**
Heterogeneity: Not applicable								
Test for overall effect: Not applicable								
**1.3.2 3-6months**								
House 2018	7.1	1.24	37	7.1	1.79	38	8.1%	0.00 [-0.45, 0.45]
Morgan 2013	6.9	0.26	89	7.4	0.36	67	8.9%	-1.62 [-1.99, -1.26]
Naik 2019	9.1	1.7	119	8.7	1.7	82	9.6%	0.23 [-0.05, 0.52]
Schneider 2016	7.8	0.9	15	7.7	0.8	14	5.7%	0.11 [-0.62, 0.84]
**Subtotal (95% CI)**			**260**			**201**	**32.2%**	**-0.33 [-1.30, 0.65]**
Heterogeneity: $Tau^2 = 0.93$; $Chi^2 = 66.74$, df = 3 ($P < 0.00001$); $I^2 = 96\%$								
Test for overall effect: Z = 0.66 (P = 0.51)								
**1.3.3 >6months**								
Ali 2020	8.19	1.8	192	8.44	1.82	202	10.2%	-0.14 [-0.34, 0.06]
Cummings 2019	8.96	2.1	67	9.04	2.2	72	9.1%	-0.04 [-0.37, 0.30]
Ell 2010	8.52	2.01	193	8.59	2.26	194	10.2%	-0.03 [-0.23, 0.17]
Jackson 2021	137.8	17.4	91	148.6	20.1	91	9.5%	-0.57 [-0.87, -0.28]
Katon 2010	131	18.2	105	132.3	17.4	106	9.7%	-0.07 [-0.34, 0.20]
McDermott 2015	9.8	2.3	84	10.3	2.2	105	9.5%	-0.22 [-0.51, 0.07]
Sajatovic 2017	7.69	1.9	100	7.77	2	100	9.6%	-0.04 [-0.32, 0.24]
**Subtotal (95% CI)**			**832**			**870**	**67.8%**	**-0.15 [-0.28, -0.02]**
Heterogeneity: $Tau^2 = 0.01$; $Chi^2 = 10.68$, df = 6 ($P = 0.10$); $I^2 = 44\%$								
Test for overall effect: Z = 2.25 (P = 0.02)								
**Total (95% CI)**			**1092**			**1071**	**100.0%**	**-0.22 [-0.47, 0.03]**
Heterogeneity: $Tau^2 = 0.15$; $Chi^2 = 80.56$, df = 10 ($P < 0.00001$); $I^2 = 88\%$								
Test for overall effect: Z = 1.72 (P = 0.09)								
Test for subgroup differences: $Chi^2 = 0.13$, df = 1 ($P = 0.72$), $I^2 = 0\%$								

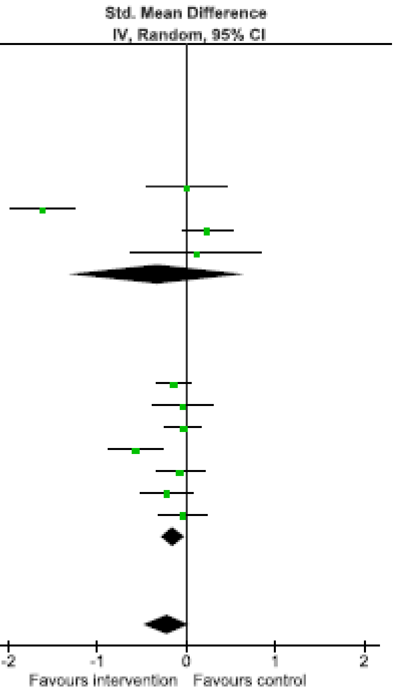

review only

Psychological distress (depression) – post-intervention by intervention length

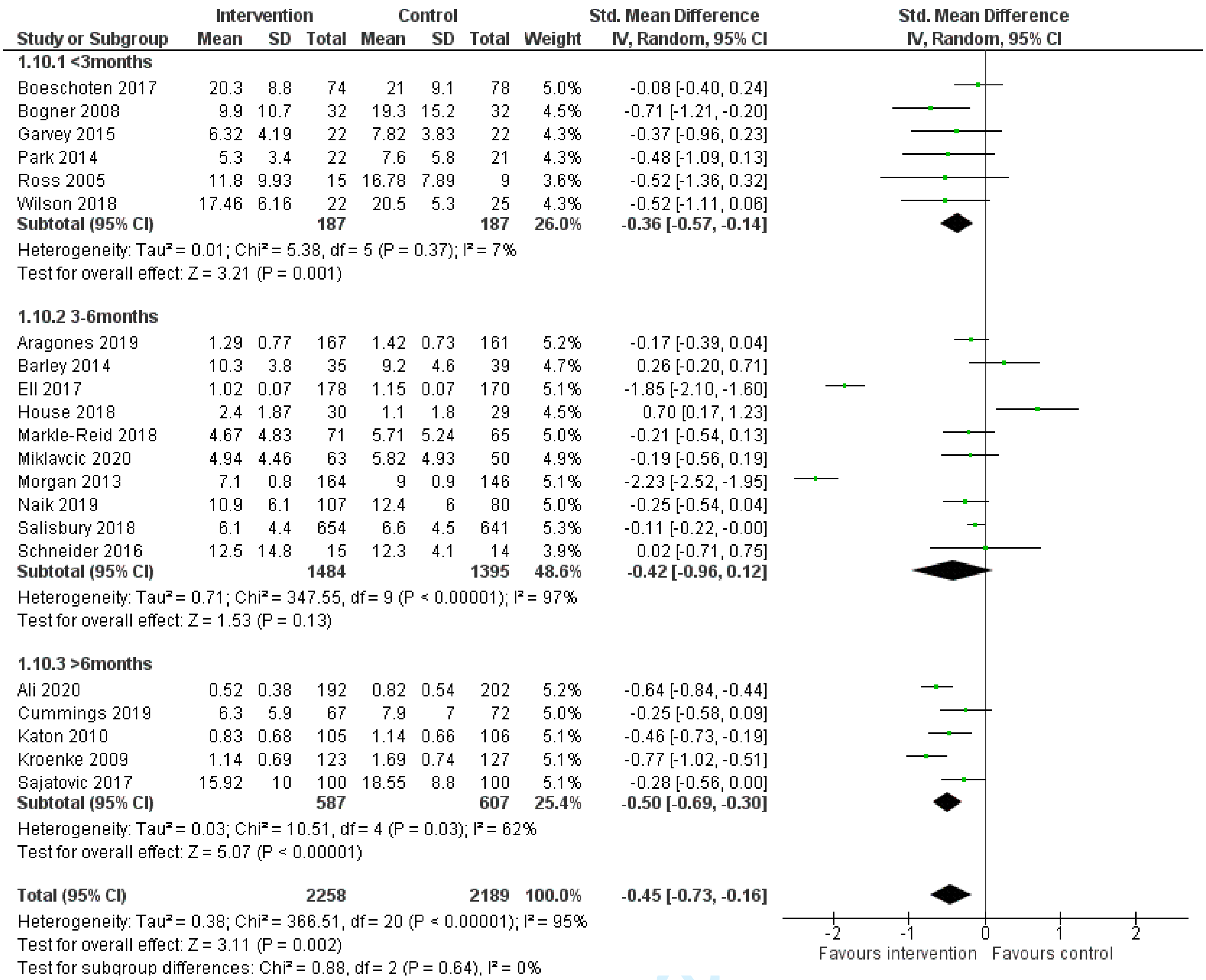

Emotional wellbeing – post-intervention by intervention length

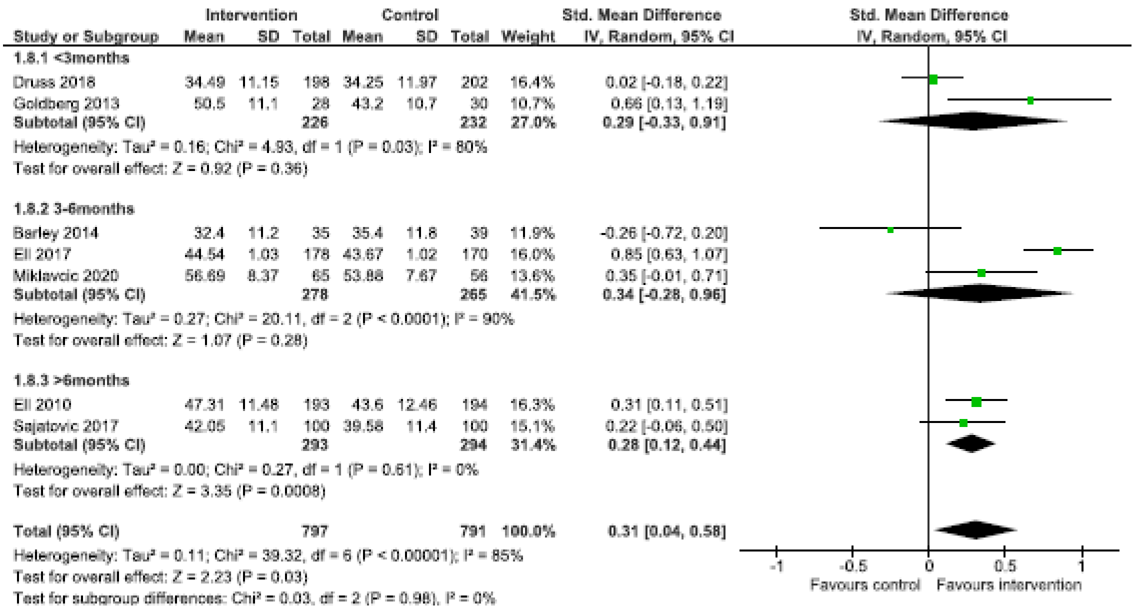

Clinical endpoints – maintenance by intervention length

Study or Subgroup	Intervention Mean	Intervention SD	Intervention Total	Control Mean	Control SD	Control Total	Weight	Std. Mean Difference IV, Random, 95% CI
**1.4.1 <3months**								
Bogner 2008	127.3	17.7	32	141.3	18.8	32	11.1%	-0.76 [-1.27, -0.25]
Griva 2019	8.78	1.54	20	9.5	2.05	24	8.7%	-0.38 [-0.98, 0.21]
**Subtotal (95% CI)**			**52**			**56**	**19.8%**	**-0.60 [-0.99, -0.21]**
Heterogeneity: Tau² = 0.00; Chi² = 0.86, df = 1 (P = 0.35); I² = 0%								
Test for overall effect: Z = 3.04 (P = 0.002)								
**1.4.2 3-6months**								
Naik 2019	8.7	1.6	108	8.9	2	78	21.9%	-0.11 [-0.40, 0.18]
**Subtotal (95% CI)**			**108**			**78**	**21.9%**	**-0.11 [-0.40, 0.18]**
Heterogeneity: Not applicable								
Test for overall effect: Z = 0.75 (P = 0.45)								
**1.4.3 >6months**								
Ali 2020	8.73	1.79	185	8.71	1.69	193	29.0%	0.01 [-0.19, 0.21]
Ell 2010	8.34	2.04	193	8.5	2.17	194	29.2%	-0.08 [-0.28, 0.12]
**Subtotal (95% CI)**			**378**			**387**	**58.3%**	**-0.03 [-0.17, 0.11]**
Heterogeneity: Tau² = 0.00; Chi² = 0.36, df = 1 (P = 0.55); I² = 0%								
Test for overall effect: Z = 0.45 (P = 0.65)								
**Total (95% CI)**			**538**			**521**	**100.0%**	**-0.16 [-0.36, 0.04]**
Heterogeneity: Tau² = 0.02; Chi² = 8.53, df = 4 (P = 0.07); I² = 53%								
Test for overall effect: Z = 1.59 (P = 0.11)								
Test for subgroup differences: Chi² = 7.30, df = 2 (P = 0.03), I² = 72.6%								

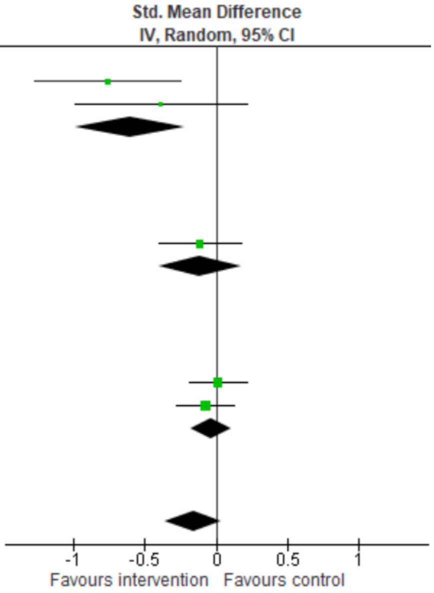

Psychological distress (depression) – maintenance by intervention length

Study or Subgroup	Intervention Mean	Intervention SD	Intervention Total	Control Mean	Control SD	Control Total	Weight	Std. Mean Difference IV, Random, 95% CI
**1.11.1 <3months**								
Boeschoten 2017	20.8	10.4	64	20.1	9.2	67	12.4%	0.07 [-0.27, 0.41]
Griva 2019	5.3	4.22	20	4.22	3.88	24	9.9%	0.26 [-0.33, 0.86]
**Subtotal (95% CI)**			**84**			**91**	**22.3%**	**0.12 [-0.18, 0.42]**
Heterogeneity: Tau² = 0.00; Chi² = 0.30, df = 1 (P = 0.58); I² = 0%								
Test for overall effect: Z = 0.78 (P = 0.43)								
**1.11.2 3-6months**								
Barley 2014	9.5	4.6	32	8.8	4.8	37	11.1%	0.15 [-0.33, 0.62]
Ell 2017	1.69	0.06	178	1.63	0.06	170	13.3%	1.00 [0.77, 1.22]
Naik 2019	10.1	6.9	96	12.6	6.5	70	12.6%	-0.37 [-0.68, -0.06]
**Subtotal (95% CI)**			**306**			**277**	**37.1%**	**0.26 [-0.67, 1.20]**
Heterogeneity: Tau² = 0.65; Chi² = 51.33, df = 2 (P < 0.00001); I² = 96%								
Test for overall effect: Z = 0.55 (P = 0.58)								
**1.11.3 >6months**								
Ali 2020	0.39	0.27	185	0.53	0.37	193	13.4%	-0.43 [-0.63, -0.23]
Aragones 2019	1.11	0.72	167	1.34	0.76	161	13.3%	-0.31 [-0.53, -0.09]
Salisbury 2018	6.1	4.6	630	6.8	4.6	625	13.9%	-0.15 [-0.26, -0.04]
**Subtotal (95% CI)**			**982**			**979**	**40.6%**	**-0.28 [-0.46, -0.10]**
Heterogeneity: Tau² = 0.02; Chi² = 6.11, df = 2 (P = 0.05); I² = 67%								
Test for overall effect: Z = 3.11 (P = 0.002)								
**Total (95% CI)**			**1372**			**1347**	**100.0%**	**0.02 [-0.33, 0.36]**
Heterogeneity: Tau² = 0.22; Chi² = 113.01, df = 7 (P < 0.00001); I² = 94%								
Test for overall effect: Z = 0.10 (P = 0.92)								
Test for subgroup differences: Chi² = 5.95, df = 2 (P = 0.05), I² = 66.4%								

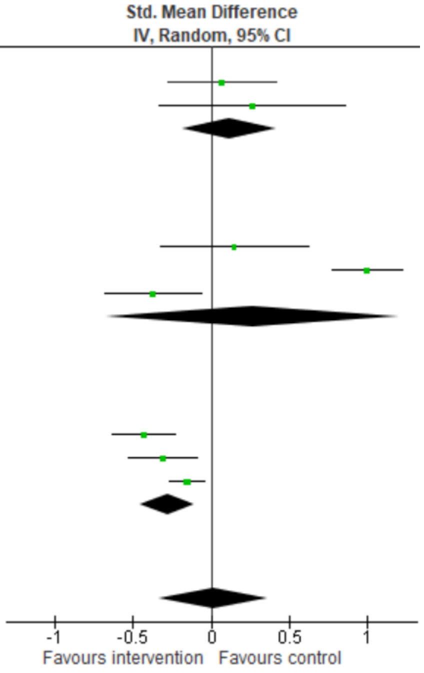

Emotional wellbeing – maintenance by intervention length

Study or Subgroup	Intervention Mean	Intervention SD	Intervention Total	Control Mean	Control SD	Control Total	Weight	Std. Mean Difference IV, Random, 95% CI
**1.9.1 <3months**								
Goldberg 2013	46.9	10.7	28	43.9	11.5	29	9.4%	0.27 [-0.26, 0.79]
Griva 2019	2.38	0.72	20	2.15	0.63	24	7.9%	0.34 [-0.26, 0.93]
**Subtotal (95% CI)**			**48**			**53**	**17.3%**	**0.30 [-0.10, 0.69]**
Heterogeneity: Tau² = 0.00; Chi² = 0.03, df = 1 (P = 0.86); I² = 0%								
Test for overall effect: Z = 1.48 (P = 0.14)								
**1.9.2 3-6months**								
Barley 2014	34.5	11.6	32	33.6	12.5	37	10.5%	0.07 [-0.40, 0.55]
Druss 2018	36.64	12.28	198	34.54	11.82	202	18.7%	0.17 [-0.02, 0.37]
Ell 2010	45.1	12.19	193	43.49	11.66	194	18.6%	0.13 [-0.06, 0.33]
Ell 2017	31.24	0.87	178	30.59	0.89	170	18.0%	0.74 [0.52, 0.95]
Kanwal 2018	41.8	13.8	114	39.4	11.9	128	16.9%	0.19 [-0.07, 0.44]
**Subtotal (95% CI)**			**715**			**731**	**82.7%**	**0.28 [0.02, 0.53]**
Heterogeneity: Tau² = 0.06; Chi² = 21.39, df = 4 (P = 0.0003); I² = 81%								
Test for overall effect: Z = 2.15 (P = 0.03)								
**1.9.3 >6months**								
**Subtotal (95% CI)**			**0**			**0**		**Not estimable**
Heterogeneity: Not applicable								
Test for overall effect: Not applicable								
**Total (95% CI)**			**763**			**784**	**100.0%**	**0.28 [0.07, 0.49]**
Heterogeneity: Tau² = 0.05; Chi² = 21.42, df = 6 (P = 0.002); I² = 72%								
Test for overall effect: Z = 2.63 (P = 0.009)								
Test for subgroup differences: Chi² = 0.01, df = 1 (P = 0.93), I² = 0%								

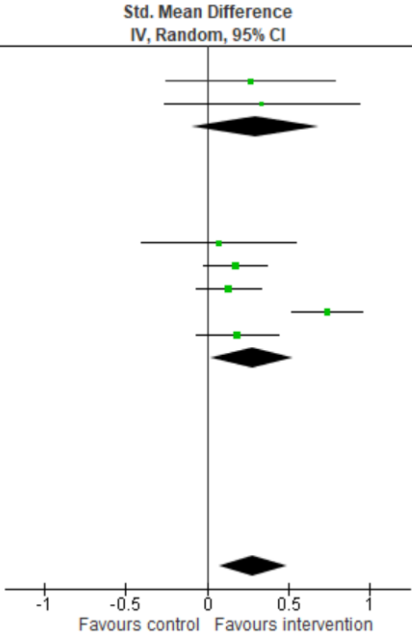

For peer review only

Supplementary table 4 significant effects forest plots

Psychological distress (depression) – post-intervention MLTCs combination type

Study or Subgroup	Intervention Mean	Intervention SD	Intervention Total	Control Mean	Control SD	Control Total	Weight	Std. Mean Difference IV, Random, 95% CI
**1.10.1 Physical-Physical**								
Garvey 2015	6.32	4.19	22	7.82	3.83	22	4.3%	-0.37 [-0.96, 0.23]
House 2018	2.4	1.87	30	1.1	1.8	29	4.5%	0.70 [0.17, 1.23]
Markle-Reid 2018	4.67	4.83	71	5.71	5.24	65	5.0%	-0.21 [-0.54, 0.13]
Miklavcic 2020	4.94	4.46	63	5.82	4.93	50	4.9%	-0.19 [-0.56, 0.19]
Park 2014	5.3	3.4	22	7.6	5.8	21	4.3%	-0.48 [-1.09, 0.13]
Salisbury 2018	6.1	4.4	654	6.6	4.5	641	5.3%	-0.11 [-0.22, -0.00]
**Subtotal (95% CI)**			**862**			**828**	**28.2%**	**-0.11 [-0.34, 0.12]**
Heterogeneity: Tau² = 0.04; Chi² = 11.64, df = 5 (P = 0.04); I² = 57%								
Test for overall effect: Z = 0.93 (P = 0.35)								
**1.10.2 Physical-Mental**								
Ali 2020	0.52	0.38	192	0.82	0.54	202	5.2%	-0.64 [-0.84, -0.44]
Aragones 2019	1.29	0.77	167	1.42	0.73	161	5.2%	-0.17 [-0.39, 0.04]
Barley 2014	10.3	3.8	35	9.2	4.6	39	4.7%	0.26 [-0.20, 0.71]
Boeschoten 2017	20.3	8.8	74	21	9.1	78	5.0%	-0.08 [-0.40, 0.24]
Bogner 2008	9.9	10.7	32	19.3	15.2	32	4.5%	-0.71 [-1.21, -0.20]
Cummings 2019	6.3	5.9	67	7.9	7	72	5.0%	-0.25 [-0.58, 0.09]
Ell 2017	1.02	0.07	178	1.15	0.07	170	5.1%	-1.85 [-2.10, -1.60]
Katon 2010	0.83	0.68	105	1.14	0.66	106	5.1%	-0.46 [-0.73, -0.19]
Kroenke 2009	1.14	0.69	123	1.69	0.74	127	5.1%	-0.77 [-1.02, -0.51]
Morgan 2013	7.1	0.8	164	9	0.9	146	5.1%	-2.23 [-2.52, -1.95]
Naik 2019	10.9	6.1	107	12.4	6	80	5.0%	-0.25 [-0.54, 0.04]
Ross 2005	11.8	9.93	15	16.78	7.89	9	3.6%	-0.52 [-1.36, 0.32]
Sajatovic 2017	15.92	10	100	18.55	8.8	100	5.1%	-0.28 [-0.56, 0.00]
Schneider 2016	12.5	14.8	15	12.3	4.1	14	3.9%	0.02 [-0.71, 0.75]
Wilson 2018	17.46	6.16	22	20.5	5.3	25	4.3%	-0.52 [-1.11, 0.06]
**Subtotal (95% CI)**			**1396**			**1361**	**71.8%**	**-0.58 [-0.94, -0.21]**
Heterogeneity: Tau² = 0.47; Chi² = 278.61, df = 14 (P < 0.00001); I² = 95%								
Test for overall effect: Z = 3.10 (P = 0.002)								
**1.10.3 Mental-Mental**								
**Subtotal (95% CI)**			**0**			**0**		**Not estimable**
Heterogeneity: Not applicable								
Test for overall effect: Not applicable								
**Total (95% CI)**			**2258**			**2189**	**100.0%**	**-0.45 [-0.73, -0.16]**
Heterogeneity: Tau² = 0.38; Chi² = 366.51, df = 20 (P < 0.00001); I² = 95%								
Test for overall effect: Z = 3.11 (P = 0.002)								
Test for subgroup differences: Chi² = 4.57, df = 1 (P = 0.03), I² = 78.1%								

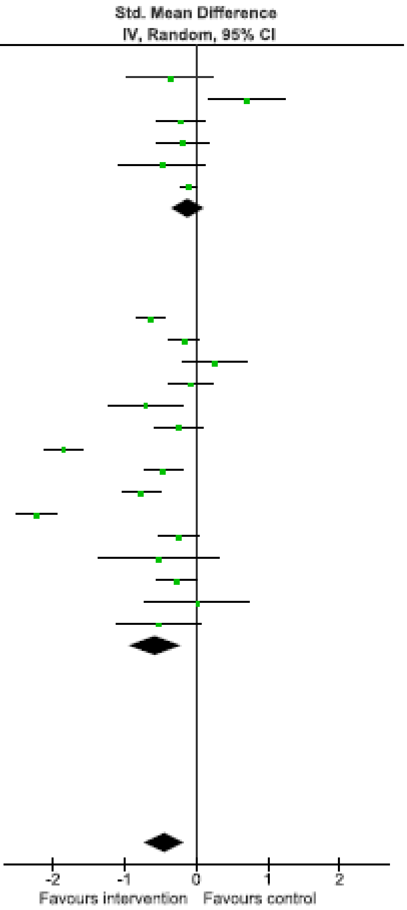

For peer review only
